# Exploiting 3-Oxidopyraziniums toward Diazabicyclo[3.2.1]octanes and Their Conversion into Diazabicyclo[2.2.2]octanes and Tricyclic Lactone-Lactams

**DOI:** 10.1021/acs.joc.3c02273

**Published:** 2024-02-08

**Authors:** Gerard Riesco-Llach, Marta Planas, Lidia Feliu, John A. Joule

**Affiliations:** † LIPPSO, Department of Chemistry, Maria Aurèlia Capmany 69, Universitat de Girona, 17003 Girona, Spain; ‡ The School of Chemistry, The University of Manchester, Manchester M13 9PL, U.K.

## Abstract

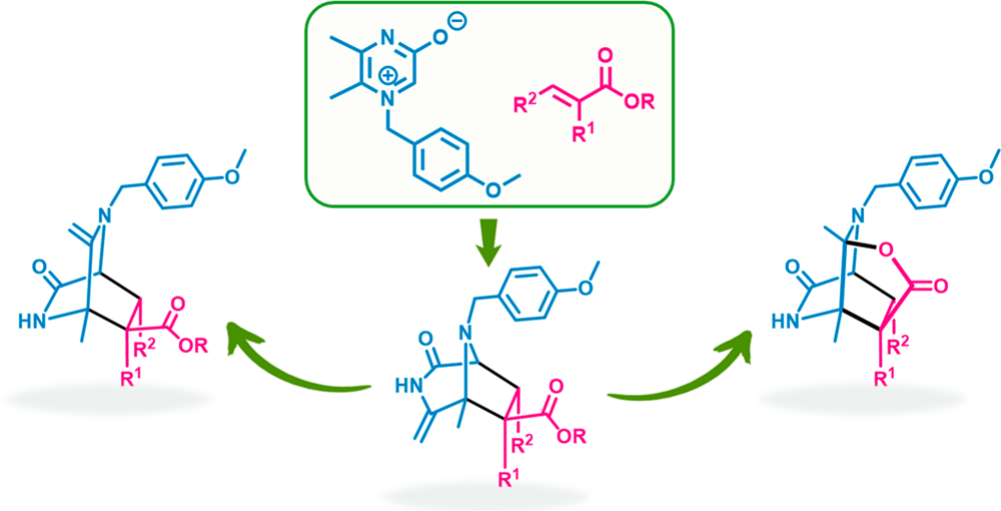

3-Oxidopyraziniums are azomethine ylides derived from 2(1*H*)-pyrazinones that can undergo 1,3-dipolar cycloadditions with acrylate and acrylic acid derivatives. The cycloaddition of 1-(4-methoxybenzyl)-5,6-dimethyl-3-oxidopyrazinium with methyl and *tert*-butyl acrylate and with methyl crotonate afforded a 3,8-diazabicyclo[3.2.1]octane in 51–73% yield together with traces of the 2,5-diazabicyclo[2.2.2]octane. In contrast, cycloaddition of this 3-oxidopyrazinium with methyl 2-phenyl acrylate provided the [2.2.2] product in 40% yield. Herein, we show that the 2,5-diazabicyclo[2.2.2]octanes were formed from the [3.2.1] compounds via a Wagner–Meerwein rearrangement. Remarkably, when acrylic acid and 2-phenylacrylic acid were employed as dipolarophiles, novel tricyclic fused lactone-lactam systems were obtained in 71% and 50% yields, respectively. The formation of these tricyclic compounds can be rationalized via the mechanism described above followed by lactonization of the 2,5-diazabicyclo[2.2.2]octane.

## Introduction

**Scheme 1:**
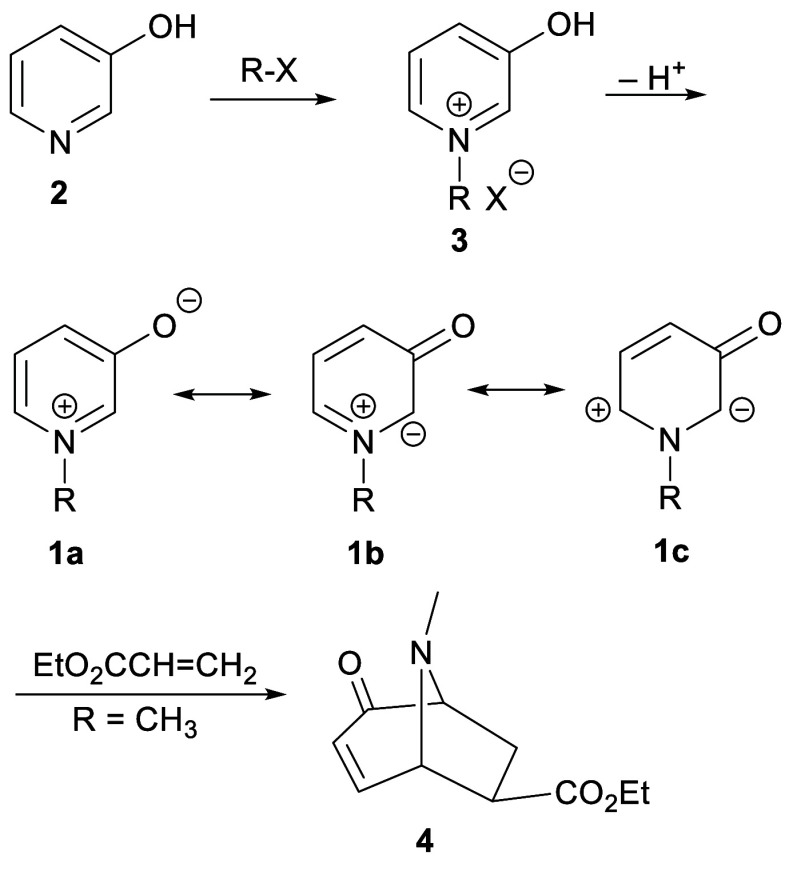
Main Resonance Contributors to 3-Oxidopyridiniums **1** and Reaction of 1-Methyl-3-oxidopyridinium with Ethyl Acrylate

A 1989 review summarized Katritzky’s explorations of the reactivity of zwitterions **1**, which were termed 3-oxidopyridiniums (Scheme 1).^1^ Thus, with R = Me, **1** is 1-methyl-3-oxidopyridinium (*Chemical Abstracts* uses the term “3-hydroxy-1-methylpyridinium, inner salt”). The interesting reactivity of these betaines has attracted the attention of researchers due to their application in the synthesis of natural products.^2,3^ These isolable species were accessed via quaternization of 3-hydroxypyridine (**2**) with an alkyl halide, giving 3-hydroxypyridinium salts **3**, and then O-deprotonation under room-temperature conditions. The chief interest was in the reactivity of 3-oxidopyridiniums **1** as 1,3-dipoles in cycloaddition reactions with alkenes, across C-2 and C-6. Thus, for example, 1-methyl-3-oxidopyridinium reacted with ethyl acrylate producing ethyl 8-methyl-2-oxo-8-azabicyclo[3.2.1]oct-3-ene-6-carboxylate (**4**) (Scheme 1). Note that the regioselectivity of this reaction is determined by the polarization implied by resonance structure **1c**.

**Scheme 2:**
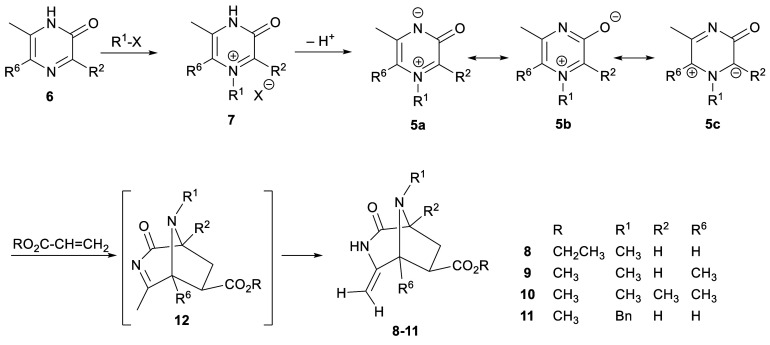
Synthesis of 3-Oxidopyraziniums **5** and Their Reaction with Acrylates

It occurred to us that comparable zwitterions and thence comparable cycloadditions might be possible in an analogous pyrazine series, though an alternative route to 3-oxidopyraziniums **5** would be required (*Chemical Abstracts* uses the term “3,4-dihydro-3-oxopyrazinium, inner salt”) (Scheme 2). This concept was put into practice by accessing 3-oxidopyraziniums **5** from 2(1*H*)-pyrazinones **6**.^4^ Regioselective quaternization of the imine nitrogen of 2(1*H*)-pyrazinones **6** gives pyrazinium salts **7**, and then N-deprotonation reveals 3-oxidopyraziniums **5**. We showed that compounds **5** undergo ready dipolar cycloadditions with typical dipolarophiles like acrylates, acrylonitrile, vinyl sulfones, α,β-unsaturated ketones, and alkynyl esters.^5,6^ The cycloaddition occurred in the regiosense indicated by resonance contributor **5c** and afforded 3,8-diazabicyclo[3.2.1]octanes **8**–**11** with an *exo* carboxylate group. The assumed intermediate products of the cycloadditions, **12** in Scheme 2, were never seen, the isolated material in each case being enamide tautomers **8**–**11**.

**Scheme 3:**
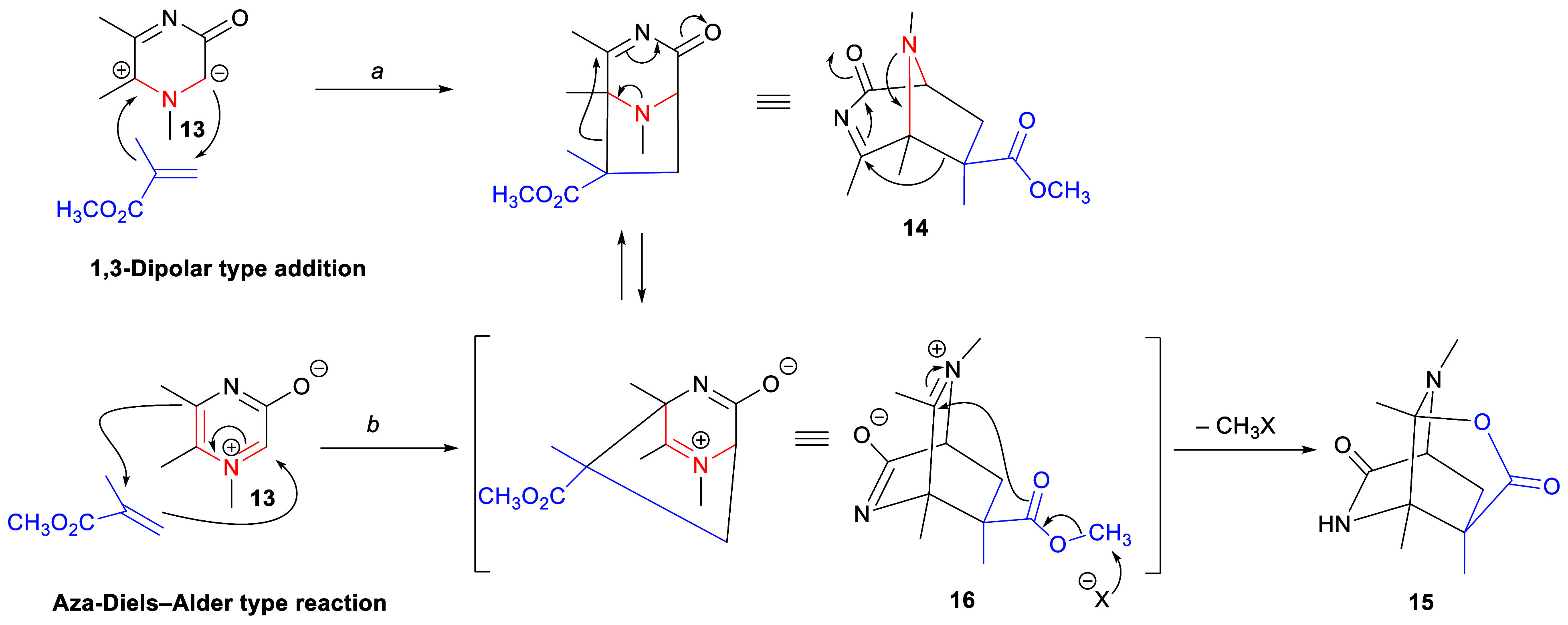
Reaction of 1,5,6-Trimethyl-3-oxidopyrazinium **13** with Methyl Methacrylate Showing the Suggested Sequence of Bond Changes

Density functional theory (DFT) studies^7−9^ of the regio- and stereoselectivity of the 1,3-dipolar cycloaddition of 3-oxidopyraziniums **5** with methyl acrylate were consistent with the experimental results in terms of the regiochemistry of cycloaddition and the stereochemistry of the ester substituent in product 3,8-diazabicyclo[3.2.1]octanes. However, in an experiment designed to test the effect of a more bulky dipolarophile, 1,5,6-trimethyl-3-oxidopyrazinium (**13**) was reacted with methyl methacrylate but did not give the expected 3,8-diazabicyclo[3.2.1]octane **14** (Scheme 3, route *a*). Instead, tricyclic fused lactam-lactone product **15**, derived from a [2.2.2] core,^10^ was obtained. Scheme 3 (route *b*) suggests a plausible mechanistic sequence that would lead to **15**. The central difference from the previously assumed sequence leading to 3,8-diazabicyclo[3.2.1]octanes is that the initial interaction between the acrylate and the 3-oxidopyrazinium is proposed to occur across carbons C-2 and C-5; i.e., it is an aza-Diels–Alder type process: C-5–C-6–N-1–C-2 being the azadiene, yielding 2,5-diazabicyclo[2.2.2]octane **16**. Afterward, an S_N_2 reaction, promoted by a halide anion, with concomitant nucleophilic attack of the created carboxylate anion on an iminium carbon, thus forms the lactone ring.

Alternatively, 2,5-diazabicyclo[2.2.2]octane **16** could be formed via a rearrangement of an initial 3,8-diazabicyclo[3.2.1]octane **14** obtained from a 1,3-dipolar type addition (Scheme 3, route *a*). In fact, this rearrangement might be reversible; i.e., 3,8-diazabicyclo[3.2.1]octane **14** might be formed from 2,5-diazabicyclo[2.2.2]octane **16**. The feasibility of the mechanism of this rearrangement was assessed by the DFT method at the B3LYP/6-31G(d) level.^11^ The conclusion was that formation of lactone-lactam **15** is a domino process involving three consecutive reactions: first a 1,3-dipolar cycloaddition between 3-oxidopyrazinium **13** and methyl methacrylate that yields 3,8-diazabicyclo[3.2.1]octane **14**, next a skeletal rearrangement, which converts this adduct into 2,5-diazabicyclo[2.2.2]octane **16**, and finally lactone ring formation. This theoretical assessment therefore suggests that the 3,8-diazabicyclo[3.2.1]octane products (Scheme 3) are formed directly, and not via an aza-Diels–Alder intermediate.

**Scheme 4:**
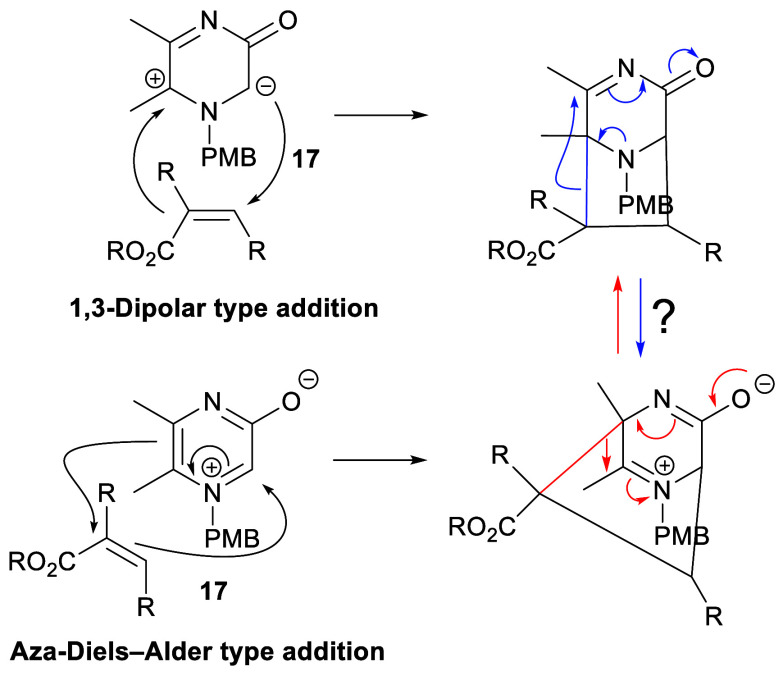
Reactions of 3-Oxidopyrazinium **17** with an Acrylate Derivative and Possible Interconversions of the Corresponding Bicyclic Products

In this work, we have explored the reactions of 1-(4-methoxybenzyl)-5,6-dimethyl-3-oxidopyrazinium (**17**) with acrylate and acrylic acid derivatives with the objective of gaining further insight into the mechanism of formation of 3,8-diazabicyclo[3.2.1]octanes (1,3-dipolar type addition) versus 2,5-diazabicyclo[2.2.2]octanes (aza-Diels–Alder type addition) and their possible interconversion (Scheme 4). The presence of the 4-methoxybenzyl (PMB) group at N-1 improves solubility, facilitates the interpretation of product ^1^H nuclear magnetic resonance (NMR) spectra, and was intended to be a late-stage removable substituent.

## Results and Discussion

### Cycloaddition Reactions of 1-(4-Methoxybenzyl)-5,6-dimethyl-3-oxidopyrazinium **17** with Acrylate Derivatives

**Scheme 5:**
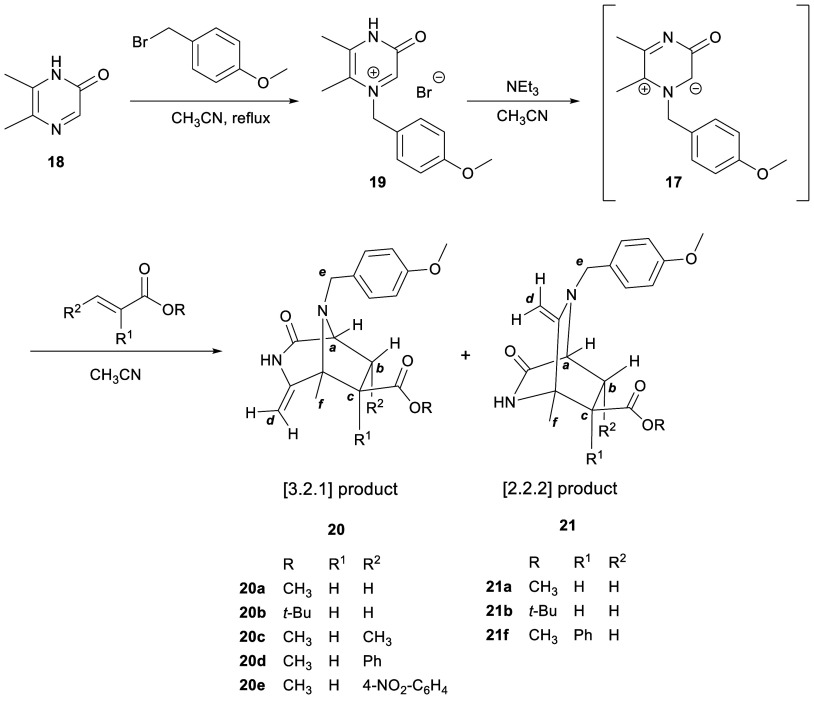
Synthesis of Pyrazinium Bromide **19** and Cycloadditions of 3-Oxidopyrazinium **17** with Acrylates

5,6-Dimethyl-2(1*H*)-pyrazinone (**18**) was prepared by Jones’ method^12^ through the condensation of butane-2,3-dione and glycinamide hydrochloride.^13^ N-1 alkylation with 4-methoxybenzyl bromide was carried out in acetonitrile under reflux for 6 h and then at room temperature overnight (Scheme 5). Purification of the resulting bromide salt by reversed-phase chromatography afforded 1-(4-methoxybenzyl)-5,6-dimethyl-3-oxo-3,4-dihydropyrazin-1-ium bromide (**19**) in 75% yield.

With pyrazinium bromide **19** in hand, we proceeded to explore the cycloaddition reactions of its corresponding 1-(4-methoxybenzyl)-5,6-dimethyl-3-oxidopyrazinium (**17**) with a range of acrylates (Table 1). Unlike our earlier studies, these cycloadditions were performed using a more convenient one-pot procedure, and without isolating the 3-oxidopyrazinium. Thus, a suspension of pyrazinium bromide **19** was treated with triethylamine at room temperature, leading to an orange solution of 3-oxidopyrazinium **17**, to which was added the corresponding dipolarophile, while the mixture was kept at room temperature (Scheme 5). The formation of 3-oxidopyrazinium **17** was checked by electrospray ionization mass spectroscopy (ESI-MS) and ^1^H NMR.

**Table 1:** Reaction of 3-Oxidopyrazinium **17** with Acrylates

entry	R	R^1^	R^2^	*T* (°C)	reaction time (h)	[3.2.1] products [yield (%)]	[2.2.2] products [yield (%)]
1	CH_3_	H	H	rt	0.75	**20a** (73)	**21a** (4)
2	*t-*Bu	H	H	rt	1.5	**20b** (63)	**21b** (7)
3	CH_3_	H	CH_3_	rt	6	**20c** (51)	–
4	CH_3_	H	Ph	rt	72	–	–
5	CH_3_	H	Ph	50	24	–	–
6	CH_3_	H	Ph	80	4	**20d** (13)^a^	–
7	CH_3_	H	4-NO_2_-C_6_H_4_	rt	72	–	–
8	CH_3_	H	4-NO_2_-C_6_H_4_	50	24	**20e** (4)	–
9	CH_3_	H	4-NO_2_-C_6_H_4_	80	4	**20e** (23)^b^	–
10	CH_3_	H	4-NH_2_-C_6_H_4_	rt	72	–	–
11	CH_3_	H	4-NH_2_-C_6_H_4_	50	24	–	–
12	CH_3_	H	4-NH_2_-C_6_H_4_	80	4	–	–
13	CH_3_	Ph	H	rt	5	–	**21f** (40)

a As a mixture of regioisomers **20da** and **20db** in a 3:4 ratio (see Figure 2).

b As a mixture of regioisomers **20ea** and **20eb** in a 3:2 ratio (see Figure 2).

Initially, we explored the reaction of 3-oxidopyrazinium **17** with methyl acrylate in acetonitrile under reflux, which afforded a mixture of adducts together with decomposition products after long reaction times. Next, this reaction was carried out at room temperature for 45 min (Table 1, entry 1), which led to a much cleaner result and the formation of cycloadduct **20a** in 73% yield accompanied by a minor isomer, which was shown to have structure **21a**, in 4% yield. This reaction was also attempted using tetrahydrofuran and CH_2_Cl_2_ as solvents, but they led to poorer results; thus, acetonitrile was the solvent of choice.

**Figure 1:**
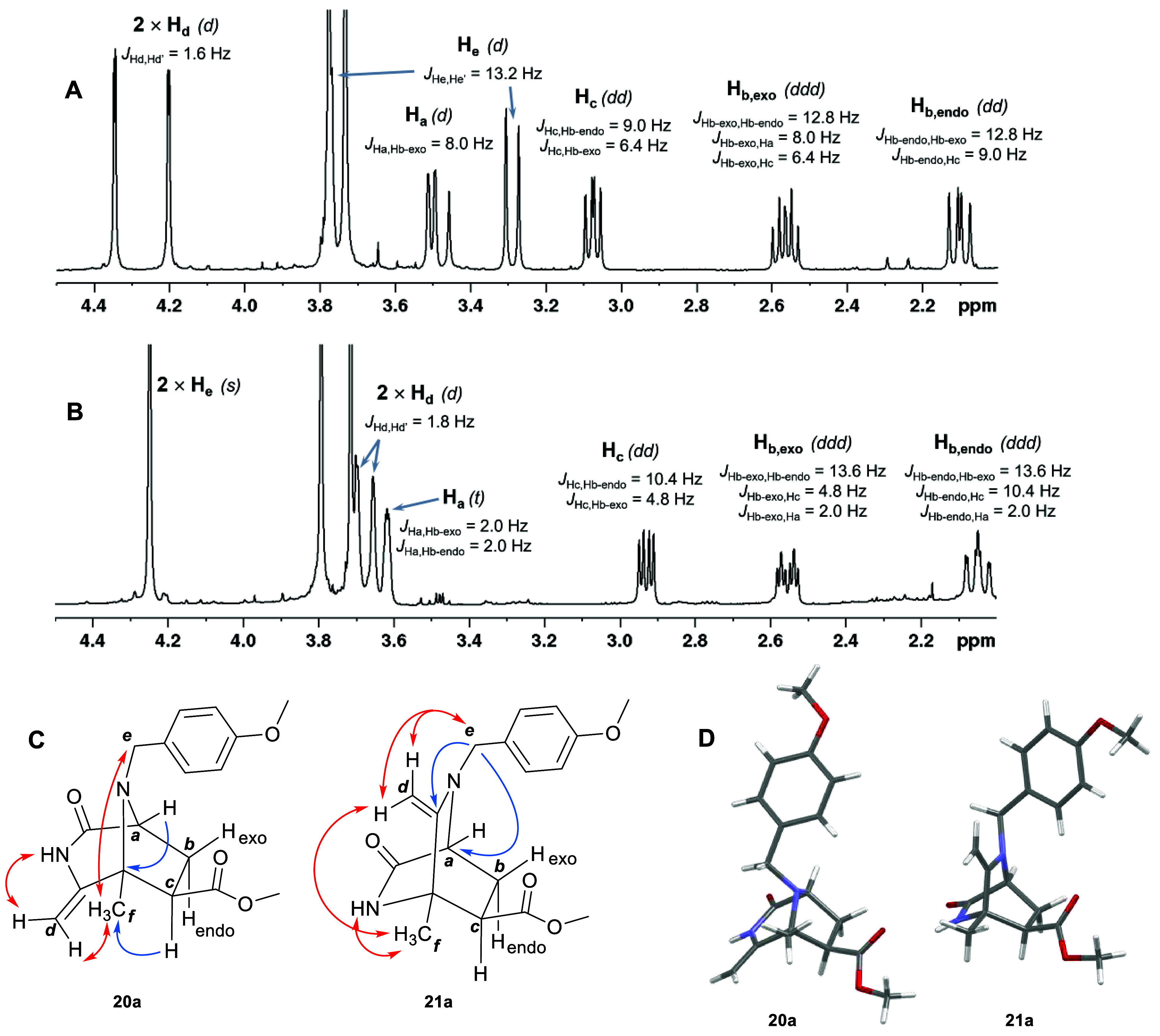
Comparison of the ^1^H NMR spectra between 2.0 and 4.5 ppm showing (A) signals for protons of diazabicyclo[3.2.1]octane **20a**, (B) signals for protons of diazabicyclo[2.2.2]octane **21a**, (C) selected key ^1^H–^13^C HMBC (blue arrows) and ^1^H–^1^H NOESY correlations (red arrows), and (D) a capped-stick representation of the corresponding X-ray crystal structures (see the Supporting Information for the ORTEP representation showing thermal ellipsoids at the 50% probability level).

Diazabicyclo[3.2.1]octane **20a**, resulting from the 1,3-dipolar cycloaddition, was fully characterized by NMR on the basis of a comparison with the data^6^ reported for comparable cycloadduct **9** containing a methyl instead of the 4-methoxybenzyl group. The most characteristic NMR data of **20a** are the chemical shift of protons *d* and *e* and the coupling constants among protons *a*, *b* and *c* (Figure 1A). Protons *d*, corresponding to the exocyclic methylene, appeared as two doublets at 4.20 and 4.34 ppm, and benzylic protons *e* as two doublets at 3.29 and 3.75 ppm. Coupling constants ^3^*J*_Ha,Hb-exo_ and ^3^*J*_Ha,Hb-endo_ were 8.0 and 0.0 Hz, respectively, and ^3^*J*_Hc,Hb-exo_ and ^3^*J*_Hc,Hb-endo_ were 9.0 and 6.4 Hz, respectively. In addition, the ^1^H–^1^H NOESY spectrum of **20a** showed cross-peaks between benzylic protons *e* and methyl *f* as well as between NH and one of the protons *d* of the exocyclic methylene (Figure 1C). In the ^1^H–^13^C HMBC spectrum, cross-peaks between proton *a* and the quaternary bridgehead carbon as well as between methyl *f* and proton *c* were observed. Accordingly, **20a** contained the carboxylate group at carbon *c* with an *exo* configuration. Its structure was unambiguously confirmed by X-ray diffraction [CCDC 2297226 (Figure 1D)].

Minor product **21a** obtained in this reaction had the same molecular composition as **20a** but was more polar, as shown by thin layer chromatography (TLC) and high-performance liquid chromatography (HPLC). The structure of **21a** was elucidated by NMR analysis, which mainly differed from the data for **20a** in the chemical shift of protons *d* and *e* and the multiplicity pattern of protons *a*, *b* and *c* (Figure 1B). Protons *d* of **21a** appeared as two doublets (3.66 and 3.69 ppm) at chemical shifts lower than those for **20a** (4.20 and 4.34 ppm). Protons *e*, which were expected to be a methylene quartet, appeared as two doublets in **20a** and were observed as an apparent singlet at 4.25 ppm in the spectrum of **21a**. With regard to the vicinal coupling constants of protons *a*, *b* and *c* in **20a** and **21a**, those between protons *a* and *b* differed considerably whereas those between protons *b* and *c* were similar. In the case of compound **20a**, the coupling constant ^3^*J*_Ha,Hb-endo_ of 0.0 Hz pointed out a perpendicular orientation between H_a_ and H_b-endo_ according to the Karplus equation.^14^ In contrast, in compound **21a** the value of both coupling constants ^3^*J*_Ha,Hb-exo_ and ^3^*J*_Ha,Hb-endo_ was 2.0 Hz, which would correspond to a dihedral angle of ∼60° between H_a_ and both H_b-endo_ and H_b-exo_. This result evidenced different bicyclic structures for **20a** and **21a**. Two-dimensional ^1^H–^1^H NOESY and ^1^H–^13^C HMBC experiments were crucial for the structural identification of compound **21a** (Figure 1C). The ^1^H–^1^H NOESY cross-peaks between protons *e* and methyl *f* as well as between NH and one of the protons *d* were not observed for **21a**. In this case, benzylic protons *e* correlated with both exocyclic protons *d* and NH correlated with methyl *f*. Unlike **20a**, the ^1^H–^13^C HMBC spectrum of **21a** showed a correlation between benzylic protons *e* and the quaternary carbon of the exocyclic methylene and no correlation was observed between proton *a* and the quaternary bridgehead carbon. Taking all of this data together, the structure of **21a** was determined to be a 2,5-diazabicyclo[2.2.2]octane. Later, this structure was unambiguously confirmed by single-crystal X-ray diffraction analysis [CCDC 2297223 (Figure 1D)].

Next, the 1,3-dipolar reaction of 3-oxidopyrazinium **17** with *tert*-butyl acrylate was carried out (Table 1, entry 2). It required a longer reaction time (1.5 h) compared to that with methyl acrylate (Table 1, entry 1), and diazabicyclo[3.2.1]octane **20b** was obtained in a yield (63%) that was slightly lower than that of **20a**. This result could be attributed to the steric hindrance posed by the *tert*-butyl group. In addition, diazabicyclo[2.2.2]octane **21b** was isolated in 7% yield. Both **20b** and **21b** were fully characterized by NMR comparisons to the data corresponding to their methyl ester analogues, **20a** and **21a**, respectively.

**Figure 2:**
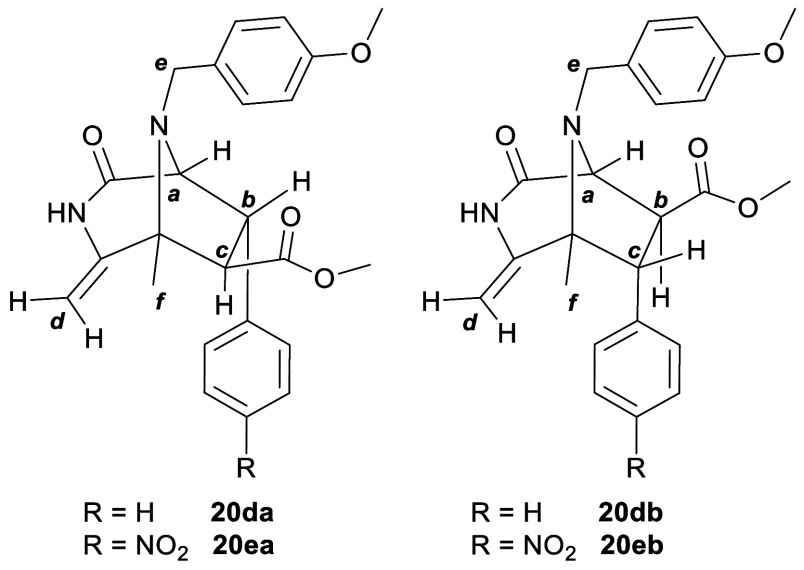
Structures of diazabicyclo[3.2.1]octanes **20da/db** and **20ea/eb**.

The influence of the substituents present in the dipolarophile on the cycloaddition reaction with 3-oxidopyrazinium **17** was then investigated. For this purpose, a methyl acrylate with a methyl, a phenyl, or a substituted phenyl at C-3 of the acrylate unit was utilized, i.e., MeCH=CHCO_2_Me, PhCH=CHCO_2_Me, 4-NO_2_-C_6_H_4_CH=CHCO_2_Me, and 4-NH_2_-C_6_H_4_CH=CHCO_2_Me (Table 1, entries 3–12). These dipolarophiles were subjected to reaction with 3-oxidopyrazinium **17**. In general, in the presence of these substituents, the corresponding diazabicyclo[3.2.1]octanes **20** were formed in low yields and the formation of diazabicyclo[2.2.2]octanes **21** was not detected. The reaction with methyl crotonate afforded **20c** in 51% yield after 6 h at room temperature (Table 1, entry 3). Compound **20c** was characterized by NMR, showing a splitting pattern similar to that of **20a**, and HMBC and NOESY experiments allowed establishment of the regio- and stereochemistry of the product. Thus, in the HMBC spectra, cross-peaks between proton *b* and carbons *a* and *c* were detected, while no NOEs were observed between the methyl group at carbon *b* and the aromatic ring. No reaction with methyl cinnamate took place either at room temperature for 72 h or at 50 °C for 24 h (Table 1, entries 4 and 5). At 80 °C for 4 h, an inseparable mixture of regioisomers **20da** and **20db** (3:4) was obtained in 13% yield (Table 1, entry 6; Figure 2). Longer reaction times led to decomposition products. Using methyl 4-nitrocinnamate, under the conditions developed for methyl cinnamate, the corresponding diazabicyclo[3.2.1]octane **20e** was formed in 4% and 23% yields at 50 and 80 °C, respectively (Table 1, entries 8 and 9, respectively). Bicyclic compound **20e** was obtained as a mixture of regioisomers **20ea** and **20eb** in a 3:2 ratio (Figure 2). Compounds **20d** and **20e** were characterized by NMR, which showed the characteristic signals and correlations of the diazabicyclo[3.2.1]octanes previously discussed. The structure of the corresponding regioisomers, **20da**/**20db** and **20ea**/**20eb**, was elucidated on the basis of the multiplicity of bridgehead proton *a* and the NOESY correlations observed for the phenyl protons of the dipolarophile. In the case of **20da** and **20ea**, proton *a* appeared as a doublet due to the coupling with vicinal proton *b*, while in **20db** and **20eb**, proton *a* appeared as a singlet with a dihedral angle close to 90° with proton *b*. In the NOESY spectra, the phenyl protons of the dipolarophile correlated with protons *a*, *b* and *c* in the case of **20da** and **20ea** and with protons *b* and *c* and methyl *f* for **20db** and **20eb**. The cycloaddition of 3-oxidopyrazinium **17** with methyl 4-aminocinnamate did not lead to a cycloadduct under any of the conditions assayed (Table 1, entries 10–12).

The lower yields of the cycloadditions of 3-oxidopyrazinium **17** with an acrylate containing a substituent at C-3 compared to that with methyl acrylate suggested the importance of a positive charge density at C-3 of the dipolarophile (Table 1, entries 1–12). The presence of a methyl group at this position increases its electron density by hyperconjugation leading to a decrease in the reactivity of the dipolarophile and, therefore, to a decrease in the yield. This effect was more pronounced when using cinnamate derivatives as dipolarophiles. The aromatic ring further decreases the electrophilic character of C-3, and the cycloadditions required heating at 80 °C. In accordance with this reasoning, the presence of a nitro group in the phenyl favored the reaction, whereas the cycloaddition did not proceed upon incorporation of an amino group even at 80 °C.

Finally, 3-oxidopyrazinium **17** was treated with an acrylate bearing a phenyl group at C-2, in particular with methyl 2-phenyl acrylate, CH_2_=CPhCO_2_Me (Table 1, entry 13). To our surprise, a diazabicyclo[3.2.1]octane was not detected and only diazabicyclo[2.2.2]octane **21f** was obtained in 40% yield after 5 h at room temperature. The NMR spectra of **21f** exhibited a pattern analogous to that observed for **21a**. Additionally, the NOESY spectra showed a correlation between methyl *f* and the phenyl protons of the dipolarophile unit, confirming that this aromatic ring is bound to carbon *c* in an *endo* configuration (Scheme 5).

### Cycloaddition Reactions of 3-Oxidopyrazinium **17** with Acrylic Acids

Having studied the reaction of 3-oxidopyrazinium **17** with various acrylic esters as dipolarophiles, we proposed to explore its reactivity with acrylic acids to yield products that would allow further functionalization. This is the first time that acrylic acids have been directly employed as dipolarophiles in these cycloadditions.

**Scheme 6:**
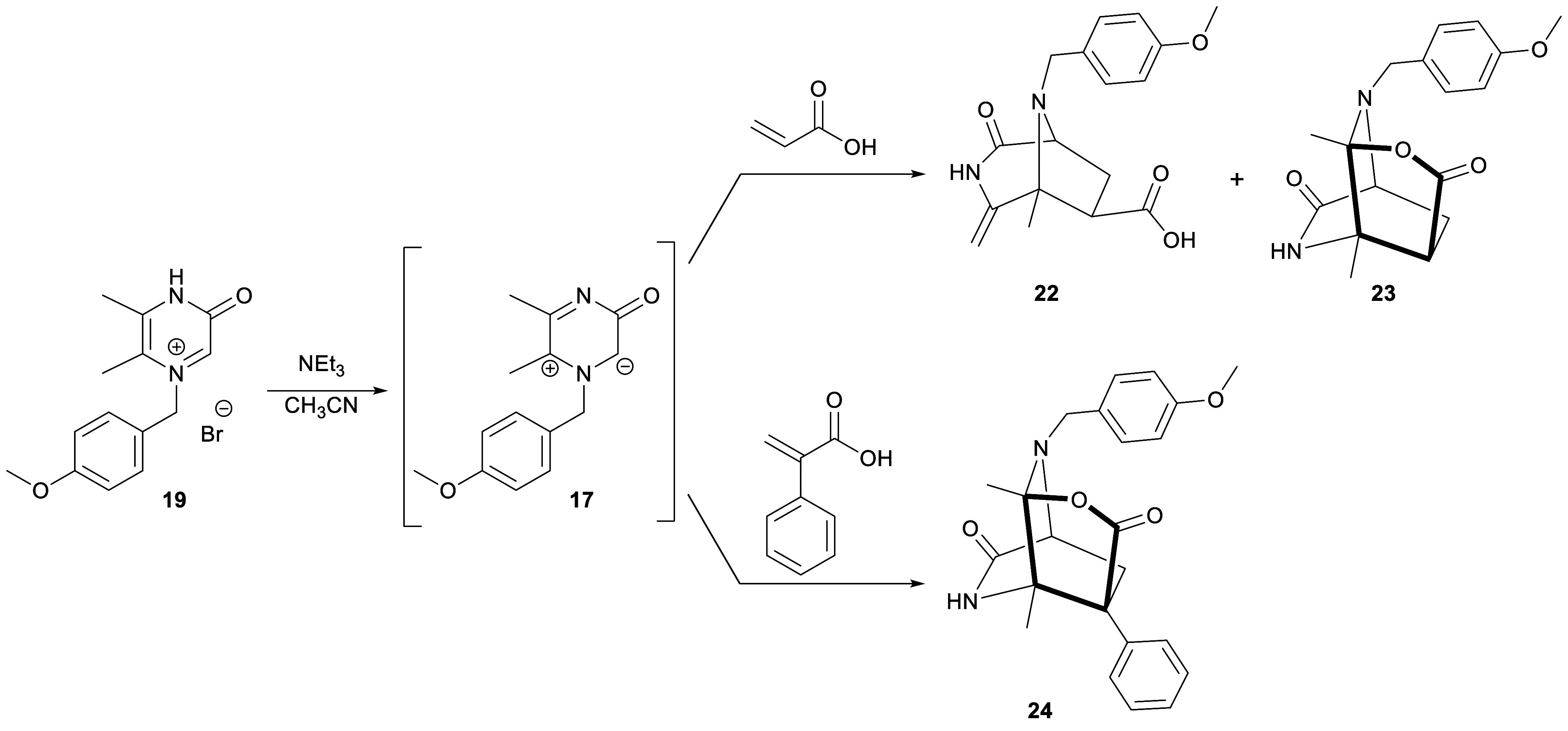
Reaction of 3-Oxidopyrazinium **17** with Acrylic and 2-Phenylacrylic Acid

After reaction of 3-oxidopyrazinium **17** with acrylic acid for 45 min at room temperature, a 1:1 mixture of **22** and **23** was obtained (Scheme 6), which during purification evolved into a 1:3 mixture. The structure of **22** corresponds to the expected diazabicyclo[3.2.1]octane from a 1,3-dipolar cycloaddition, whereas **23** is a tricyclic fused lactone-lactam system derived from a [2.2.2] core. Unexpectedly, compound **22** gradually converted into **23** over time. After 10 days, conversion to **23** was total, and this compound was obtained in 71% yield.

To gain further insight into this cycloaddition, we performed the reaction with a bulkier acid, in particular with 2-phenylacrylic acid. In this case, after reaction for 1.5 h at room temperature, only compound **24** was isolated in 50% yield.

**Figure 3:**
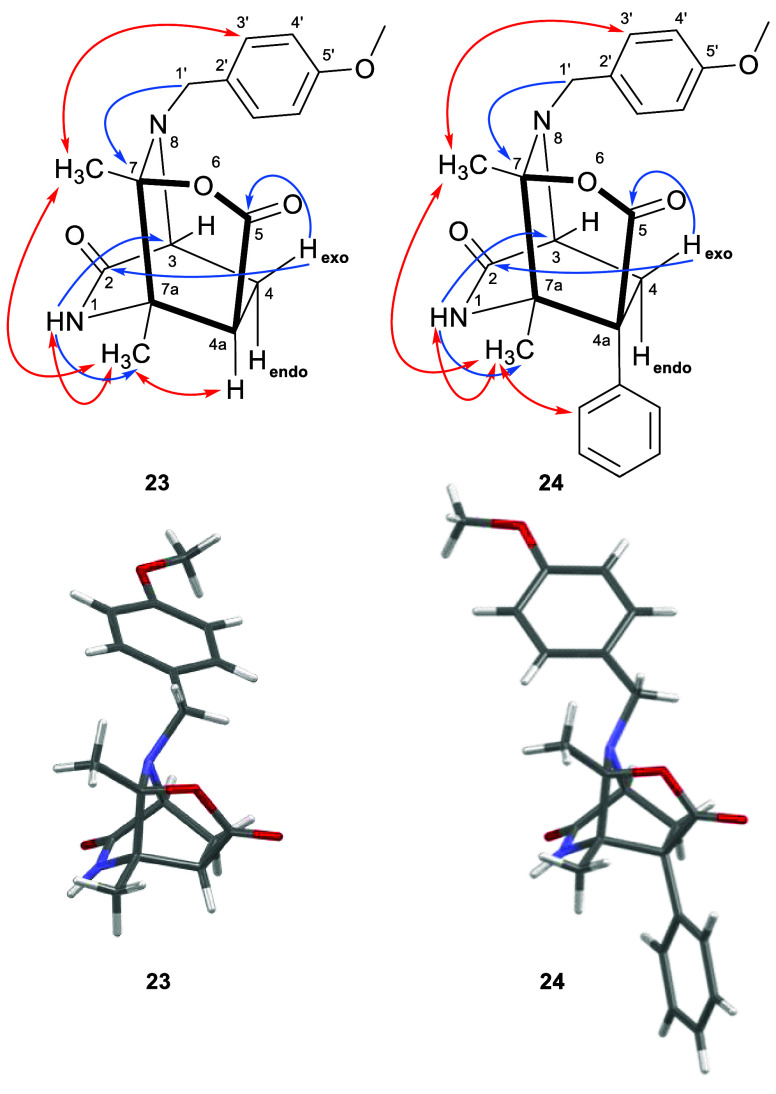
Selected key ^1^H–^13^C HMBC (blue arrows), ^1^H–^1^H NOESY correlations (red arrows), and capped-stick representations of the X-ray crystal structures of **23** and **24** (see the Supporting Information for the ORTEP representation showing thermal ellipsoids at the 50% probability level).

Characterization of compounds **23** and **24** was achieved by two-dimensional ^1^H–^13^C HMBC and ^1^H–^1^H NOESY experiments (Figure 3). Their HMBC spectra showed cross-peaks between NH and both C-3 and the methyl group bound to C-7a. In addition, the benzylic protons correlated with C-7 and H-4exo correlated with the two carbonyl carbons. In contrast, the methyl bound to C-7 did not show any HMBC correlations. The most relevant NOESY correlations were those observed for the methyl bound to C-7a that correlated with NH, with the methyl bound to C-7, and with the protons of the phenyl (**24**) or with H-4a (**23**). Moreover, the methyl bound to C-7 showed cross-peaks with protons H-3′ of the 4-methoxybenzyl unit. The structures of compounds **23** and **24** were unambiguously established by single-crystal X-ray diffraction [CCDC 2297225 and 2297224 (Figure 3)].

### Mechanism of the Formation of the Diazabicyclo[2.2.2]octanes from the Reaction of 3-Oxidopyrazinium **17** with Acrylates and Acrylic Acids

The formation of diazabicyclo[2.2.2]octanes from the reaction of 3-oxidopyrazinium **17** with acrylate derivatives together with the observed progressive conversion of diazabicyclo[3.2.1]octane **22** into tricyclic fused lactone-lactam system **23** prompted us to explore whether the diazabicyclo[2.2.2]octanes are formed through an aza-Diels–Alder type reaction or derived from a diazabicyclo[3.2.1]octane via a rearrangement.

**Scheme 7:**
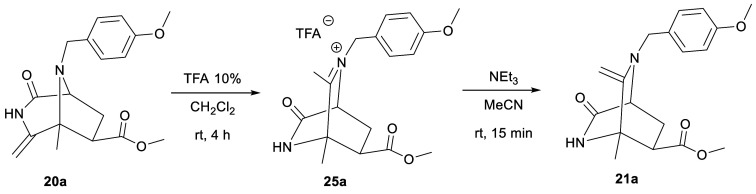
Formation of Diazabicyclo[2.2.2]octane **21a** from Diazabicyclo[3.2.1]octane **20a**

To shed light on this question, we treated diazabicyclo[3.2.1]octane **20a** with 10% TFA in CH_2_Cl_2_ (Scheme 7). After 4 h, we observed full conversion of **20a** into a very polar compound **25a**, and its structure was elucidated by ESI-MS and NMR as the iminium salt derivative of diazabicyclo[2.2.2]octane **21a**. The NMR spectra did not show the signals corresponding to the exocyclic methylene protons. In contrast, in the ^1^H NMR spectrum appeared a significantly deshielded methyl group at 2.86 ppm corresponding to the CH_3_ attached to the iminium group and the ^13^C{^1^H} NMR spectrum showed a low field signal at 190.1 ppm that can be assigned to the carbon of this iminium. The presence of this cationic group also caused an important upfield shift of the bridgehead proton (4.75 ppm) and of the benzyl protons (5.07 and 5.41 ppm). In addition, the ^1^H–^13^C HMBC spectrum did not show cross-peaks between the bridgehead atoms. All of these data pointed toward the formation of diazabicyclo[2.2.2]octane **25** resulting from the rearrangement of diazabicyclo[3.2.1]octane **20a**. Treatment of iminium salt **25** with triethylamine yielded diazabicyclo[2.2.2]octane **21a**, providing further evidence of the structure of **25**.

**Scheme 8:**
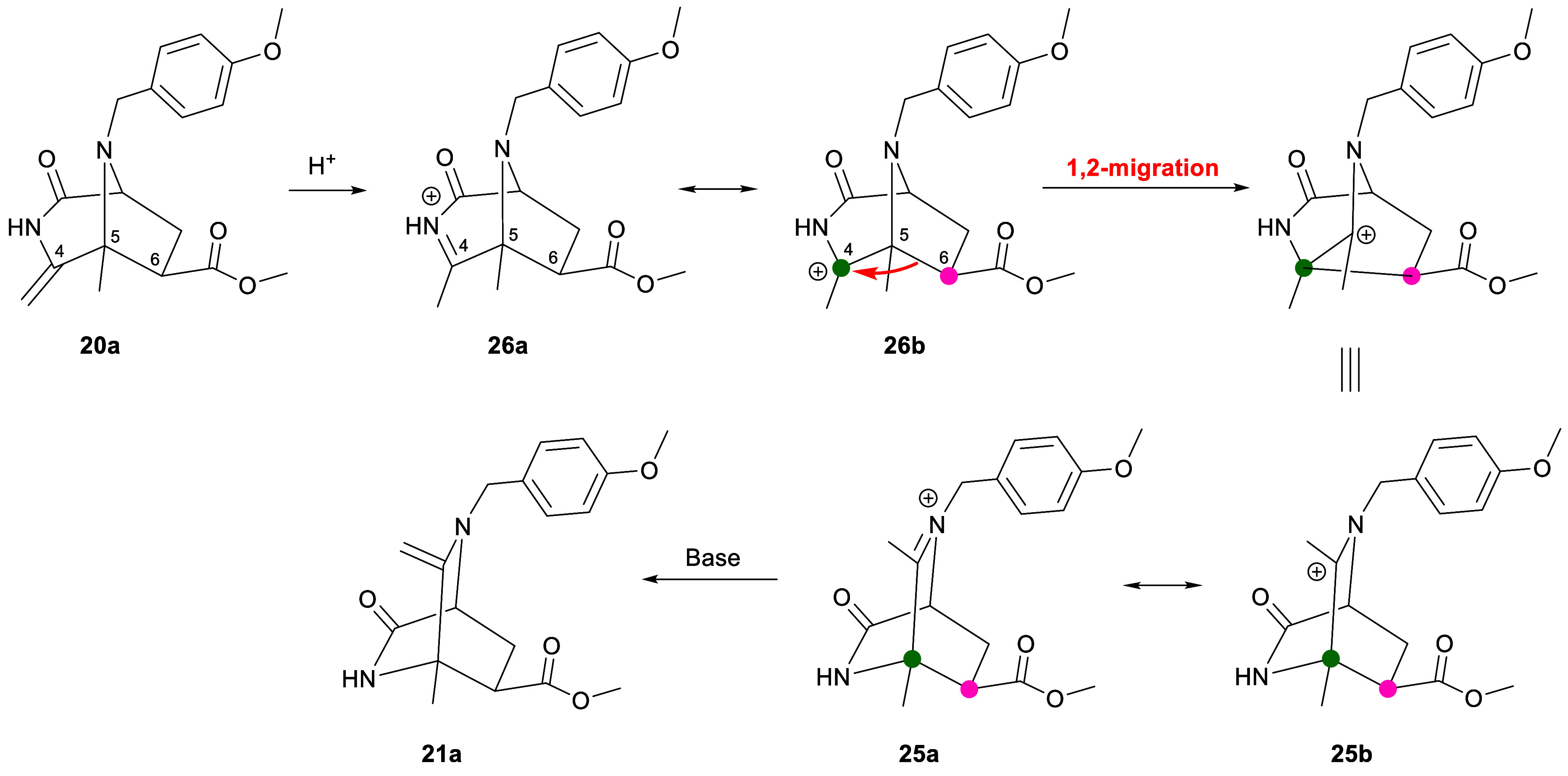
Acid-Catalyzed Mechanism for the Conversion of **20a** into **21a**

The result presented above made clear that the diazabicyclo[2.2.2]octanes obtained in the reactions of 3-oxidopyrazinium **17** with acrylate derivatives are formed from the corresponding diazabicyclo[3.2.1]octanes. A plausible mechanism for this transformation is depicted in Scheme 8 for **20a**. The first step would involve the C protonation of the enamide producing cation **26**. Next, a Wagner–Meerwein rearrangement that implies the 1,2-migration of the C-5–C-6 bond to the C-4 carbocation followed by deprotonation would provide bicyclic compound **21a**. This result confirms the theoretical assessment previously obtained by the DFT method.^11^

**Scheme 9:**
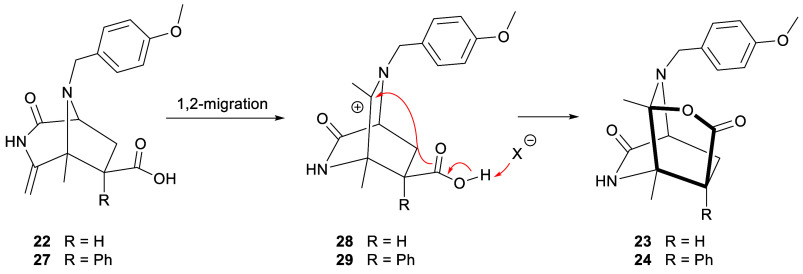
Mechanism for the Conversion of Diazabicyclo[3.2.1]octanes **22** and **27** into Tricyclic Fused Lactone-Lactam Systems **23** and **24**

The formation of tricyclic fused lactone-lactam systems **23** and **24** in the cycloadditions of **17** with an acrylic acid can be rationalized via a mechanism analogous to that described in Scheme 8 followed by a lactonization step. Thus, the initial cycloaddition would provide diazabicyclo[3.2.1]octanes **22** and **27**, which would be readily transformed into iminium salts **28** and **29**, respectively, via a 1,2-migration (Scheme 9). Finally, the nucleophilic attack of the carboxylate on the carbocation would yield tricyclic compounds **23** and **24**.

## Conclusions

3-Oxidopyraziniums generated by simple deprotonation of pyrazin-2-one *N*-alkyl salts react with acrylates forming 3,8-diazabicyclo[3.2.1]octanes. In some cases, isomeric 2,5-diazabicyclo[2.2.2]octanes are also formed. Evidence is presented that suggests that the [2.2.2] products are formed from the [3.2.1] products through a mechanism that involves a Wagner–Meerwein rearrangement. Further evidence of this [3.2.1] to [2.2.2] product conversion is provided from the fused lactone-lactam system obtained from the reaction of 3-oxidopyraziniums with acrylic acids. In this case, after the Wagner–Meerwein rearrangement, a subsequent lactonization occurs. To the best of our knowledge, these are the first examples of the cycloaddition of 3-oxidopyraziniums with acrylic acids. We note that, the tricyclic products from these reactions are highly functionalized and can be obtained from commercially available starting materials in three steps.

## Experimental Section

### General Methods

Anhydrous CH_3_CN and CH_2_Cl_2_ were obtained from an MBraun SPS-800 (Garching, Germany) solvent purification system. TLC analyses were performed on precoated TLC plates with silica gel 60 F254 (Merck), and detection was done with ultraviolet light (254 nm). Flash chromatography purifications were performed on silica gel 60 (0.040–0.063 mm, Merck). Analysis by HPLC was carried out with a model 1260 Infinity II instrument (Agilent Technologies) consisting of a 1260 vial sampler, a Pump VL quaternary pump, and a diode array HS detector and controlled by OpenLab CDS ChemStation software. The analysis was performed in reverse phase using a Kromasil 100 C_18_ column (3 μm, 4.6 mm × 40 mm) and a mobile phase consisting of H_2_O with 0.1% TFA (solvent A) and CH_3_CN with 0.1% TFA (solvent B) and a flow rate of 1 mL/min. For the elution, a linear gradient from 2% to 100% was applied over 12 min. NMR experiments were performed in the Serveis Tècnics de Recerca de la Universitat de Girona (STR-UdG) with an Ultrashield Avance III 400 (9.4 T) spectrometer from Bruker (^1^H, 400 MHz; ^13^C, 100 MHz), equipped with an RT BBI probe and a temperature control unit (BCU Xtreme) or with an Ultrashield ASCEND Nanobay 400 instrument (9.4 T) from Bruker (^1^H, 400 MHz; ^13^C, 100 MHz). Structural assignments were made with additional information from gCOSY, gHSQC, and gHMBC experiments. NMR spectra were processed and analyzed using TopSpin 3.6.2. Chemical shifts were reported as δ (parts per million) directly calibrated with the solvent signal. IR spectra were recorded with a Cary 630 FT-IR spectrophotometer (Agilent Technologies) equipped with a Golden Gate Single Reflection, ATR MK-II system. For the acquisition, the instrument was controlled with MicroLabPC software, and spectra were analyzed with ResolutionsPro version 5.2.0. ESI-MS analyses were performed with an Esquire 6000 ESI Bruker ion trap LC/MS instrument equipped with an electrospray ion source (STR-UdG) operating in both positive and negative ion modes. Samples (5 μL) were introduced into the mass spectrometer ion source directly through a 1200 series Agilent HPLC autosampler. The mobile phase, CH_3_CN/H_2_O (80:20), was delivered by an Agilent 1200 series HPLC pump at a flow rate of 100 μL/min. Nitrogen was employed as the drying and nebulizing gas. HRMS spectra were recorded under ESI conditions with a Bruker MicroTOF-Q II instrument using a hybrid quadrupole time-of-flight mass spectrometer (STR-UdG). Samples were introduced into the mass spectrometer ion source by direct infusion through a syringe pump and externally calibrated using sodium formate. Single-crystal X-ray diffraction (XRD) data were acquired on a Bruker D8 Quest Eco diffractometer (STR-UdG) equipped with graphite-monochromated molybdenum Kα radiation (λ = 0.71073 Å) and a Photon II area detector. The melting point of the compounds was determined with a Melting Point SMP10 (Stuart) instrument, and their values are expressed in degrees Celsius.

### 5,6-Dimethyl-2(1*H*)-pyrazinone (**18**)

In a 250 mL round-bottom flask, glycinamide hydrochloride (2.5 g, 22.6 mmol, 1 equiv) was dissolved in MeOH (40 mL) and the solution cooled to −30 to −40 °C. Aqueous NaOH (12.5 M, 56.5 mmol, 2.5 equiv) was added, and the mixture stirred for 10 min. Then, a solution of butane-2,3-dione (2.4 mL, 27.2 mmol, 1.2 equiv) in MeOH (10 mL), previously cooled, was added dropwise, and the mixture was maintained at −30 °C for 30 min and then at room temperature for 3 h. The formation of the 2(1*H*)-pyrazinone was monitored by TLC, and once it was complete, concentrated HCl was added to the crude reaction mixture followed by neutralization with a saturated aqueous solution of NaHCO_3_. MeOH was evaporated under reduced pressure, and the remaining aqueous solution extracted with CHCl_3_ (3 × 50 mL). The organic extract was dried over anhydrous MgSO_4_, filtered, and evaporated to give 5,6-dimethyl-2(1*H*)-pyrazinone. Recrystallization of the crude product from acetone afforded 5,6-dimethyl-2(1*H*)-pyrazinone as a pale pink solid (1.21 g, 43% yield):^13^ MW (C_6_H_8_N_2_O) 124.1 g/mol; TLC (8:6:0.5 CHCl_3_/MeOH/AcOH) *R*_*f*_ = 0.63; mp 194–196 °C; FT-IR (ATR) *v* (cm^–1^) 3271 (N–H), 2855 (C–H), 1683 (C=O), 1607 (N–C=O); ^1^H NMR (400 MHz, CDCl_3_) δ 13.90 (br, H-1), 8.01 (s, 1H, H-3), 2.32 (s, 3H, 5-CH_3_), 2.29 (s, 3H, 6-CH_3_); ^13^C{^1^H} NMR (100 MHz, CDCl_3_) δ 159.2 (C-2), 144.4 (CH, C-3), 134.5 (C-6), 132.4 (C-5), 19.3 (6-CH_3_), 17.1 (5-CH_3_); ESI-MS (*m*/*z*) 125.1 [M + H]^+^, 147.0 [M + Na]^+^; ESI-HRMS (*m*/*z*) calcd for C_6_H_8_N_2_ONa [M + Na]^+^ 147.0529, found 147.0537; ESI-HRMS (*m*/*z*) calcd for (C_6_H_8_N_2_O)_2_Na [2M + Na]^+^ 271.1165, found 271.1169. Spectral data in accordance with literature values.^14^

### 1-(4-Methoxybenzyl)-5,6-dimethyl-3-oxo-3,4-dihydropyrazin-1-ium Bromide (**19**)

In a round-bottom flask, 5,6-dimethyl-1*H*-pyrazin-2-one (1.18 g, 9.52 mmol, 1 equiv) was dissolved in anhydrous CH_3_CN under N_2_ and, subsequently, *p*-methoxybenzyl bromide (3.83 g, 19.04 mmol, 2 equiv) was added. The mixture was heated at reflux using an oil bath for 6 h and then stirred at room temperature for 18 h, until the reaction had reached completion as evidenced by TLC. Then, the solvent was evaporated under reduced pressure and the residue was purified by reversed-phase chromatography. Elution with H_2_O/CH_3_CN (85:15) afforded 1-(4-methoxybenzyl)-5,6-dimethyl-3-oxo-3,4-dihydropyrazin-1-ium bromide (2.33 g, 75% yield) as a pale brown solid: MW (C_14_H_17_N_2_O_2_Br) 325.2 g/mol; TLC (9:1 CHCl_3_/MeOH) *R*_*f*_ = 0.14; mp 108–112 °C; FT-IR (ATR) *v* (cm^–1^) 3338 (N–H), 2917 (C–H), 1673 (C=O), 1606 (Ar), 1510 (N–C=O), 1247 and 1176 (C–O); ^1^H NMR (400 MHz, CDCl_3_) δ 8.23 (s, 1H, H-2), 7.30 (d, *J* = 8.4 Hz, 2H, H-3′), 6.89 (d, *J* = 8.4 Hz, 2H, H-4’), 5.72 (s, 2H, H-1′), 3.75 (s, 3H, 5′-OCH_3_), 2.59 (s, 3H, 6-CH_3_), 2.58 (s, 3H, 5-CH_3_); ^13^C{^1^H} NMR (100 MHz, CDCl_3_) δ 160.8 (C-5′), 159.4 (C-3), 155.6 (C-5), 133.6 (C-6), 130.8 (2CH, C-3′), 128.9 (CH, C-2), 121.7 (C-2′), 115.2 (2CH, C-4′), 62.9 (CH_2_, C-1′), 55.4 (5′-OCH_3_), 21.6 (5-CH_3_), 15.2 (6-CH_3_); ESI-MS (*m*/*z*) 121.0 [CH_3_O – C_6_H_4_ – CH_2_]^+^, 245.1 [M]^+^, 267.1 [M – H + Na]^+^; ESI-HRMS (*m*/*z*) calcd for C_14_H_16_N_2_O_2_Na [M – H + Na]^+^ 267.1104, found 267.1116; ESI-HRMS (*m*/*z*) calcd for (C_14_H_16_N_2_O_2_)_2_Na [2M – 2H + Na]^+^ 511.2316, found 511.2327.

### General Procedure for Cycloadditions

To a suspension of 1-(4-methoxybenzyl)-5,6-dimethyl-3-oxo-3,4-dihydropyrazin-1-ium bromide (1 equiv) in anhydrous CH_3_CN (2 mL) was added dropwise triethylamine (1.5 equiv), and the mixture was stirred at room temperature under nitrogen. After 10 min, the formation of an orange transparent solution evidenced the formation of the 3-oxidopyrazinium. Then, the corresponding dipolarophile (1.5 equiv) was added dropwise to the ylide and the resulting mixture was stirred under nitrogen. The progress of the reaction was monitored by TLC. Once the reaction had reached completion, the solvent was removed under reduced pressure and the resulting residue was purified by flash chromatography using CH_2_Cl_2_/MeOH mixtures of increasing polarity.

### Methyl 8-(4-Methoxybenzyl)-5-methyl-4-methylene-2-oxo-3,8-diazabicyclo[3.2.1]octane-6-carboxylate (**20a**)

This compound was prepared following the general procedure described above starting from 1-(4-methoxybenzyl)-5,6-dimethyl-3-oxo-3,4-dihydropyrazin-1-ium bromide (88 mg, 0.27 mmol, 1 equiv) and methyl acrylate (37 μL, 0.41 mmol, 1.5 equiv) as the dipolarophile. The reaction was performed at room temperature for 45 min. Final purification by flash chromatography eluting with CH_2_Cl_2_/MeOH (99:1) afforded **20a** (66 mg, 73% yield) as a pale yellow solid. Suitable crystals for X-ray diffraction were obtained by slow diffusion of hexane into a CH_2_Cl_2_ solution of the compound: MW (C_18_H_22_N_2_O_4_) 330.4 g/mol; TLC (9:1 CHCl_3_/MeOH) *R*_*f*_ = 0.58; HPLC (λ = 220 nm) *t*_R_ = 6.33 min (94% purity); mp 143–145 °C; FT-IR (ATR) *v* (cm^–1^) 2925 (C–H), 1773 and 1663 (C=O), 1507 (Ar), 1180 (C–O); ^1^H NMR (400 MHz, CDCl_3_) δ 8.53 (s, 1H, H-3), 7.22 (d, *J* = 8.8 Hz, 2H, H-3′), 6.83 (d, *J* = 8.8 Hz, 2H, H-4′), 4.34 (d, *J* = 1.6 Hz, 1H, H-9), 4.20 (d, *J* = 1.6 Hz, 1H, H-9), 3.78 (s, 3H, CO_2_CH_3_), 3.75 (d, *J* = 13.2 Hz, 1H, H-1′), 3.73 (s, 3H, 5′-OCH_3_), 3.50 (d, *J* = 8.0 Hz, 1H, H-1), 3.29 (d, *J* = 13.2 Hz, 1H, H-1′), 3.07 (dd, *J* = 9.0 Hz, *J*′ = 6.4 Hz, 1H, H-6), 2.56 (ddd, *J* = 12.8 Hz, *J*′ = 8.0 Hz, *J*″ = 6.4 Hz, 1H, H-7_exo_), 2.10 (dd, *J* = 12.8 Hz, *J*′ = 9.0 Hz, 1H, H-7_endo_), 1.36 (s, 3H, 5-CH_3_); ^13^C{^1^H} NMR (100 MHz, CDCl_3_) δ 173.3 (*C*O_2_CH_3_), 172.2 (C-2), 158.7 (C-5′), 146.2 (C-4), 130.2 (C-2′), 129.6 (2CH, C-3′), 113.7 (2CH, C-4′), 90.3 (CH_2_, C-9), 63.5 (CH, C-1), 62.7 (C-5), 55.2 (CO_2_*C*H_3_), 52.5 (CH, C-6), 51.9 (5′-OCH_3_), 49.0 (CH_2_, C-1′), 31.8 (CH_2_, C-7), 17.8 (5-CH_3_); ESI-MS (*m*/*z*) 331.2 [M + H]^+^; ESI-HRMS (*m*/*z*) calcd for C_18_H_22_N_2_O_4_Na [M + Na]^+^ 353.1472, found 353.1478.

### *tert*-Butyl 8-(4-Methoxybenzyl)-5-methyl-4-methylene-2-oxo-3,8-diazabicyclo[3.2.1]octane-6-carboxylate (**20b**) and *tert*-Butyl 5-(4-Methoxybenzyl)-1-methyl-6-methylene-3-oxo-2,5-diazabicyclo[2.2.2]octane-7-carboxylate (**21b**)

These compounds were prepared following the general procedure described above starting from 1-(4-methoxybenzyl)-5,6-dimethyl-3-oxo-3,4-dihydropyrazin-1-ium bromide (104 mg, 0.32 mmol, 1 equiv) and *tert*-butyl acrylate (71 μL, 0.48 mmol, 1.5 equiv) as the dipolarophile. The reaction was performed at room temperature for 1.5 h. Final purification by flash chromatography eluting with CH_2_Cl_2_/MeOH (99:1) afforded **20b** (76 mg, 63% yield) as a white solid. Also, **21b** (8 mg, 7% yield) was isolated as a byproduct as a white solid.

#### *tert*-Butyl 8-(4-Methoxybenzyl)-5-methyl-4-methylene-2-oxo-3,8-diazabicyclo[3.2.1]octane-6-carboxylate (**20b**)

MW (C_21_H_28_N_2_O_4_) 372.5 g/mol; TLC (9:1 CHCl_3_/MeOH) *R*_*f*_ = 0.67; HPLC (λ = 220 nm) *t*_R_ = 8.04 min (97% purity); mp 142–145 °C; FT-IR (ATR) *v* (cm^–1^) 2974 (C–H), 1722 and 1669 (C=O), 1509 (Ar), 1147 (C–O); ^1^H NMR (400 MHz, CDCl_3_) δ 8.72 (s, 1H, H-3), 7.24 (d, *J* = 8.4 Hz, 2H, H-3′), 6.83 (d, *J* = 8.4 Hz, 2H, H-4′), 4.35 (d, *J* = 1.4 Hz, 1H, H-9), 4.20 (d, *J* = 1.4 Hz, 1H, H-9), 3.78 (s, 3H, 5′-OCH_3_), 3.76 (d, *J* = 13.2 Hz, 1H, H-1′), 3.49 (d, *J* = 7.6 Hz, 1H, H-1), 3.30 (d, *J* = 13.2 Hz, 1H, H-1′), 2.96 (dd, *J* = 9.2 Hz, *J*′ = 6.8 Hz, 1H, H-6), 2.52 (ddd, *J* = 12.8 Hz, *J*′ = 7.6 Hz, *J*″ = 6.8 Hz, 1H, H-7_exo_), 2.08 (dd, *J* = 12.8 Hz, *J*′ = 9.2 Hz, 1H, H-7_endo_), 1.49 [s, 9H, C(CH_3_)_3_], 1.44 (s, 3H, 5-CH_3_); ^13^C{^1^H} NMR (100 MHz, CDCl_3_) δ 172.6 (C-2), 172.0 (CO_2_*t*-Bu), 158.7 (C-5′), 146.5 (C-4), 130.5 (C-2′), 129.5 (2CH, C-3′), 113.7 (2CH, C-4′), 90.3 (CH_2_, C-9), 81.2 [*C*(CH_3_)_3_], 63.5 (CH, C-1), 62.7 (C-5), 55.2 (5′-OCH_3_), 53.2 (CH, C-6), 49.0 (CH_2_, C-1′), 32.0 (CH_2_, C-7), 28.1 [3CH_3_, C(*C*H_3_)_3_], 17.9 (5-CH_3_); ESI-MS (*m*/*z*) 373.2 [M + H]^+^; ESI-HRMS (*m*/*z*) calcd for C_21_H_28_N_2_O_4_Na [M + Na]^+^ 395.1941, found 395.1941; ESI-HRMS (*m*/*z*) calcd for (C_21_H_28_N_2_O_4_)_2_Na [2M + Na]^+^ 767.3990, found 767.3982.

#### *tert*-Butyl 5-(4-Methoxybenzyl)-1-methyl-6-methylene-3-oxo-2,5-diazabicyclo[2.2.2]octane-7-carboxylate (**21b**)

MW (C_21_H_28_N_2_O_4_) 372.5 g/mol; TLC (9:1 CHCl_3_/MeOH) *R*_*f*_ = 0.41; ^1^H NMR (400 MHz, CDCl_3_) δ 7.23 (d, *J* = 8.5 Hz, 2H, H-3′), 6.86 (d, *J* = 8.5 Hz, 2H, H-4′), 6.32 (br, 1H, H-2), 4.23 (s, 2H, H-1′), 3.79 (s, 3H, 5′-OCH_3_), 3.70–3.67 (m, 2H, H-9), 3.59 (m, 1H, H-4), 2.79 (dd, *J* = 11.7 Hz, *J*′ = 4.6 Hz, 1H, H-7), 2.52 (dt, *J* = 11.7 Hz, *J*′ = 4.6 Hz, 1H, H-8_exo_), 2.00 (t, *J* = 11.7 Hz, 1H, H-8_endo_), 1.58 (s, 3H, 1-CH_3_), 1.46 [s, 9H, C(CH_3_)_3_]; ESI-MS (*m*/*z*) 373.2 [M + H]^+^.

### Methyl 8-(4-Methoxybenzyl)-5,7-dimethyl-4-methylene-2-oxo-3,8-diazabicyclo[3.2.1]octane-6-carboxylate (**20c**)

This compound was prepared following the general procedure described above starting from 1-(4-methoxybenzyl)-5,6-dimethyl-3-oxo-3,4-dihydropyrazin-1-ium bromide (107 mg, 0.33 mmol, 1 equiv) and methyl crotonate (54 μL, 0.50 mmol, 1.5 equiv) as the dipolarophile. The reaction was performed at room temperature for 6 h. Final purification by flash chromatography eluting with CH_2_Cl_2_/MeOH (99:1) afforded **20c** (58 mg, 51% yield) as a white solid: MW (C_19_H_24_N_2_O_4_) 344.4 g/mol; TLC (9:1 CHCl_3_/MeOH) *R*_*f*_ = 0.70; HPLC (λ = 220 nm) *t*_R_ = 6.49 min (98% purity); mp 179–182 °C; FT-IR (ATR) *v* (cm^–1^) 2951 (C–H), 1733 and 1680 (C=O), 1509 (Ar), 1165 (C–O); ^1^H NMR (400 MHz, CDCl_3_) δ 8.04 (s, 1H, H-3), 7.22 (d, *J* = 8.6 Hz, 2H, H-3′), 6.83 (d, *J* = 8.6 Hz, 2H, H-4′), 4.31 (d, *J* = 1.6 Hz, 1H, H-9), 4.19 (d, *J* = 1.6 Hz, 1H, H-9), 3.79 (s, 3H, 5′-OCH_3_), 3.76 (s, 3H, CO_2_CH_3_), 3.72 (d, *J* = 13.6 Hz, 1H, H-1′), 3.38 (dd, *J* = 7.2 Hz, *J*′ = 1.2 Hz, 1H, H-1), 3.26 (d, *J* = 13.6 Hz, 1H, H-1′), 2.95 (sext, *J* = 7.2 Hz, 1H, H-7), 2.60 (d, *J* = 7.2 Hz, 1H, H-6), 1.36 (s, 3H, 5-CH_3_), 1.04 (d, *J* = 7.2 Hz, 3H, 7-CH_3_); ^13^C{^1^H} NMR (100 MHz, CDCl_3_) δ 173.2 (*C*O_2_CH_3_), 170.3 (C-2), 158.9 (C-5′), 146.8 (C-4), 130.3 (C-2′), 129.8 (2CH, C-3′), 113.9 (2CH, C-4′), 90.0 (CH_2_, C-9), 67.7 (CH, C-1), 64.0 (C-5), 60.9 (CH, C-6), 55.4 (5′-OCH_3_), 52.1 (CO_2_*C*H_3_), 49.4 (CH_2_, C-1′), 38.7 (CH, C-7), 17.7 (5-CH_3_), 15.0 (7-CH_3_); ESI-MS (*m*/*z*) 345.1 [M + H]^+^; ESI-HRMS (*m*/*z*) calcd for C_19_H_24_N_2_O_4_Na [M + Na]^+^ 367.1628, found 367.1625; ESI-HRMS (*m*/*z*) calcd for (C_19_H_24_N_2_O_4_)_2_Na [2M + Na]^+^ 711.3364, found 711.3327.

### Methyl 8-(4-Methoxybenzyl)-5-methyl-4-methylene-2-oxo-7-phenyl-3,8-diazabicyclo[3.2.1]octane-6-carboxylate (**20da**) and Methyl 8-(4-Methoxybenzyl)-1-methyl-2-methylene-4-oxo-7-phenyl-3,8-diazabicyclo[3.2.1]octane-6-carboxylate (**20db**)

These compounds were prepared following the general procedure described above starting from 1-(4-methoxybenzyl)-5,6-dimethyl-3-oxo-3,4-dihydropyrazin-1-ium bromide (218 mg, 0.67 mmol, 1 equiv) and methyl cinnamate (163 mg, 1.00 mmol, 1.5 equiv) as the dipolarophile. The reaction was performed by heating with an oil bath at 80 °C for 4 h. Final purification by flash chromatography eluting with CH_2_Cl_2_/MeOH (99:1) afforded an inseparable mixture of regioisomers **20da** and **20db** (3:4) (36 mg, 13% yield): MW (C_24_H_26_N_2_O_4_) 406.5 g/mol; TLC (9:1 CHCl_3_/MeOH) *R*_*f*_ = 0.43; ^1^H NMR (400 MHz, CDCl_3_) δ 8.28 (s, 1H, H-3a), 8.06 (s, 1H, H-3b), 7.29–7.20 (m, 14H, H-3′a, H-3′b, H-11a, H-11b, H-12a, H-12b, H-13a, H-13b), 6.87–6.85 (m, 4H, H-4′a, H-4′b), 4.33 (d, *J* = 1.6 Hz, 1H, H-9a), 4.26 (d, *J* = 1.6 Hz, 1H, H-9a), 4.24 (m, 1H, H-7a), 4.16 (d, *J* = 1.6 Hz, 1H, H-9b), 3.89 (d, 1H, *J* = 13.6 Hz, H-1′a), 3.86 (s, 1H, H-5b), 3.80 (d, *J* = 13.2 Hz, 1H, H-1′b), 3.80 (s, 3H, 5′-OCH_3_-a), 3.79 (s, 3H, 5′-OCH_3_-b), 3.73 (m, 1H, H-7b), 3.73 (s, 3H, CO_2_CH_3_-a), 3.68 (d, *J* = 6.4 Hz, 1H, H-1a), 3.65 (d, *J* = 1.6 Hz, 1H, H-9b), 3.62 (s, 3H, CO_2_CH_3_-b), 3.40 (d, *J* = 8.4 Hz, 1H, H-6a), 3.38 (d, *J* = 13.6 Hz, 1H, H-1′a), 3.31 (d, *J* = 13.6 Hz, 1H, H-1′b), 3.21 (d, *J* = 6.4 Hz, 1H, H-6b), 1.46 (s, 3H, 5-CH_3_-a), 1.39 (s, 3H, 1-CH_3_-b); ^13^C{^1^H} NMR (100 MHz, CDCl_3_) δ 172.9 (*C*O_2_CH_3_-b), 172.8 (*C*O_2_CH_3_-a), 170.5 (C-4b), 169.5 (C-2a), 159.0 (2C, C-5′a and C-5′b), 146.7 (C-4a), 141.5 (C-2b), 137.0 (C-10b), 136.2 (C-10a), 130.1, 130.0, 129.9, 129.6, 128.9, 128.7, 128.6, 128.4, 128.0 (C-2′, C-3′, C-11, C-12, C-13a and -b), 114.0 (2CH, C-4′a), 113.9 (2CH, C-4′b), 95.0 (CH_2_, C-9b), 90.3 (CH_2_, C-9a), 68.7 (CH, C-1a), 67.3 (C-1b), 65.4 (CH, C-5b), 64.3 (C-5a), 58.7 (CH, C-7b), 58.0 (CH, C-6a), 55.4 (2CH_3_, 5′-OCH_3_-a and 5′-OCH_3_-b), 52.5 (CO_2_*C*H_3_-b), 52.2 (CO_2_*C*H_3_-a), 51.2 (CH, C-6b), 50.1 (CH, C-7a), 49.2 (2CH_2_, C-1′a and C-1′b), 20.7 (1-CH_3_-b), 17.8 (5-CH_3_-a); ESI-MS (*m*/*z*) 407.2 [M + H]^+^; ESI-HRMS (*m*/*z*) calcd for C_24_H_27_N_2_O_4_ [M + H]^+^ 407.1965, found 407.1978.

### Methyl 8-(4-Methoxybenzyl)-5-methyl-4-methylene-7-(4-nitrophenyl)-2-oxo-3,8-diazabicyclo[3.2.1]octane-6-carboxylate (**20ea**) and Methyl 8-(4-Methoxybenzyl)-1-methyl-2-methylene-7-(4-nitrophenyl)-4-oxo-3,8-diazabicyclo[3.2.1]octane-6-carboxylate (**20eb**)

Compound **20ea** was prepared following the general procedure described above starting from 1-(4-methoxybenzyl)-5,6-dimethyl-3-oxo-3,4-dihydropyrazin-1-ium bromide (182 mg, 0.56 mmol, 1 equiv) and methyl *p*-nitrocinnamate^15^ (173 mg, 0.84 mmol, 1.5 equiv) as the dipolarophile. The reaction was performed by heating with an oil bath at 50 °C for 24 h. Final purification by flash chromatography eluting with CH_2_Cl_2_/MeOH (99.5:0.5) afforded only regioisomer **20ea** (11 mg, 4% yield) as a white solid. When the reaction was performed under reflux at 80 °C, an inseparable mixture of regioisomers **20ea** and **20eb** (3:2) was obtained in 23% yield.

#### Methyl 8-(4-Methoxybenzyl)-5-methyl-4-methylene-7-(4-nitrophenyl)-2-oxo-3,8-diazabicyclo[3.2.1]octane-6-carboxylate (**20ea**)

MW (C_24_H_25_N_3_O_6_) 451.5 g/mol; TLC (9:1 CHCl_3_/MeOH) *R*_*f*_ = 0.53; HPLC (λ = 220 nm) *t*_R_ = 8.41 min (>99% purity); mp 188–192 °C; FT-IR (ATR) *v* (cm^–1^) 2849 (C–H), 1735 and 1511 (C=O), 1684 (Ar), 1346 (NO_2_), 1147 (C–O); ^1^H NMR (400 MHz, CDCl_3_) δ 8.11 (d, *J* = 8.8 Hz, 2H, H-12), 8.04 (s, 1H, H-3), 7.36 (d, *J* = 8.8 Hz, 2H, H-11), 7.23 (d, *J* = 8.6 Hz, 2H, H-3′), 6.86 (d, *J* = 8.6 Hz, 2H, H-4′), 4.32 (t, *J* = 7.2 Hz, 1H, H-7), 4.26 (s, 2H, H-9), 3.81 (d, *J* = 13.2 Hz, 1H, H-1′), 3.81 (s, 3H, 5′-OCH_3_), 3.78 (s, 3H, CO_2_CH_3_), 3.72 (dd, *J* = 7.2 Hz, *J*′ = 1.2 Hz, 1H, H-1), 3.34 (d, *J* = 7.2 Hz, 1H, H-6), 3.26 (d, *J* = 13.2 Hz, 1H, H-1′), 1.47 (s, 3H, 5-CH_3_); ^13^C{^1^H} NMR (100 MHz, CDCl_3_) δ 172.1 (*C*O_2_CH_3_), 168.6 (C-2), 159.0 (C-5′), 147.2 (C-13), 145.9 (C-4), 143.8 (C-10), 129.8 (2CH, C-3′), 129.4 (C-2′), 128.8 (2CH, C-11), 123.7 (2CH, C-12), 113.9 (2CH, C-4′), 90.7 (CH_2_, C-9), 67.9 (CH, C-1), 64.2 (C-5), 57.6 (CH, C-6), 55.3 (5′-OCH_3_), 52.4 (CO_2_*C*H_3_), 49.6 (CH, C-7), 49.1 (CH_2_, C-1′), 17.5 (5-CH_3_); ESI-MS (*m*/*z*) 452.2 [M + H]^+^; ESI-HRMS (*m*/*z*) calcd for C_24_H_26_N_3_O_6_ [M + H]^+^ 452.1816, found 452.1811.

#### Methyl 8-(4-Methoxybenzyl)-1-methyl-2-methylene-7-(4-nitrophenyl)-4-oxo-3,8-diazabicyclo[3.2.1]octane-6-carboxylate (**20eb**)

NMR data were obtained from a 3:2 mixture with **20ea**: ^1^H NMR (400 MHz, CDCl_3_) δ 8.14 (d, *J* = 8.8 Hz, 2H, H-12), 7.36 (d, *J* = 8.8 Hz, 2H, H-11), 7.23 (d, *J* = 8.6 Hz, 2H, H-3′), 6.85 (d, *J* = 8.6 Hz, 2H, H-4’), 4.15 (d, *J* = 1.6 Hz, 1H, H-9), 3.92 (d, *J* = 13.2 Hz, 1H, H-1′), 3.91 (s, 1H, H-5), 3.87 (d, *J* = 6.4 Hz, 1H, H-7), 3.81 (s, 3H, 5′-OCH_3_), 3.64 (d, *J* = 1.6 Hz, 1H, H-9), 3.62 (s, 3H, CO_2_CH_3_), 3.38 (d, *J* = 13.2 Hz, 1H, H-1′), 3.19 (d, *J* = 6.4 Hz, 1H, H-6), 1.42 (s, 3H, 1-CH_3_); ^13^C{^1^H} NMR (100 MHz, CDCl_3_) δ 171.9 (*C*O_2_CH_3_), 169.8 (C-4), 159.0 (C-5′), 147.4 (C-13), 144.5 (C-10), 140.7 (C-2), 129.6 (4CH, C-11, C-3′), 128.6 (C-2′), 123.5 (2CH, C-12), 113.9 (2CH, C-4′), 95.3 (CH_2_, C-9), 67.4 (C-1), 65.0 (CH, C-5), 58.1 (CH, C-7), 55.3 (5′-OCH_3_), 52.6 (CO_2_*C*H_3_), 51.0 (CH, C-6), 49.1 (CH_2_, C-1′), 20.5 (1-CH_3_).

### Methyl 5-(4-Methoxybenzyl)-1-methyl-6-methylene-3-oxo-7-phenyl-2,5-diazabicyclo[2.2.2]octane-7-carboxylate (**21f**)

This compound was prepared following the general procedure described above starting from 1-(4-methoxybenzyl)-5,6-dimethyl-3-oxo-3,4-dihydropyrazin-1-ium bromide (198 mg, 0.61 mmol, 1 equiv) and methyl 2-phenyl acrylate^16^ (149 mg, 0.92 mmol, 1.5 equiv) as the dipolarophile. The reaction was performed at room temperature for 5 h. Final purification by flash chromatography eluting with CH_2_Cl_2_/MeOH (99:1) afforded **21f** (96 mg, 40% yield) as a white solid: MW (C_24_H_26_N_2_O_4_) 406.2 g/mol; TLC (9:1 CHCl_3_/MeOH) *R*_*f*_ = 0.38; mp 88–90 °C; FT-IR (ATR) *v* (cm^–1^) 2946 (C–H), 1686 (C=O), 1509 (Ar), 1240 (C–O); ^1^H NMR (400 MHz, CDCl_3_) δ 7.27–7.19 (m, 3H, H-12, H-13), 7.13–7.10 (m, 4H, H-3′, H-11), 6.79 (d, *J* = 8.4 Hz, 2H, H-4′), 6.16 (s, 1H, H-2), 4.22 (d, *J* = 15.4 Hz, 1H, H-1′), 4.06 (d, *J* = 15.4 Hz, 1H, H-1′), 3.89 (s, 1H, H-9), 3.74 (s, 1H, H-9), 3.72 (s, 3H, 5′-OCH_3_), 3.69 (s, 3H, CO_2_CH_3_), 3.62–3.60 (m, 1H, H-4), 3.19 (dd, *J* = 14.8 Hz, *J*′ = 4.4 Hz, 1H, H-8_exo_), 2.33 (dd, *J* = 14.8 Hz, *J*′ = 1.6 Hz, 1H, H-8_endo_), 1.31 (s, 3H, 1-CH_3_); ^13^C{^1^H} NMR (100 MHz, CDCl_3_) δ 173.1 (*C*O_2_CH_3_), 172.9 (C-3), 159.0 (C-5′), 151.7 (C-6), 139.6 (C-10), 130.0 (C-2′), 128.7 (2CH, C-3′), 128.5 (2CH, C-12), 127.8 (2CH, C-11), 127.7 (CH, C-13), 114.1 (2CH, C-4′), 78.3 (CH_2_, C-9), 62.1 (C-1), 59.8 (CH, C-4), 58.5 (CH, C-7), 55.4 (5′-OCH_3_), 54.8 (CH_2_, C-1′), 52.5 (CH_3_, CO_2_*C*H_3_), 39.7 (CH_2_, C-8), 18.7 (1-CH_3_); ESI-MS (*m*/*z*) 407.1 [M + H]^+^; ESI-HRMS (*m*/*z*) calcd for C_24_H_27_N_2_O_4_ [M + H]^+^ 407.1965, found 407.1964.

### 8-(4-Methoxybenzyl)-5-methyl-4-methylene-2-oxo-3,8-diazabicyclo[3.2.1]octane-6-carboxylic Acid (**22**) and 8-(4-Methoxybenzyl)-7,7a-dimethyl-1,3,4,4a-tetrahydro-3,7-epiminofuro[3,4-*b*]pyridine-2,5-dione (**23**)

Compound **23** was prepared following the general procedure described above starting from 1-(4-methoxybenzyl)-5,6-dimethyl-3-oxo-3,4-dihydropyrazin-1-ium bromide (275 mg, 0.85 mmol, 1 equiv) and acrylic acid (87 μL, 1.28 mmol, 1.5 equiv) as the dipolarophile. The reaction was performed at room temperature for 45 min, and the mixture purified by flash chromatography eluting with CH_2_Cl_2_/MeOH (99:1). After purification, an initial mixture of **22** and **23** (1:3) was obtained, which gradually was converted into **23**, isolated as a white solid (189 mg, 71% yield). Crystals suitable for X-ray diffraction of **23** were obtained by slow evaporation of a saturated CH_2_Cl_2_ solution of the compound.

#### 8-(4-Methoxybenzyl)-5-methyl-4-methylene-2-oxo-3,8-diazabicyclo[3.2.1]octane-6-carboxylic Acid (**22**)

NMR data were obtained from a 1:3 mixture with compound **23**: MW (C_17_H_20_N_2_O_4_) 316.4 g/mol; TLC (9:1 CHCl_3_/MeOH) *R*_*f*_ = 0.47; ^1^H NMR (400 MHz, CDCl_3_) δ 8.58 (br, 1H, H-3), 7.18 (d, *J* = 8.8 Hz, 2H, H-3′), 6.85 (d, *J* = 8.8 Hz, 2H, H-4′), 4.41 (d, *J* = 1.8 Hz, 1H, H-9), 4.27 (d, *J* = 1.8 Hz, 1H, H-9), 3.81 (d, *J* = 13.2 Hz, 1H, H-1′), 3.78 (s, 3H, 5′-OCH_3_), 3.51–3.49 (m, 1H, H-1), 3.31 (d, *J* = 13.2 Hz, 1H, H-1′), 3.03 (dd, *J* = 9.2 Hz, *J*′ = 6.0 Hz, 1H, H-6), 2.48 (ddd, *J* = 13.4 Hz, *J*′ = 9.2 Hz, *J*″ = 6.0 Hz, 1H, H-7_exo_), 2.14 (dd, *J* = 13.4 Hz, *J*′ = 9.2 Hz, 1H, H-7_endo_), 1.53 (s, 3H, 5-CH_3_); ^13^C{^1^H} NMR (100 MHz, CDCl_3_, assigned from HSQC and HMBC) δ 175.0 (COOH), 171.4 (C-2), 158.7 (C-5′), 146.2 (C-4), 130.2 (C-2′), 129.6 (2CH, C-3′), 113.7 (2CH, C-4′), 91.9 (CH_2_, C-9), 63.4 (C-5), 61.5 (CH, C-1), 55.4 (5′-OCH_3_), 53.3 (CH, C-6), 49.1 (CH_2_, C-1′), 31.8 (CH_2_, C-7), 18.1 (5-CH_3_); (−)-ESI-MS (*m*/*z*) 314.9 [M – H]^−^.

#### 8-(4-Methoxybenzyl)-7,7a-dimethyl-1,3,4,4a-tetrahydro-3,7-epiminofuro[3,4-*b*]pyridine-2,5-dione (**23**)

MW (C_17_H_20_N_2_O_4_) 316.4 g/mol; TLC (9:1 CHCl_3_/MeOH) *R*_*f*_ = 0.47; mp 182–186 °C; FT-IR (ATR) *v* (cm^–1^) 1785 and 1694 (C=O), 1510 (Ar), 1238 (C–O); ^1^H NMR (400 MHz, CDCl_3_) δ 7.18 (d, *J* = 8.8 Hz, 2H, H-3′), 6.86 (d, *J* = 8.8 Hz, 2H, H-4′), 4.13 (d, *J* = 15.2 Hz, 1H, H-1′), 3.78 (s, 3H, 5′-OCH_3_), 3.69 (d, *J* = 15.2 Hz, 1H, H-1′), 3.36–3.34 (m, 1H, H-3), 2.58 (dd, *J* = 10.4 Hz, *J*′ = 1.2 Hz, 1H, H-4a), 2.25 (ddd, *J* = 14.4 Hz, *J*′ = 4.4 Hz, *J*″ = 1.2 Hz, 1H, H-4_endo_), 2.01 (ddd, *J* = 14.4 Hz, *J*′ = 10.4 Hz, *J*″ = 0.8 Hz, 1H, H-4_exo_), 1.54 (s, 3H, 7-CH_3_), 1.48 (s, 3H, 7a-CH_3_); ^13^C{^1^H} NMR (100 MHz, CDCl_3_) δ 176.0 (C-5), 174.1 (C-2), 159.1 (C-5′), 129.7 (C-2′), 129.1 (2CH, C-3′), 114.2 (2CH, C-4′), 101.7 (C-7), 61.8 (C-7a), 55.3 (5′-OCH_3_), 54.8 (CH, C-3), 48.7 (CH_2_, C-1′), 45.6 (CH, C-4a), 24.6 (CH_2_, C-4), 18.2 (7a-CH_3_), 18.0 (7-CH_3_); ESI-MS (*m*/*z*) 317.1 [M + H]^+^; ESI-HRMS (*m*/*z*) calcd for C_17_H_21_N_2_O_4_ [M + H]^+^ 317.1496, found 317.1500.

### 8-(4-Methoxybenzyl)-7,7a-dimethyl-4a-phenyl-3,4-dihydro-3,7-epiminofuro[3,4-*b*]pyridine-2,5(1*H*)-dione (**24**)

This compound was prepared following the general procedure described above starting from 1-(4-methoxybenzyl)-5,6-dimethyl-3-oxo-3,4-dihydropyrazin-1-ium bromide (111 mg, 0.34 mmol, 1 equiv) and 2-phenylacrylic acid (76 mg, 0.51 mmol, 1.5 equiv) as the dipolarophile. The reaction was performed at room temperature for 5 h. Final purification by flash chromatography eluting with CH_2_Cl_2_/MeOH (99:1) afforded **24** (67 mg, 50% yield) as a white solid. Crystals suitable for X-ray diffraction were obtained by slow evaporation of a saturated CH_2_Cl_2_ solution of the compound: MW (C_23_H_24_N_2_O_4_) 392.5 g/mol; TLC (9:1 CHCl_3_/MeOH) *R*_*f*_ = 0.78; HPLC (λ = 220 nm) *t*_R_ = 7.73 min (>99% purity); mp 208–210 °C; FT-IR (ATR) *v* (cm^–1^) 1755 and 1703 (C=O), 1510 (Ar), 1246 (C–O); ^1^H NMR (400 MHz, CDCl_3_) δ 7.45–7.43 (m, 2H, H-10), 7.41–7.34 (m, 3H, H-11, H-12), 7.22 (d, *J* = 8.8 Hz, 2H, H-3′), 6.87 (d, *J* = 8.8 Hz, 2H, H-4′), 6.22 (br, 1H, H-1), 4.24 (d, *J* = 15.2 Hz, 1H, H-1′), 3.80 (s, 3H, 5′-OCH_3_), 3.75 (d, *J* = 15.2 Hz, 1H, H-1′), 3.49–3.48 (m, 1H, H-3), 2.68 (dd, *J* = 15.2 Hz, *J*′ = 4.0 Hz, 1H, H-4_exo_), 2.36 (dd, *J* = 15.2 Hz, *J*′ = 1.2 Hz, 1H, H-4_endo_), 1.61 (s, 3H, 7-CH_3_), 0.94 (s, 3H, 7a-CH_3_); ^13^C{^1^H} NMR (100 MHz, CDCl_3_) δ 175.5 (C-5), 172.9 (C-2), 159.1 (C-5′), 135.1 (C-9), 129.6 (C-2′), 129.0 (2CH, C-3′), 128.6 (2CH, C-11), 128.1 (CH, C-12), 127.7 (2CH, C-10), 114.2 (2CH, C-4′), 98.7 (C-7), 66.0 (C-7a), 55.6 (CH, C-3), 55.3 (5′-OCH_3_), 53.7 (C-4a), 48.6 (CH_2_, C-1′), 33.5 (CH_2_, C-4), 18.6 (7-CH_3_), 17.3 (7a-CH_3_); ESI-MS (*m*/*z*) 393.1 [M + H]^+^; ESI-HRMS (*m*/*z*) calcd for C_23_H_24_N_2_O_4_Na [M + Na]^+^ 415.1628, found 415.1631; ESI-HRMS (*m*/*z*) calcd for (C_23_H_24_N_2_O_4_)_2_Na [2M + Na]^+^ 807.3364, found 807.3358.

### 2-(4-Methoxybenzyl)-8-(methoxycarbonyl)-3,4-dimethyl-6-oxo-2,5-diazabicyclo[2.2.2]oct-2-en-2-ium Trifluoroacetate (**25a**)

Cycloadduct **20a** was treated with 10% TFA in anhydrous CH_2_Cl_2_, and the mixture stirred at room temperature for 4 h. Subsequently, the solvents were removed under reduced pressure, affording quantitatively **25a** as a yellowish oil: MW ([C_18_H_23_N_2_O_4_]^+^[CF_3_COO]^−^) 444.4 g/mol; TLC (9:1 CHCl_3_/MeOH) *R*_*f*_ = 0.02; HPLC (λ = 220 nm) *t*_R_ = 4.21 min (94% purity); ^1^H NMR (400 MHz, CDCl_3_) δ 7.32 (d, *J* = 8.4 Hz, 2H, H-3′), 6.92 (d, *J* = 8.4 Hz, 2H, H-4′), 5.41 (d, *J* = 14.4 Hz, 1H, H-1′), 5.07 (d, *J* = 14.4 Hz, 1H, H-1′), 4.75 (m, 1H, H-1), 3.81 (s, 3H, 5′-OCH_3_), 3.71 (s, 3H, CO_2_CH_3_), 3.31 (dd, *J* = 11.8 Hz, *J*′ = 5.2 Hz, 1H, H-8), 2.86 (s, 3H, 3-CH_3_), 2.44 (t, *J* = 11.8 Hz, 1H, H-7_endo_), 1.77 (s, 3H, 4-CH_3_), 1.42–1.39 (m, 1H, H-7_exo_); ^13^C{^1^H} NMR (100 MHz, CDCl_3_) δ 190.1 (C-3), 172.7 (*C*O_2_CH_3_), 167.0 (C-6), 161.1 (C-5′), 160.4 (q, *J* = 38 Hz, CF_3_*C*OO^–^), 131.1 (2CH, C-3′), 120.5 (C-2′), 115.6 (q, *J* = 289 Hz, *C*F_3_COO^–^), 115.0 (2CH, C-4′), 64.2 (CH, C-1), 61.0 (C-4), 58.7 (CH_2_, C-1′), 55.4 (5′-OCH_3_), 53.3 (CO_2_*C*H_3_), 51.1 (CH, C-8), 29.5 (CH_2_, C-7), 19.3 (3-CH_3_), 17.0 (4-CH_3_); ^19^F NMR (377 MHz, CDCl_3_) δ −76.8 (s, 3F, CF_3_COO^–^); ESI-MS (*m*/*z*) 331.2 [C_18_H_23_N_2_O_4_]^+^; ESI-HRMS (*m*/*z*) calcd for C_18_H_23_N_2_O_4_ [M]^+^ 331.1652, found 331.1656.

### Methyl 5-(4-Methoxybenzyl)-1-methyl-6-methylene-3-oxo-2,5-diazabicyclo[2.2.2]octane-7-carboxylate (**21a**)

This compound was prepared by deprotonation of iminium salt **25a** with triethylamine in anhydrous acetonitrile. Final purification by flash chromatography (elution with 98:2 CH_2_Cl_2_/MeOH) afforded quantitatively the title heterocycle as a white solid. Crystals suitable for X-ray diffraction were obtained by recrystallization from EtOAc: MW (C_18_H_22_N_2_O_4_) 330.4 g/mol; TLC (9:1 CHCl_3_/MeOH) *R*_*f*_ = 0.35; HPLC (λ = 220 nm) *t*_R_ = 4.29 min (94% purity); mp 132–134 °C; FT-IR (ATR) *v* (cm^–1^) 3072 (C–H), 1687 (C=O), 1510 (Ar), 1139 (C–O); ^1^H NMR (400 MHz, CDCl_3_) δ 7.25 (d, *J* = 8.4 Hz, 2H, H-3′), 6.87 (d, *J* = 8.4 Hz, 2H, H-4′), 4.25 (s, 2H, H-1′), 3.79 (s, 3H, 5′-OCH_3_), 3.71 (s, 3H, CO_2_CH_3_), 3.69 (d, *J* = 1.8 Hz, 1H, H-9), 3.66 (d, *J* = 1.8 Hz, 1H, H-9), 3.61 (t, *J* = 2.0 Hz, 1H, H-4), 2.93 (dd, *J* = 10.4 Hz, *J*′ = 4.8 Hz, 1H, H-7), 2.55 (ddd, *J* = 13.6 Hz, *J*′ = 4.8 Hz, *J*″ = 2.0 Hz, 1H, H-8_exo_), 2.05 (ddd, *J* = 13.6 Hz, *J*′ = 10.4 Hz, *J*″ = 2.0 Hz, 1H, H-8_endo_), 1.57 (s, 3H, 1-CH_3_); ^13^C{^1^H} NMR (100 MHz, CDCl_3_) δ 173.8 (C-3), 172.4 (*C*O_2_CH_3_), 159.5 (C-5′), 151.0 (C-6), 130.7 (C-2′), 129.1 (2CH, C-3′), 114.7 (2CH, C-4′), 77.0 (CH_2_, C-9), 59.7 (CH, C-4), 59.3 (C-1), 55.9 (5′-OCH_3_), 55.1 (CH_2_, C-1′), 52.8 (CO_2_*C*H_3_), 50.2 (CH, C-7), 29.7 (CH_2_, C-8), 20.7 (1-CH_3_); ESI-MS (*m*/*z*) 331.2 [M + H]^+^; ESI-HRMS (*m*/*z*) calcd for C_18_H_23_N_2_O_4_ [M + H]^+^ 331.1652, found 331.1661; ESI-HRMS (*m*/*z*) calcd for C_18_H_22_N_2_O_4_Na [M + Na]^+^ 353.1472, found 353.1480.

## Data Availability

The data underlying this study are available in the published article and its Supporting Information.

## Supporting Information Available

Characterization data (NMR, FT-IR, HPLC, ESI-MS, and HRMS) for all compounds (PDF)
