# Supplementary material for: Exploiting 3-Oxidopyraziniums toward Diazabicyclo[3.2.1]octanes and Their Conversion into Diazabicyclo[2.2.2]octanes and Tricyclic Lactone-Lactams
Source: J Org Chem. 2024 Feb 8;89(5):2904–15. doi: 10.1021/acs.joc.3c02273 (PMC10913038; doi:10.1021/acs.joc.3c02273)
Supplement: Supplementary file 1 — jo3c02273_si_001.pdf [file jo3c02273_si_001.pdf]

Supporting Information

Exploiting 3-Oxidopyraziniums towards Diazabicyclo[3.2.1]octanes and their Conversion into Diazabicyclo[2.2.2]octanes and Tricyclic Lactone-Lactams

Gerard Riesco-Llach,[a] Marta Planas,[a,*] Lidia Feliu[a,*] and John A. Joule[b]

[a] LIPPSO, Department of Chemistry, Maria Aurèlia Capmany 69, Universitat de Girona, 17003 Girona, Spain. E-mail: lidia.feliu@udg.edu; marta.planas@udg.edu

[b] The School of Chemistry, The University of Manchester, Manchester M13 9PL, U. K. E-mail: John.Joule@manchester.ac.uk

Table of contents

1. Spectroscopic data: NMR, IR, HPLC, ESI-MS and HRMS S4

5,6-Dimethyl-2(1*H*)-pyrazinone (**18**)... S4

1-(4-methoxybenzyl)-5,6-dimethyl-3-oxo-3,4-dihydropyrazin-1-ium bromide (**19**)................. S8

Methyl 8-(4-methoxybenzyl)-5-methyl-4-methylene-2-oxo-3,8-diazabicyclo[3.2.1]octane-6-carboxylate (**20a**)... S15

tert-Butyl 8-(4-methoxybenzyl)-5-methyl-4-methylene-2-oxo-3,8-diazabicyclo[3.2.1]octane-6-carboxylate (**20b**)... S23

tert-Butyl 5-(4-methoxybenzyl)-1-methyl-6-methylene-3-oxo-2,5-diazabicyclo[2.2.2]octane-7-carboxylate (**21b**)... S32

Methyl 8-(4-methoxybenzyl)-5,7-dimethyl-4-methylene-2-oxo-3,8-diazabicyclo[3.2.1]octane-6-carboxylate (**20c**)... S34

Methyl 8-(4-methoxybenzyl)-5-methyl-4-methylene-2-oxo-7-phenyl-3,8-diazabicyclo[3.2.1]octane-6-carboxylate (**20da**) and methyl 8-(4-methoxybenzyl)-1-methyl-2-methylene-4-oxo-7-phenyl-3,8-diazabicyclo[3.2.1]octane-6-carboxylate (**20db**) S42

Methyl 8-(4-methoxybenzyl)-5-methyl-4-methylene-7-(4-nitrophenyl)-2-oxo-3,8-diazabicyclo[3.2.1]octane-6-carboxylate (**20ea**) S47

Methyl 8-(4-methoxybenzyl)-5-methyl-4-methylene-7-(4-nitrophenyl)-2-oxo-3,8-diazabicyclo[3.2.1]octane-6-carboxylate (**20ea**) and methyl 8-(4-methoxybenzyl)-1-methyl-2-methylene-7-(4-nitrophenyl)-4-oxo-3,8-diazabicyclo[3.2.1]octane-6-carboxylate (**20eb**) S55

Methyl 5-(4-methoxybenzyl)-1-methyl-6-methylene-3-oxo-7-phenyl-2,5-diazabicyclo[2.2.2]octane-7-carboxylate (**21f**)... S60

8-(4-Methoxybenzyl)-5-methyl-4-methylene-2-oxo-3,8-diazabicyclo[3.2.1]octane-6-carboxylic acid (**22**) and 8-(4-methoxybenzyl)-7,7a-dimethyl-1,3,4,4a-tetrahydro-3,7-epiminofuro[3,4-*b*]pyridine-2,5-dione (**23**) S66

8-(4-Methoxybenzyl)-7,7a-dimethyl-1,3,4,4a-tetrahydro-3,7-epiminofuro[3,4-*b*]pyridine-2,5-dione (**23**).. S70

8-(4-Methoxybenzyl)-7,7a-dimethyl-4a-phenyl-3,4-dihydro-3,7-epiminofuro[3,4-*b*]pyridine-2,5(1*H*)-dione (**24**)... S76

2-(4-Methoxybenzyl)-8-(methoxycarbonyl)-3,4-dimethyl-6-oxo-2,5-diazabicyclo[2.2.2]oct-2-en-2-ium trifluoroacetate (**25a**) S84

Methyl 5-(4-methoxybenzyl)-1-methyl-6-methylene-3-oxo-2,5-diazabicyclo[2.2.2]octane-7-carboxylate (**21a**)... S91

2. Single-crystal X-ray diffraction reports **S99**

Methyl 8-(4-methoxybenzyl)-5-methyl-4-methylene-2-oxo-3,8-diazabicyclo[3.2.1]octane-6-carboxylate (**20a**) S99

Methyl 5-(4-methoxybenzyl)-1-methyl-6-methylene-3-oxo-2,5-diazabicyclo[2.2.2]octane-7-carboxylate (**21a**) S105

8-(4-methoxybenzyl)-7,7a-dimethyl-1,3,4,4a-tetrahydro-3,7-epiminofuro[3,4-*b*]pyridine-2,5-dione (**23**) S111

8-(4-Methoxybenzyl)-7,7a-dimethyl-4a-phenyl-3,4-dihydro-3,7-epiminofuro[3,4-*b*]pyridine-2,5(1*H*)-dione (**24**) S117

1. Spectroscopic data: NMR, IR, HPLC, ESI-MS and HRMS

5,6-Dimethyl-2(1*H*)-pyrazinone (18)

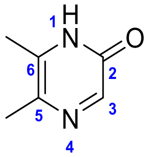

18

^{1}H-NMR (400 MHz, CDCl$_3$), δ (ppm)

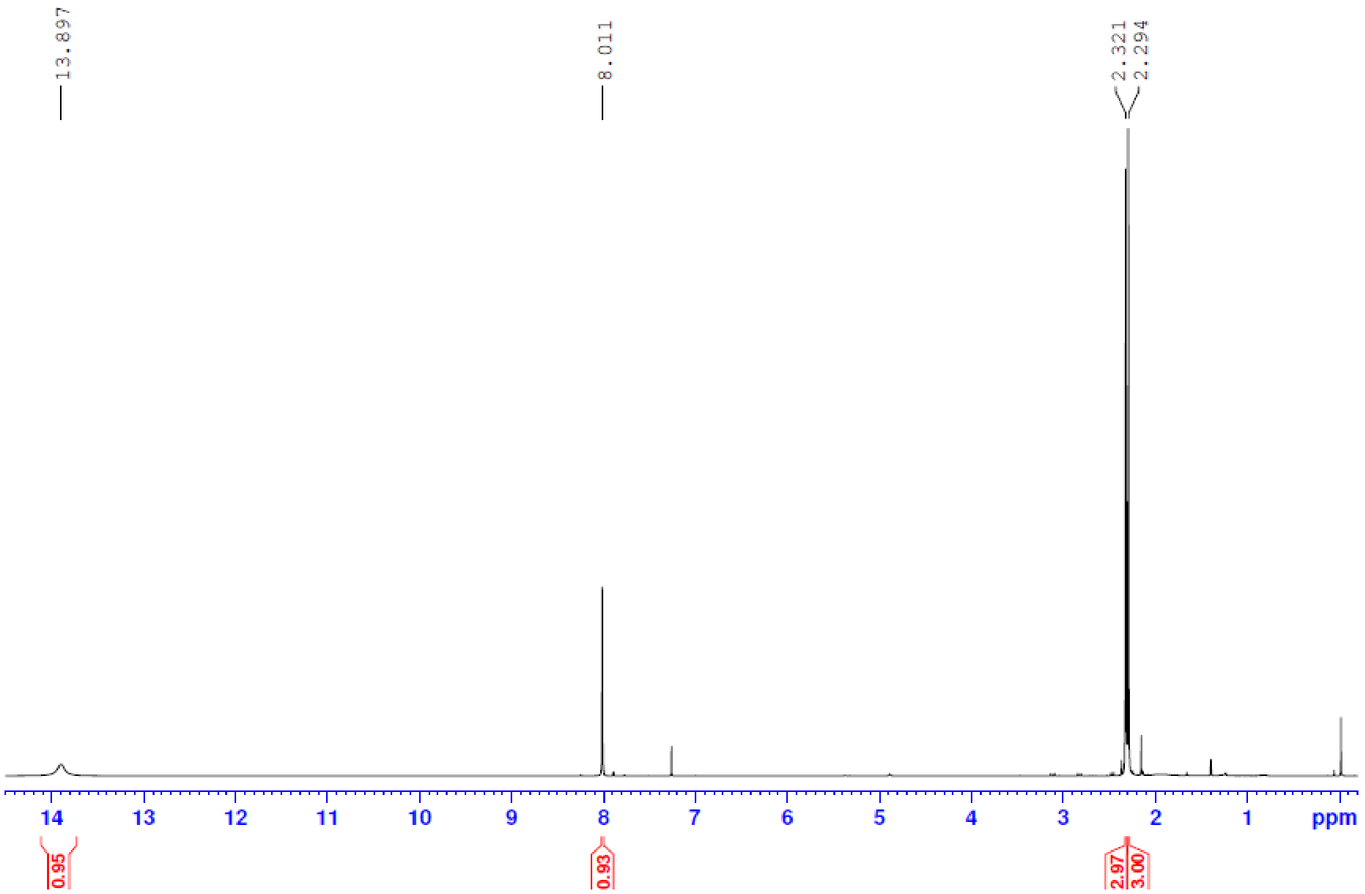

$^{13}C\{^{1}H\}$-NMR (100 MHz, $CDCl_3$), δ (ppm)

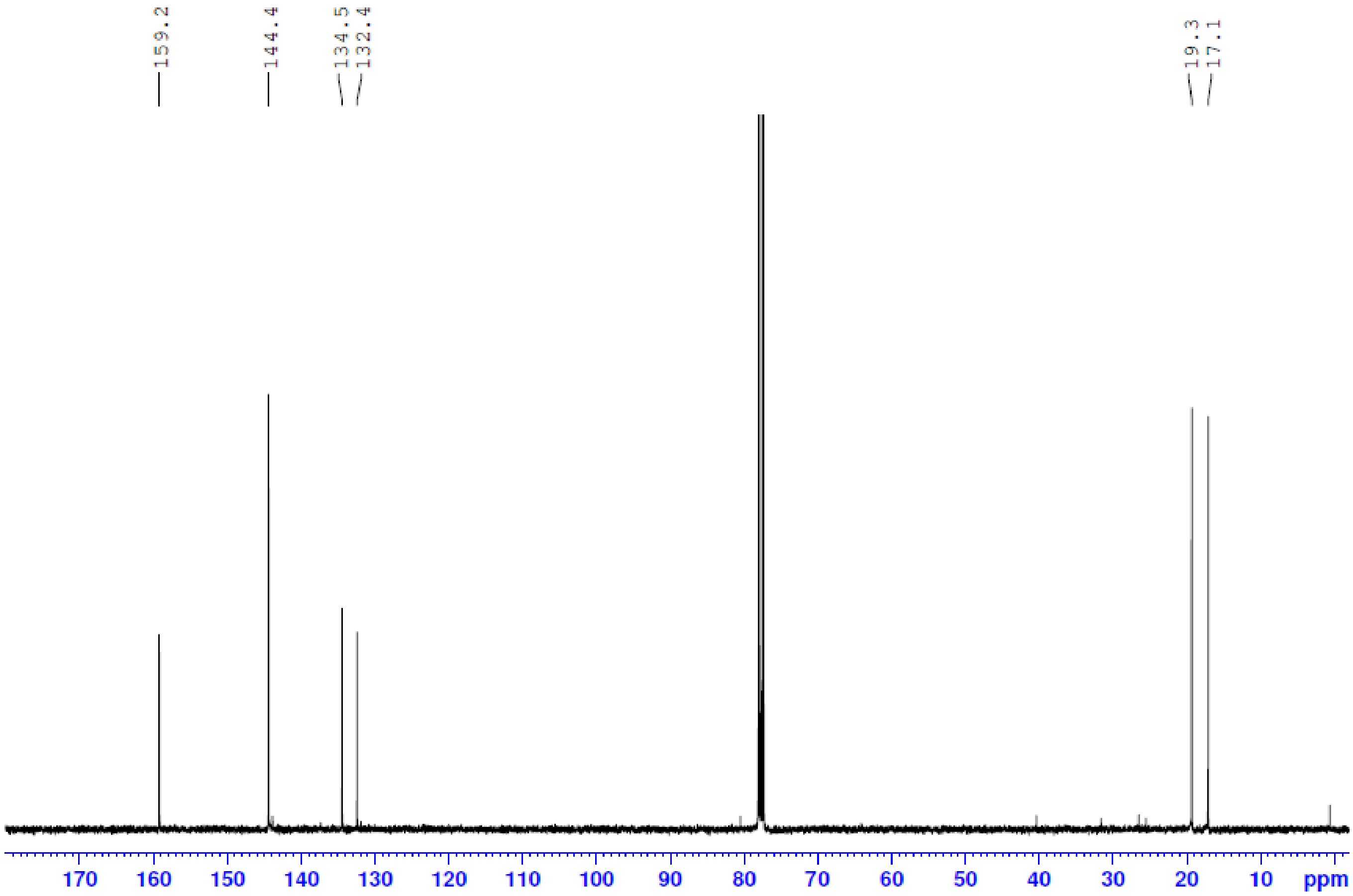

HSQC ^{1}H-^{13}C ($CDCl_3$), δ (ppm)

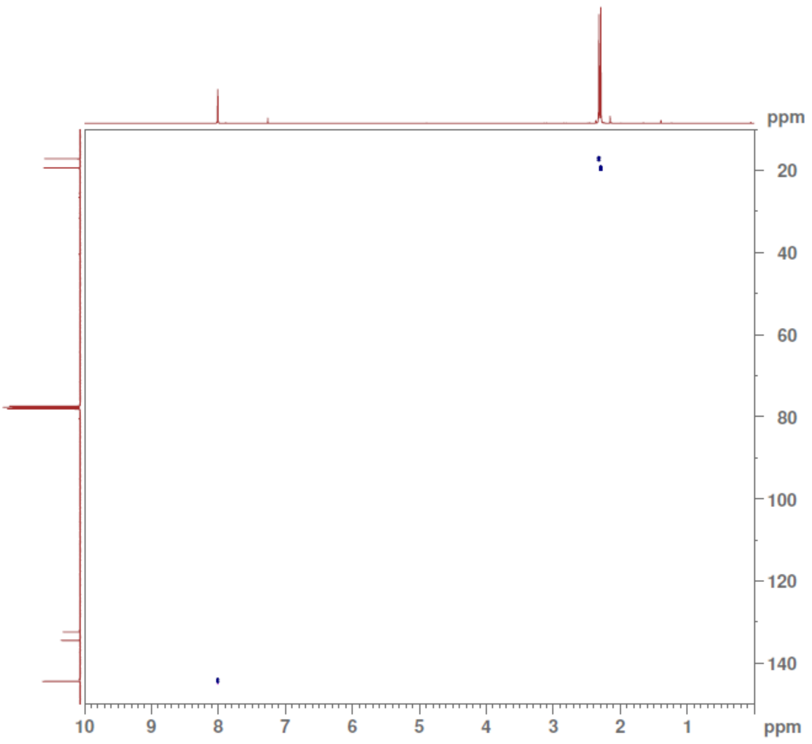

FT-IR (neat), ν (cm^{-1})

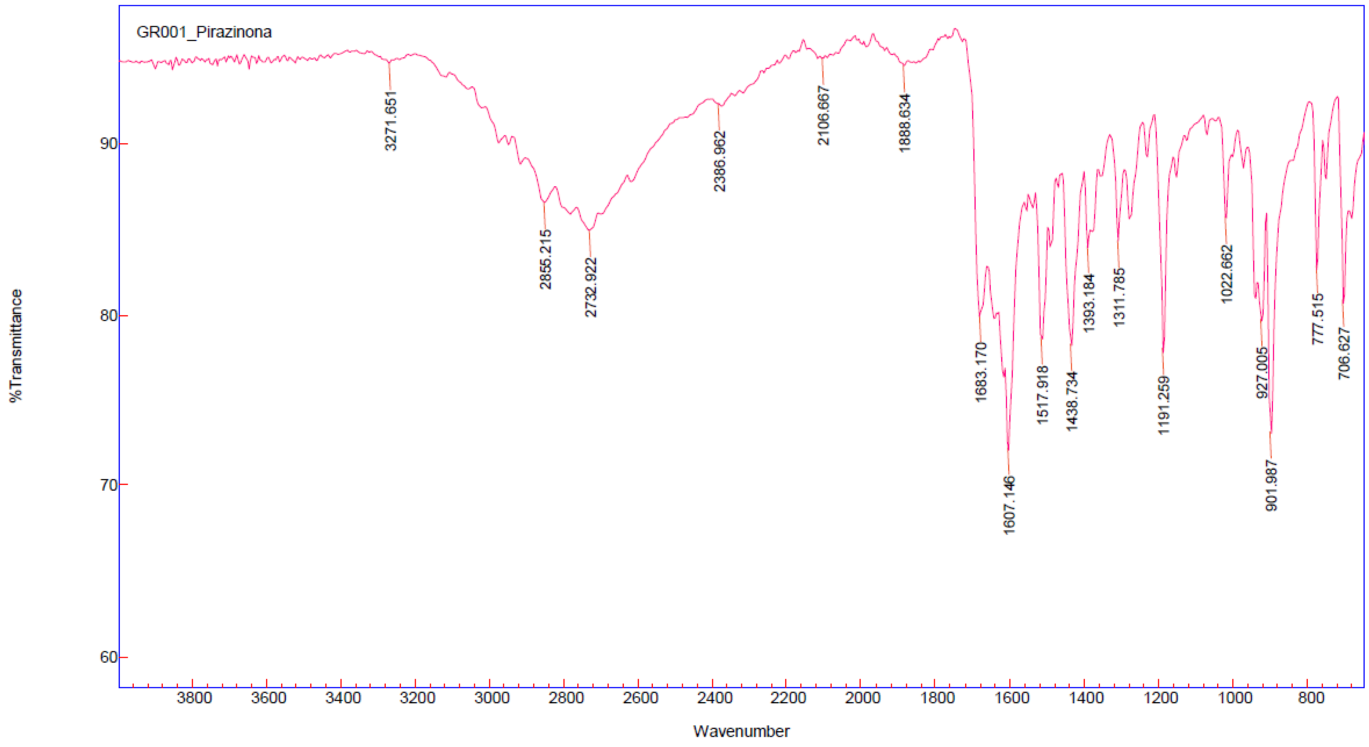

ESI-MS (*m/z*)

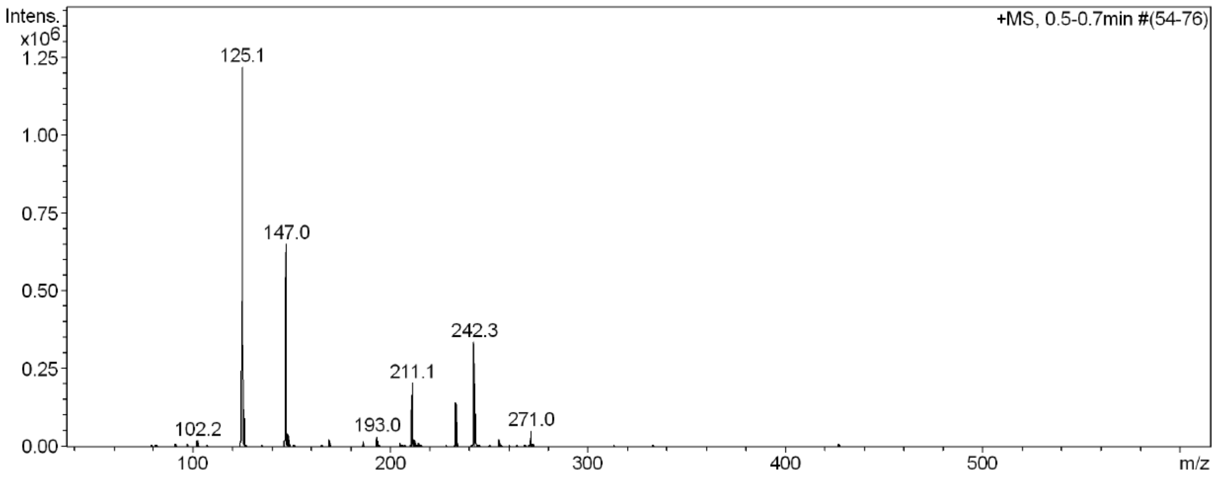

HRMS (*m/z*)

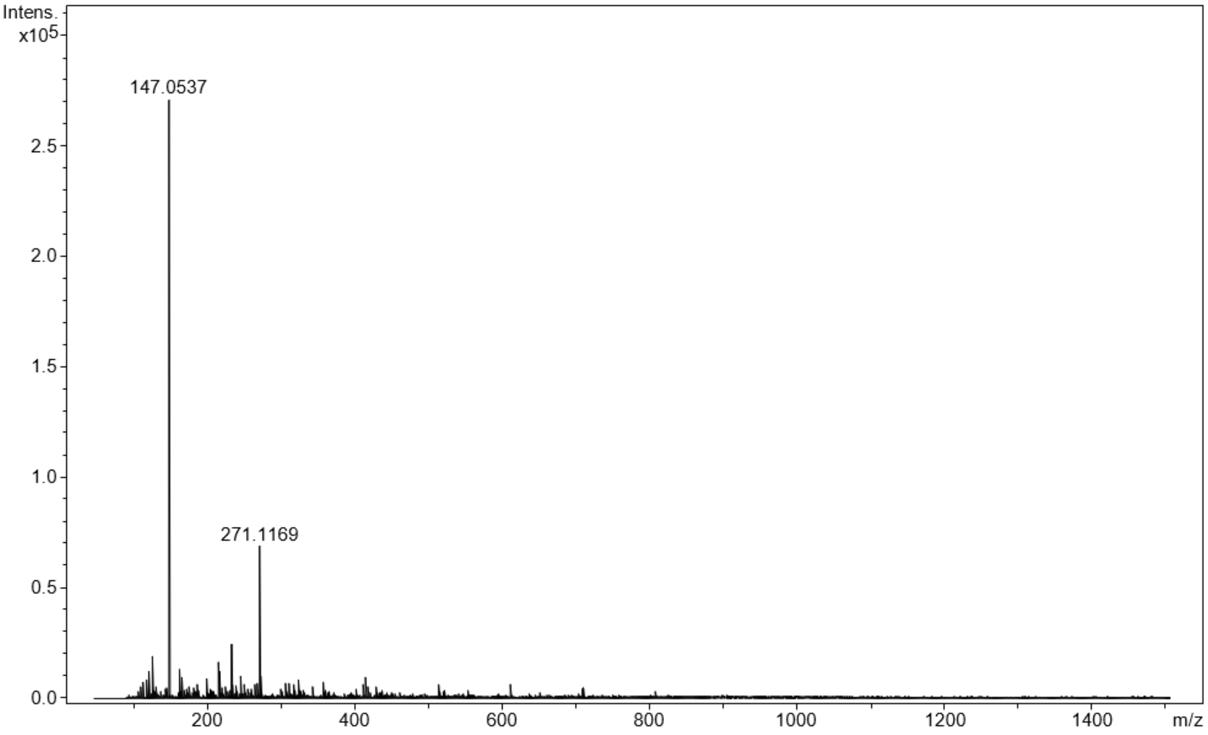

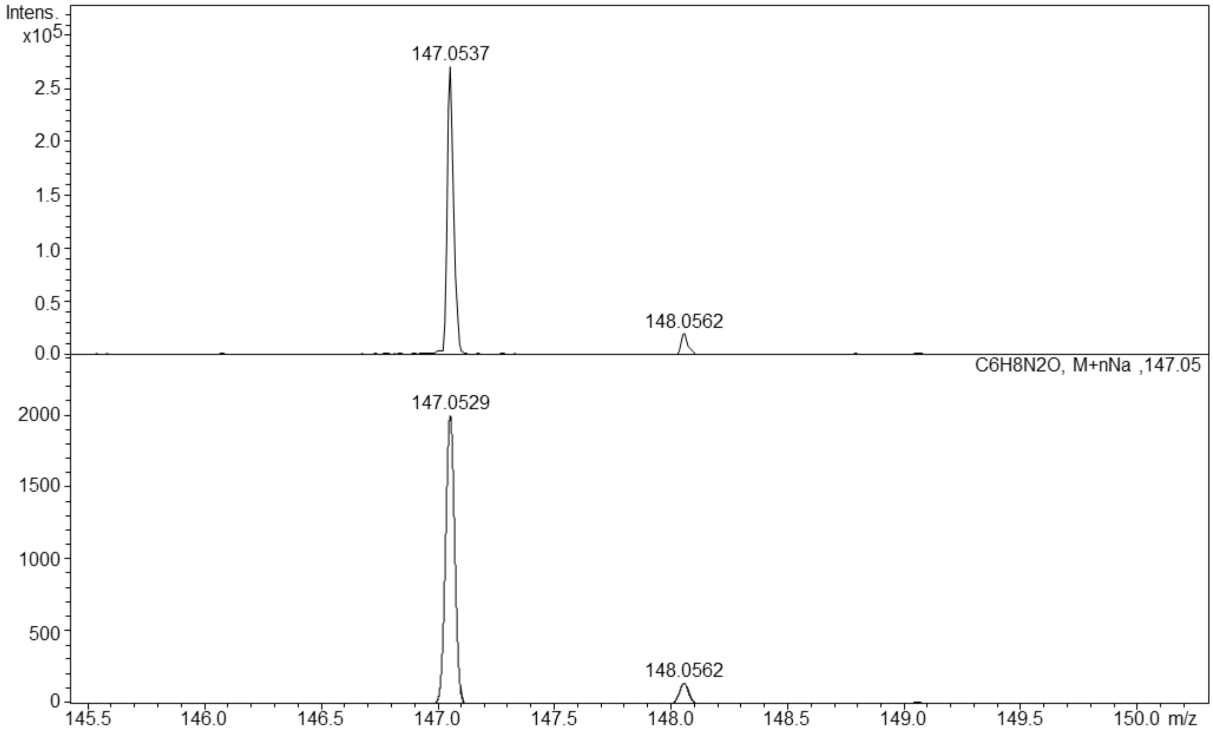

	Molecular formula	Calculated	Found
**[M+Na]+**	$C_6H_8N_2ONa$	147.0529	147.0537
**[2M+Na]+**	$(C_6H_8N_2O)_2Na$	271.1165	271.1169

1-(4-methoxybenzyl)-5,6-dimethyl-3-oxo-3,4-dihydropyrazin-1-ium bromide (19)

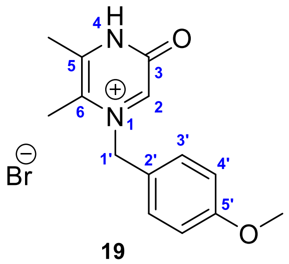

19

^{1}H-NMR (400 MHz, CDCl$_3$), δ (ppm)

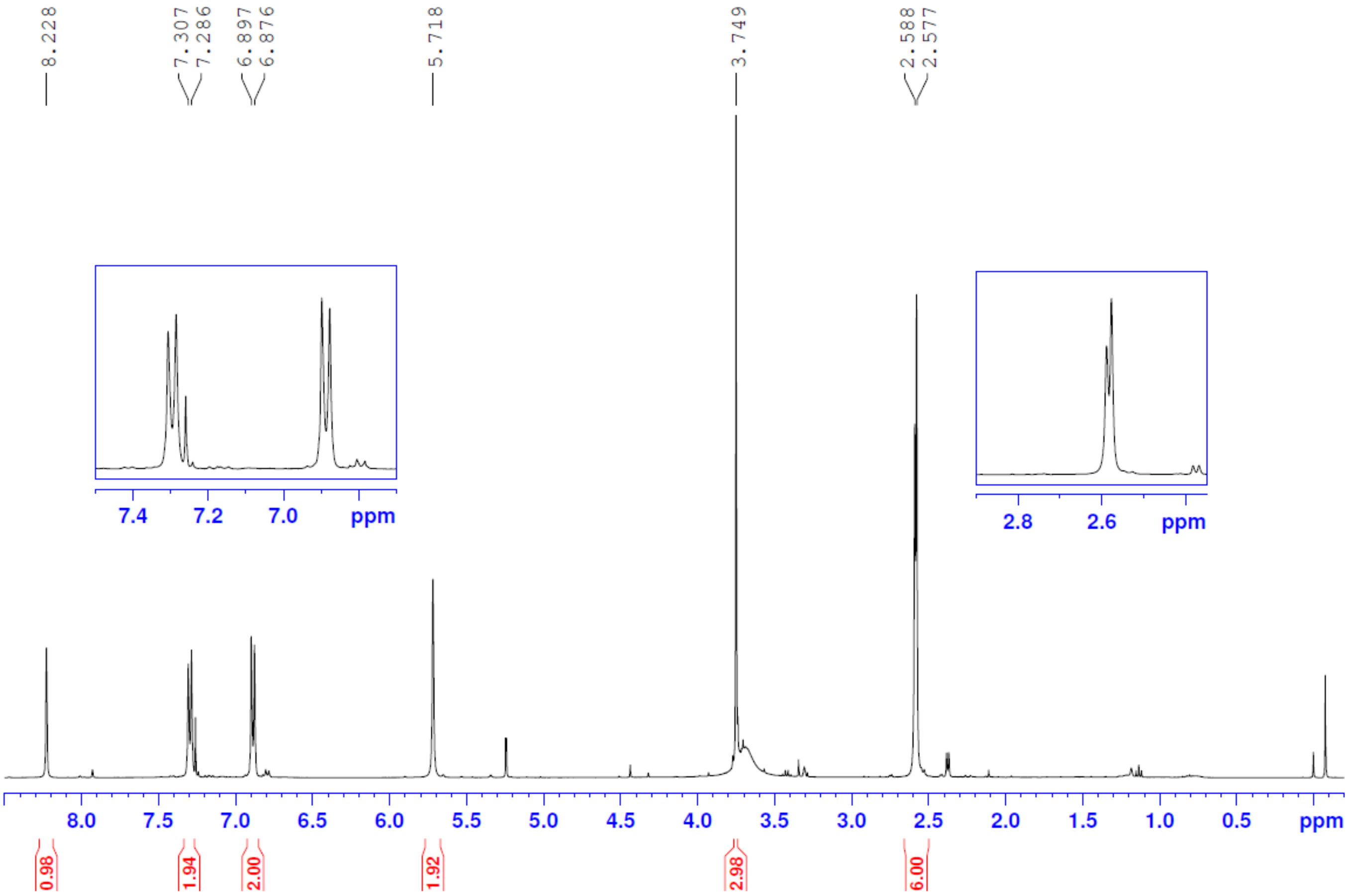

$^{13}C\{^{1}H\}$-NMR (100 MHz, CDCl$_3$), δ (ppm)

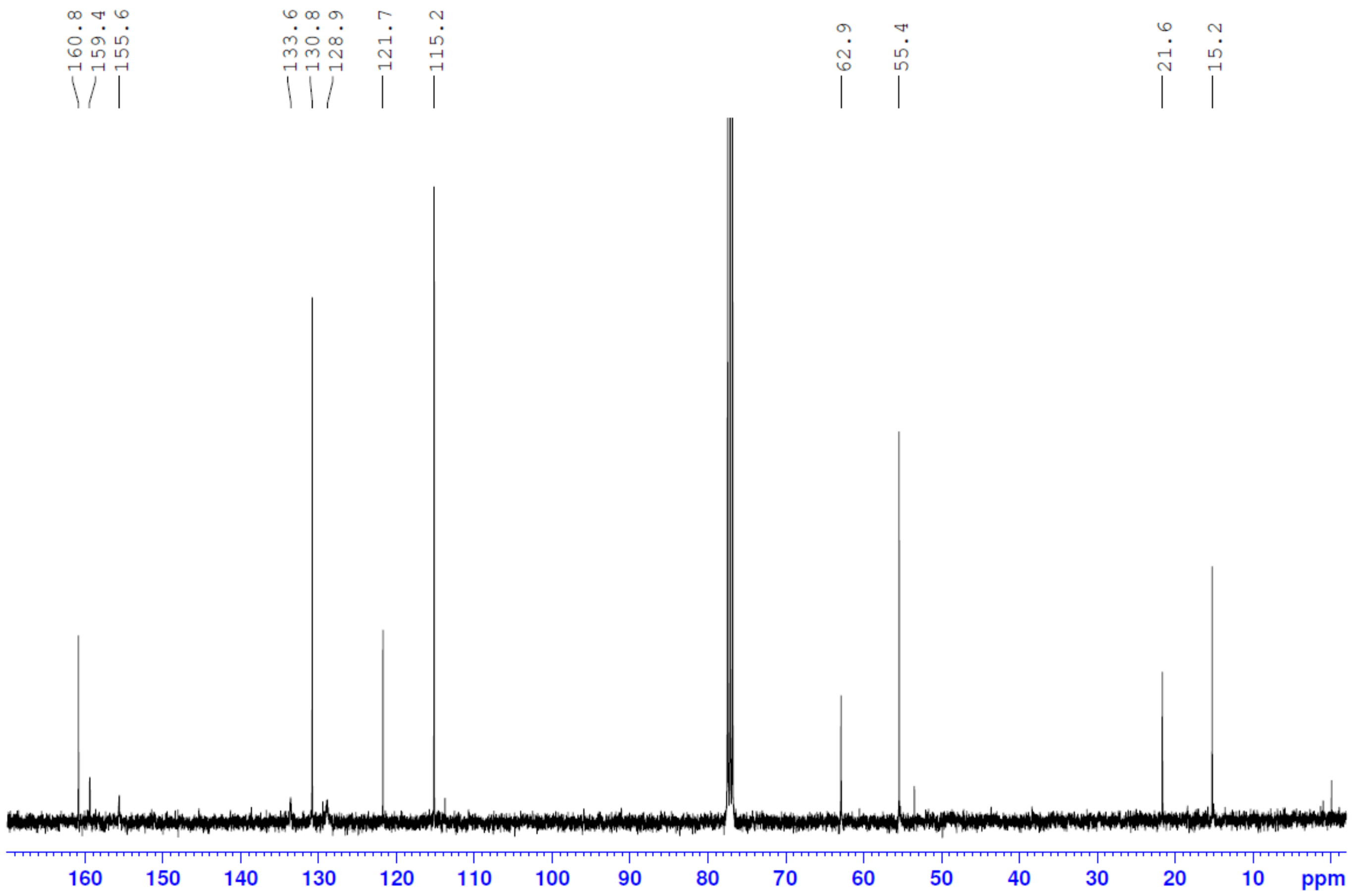

HSQC ^{1}H-^{13}C (CDCl$_3$), δ (ppm)

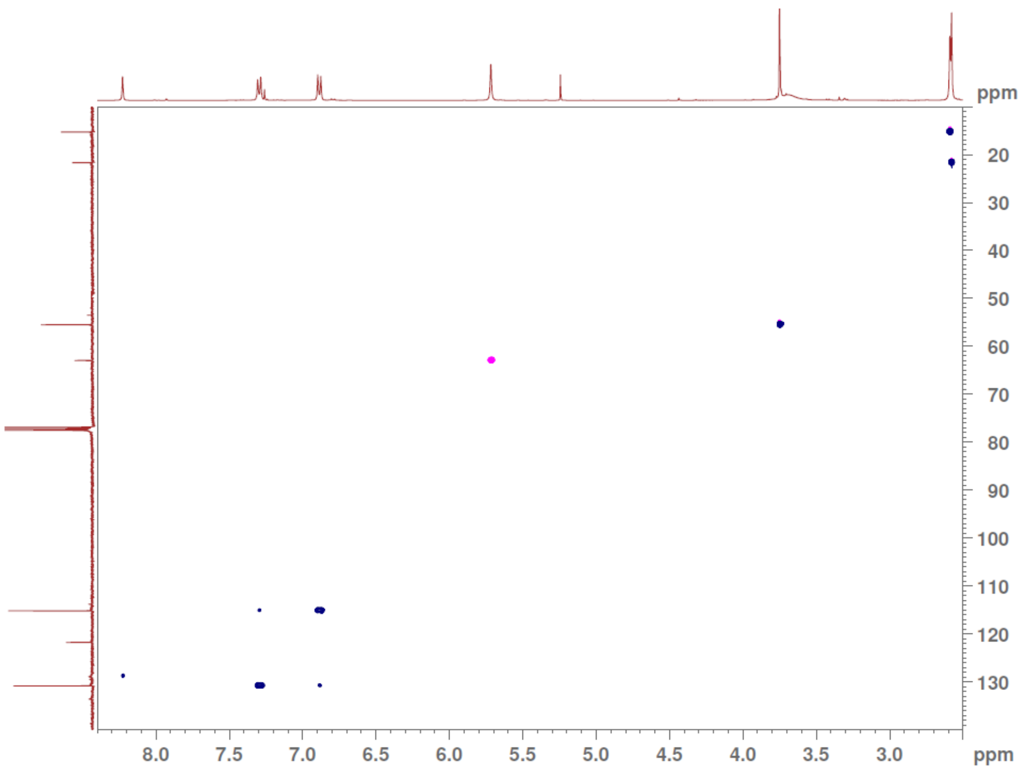

HMBC ^{1}H-^{13}C ($CDCl_3$), δ (ppm)

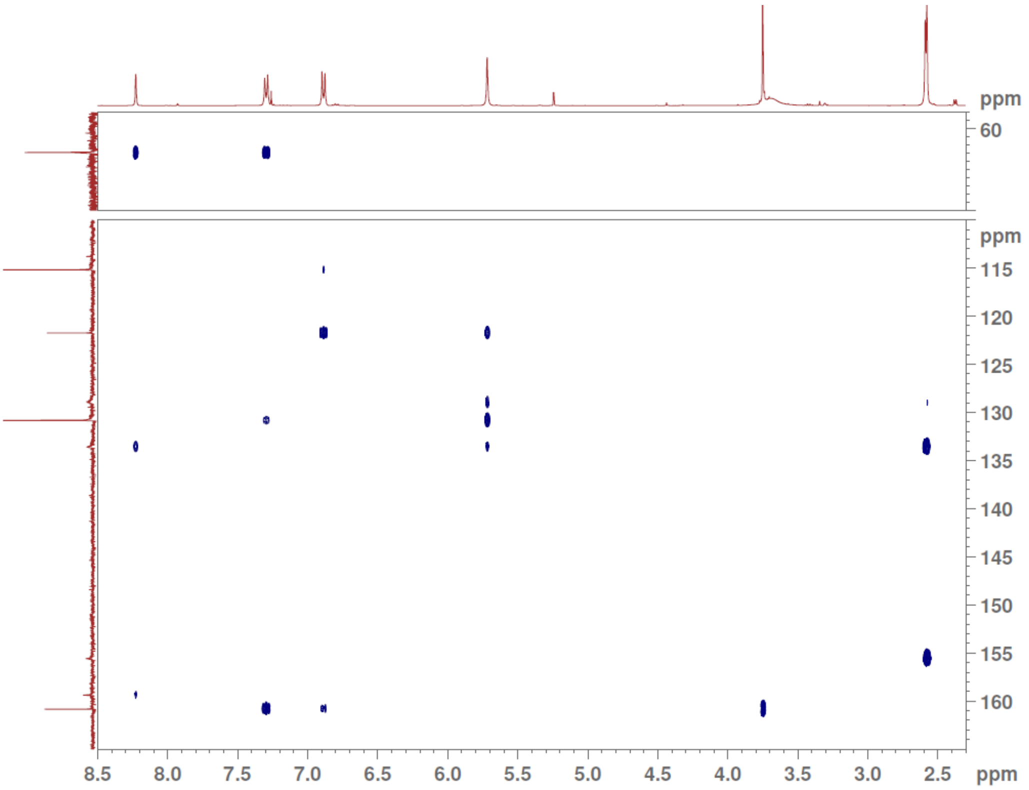

NOESY ^{1}H-^{1}H ($CDCl_3$), δ (ppm)

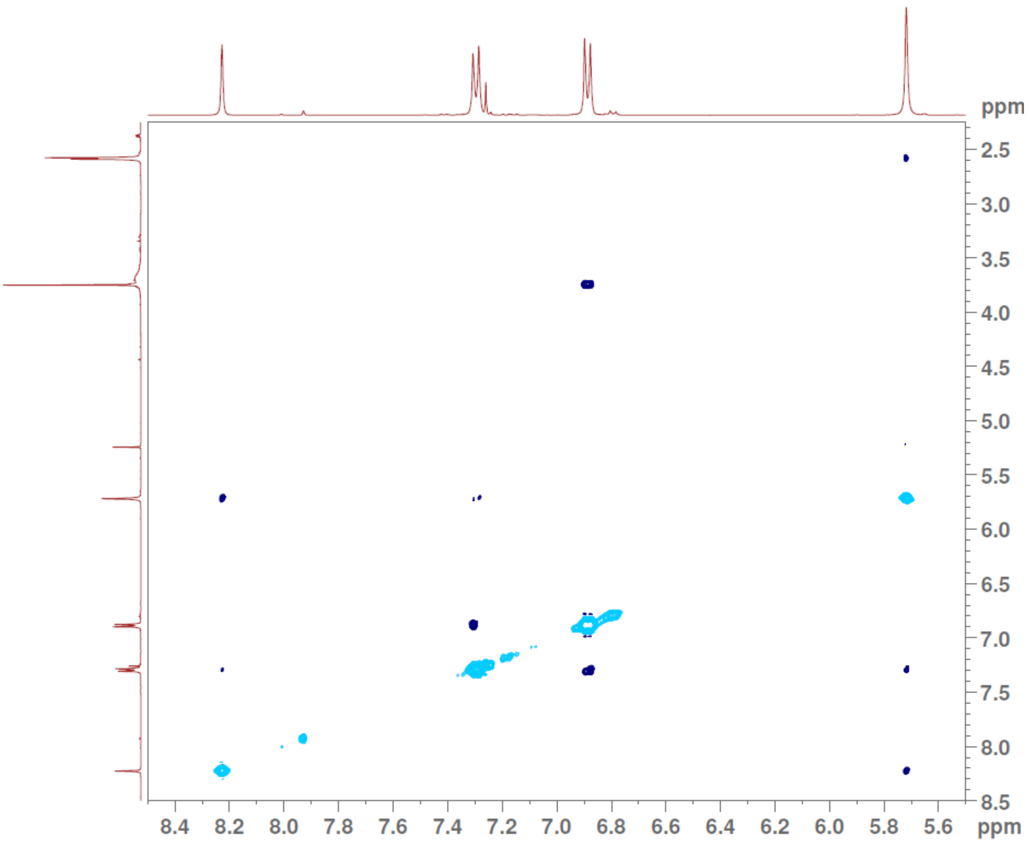

FT-IR (neat), ν (cm^{-1})

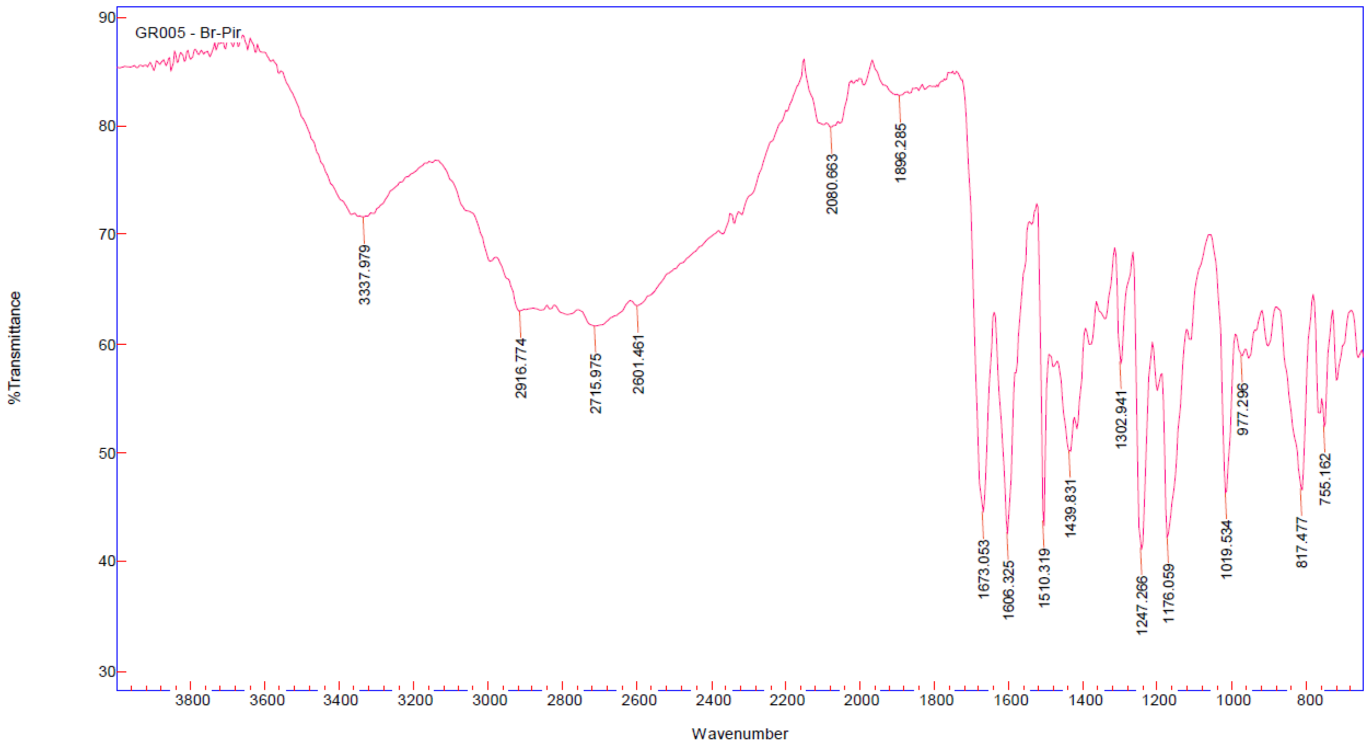

ESI-MS (*m/z*)

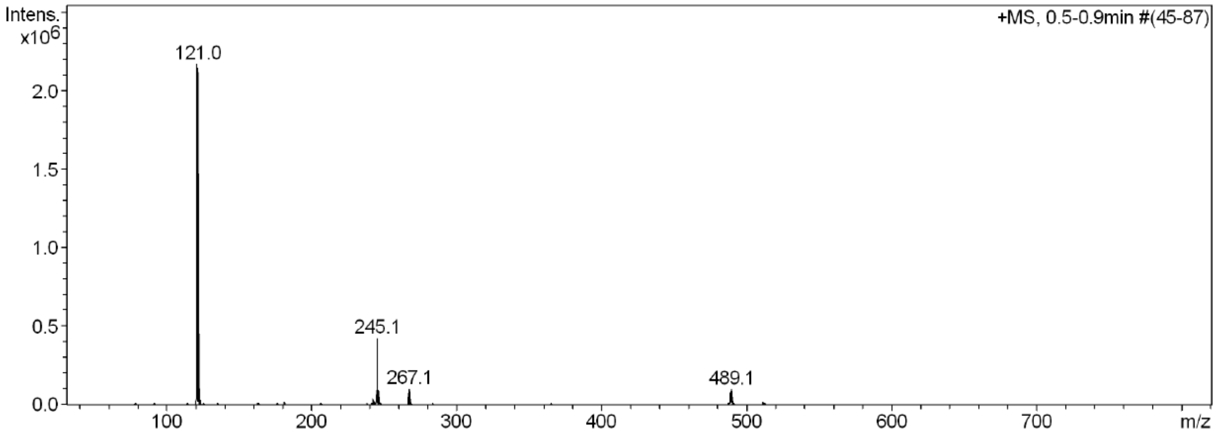

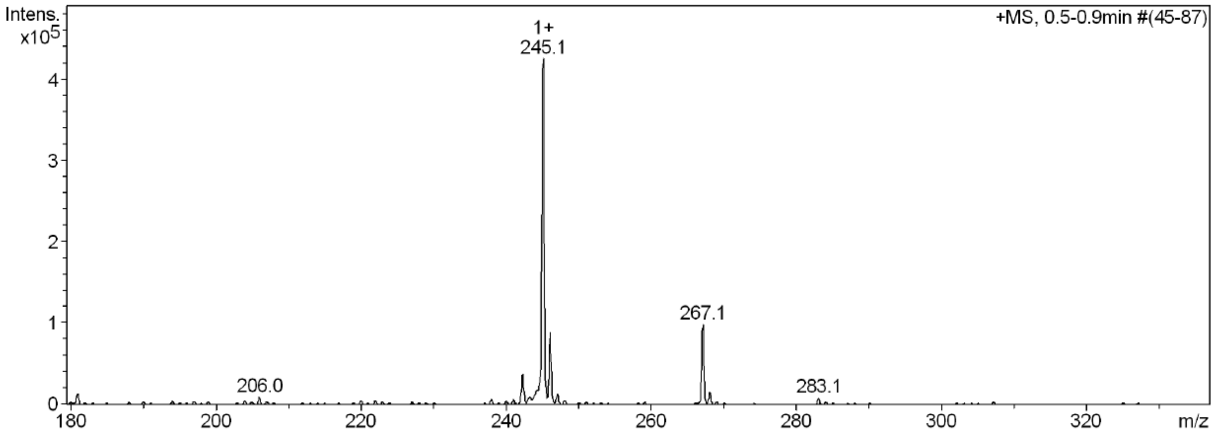

ESI-MS/MS (*m/z* = 245.1)

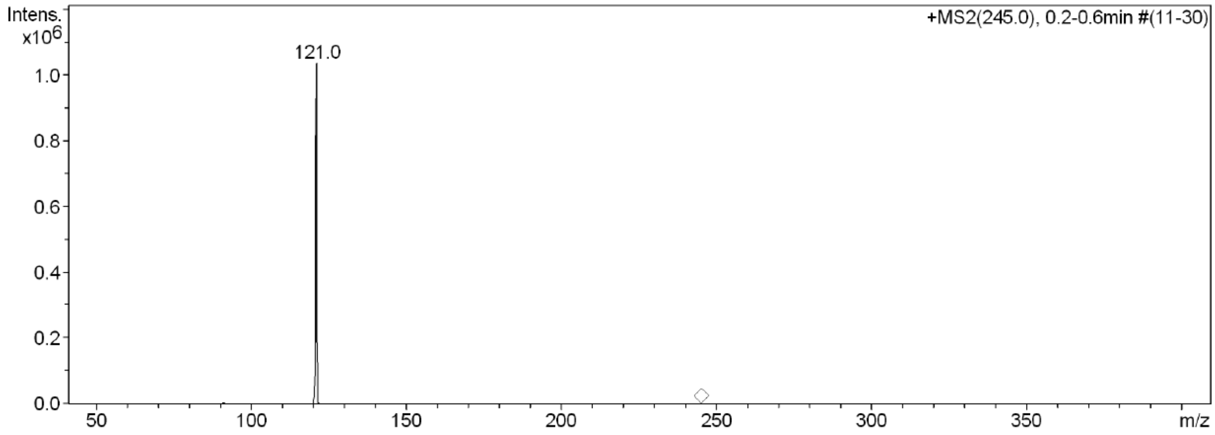

HRMS (*m/z*)

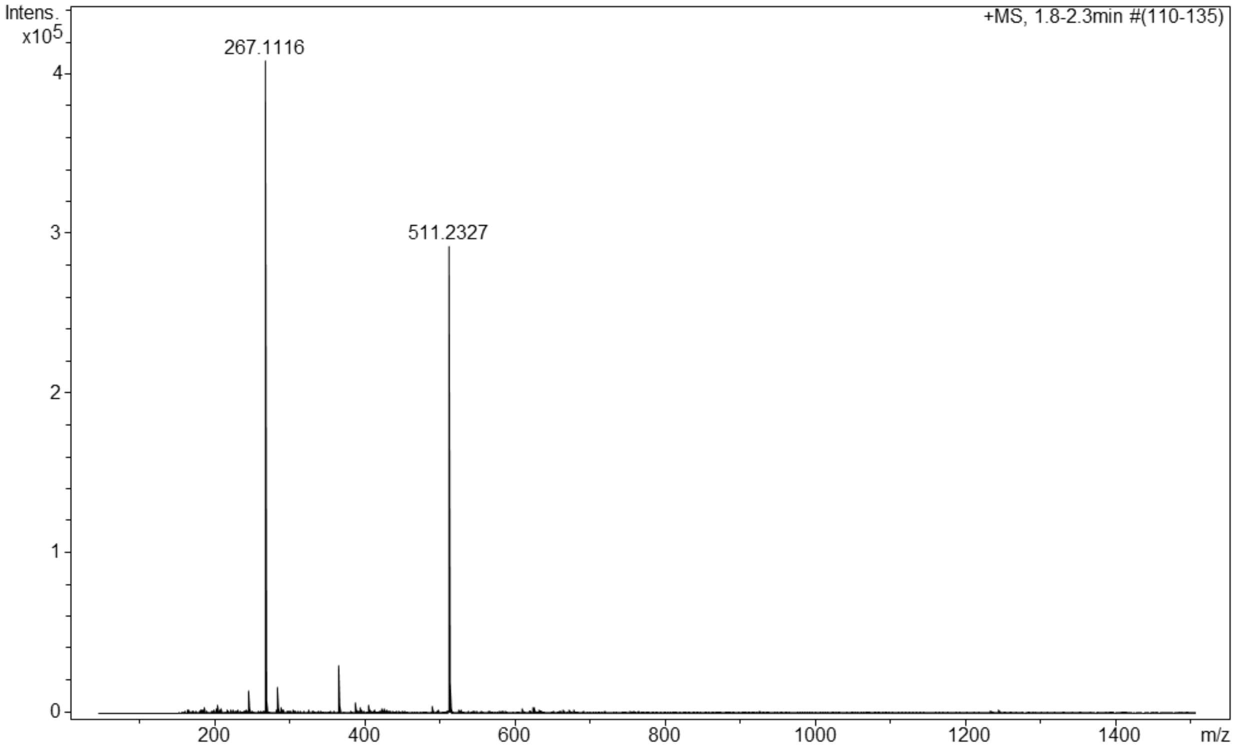

Intens.
x10^5
+MS, 1.8-2.3min #(110-135)
267.1116
511.2327
4
3
2
1
0
200
400
600
800
1000
1200
1400
m/z

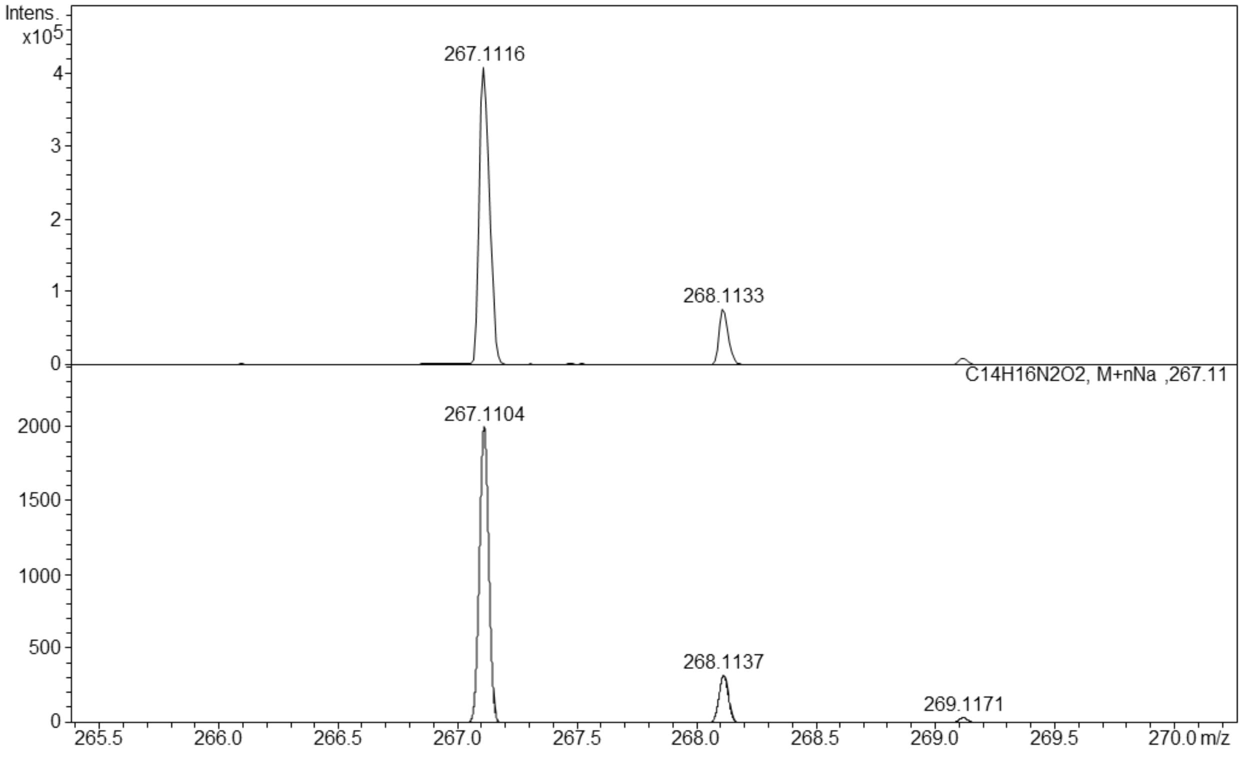

Intens.
x10^5
267.1116
268.1133
C14H16N2O2, M+nNa ,267.11
267.1104
268.1137
269.1171
4
3
2
1
0
2000
1500
1000
500
0
265.5
266.0
266.5
267.0
267.5
268.0
268.5
269.0
269.5
270.0
m/z

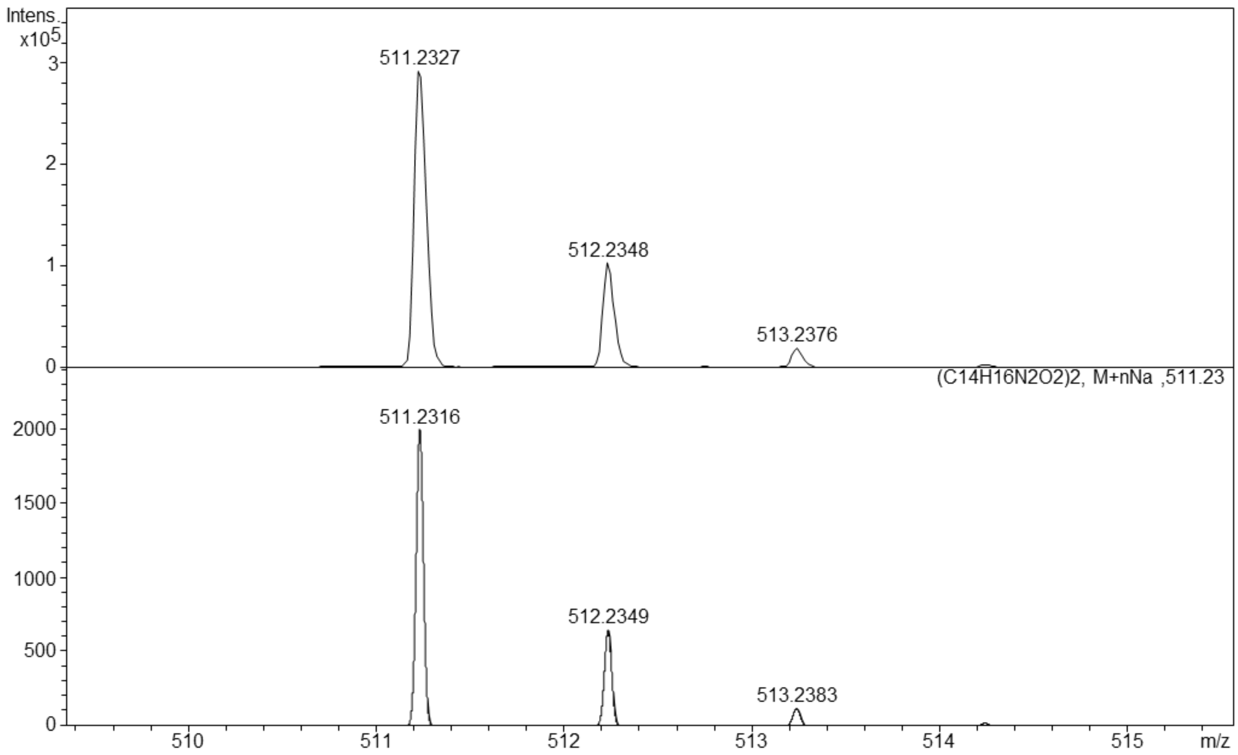

	Molecular formula	Calculated	Found
**[M–H+Na]$^+$**	$C_{14}H_{16}N_2O_2Na$	267.1104	267.1116
**[2M–2H+Na]$^+$**	$(C_{14}H_{16}N_2O_2)_2Na$	511.2316	511.2327

Methyl 8-(4-methoxybenzyl)-5-methyl-4-methylene-2-oxo-3,8-diazabicyclo[3.2.1]octane-6-carboxylate (20a)

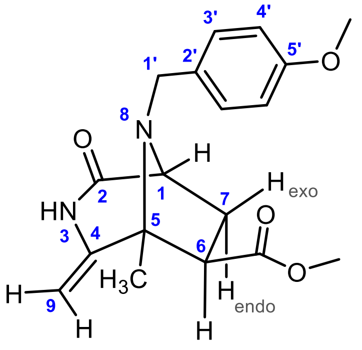

20a

^{1}H-NMR (400 MHz, CDCl$_3$), δ (ppm)

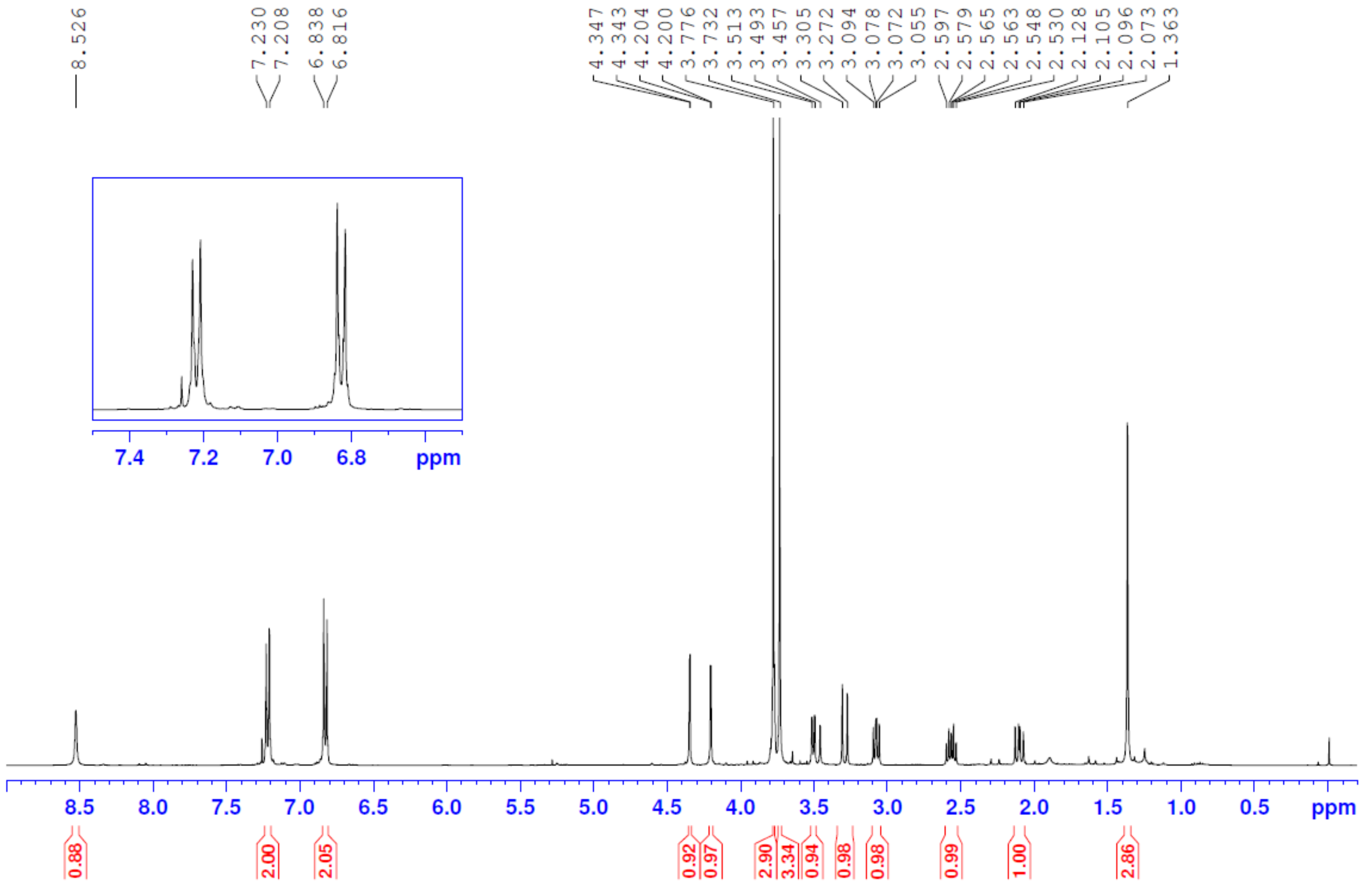

^{1}H-NMR (400 MHz, CDCl$_3$), δ (ppm) – Aliphatic zoom

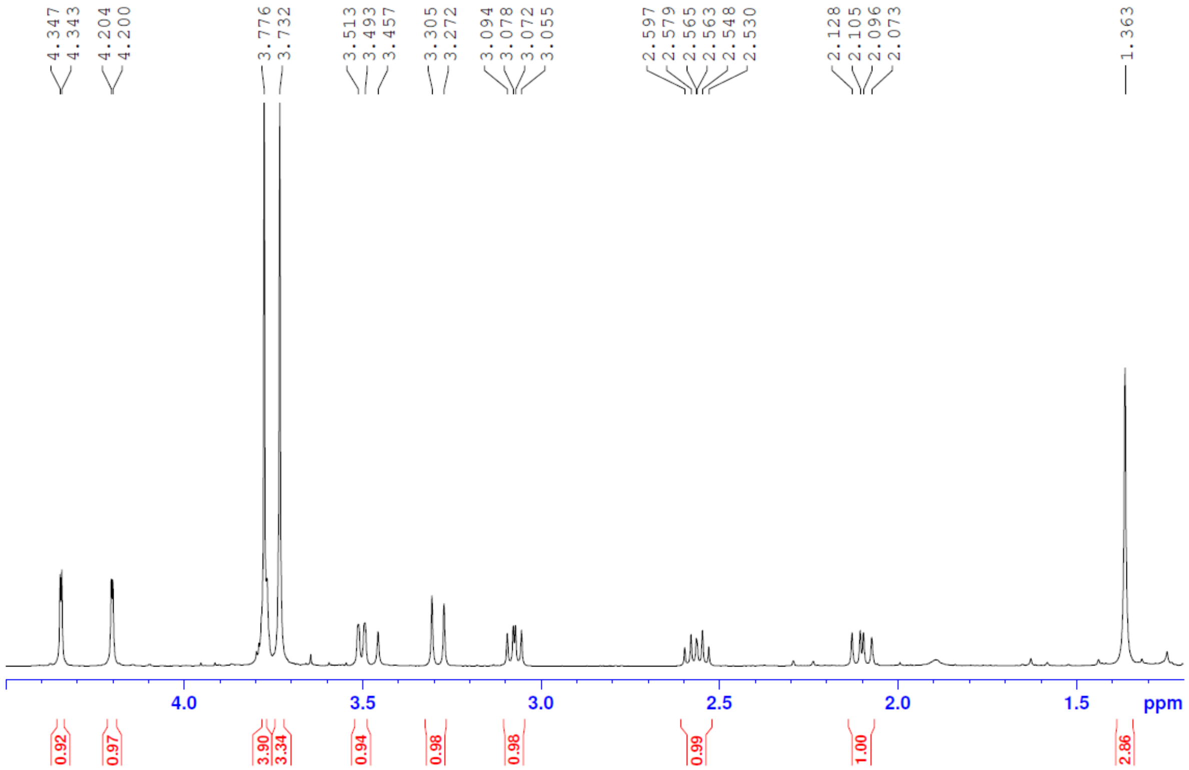

^{13}C{^{1}H}-NMR (100 MHz, CDCl$_3$), δ (ppm)

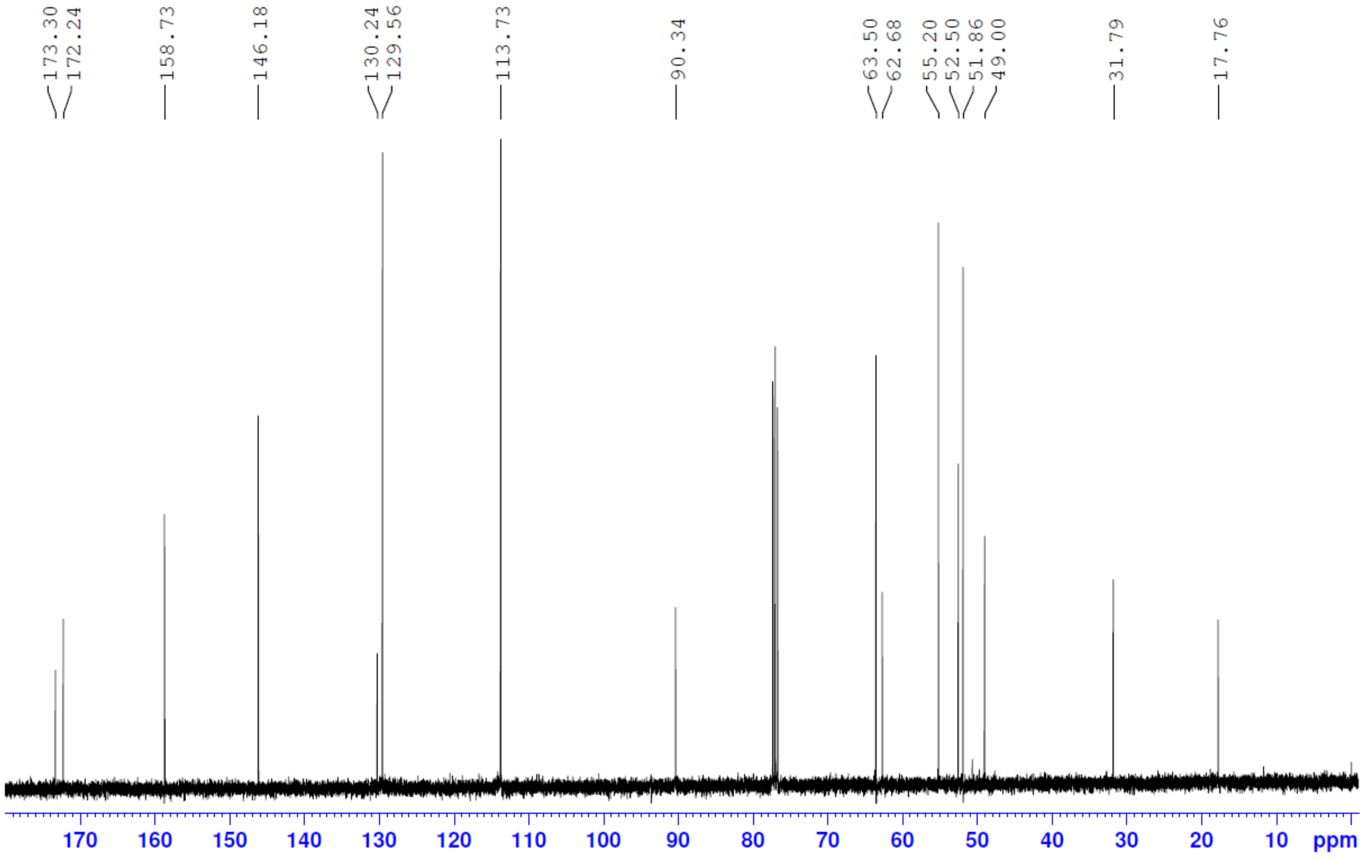

COSY ¹H-¹H (CDCl₃), δ (ppm)

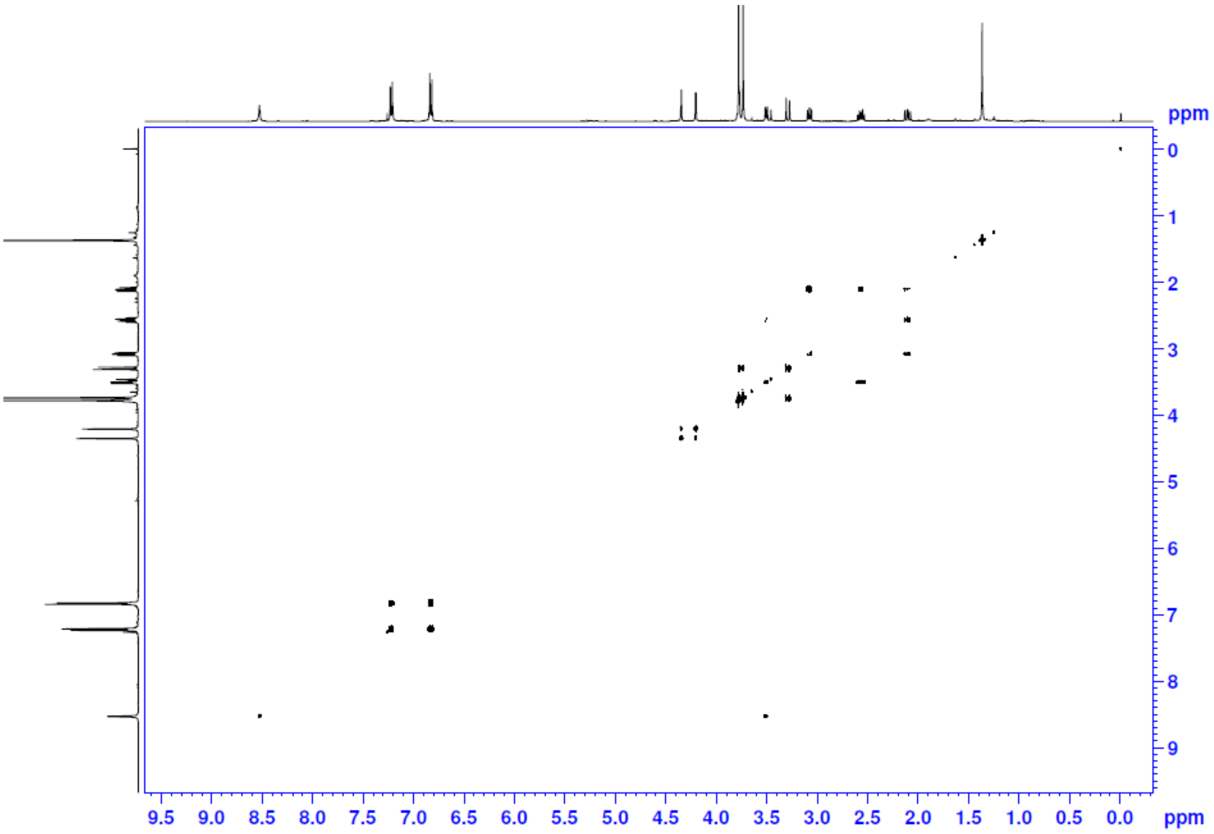

COSY ¹H-¹H (CDCl₃), δ (ppm) – Aliphatic Zoom

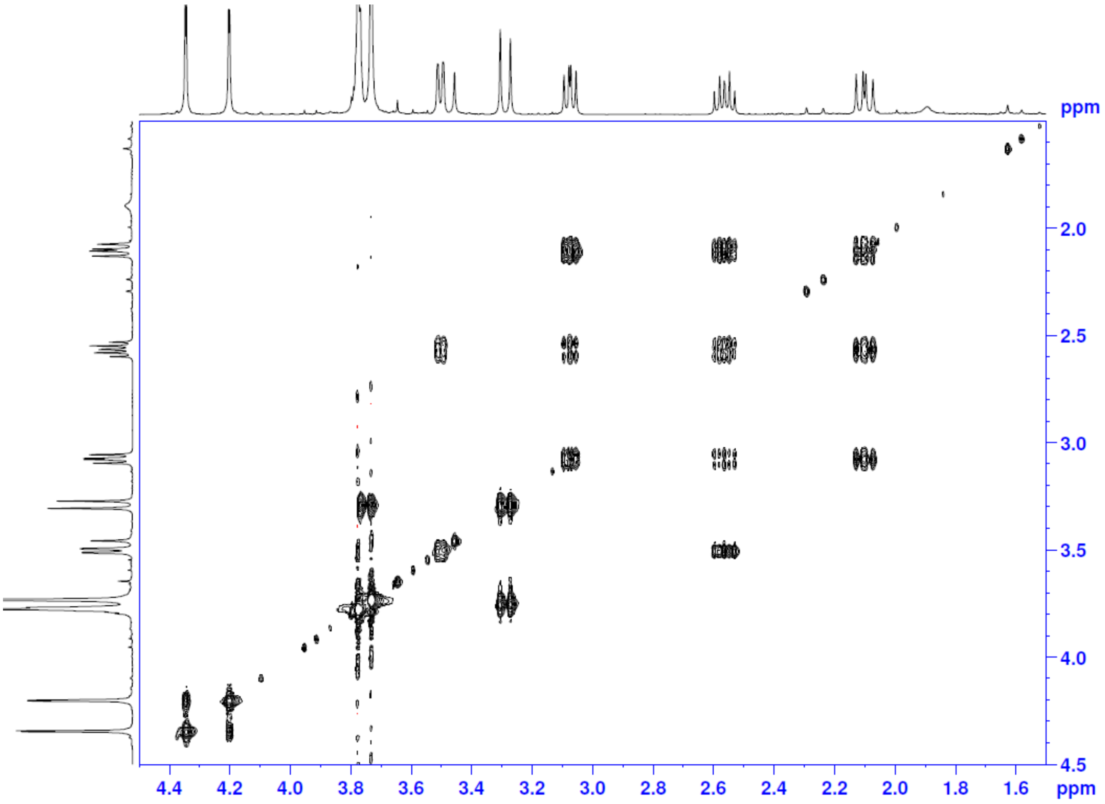

NOESY ^{1}H-^{1}H ($CDCl_3$), δ (ppm)

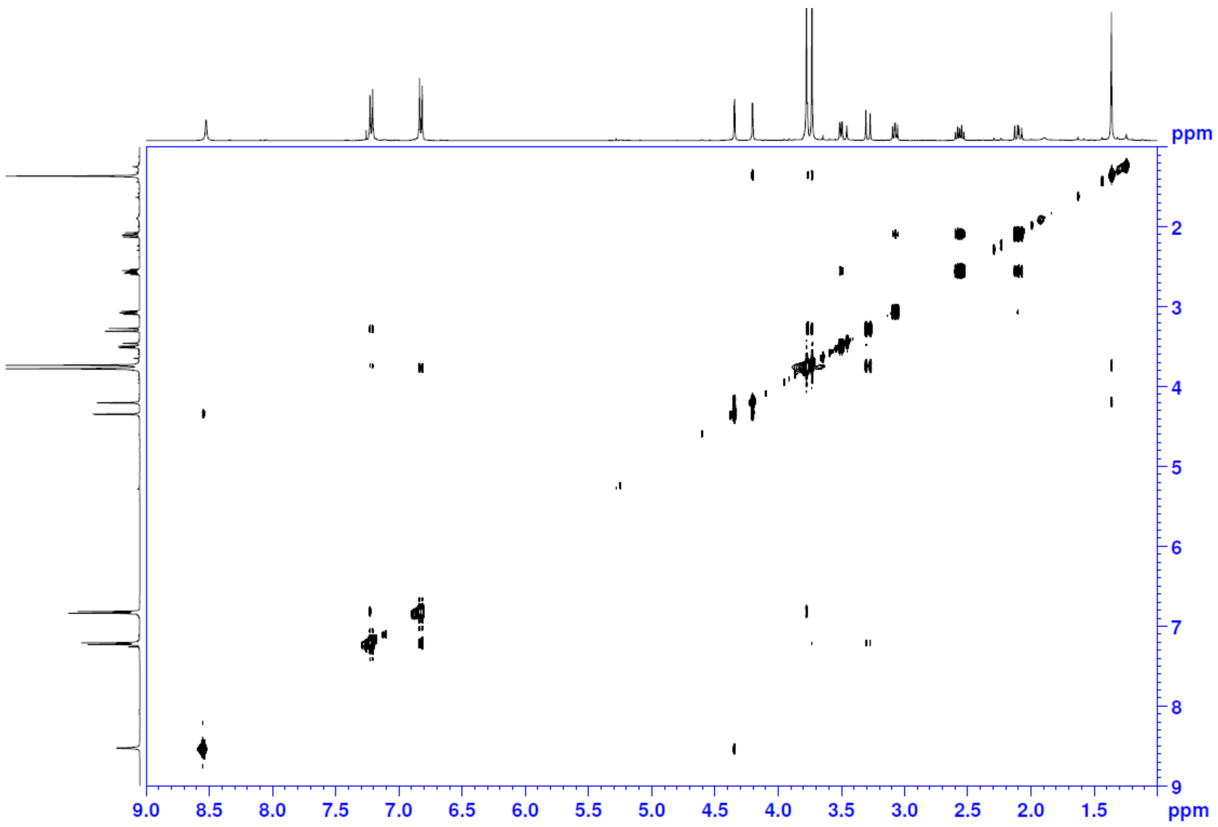

NOESY ^{1}H-^{1}H ($CDCl_3$), δ (ppm) – Aliphatic Zoom

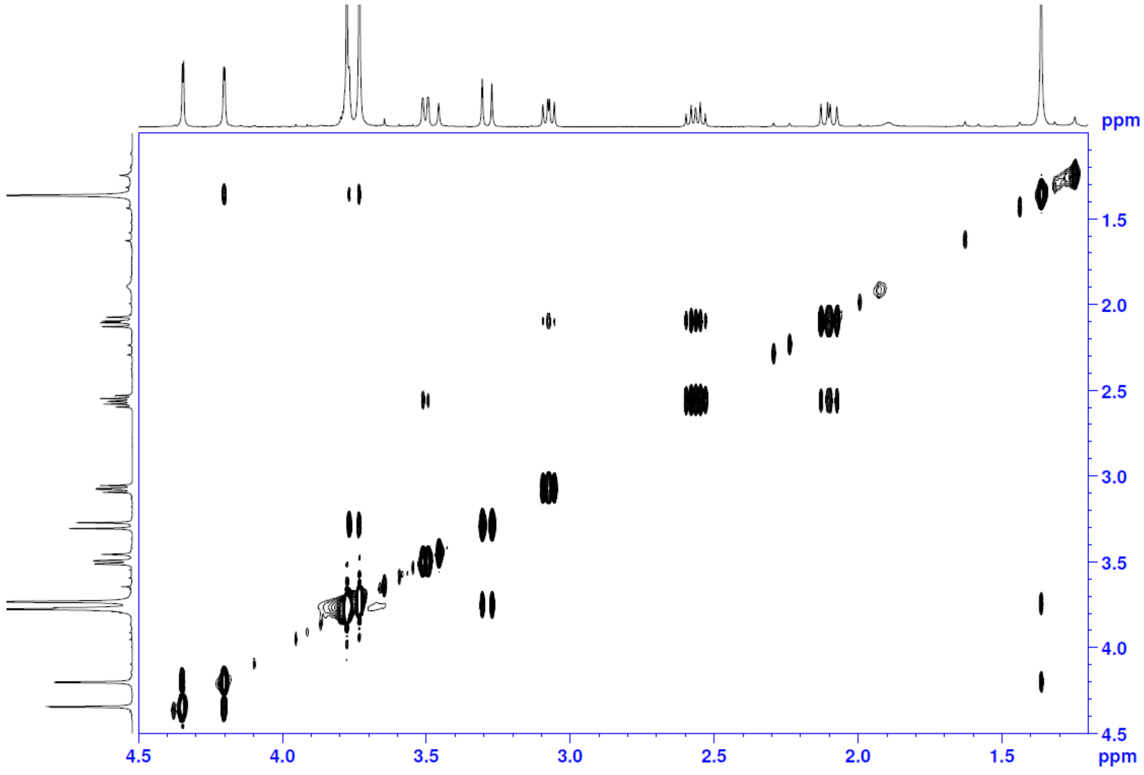

HSQC ^1H-^{13}C ($CDCl_3$), δ (ppm)

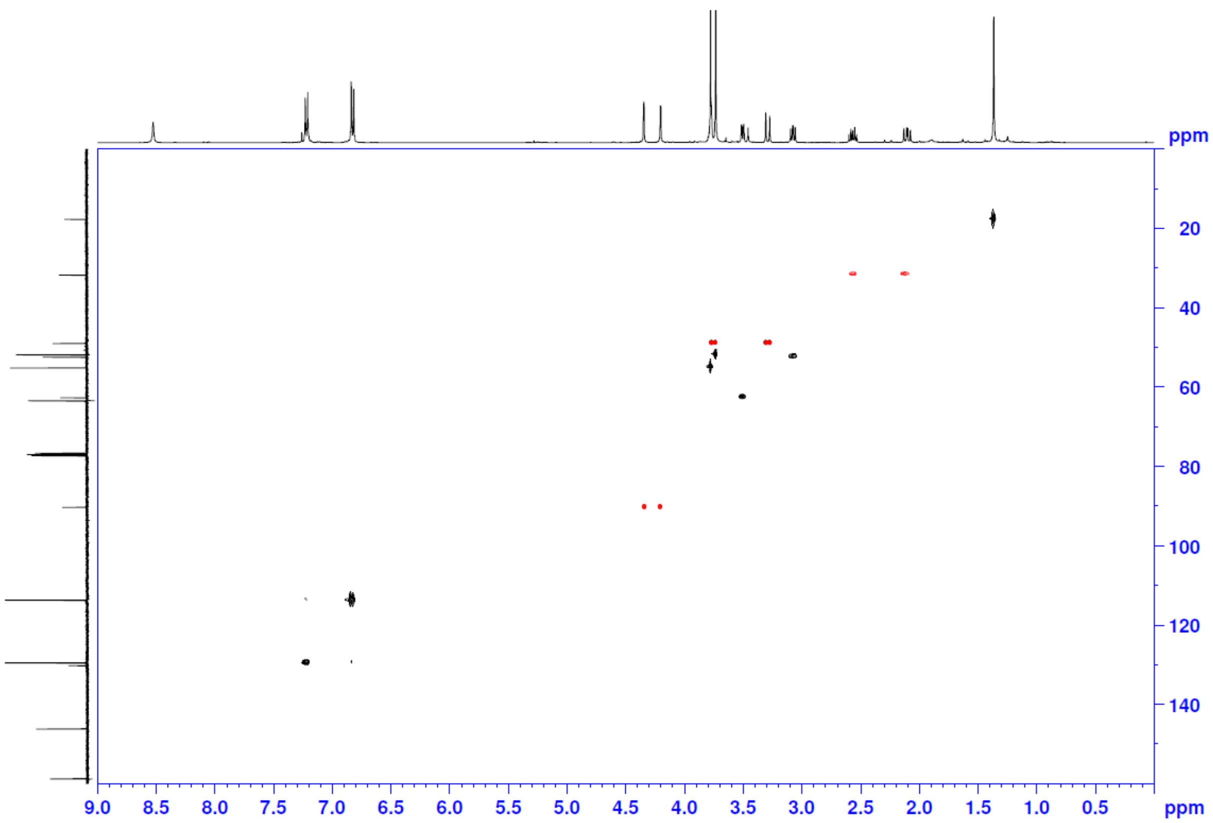

HMBC ^1H-^{13}C ($CDCl_3$), δ (ppm)

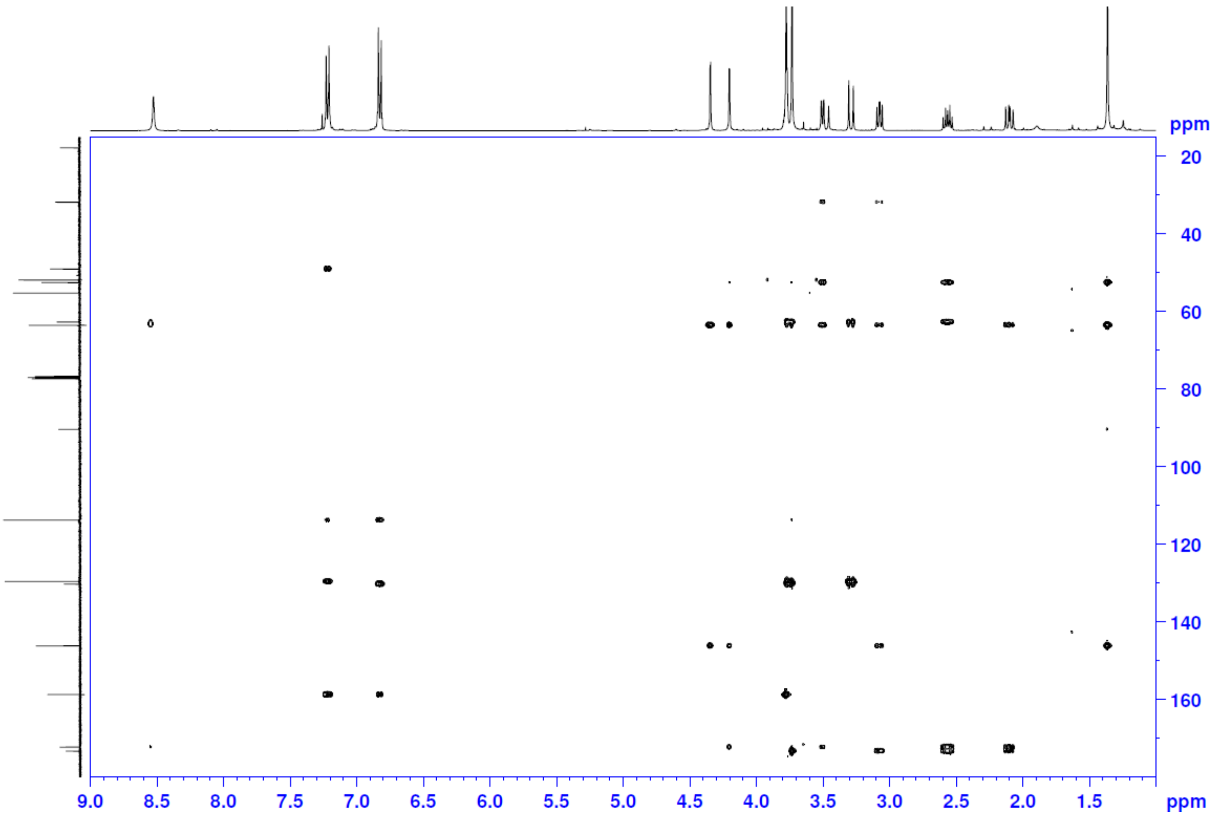

FT-IR (neat), ν (cm^{-1})

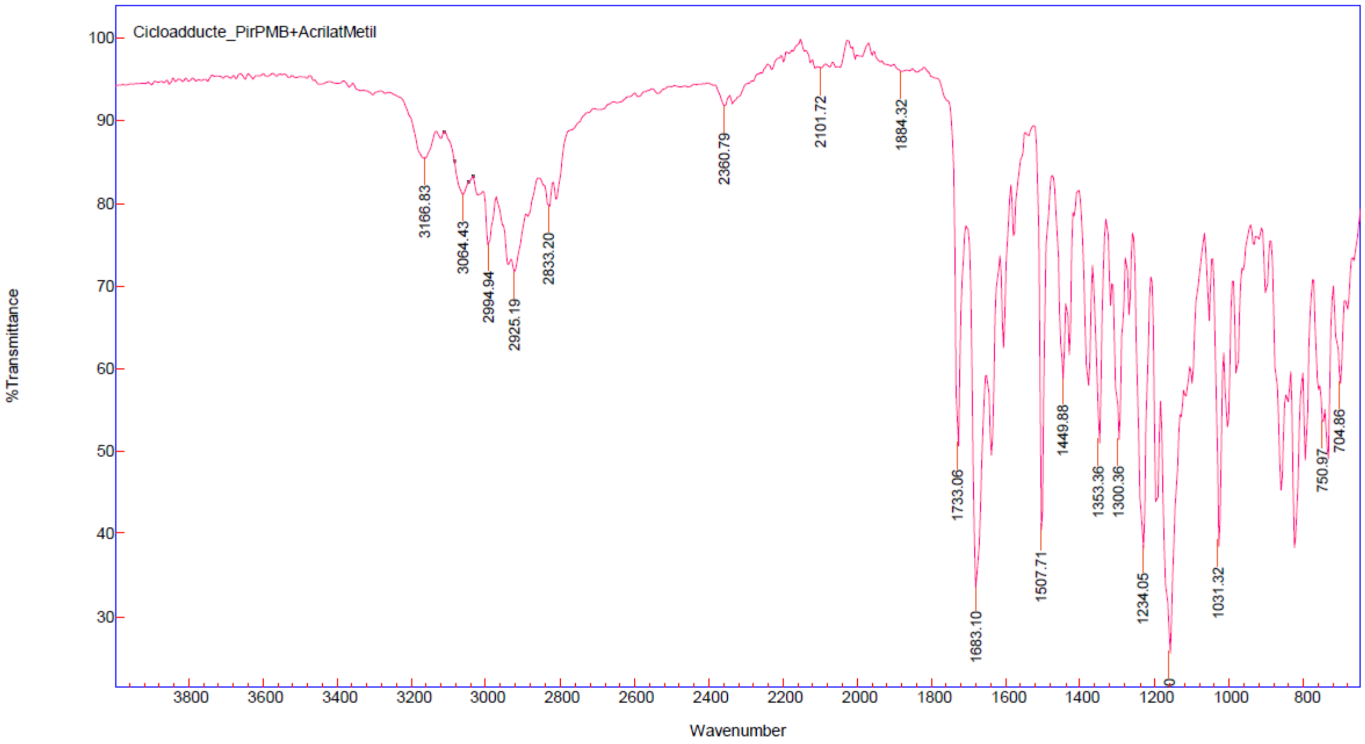

ESI-MS (*m/z*)

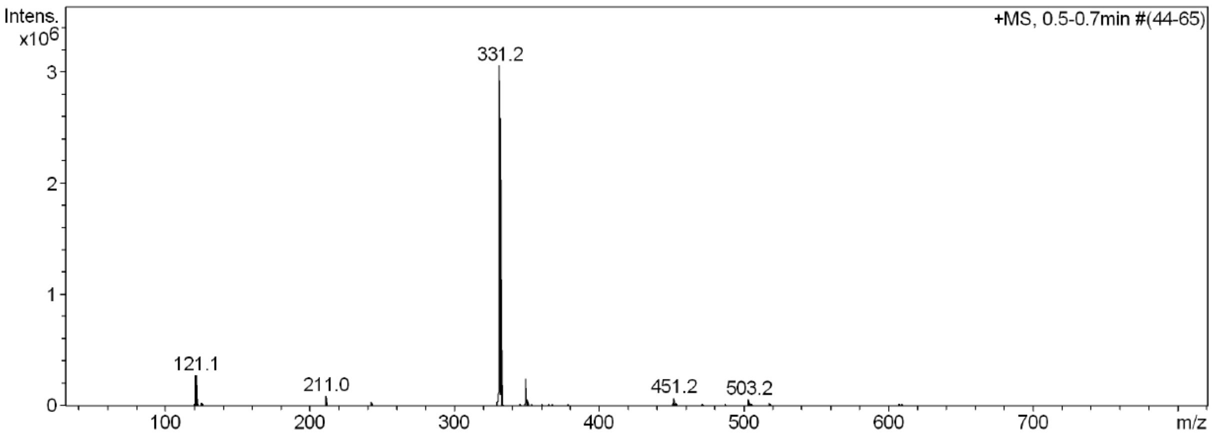

HPLC (λ = 220 nm)

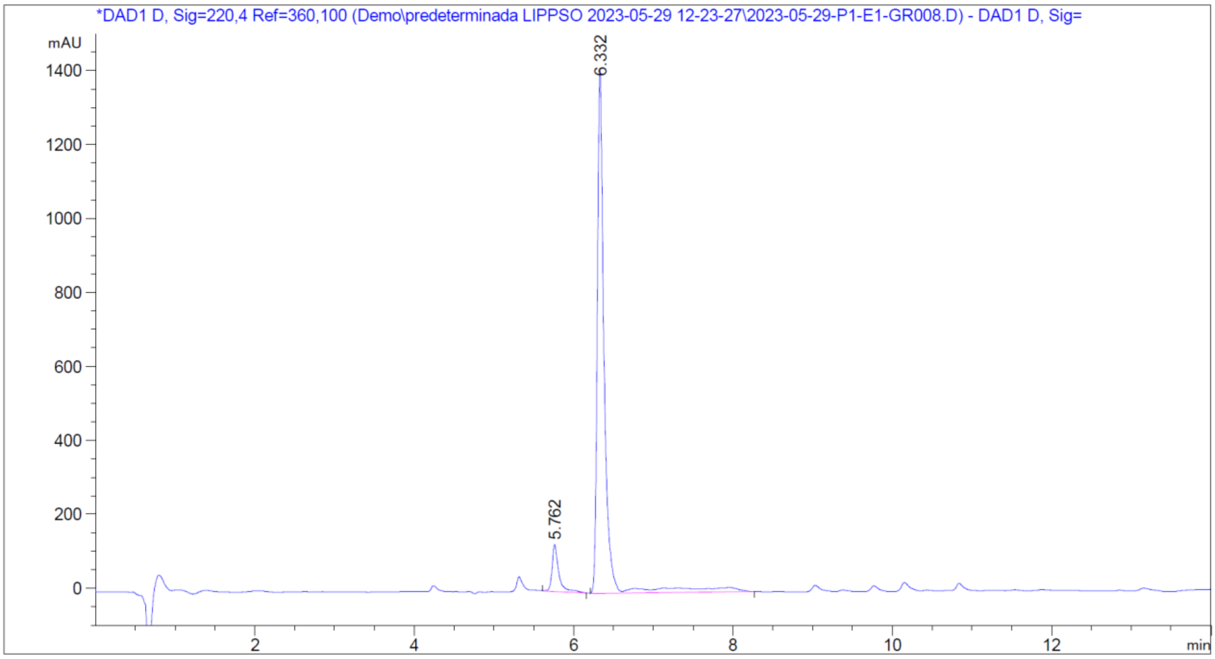

Peak #	RetTime [min]	Type	Width [min]	Area [mAU*s]	Height [mAU]	Area %
1	5.762	BB	0.0851	737.38477	126.31142	6.4693
2	6.332	BV R	0.0913	9004.38574	1414.25037	93.5307

Totals : 9627.19806 1540.56179

HRMS (*m/z*)

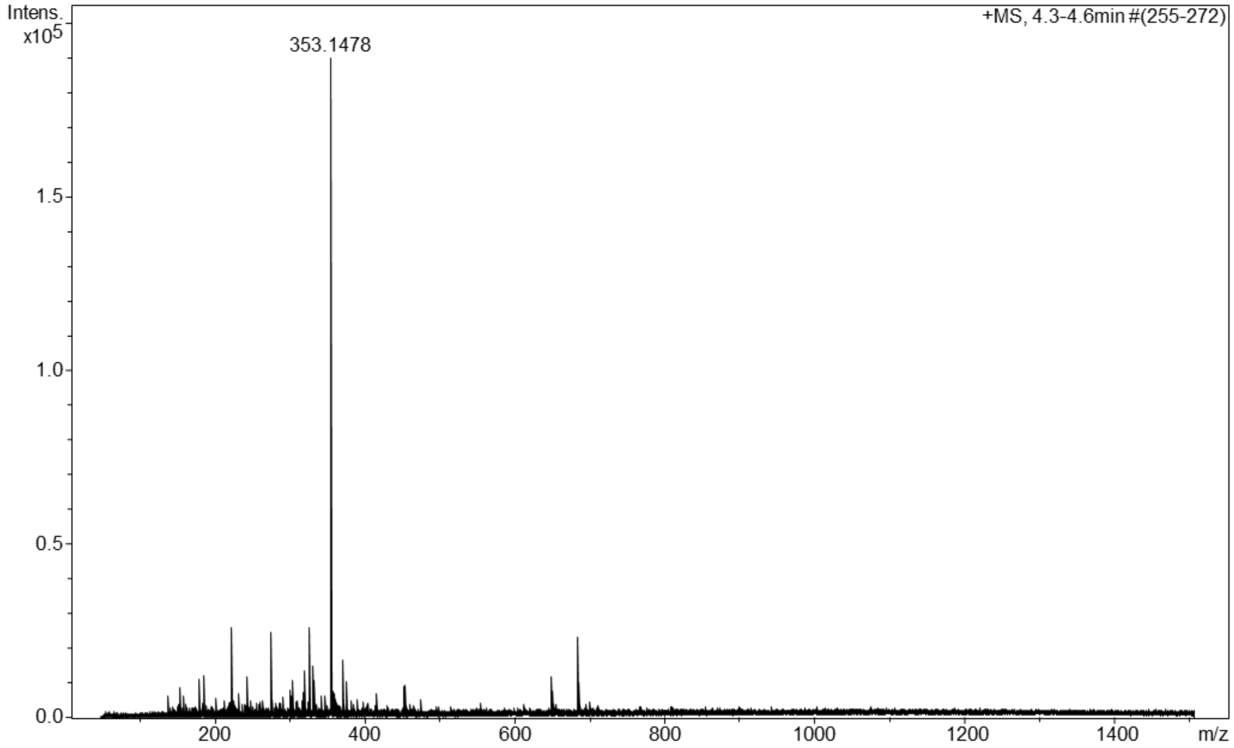

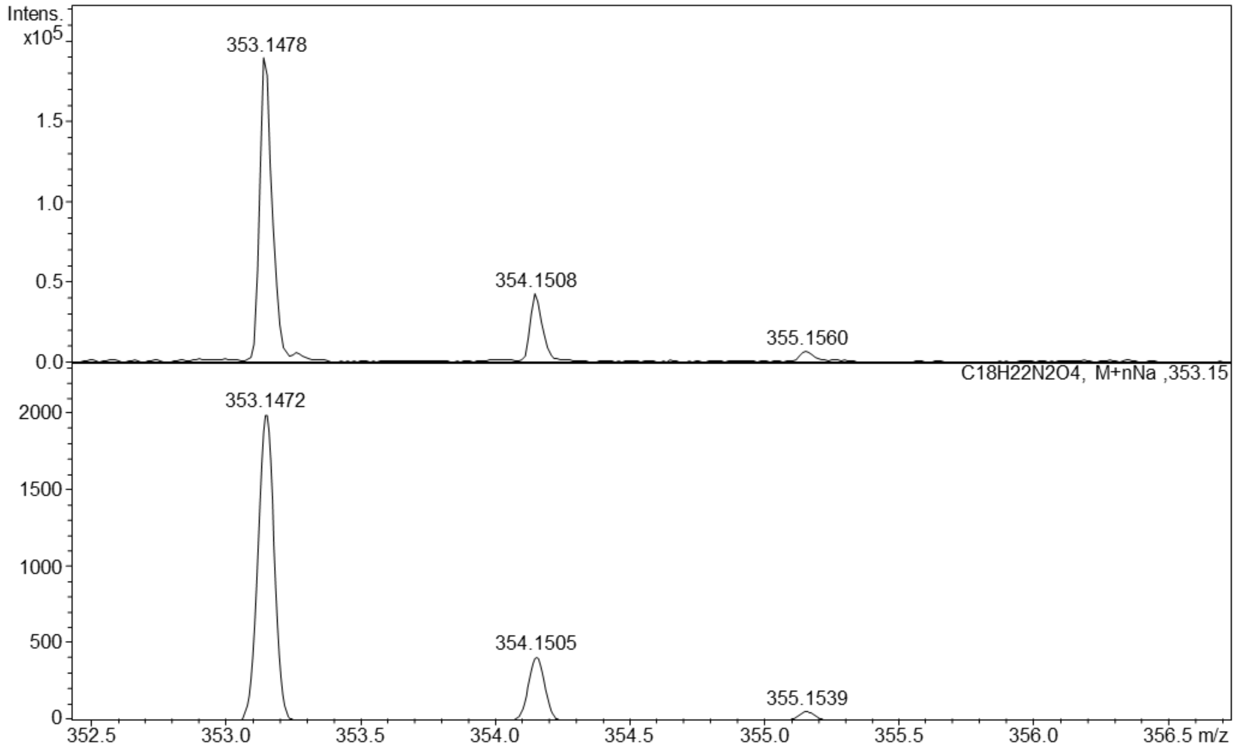

	Molecular formula	Calculated	Found
**[M+Na]+**	$C_{18}H_{22}N_2O_4Na$	353.1472	353.1478

tert-Butyl 8-(4-methoxybenzyl)-5-methyl-4-methylene-2-oxo-3,8-diazabicyclo[3.2.1]octane-6-carboxylate (20b)

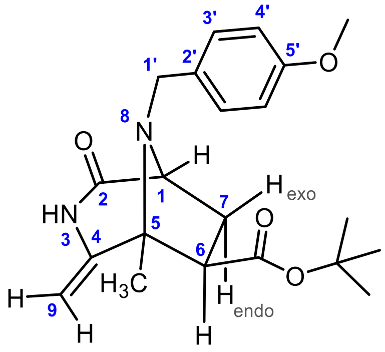

20b

^{1}H-NMR (400 MHz, CDCl3), _δ_ (ppm)

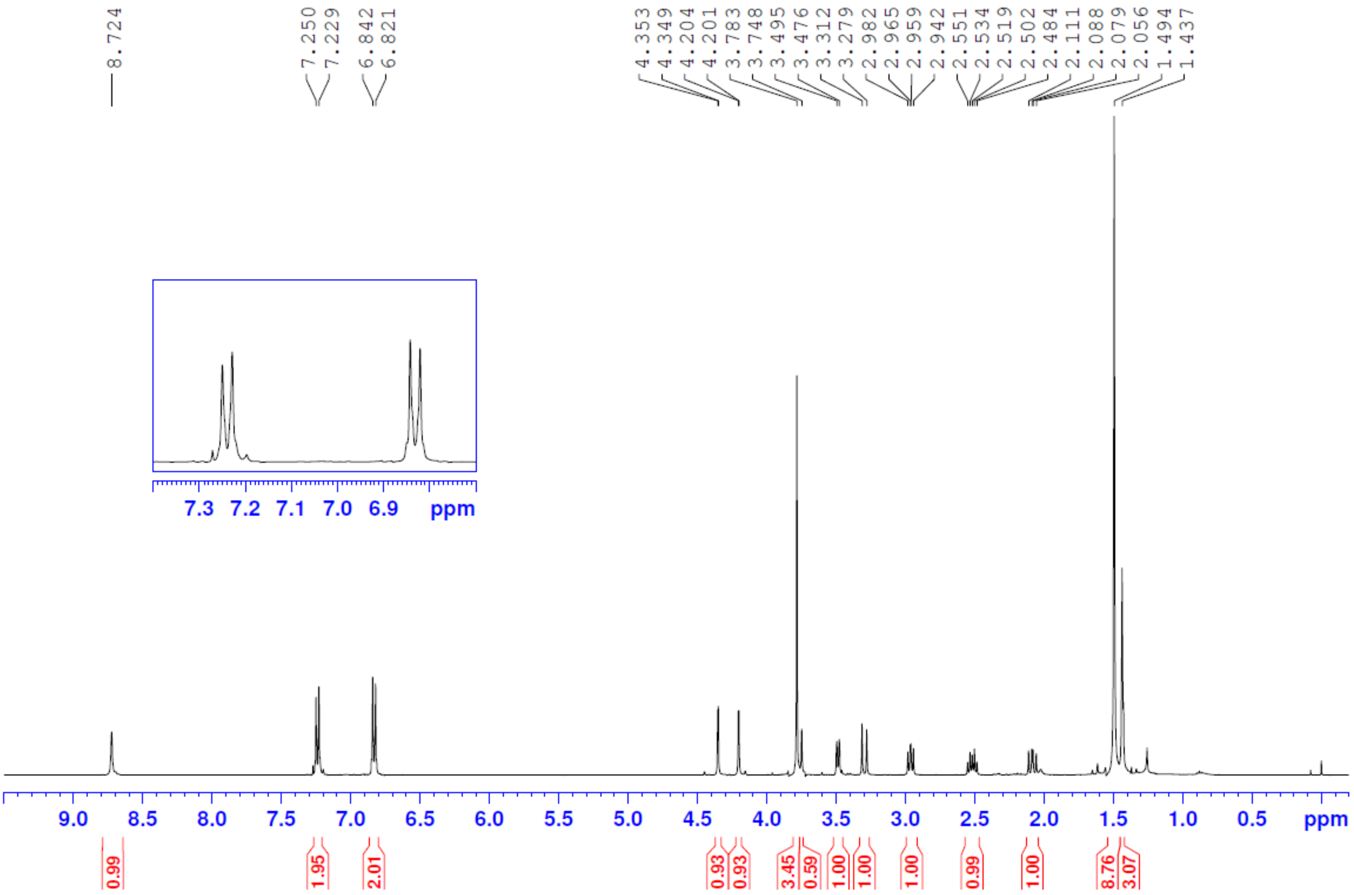

^{1}H-NMR (400 MHz, CDCl3), δ (ppm) – Aliphatic zoom

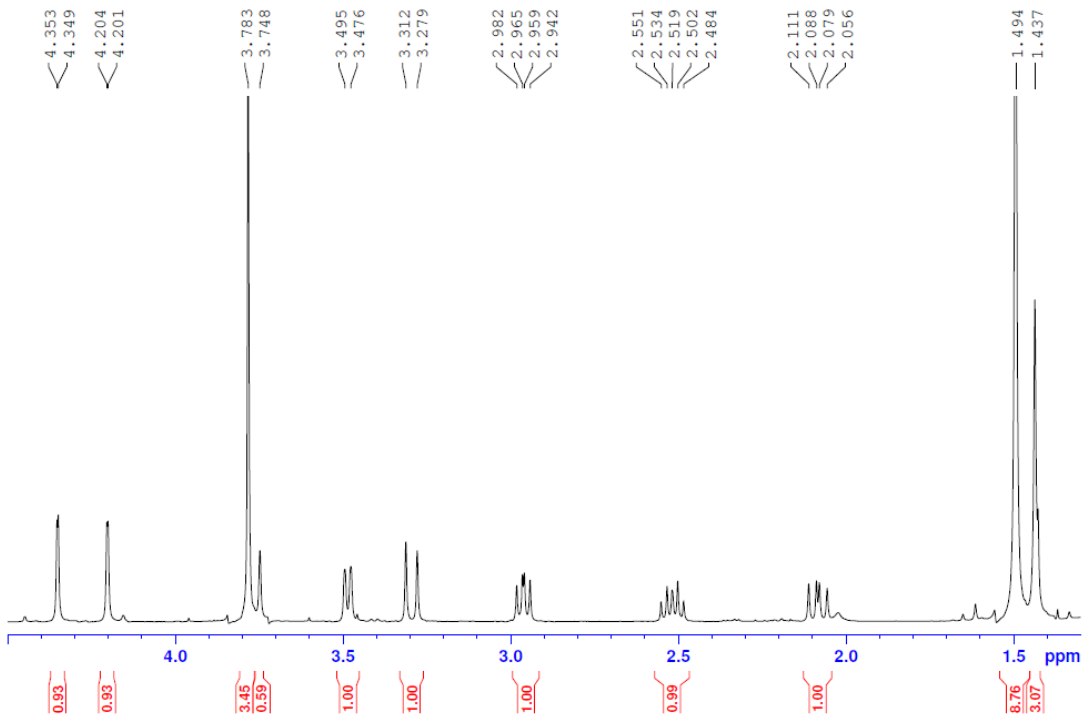

^{13}C{^{1}H}-NMR (100 MHz, CDCl3), δ (ppm)

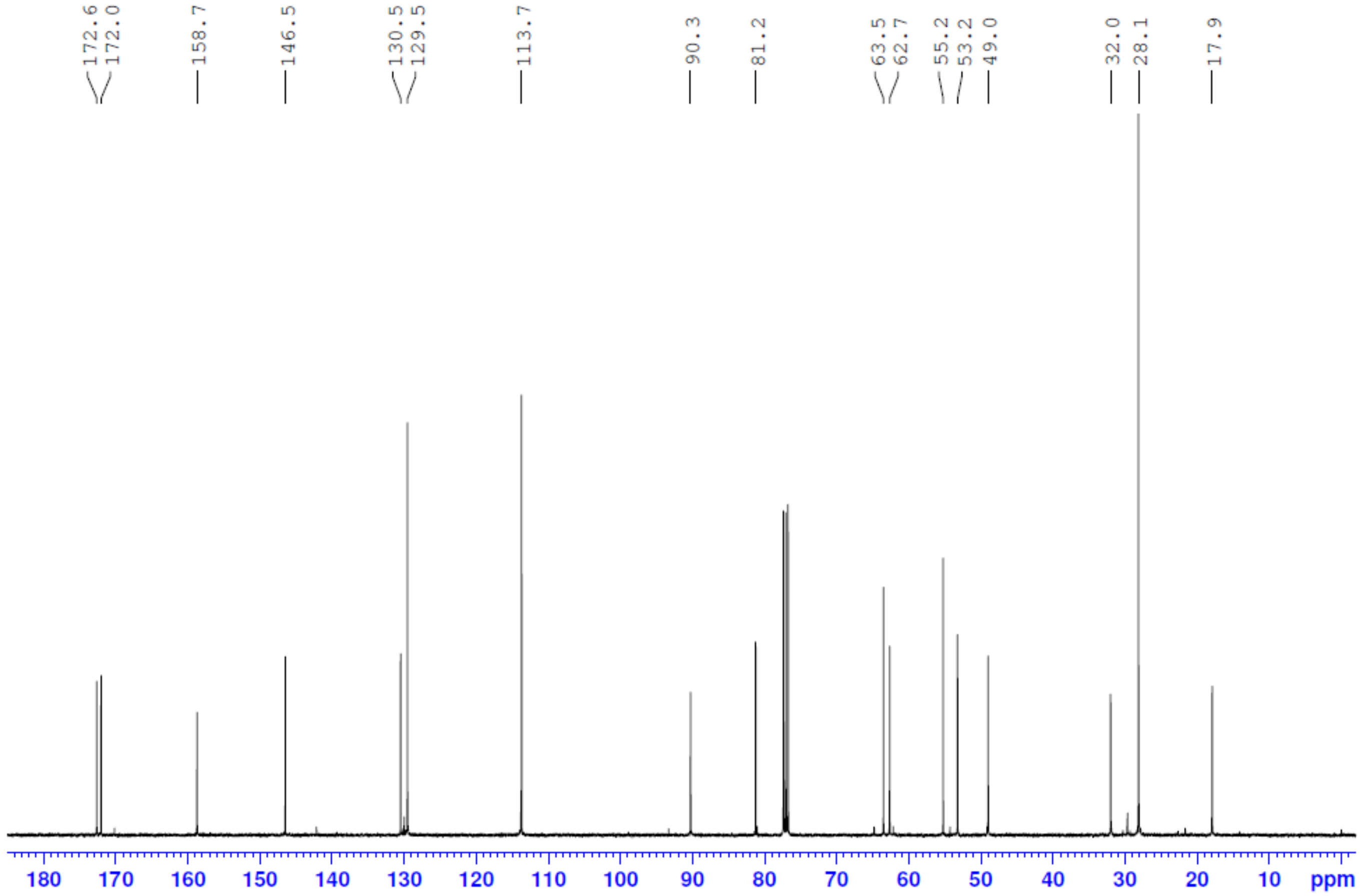

COSY ^{1}H-^{1}H ($CDCl_3$), δ (ppm)

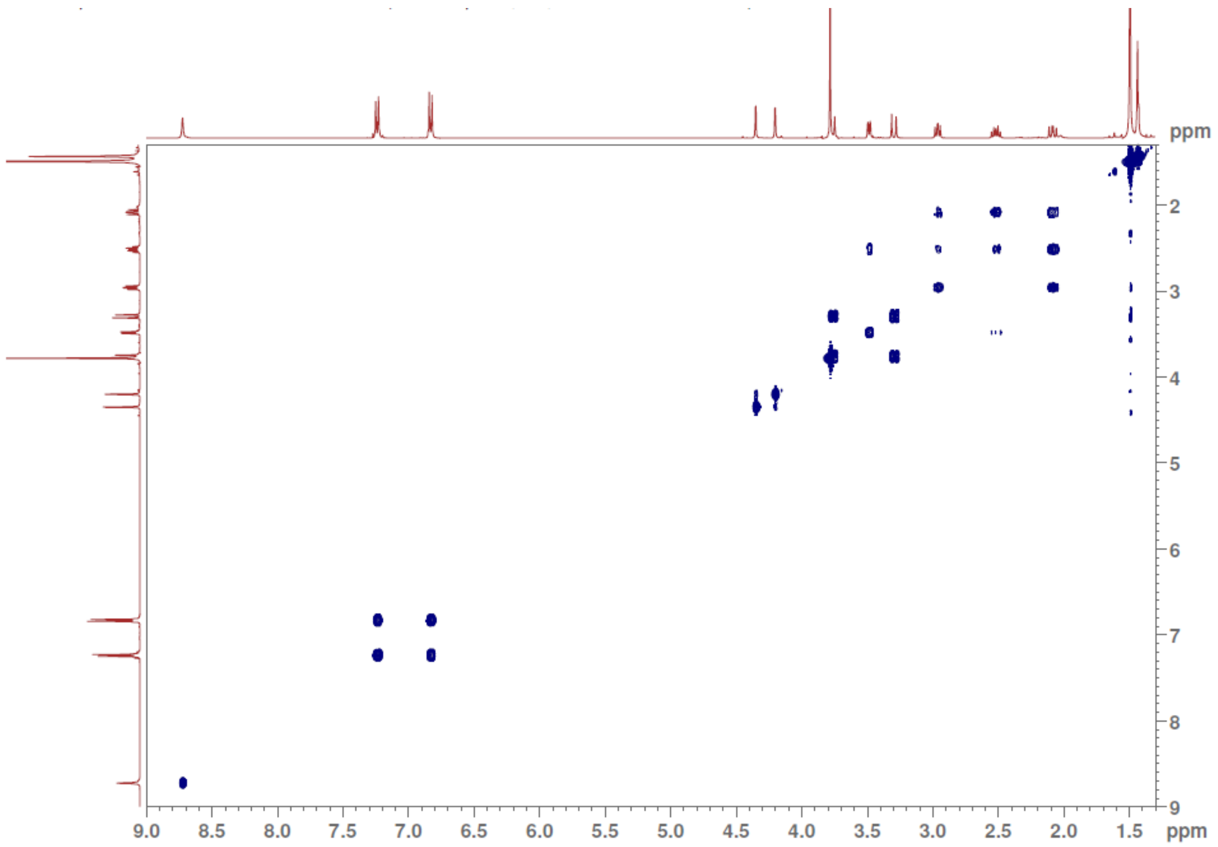

COSY ^{1}H-^{1}H ($CDCl_3$), δ (ppm) – Aliphatic Zoom

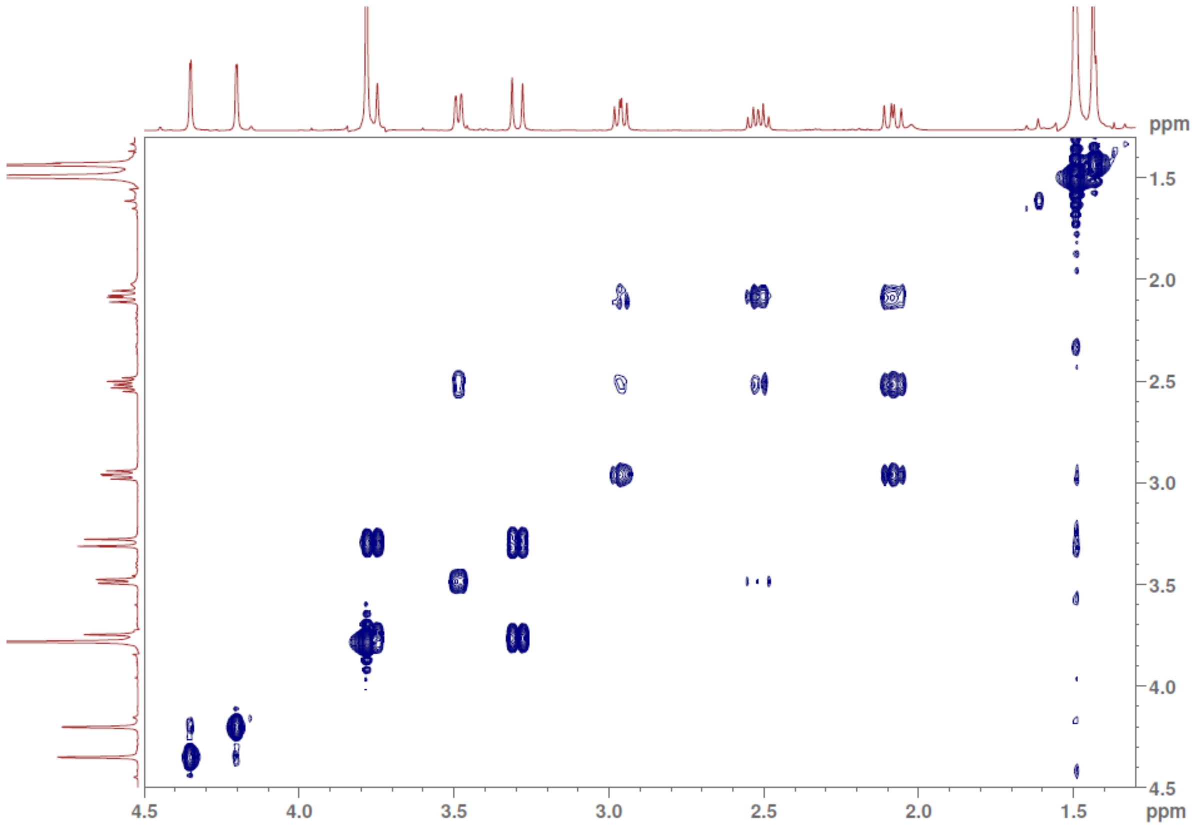

NOESY ¹H-¹H (CDCl₃), *δ* (ppm)

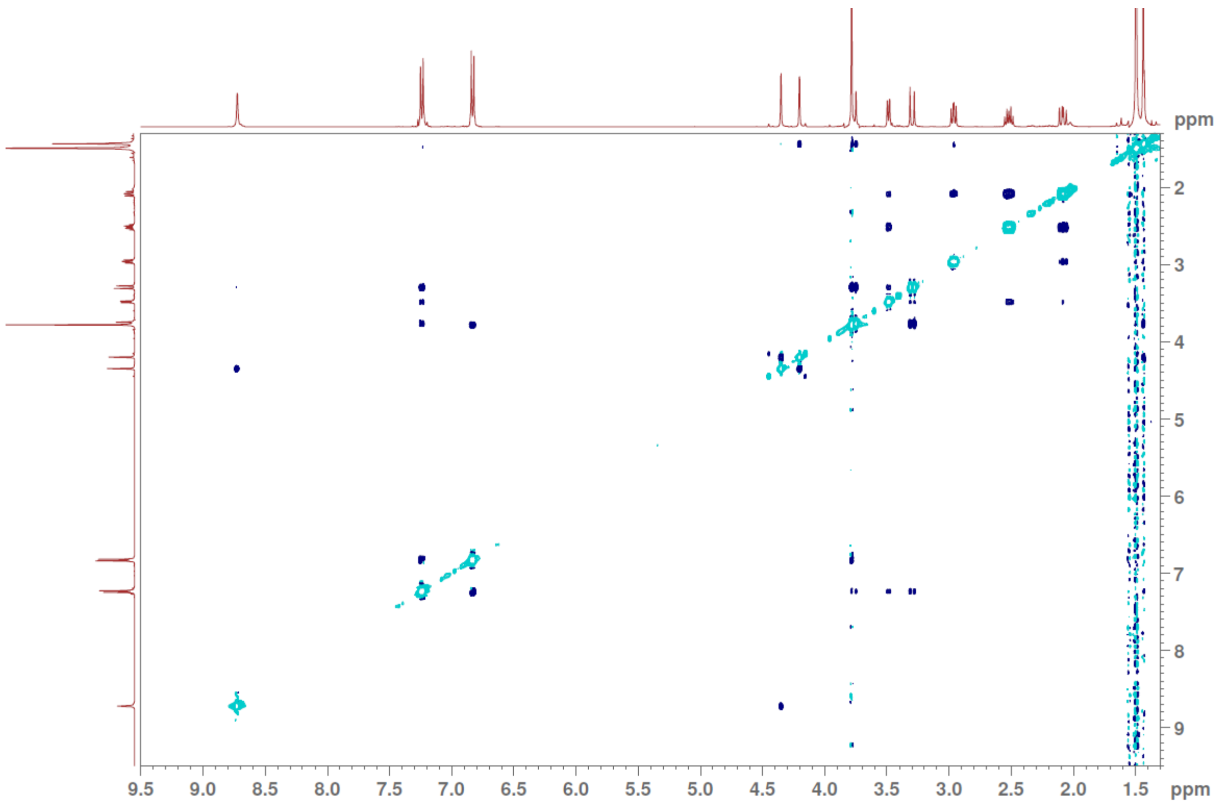

NOESY ¹H-¹H (CDCl₃), *δ* (ppm) – Aliphatic Zoom

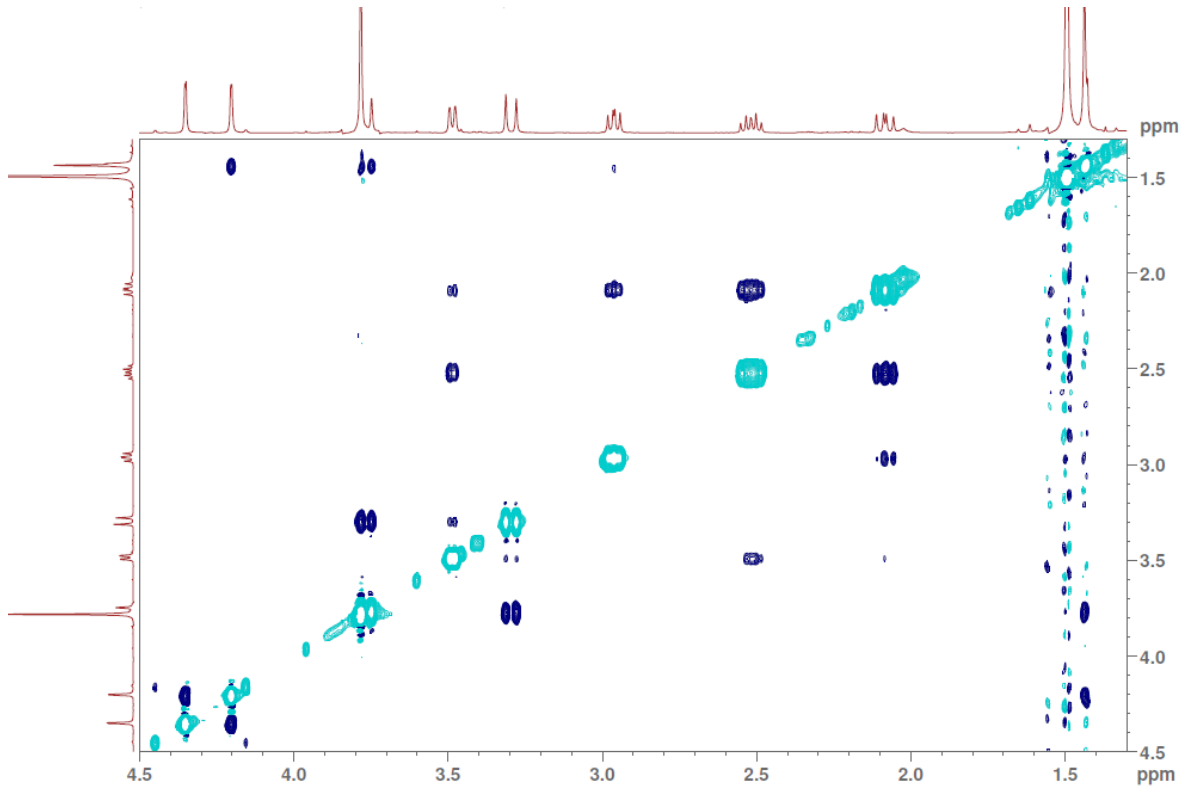

HSQC ^{1}H-^{13}C ($CDCl_3$), δ (ppm)

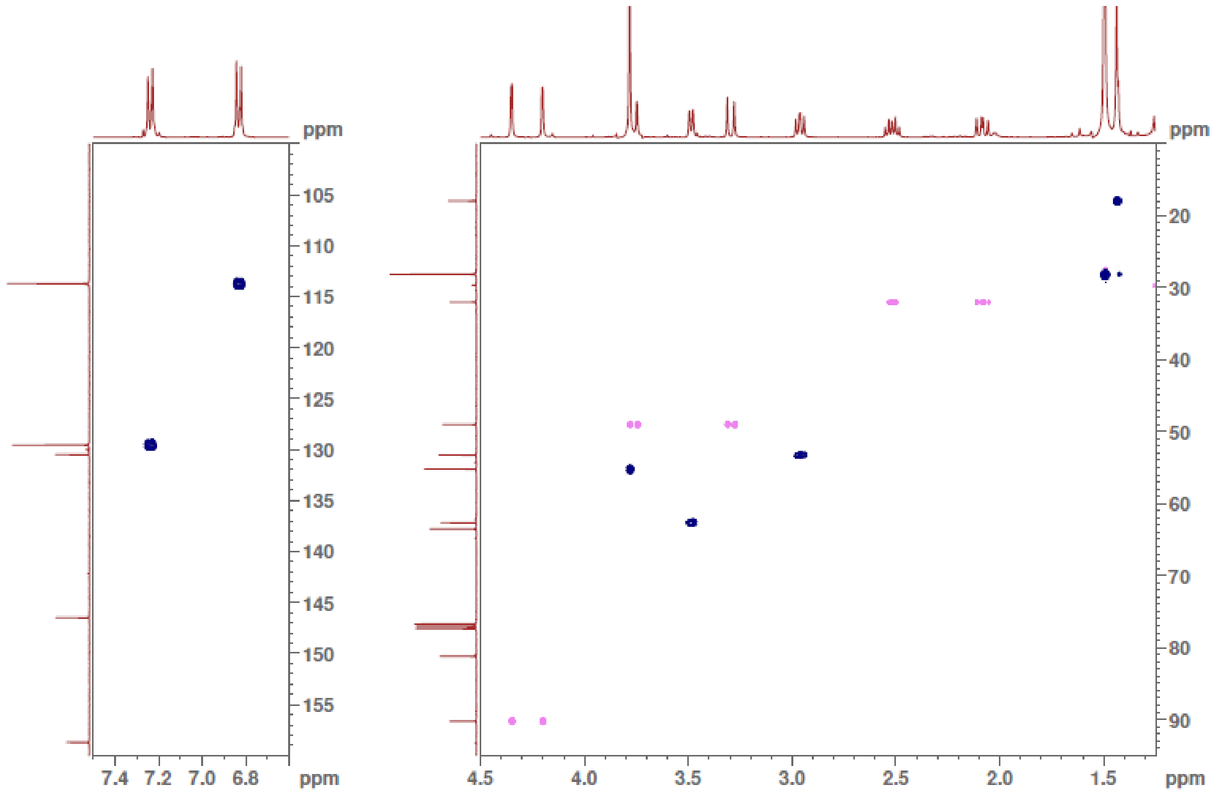

HMBC ^{1}H-^{13}C ($CDCl_3$), δ (ppm)

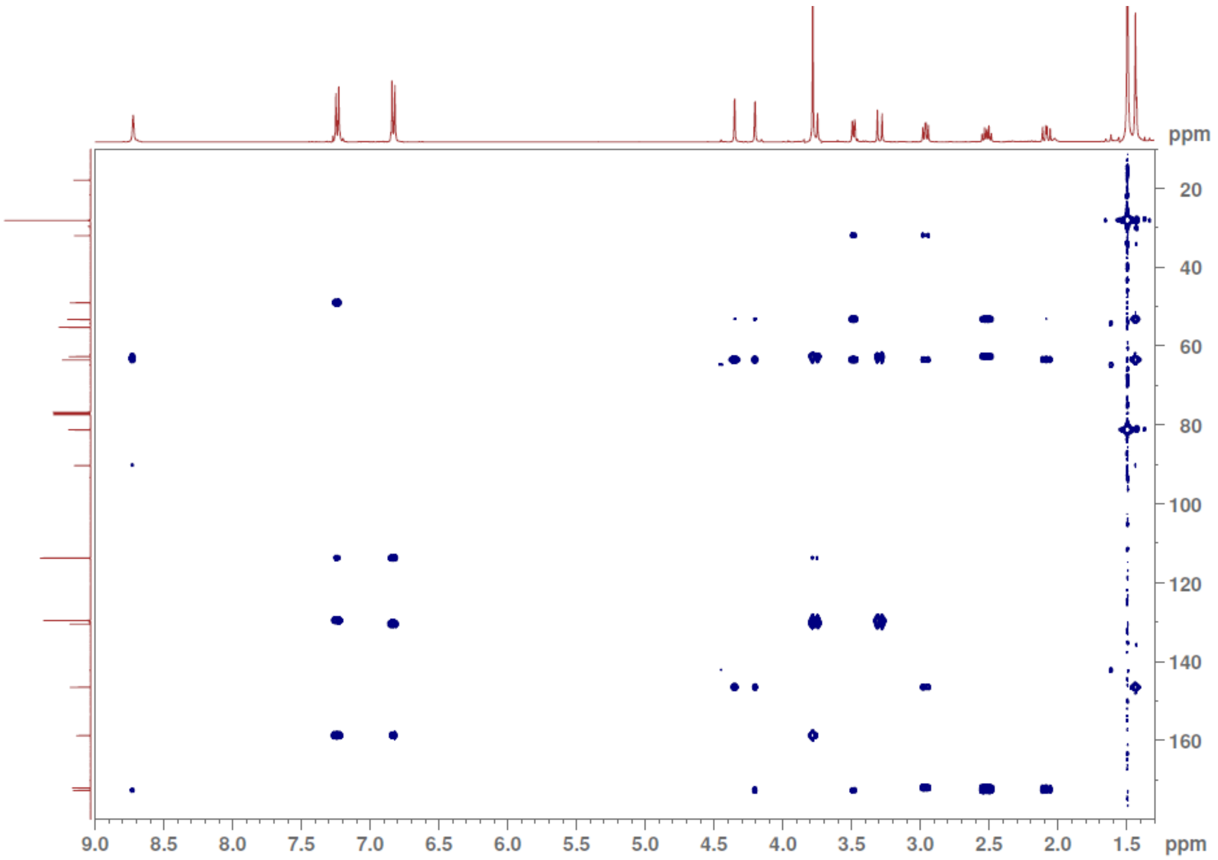

FT-IR (neat), ν (cm^{-1})

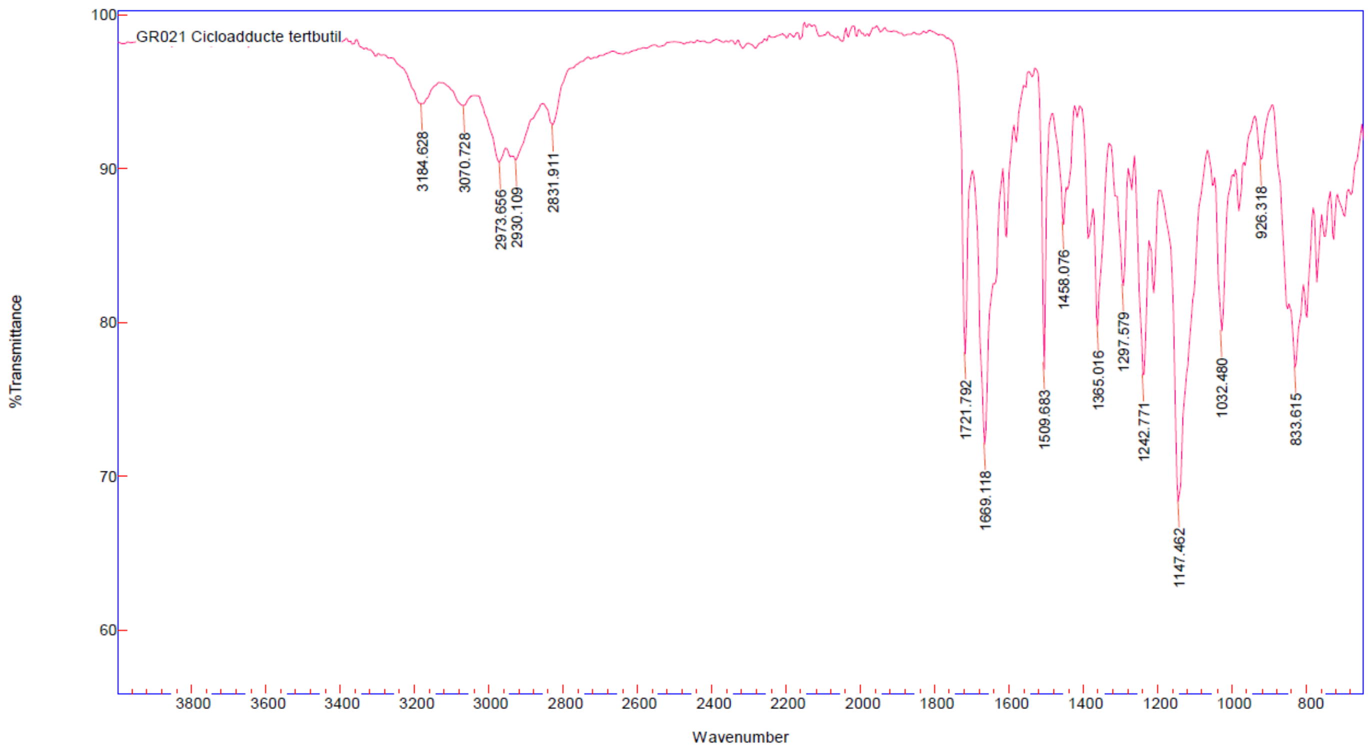

HPLC (λ = 220 nm)

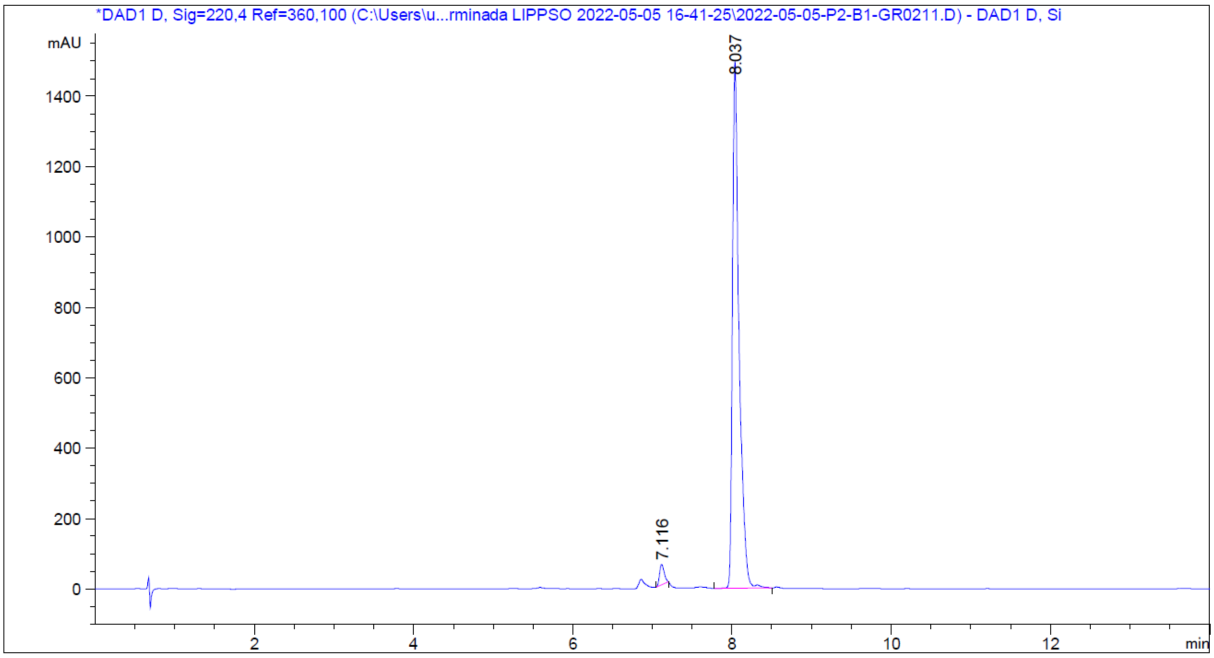

Peak #	RetTime [min]	Type	Width [min]	Area [mAU*s]	Height [mAU]	Area %
1	7.116	BBA	0.0628	236.60632	57.69373	2.6651
2	8.037	BV R	0.0842	8641.20313	1499.35510	97.3349
Totals :				8877.80945	1557.04883	

ESI-MS (*m/z*)

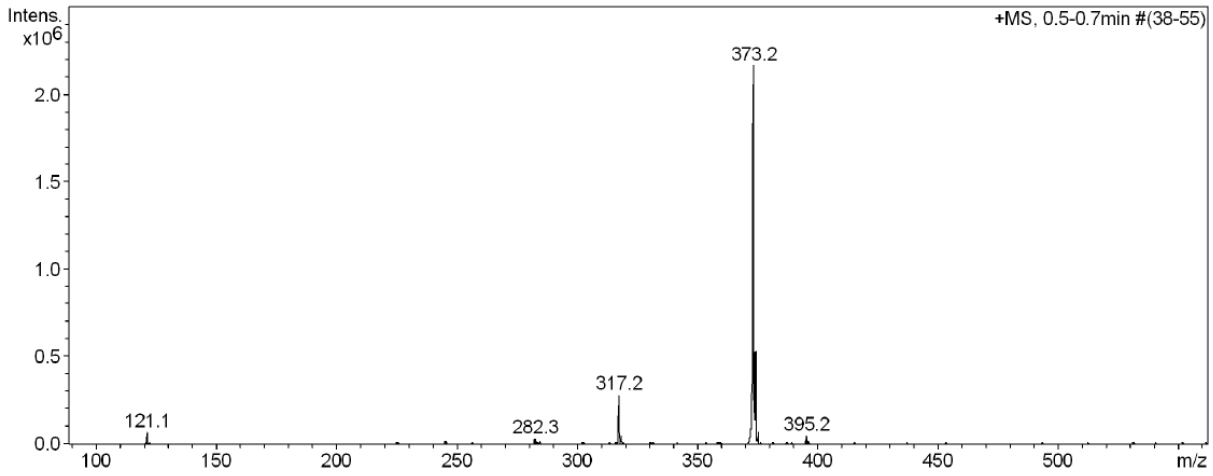

ESI-MS/MS (*m/z* = 373.2)

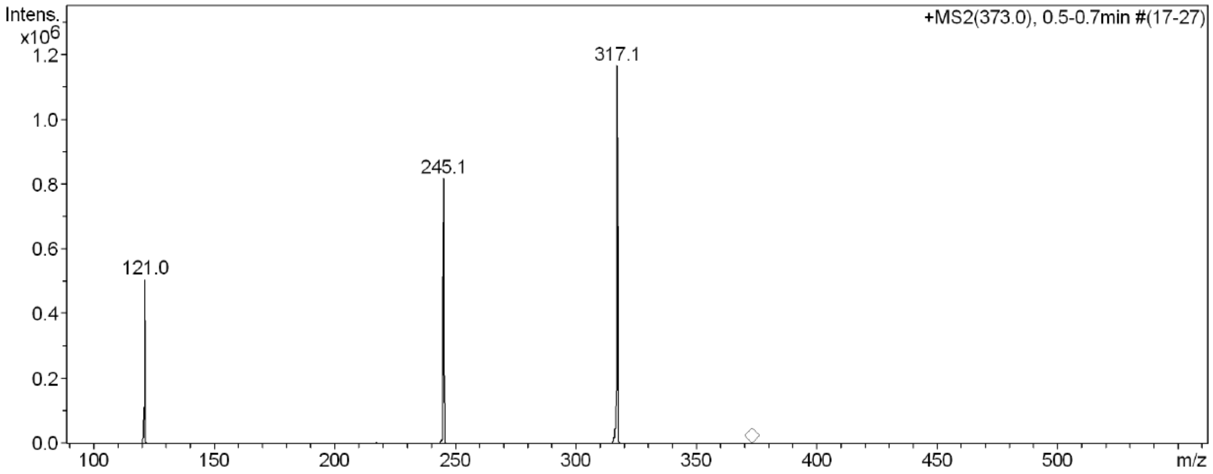

HRMS (*m/z*)

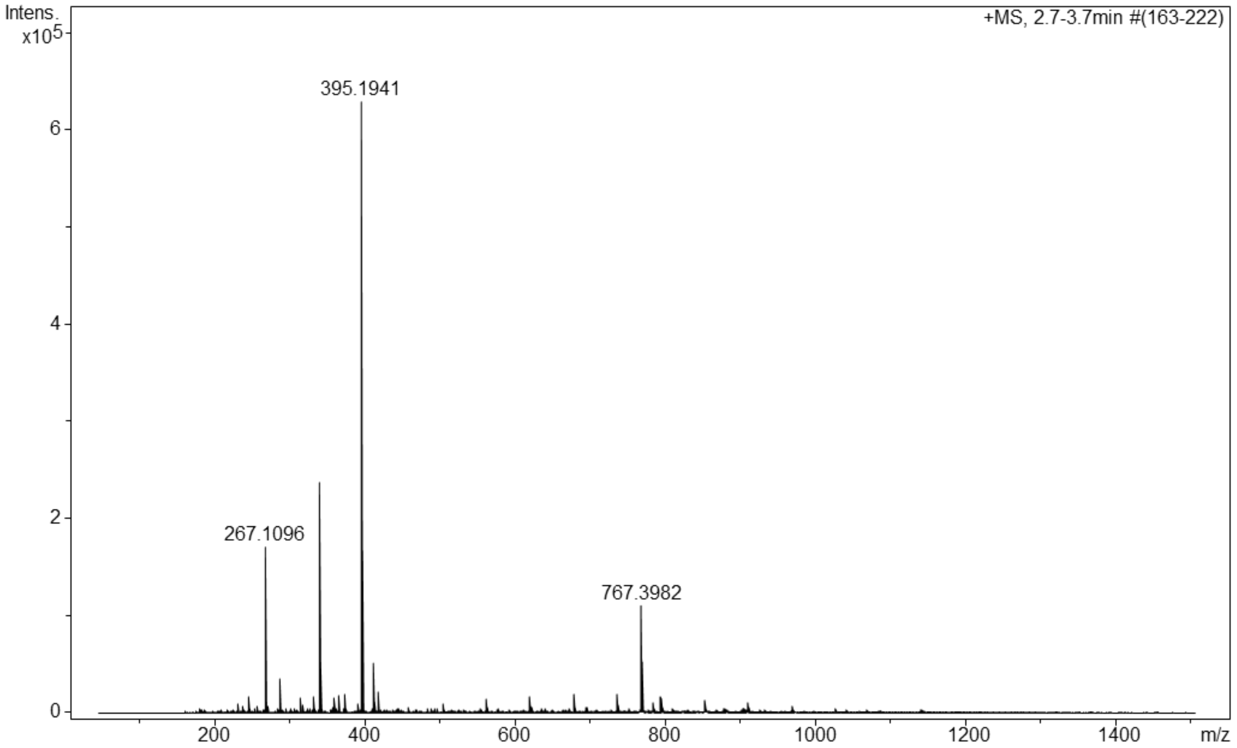

Intens.
x10^5
+MS, 2.7-3.7min #(163-222)
395.1941
267.1096
767.3982
m/z

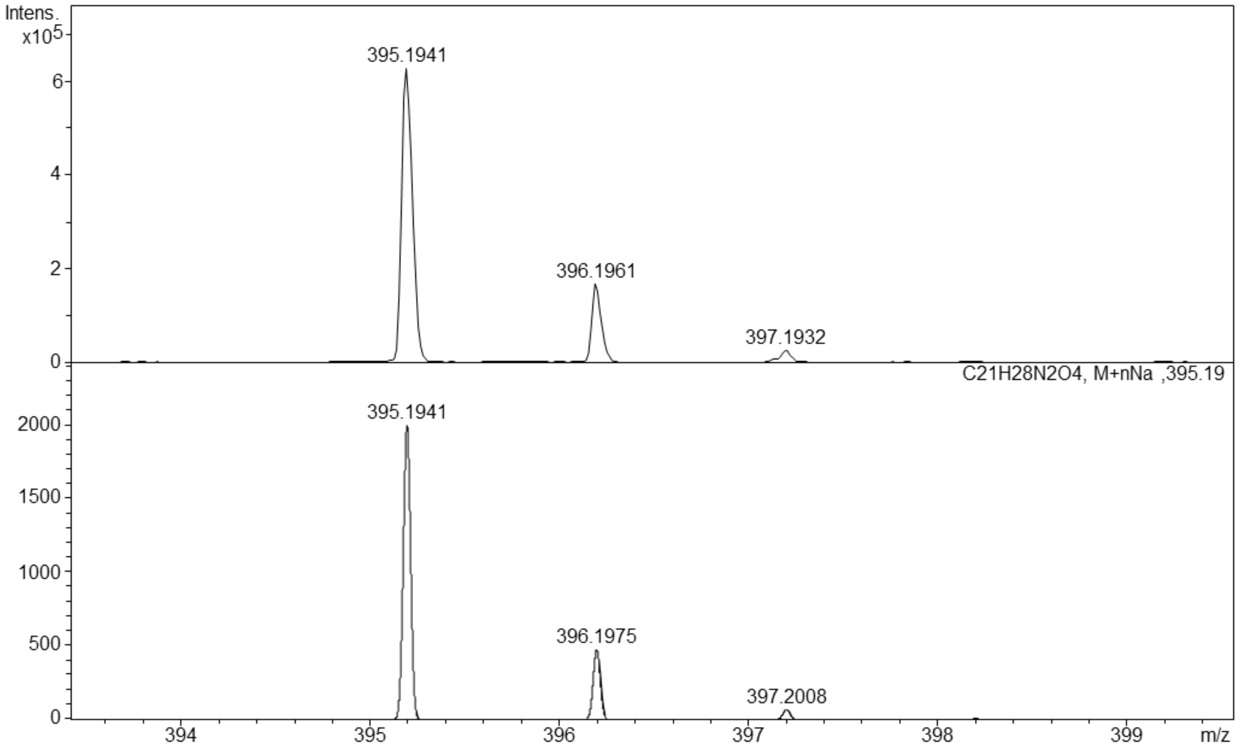

Intens.
x10^5
395.1941
396.1961
397.1932
C21H28N2O4, M+nNa ,395.19
395.1941
396.1975
397.2008
m/z

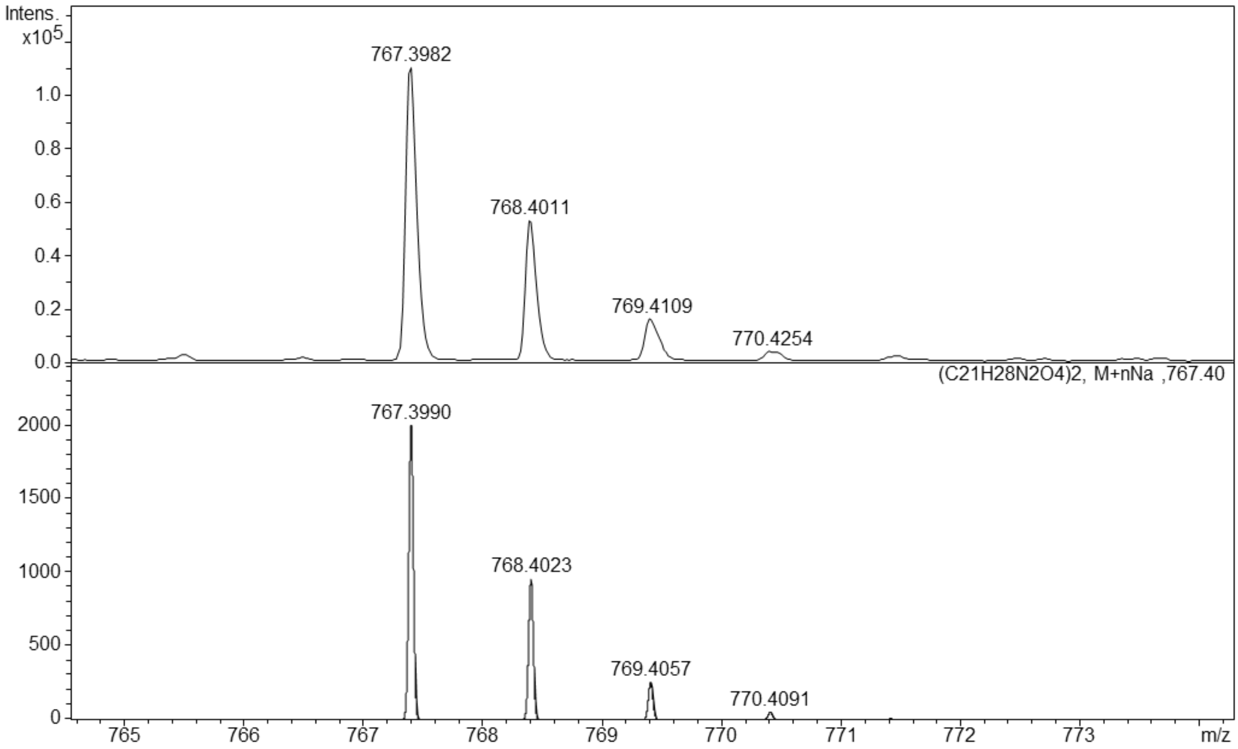

	Molecular formula	Calculated	Found
**[M+Na]+**	$C_{21}H_{28}N_2O_4$	395.1941	395.1941
**[2M+Na]+**	$(C_{21}H_{28}N_2O_4)_2Na$	767.3990	767.3982

***tert*-Butyl 5-(4-methoxybenzyl)-1-methyl-6-methylene-3-oxo-2,5-diazabicyclo[2.2.2]octane-7-carboxylate (21b)**

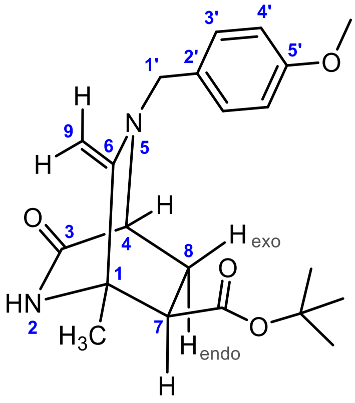

21b

^{1}H-NMR (400 MHz, CDCl3), *δ* (ppm)

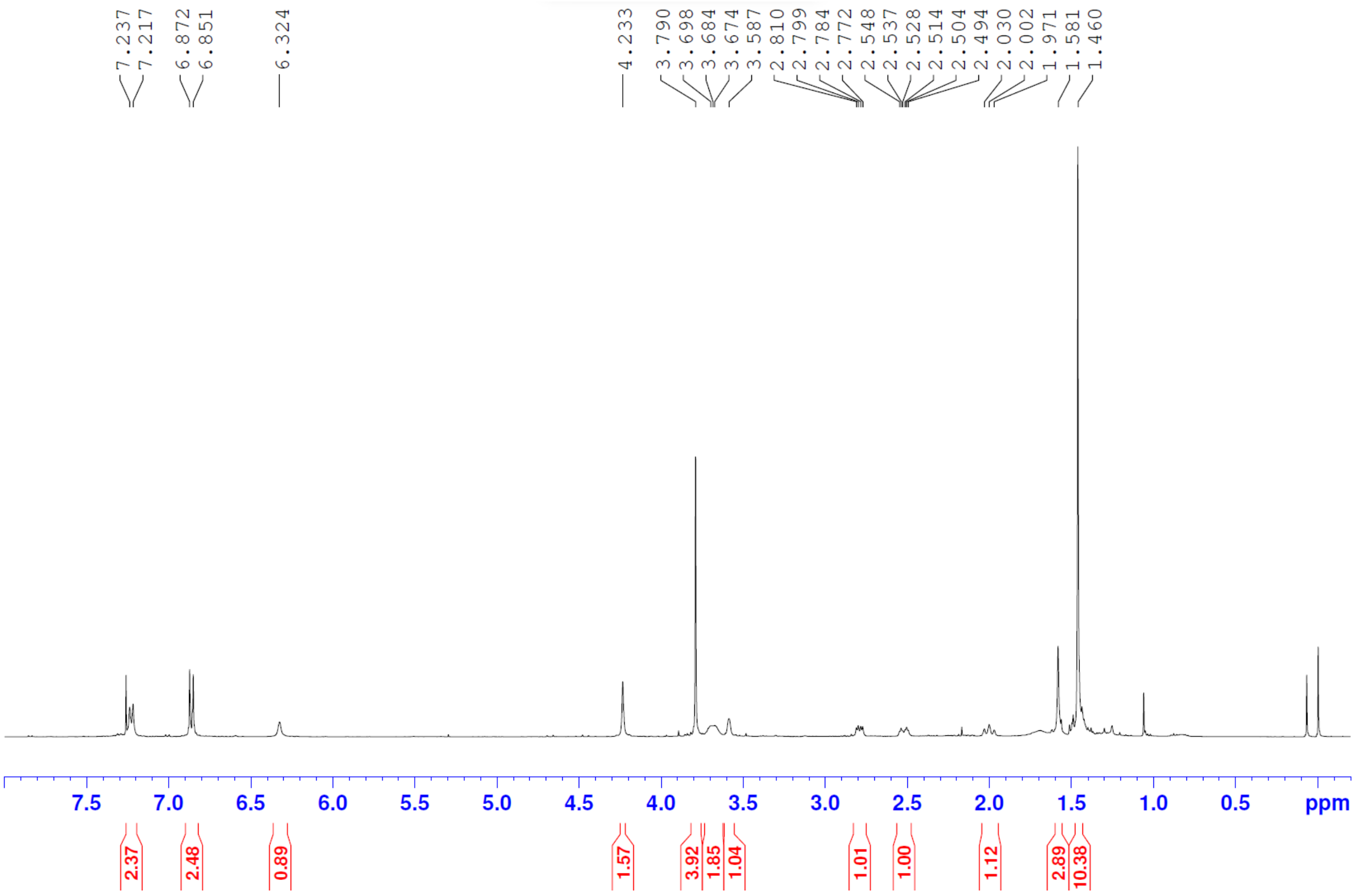

^{1}H-NMR (400 MHz, $CDCl_3$), δ (ppm) – Aliphatic zoom

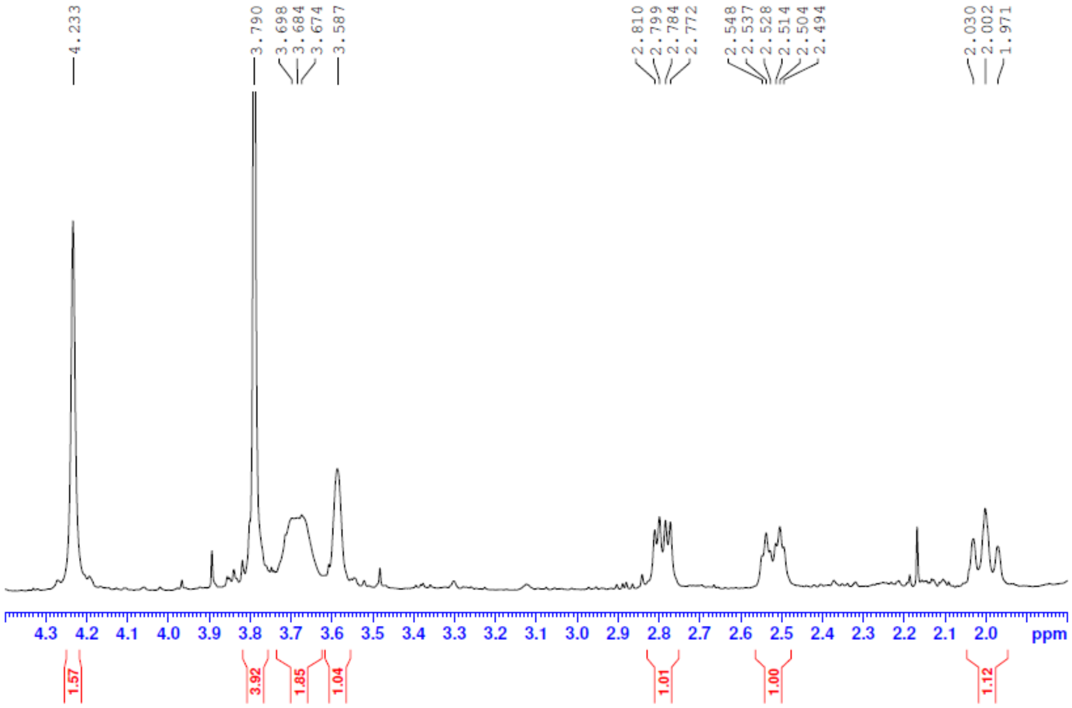

ESI-MS (*m/z*)

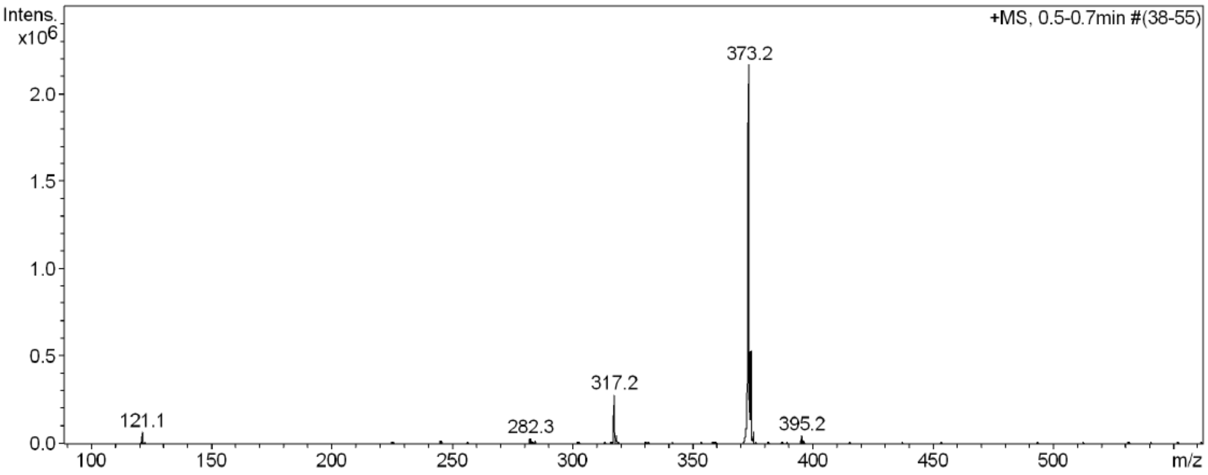

Methyl 8-(4-methoxybenzyl)-5,7-dimethyl-4-methylene-2-oxo-3,8-diazabicyclo[3.2.1]octane-6-carboxylate (20c)

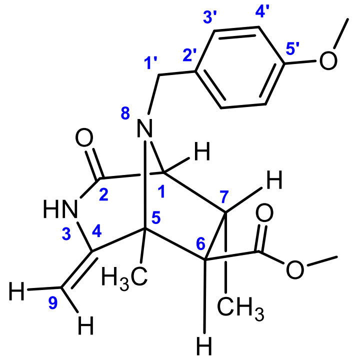

20c

^{1}H-NMR (400 MHz, CDCl$_3$), δ (ppm)

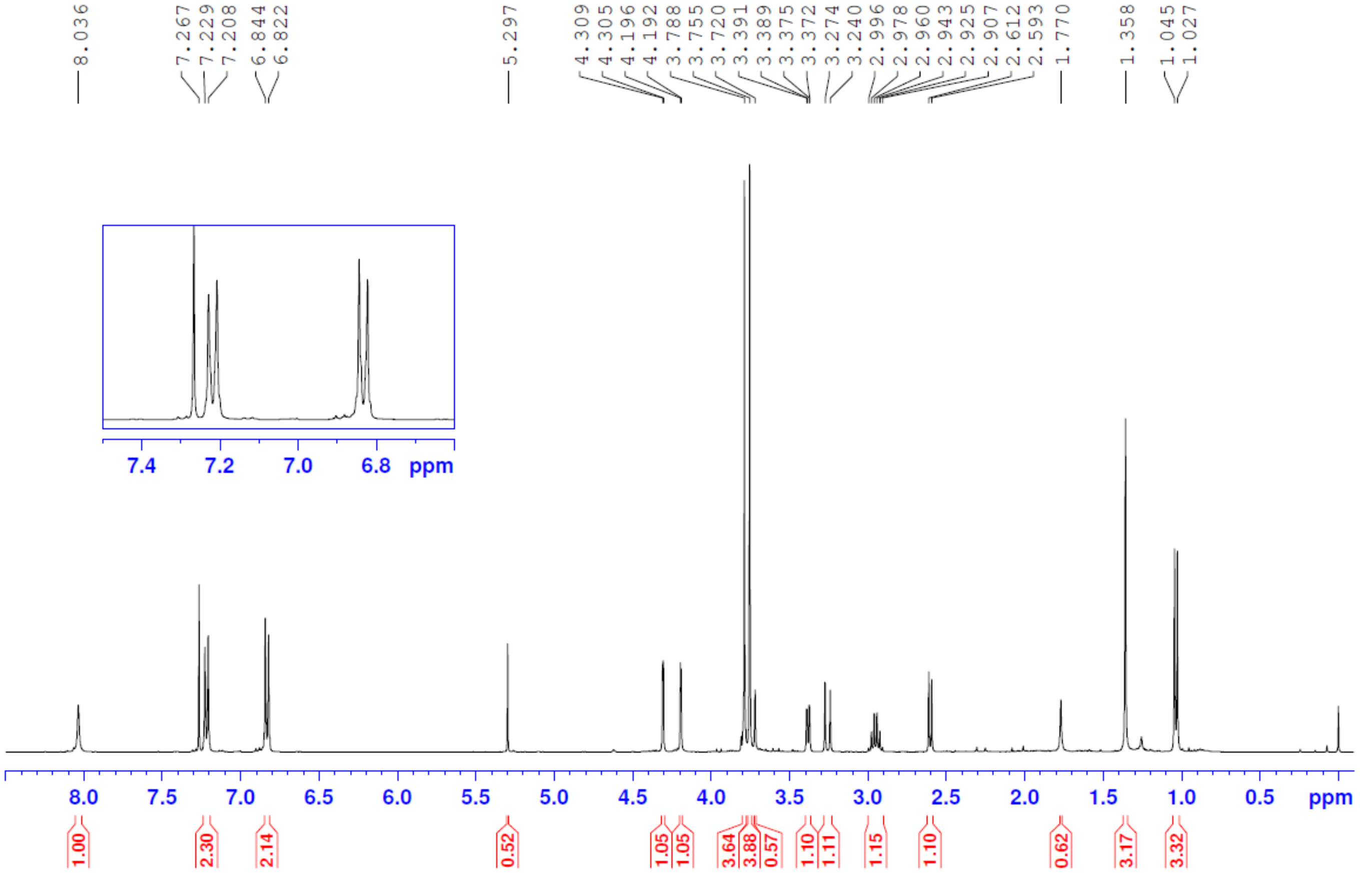

^{1}H-NMR (400 MHz, CDCl$_3$), δ (ppm) – Aliphatic zoom

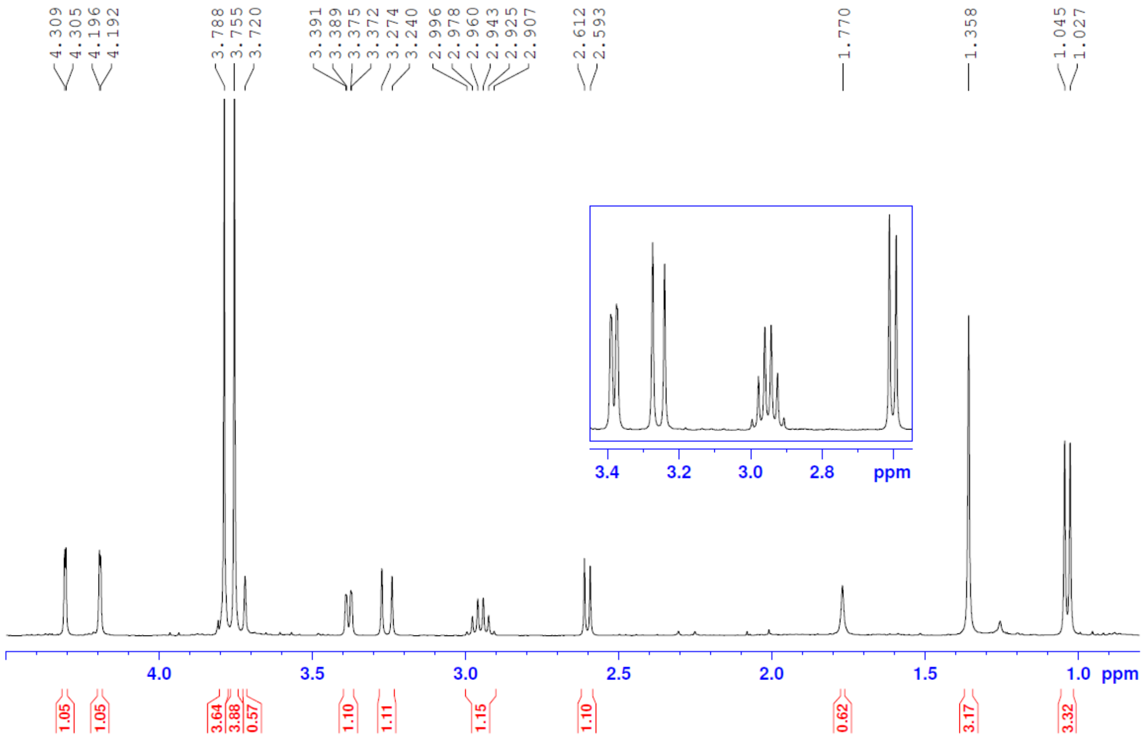

^{13}C{^{1}H}-NMR (100 MHz, CDCl$_3$), δ (ppm)

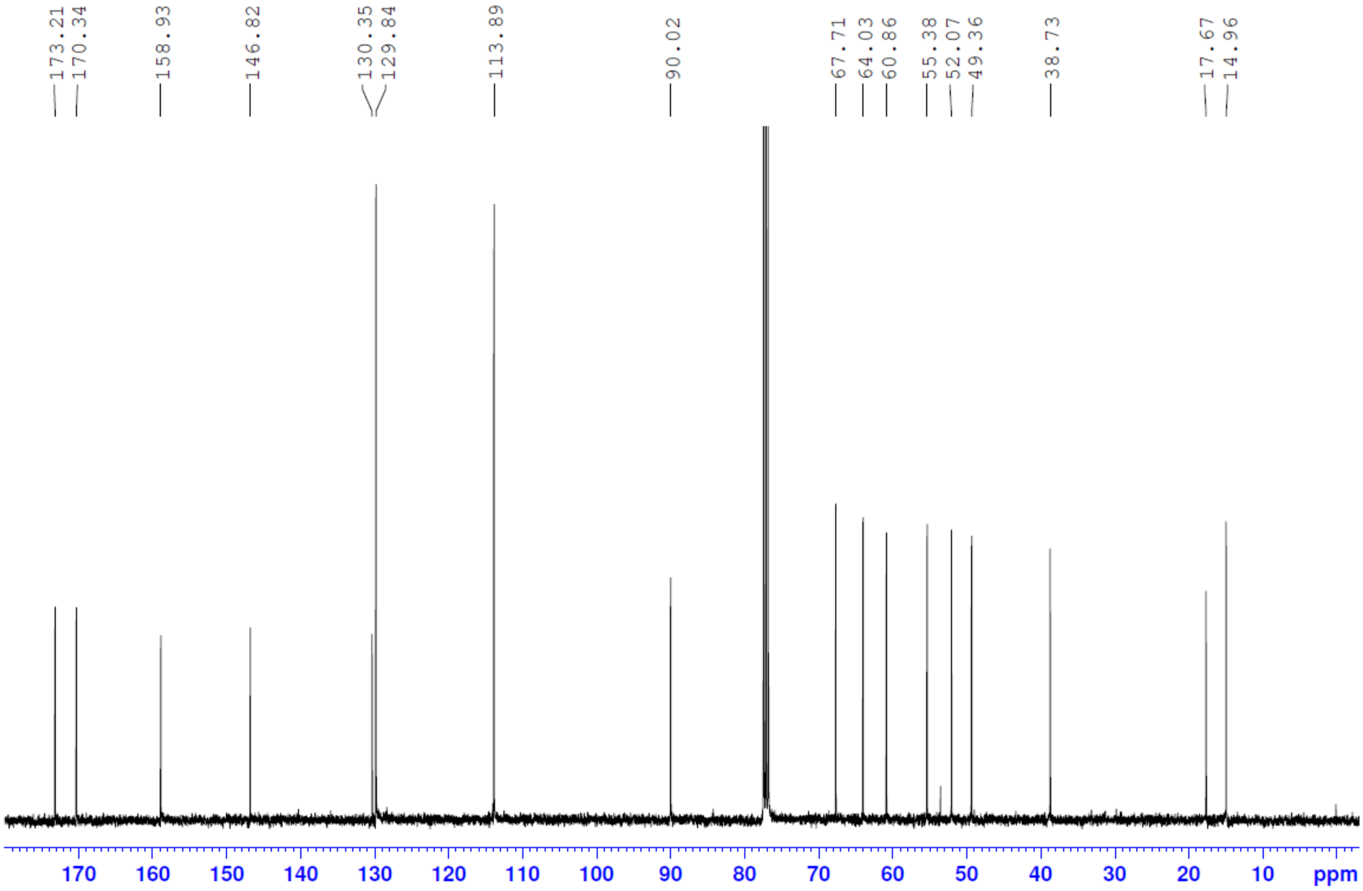

COSY ^1H-^1H ($CDCl_3$), δ (ppm)

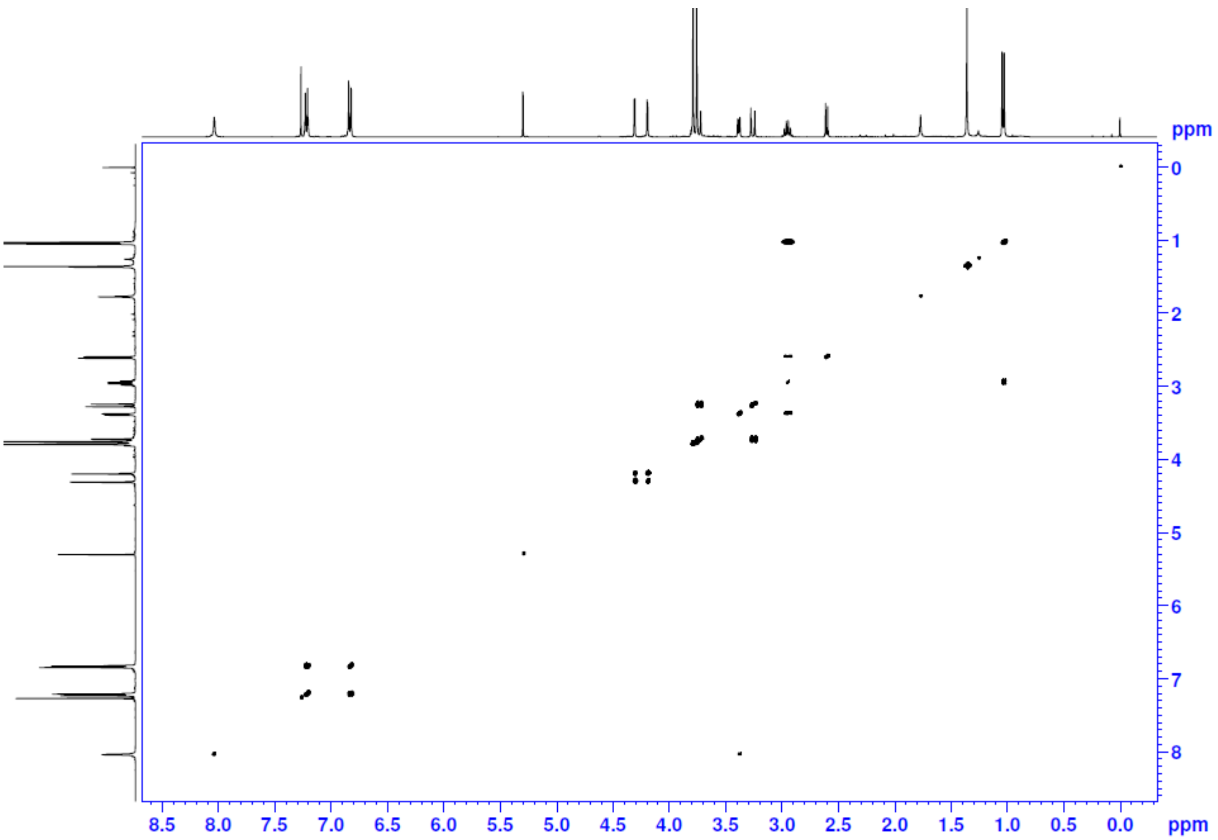

COSY ^1H-^1H ($CDCl_3$), δ (ppm) – Aliphatic Zoom

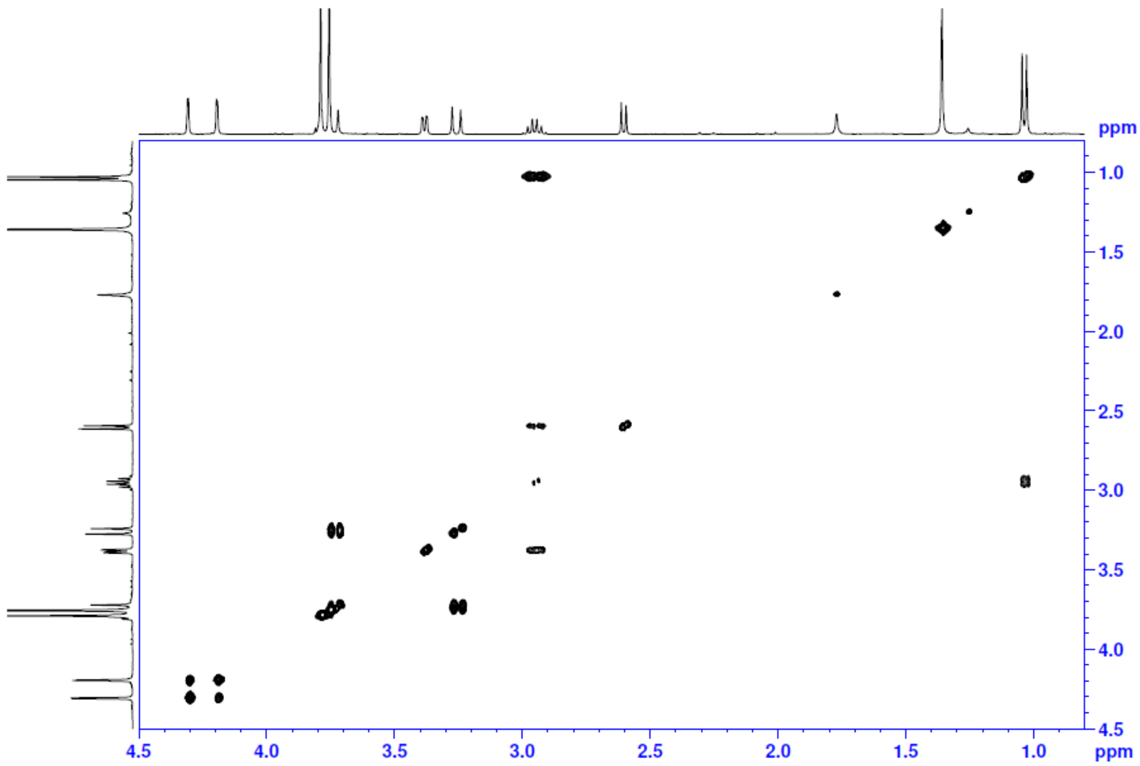

NOESY ^1H-^1H ($CDCl_3$), δ (ppm)

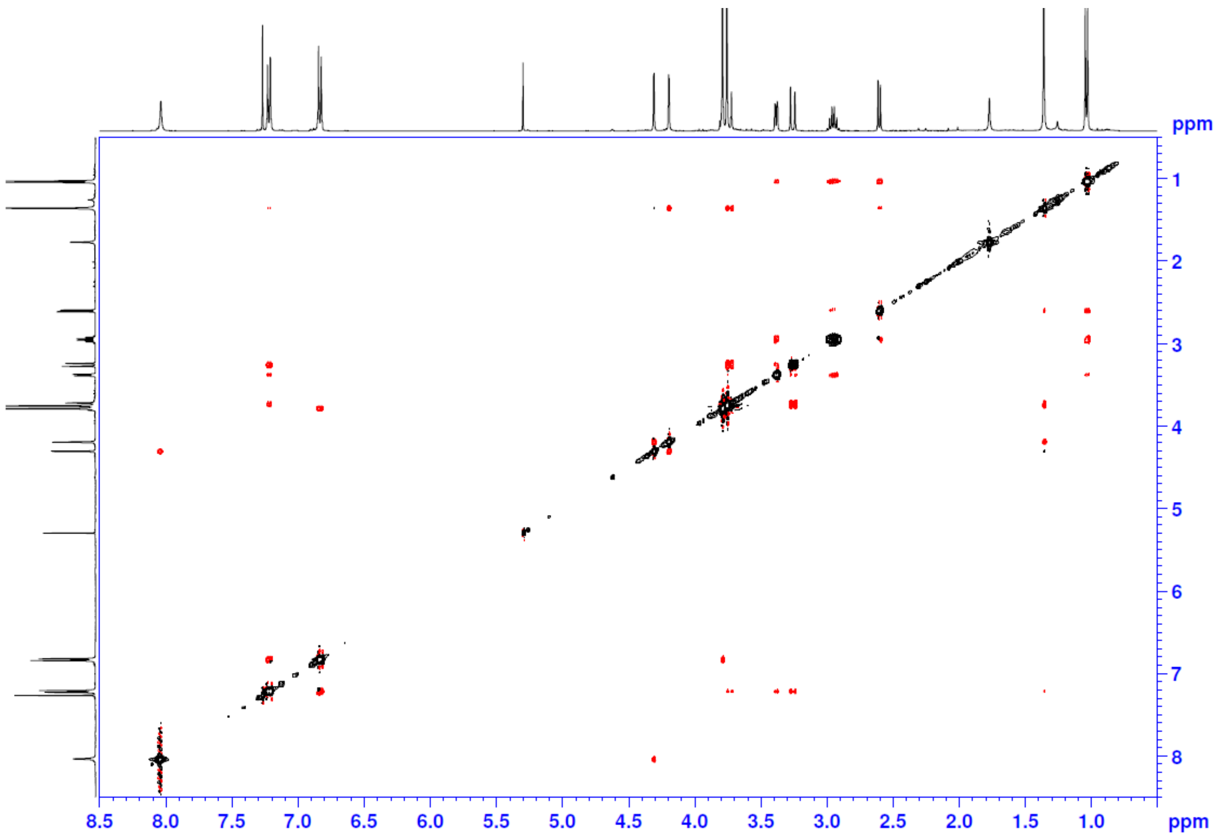

NOESY ^1H-^1H ($CDCl_3$), δ (ppm) – Aliphatic Zoom

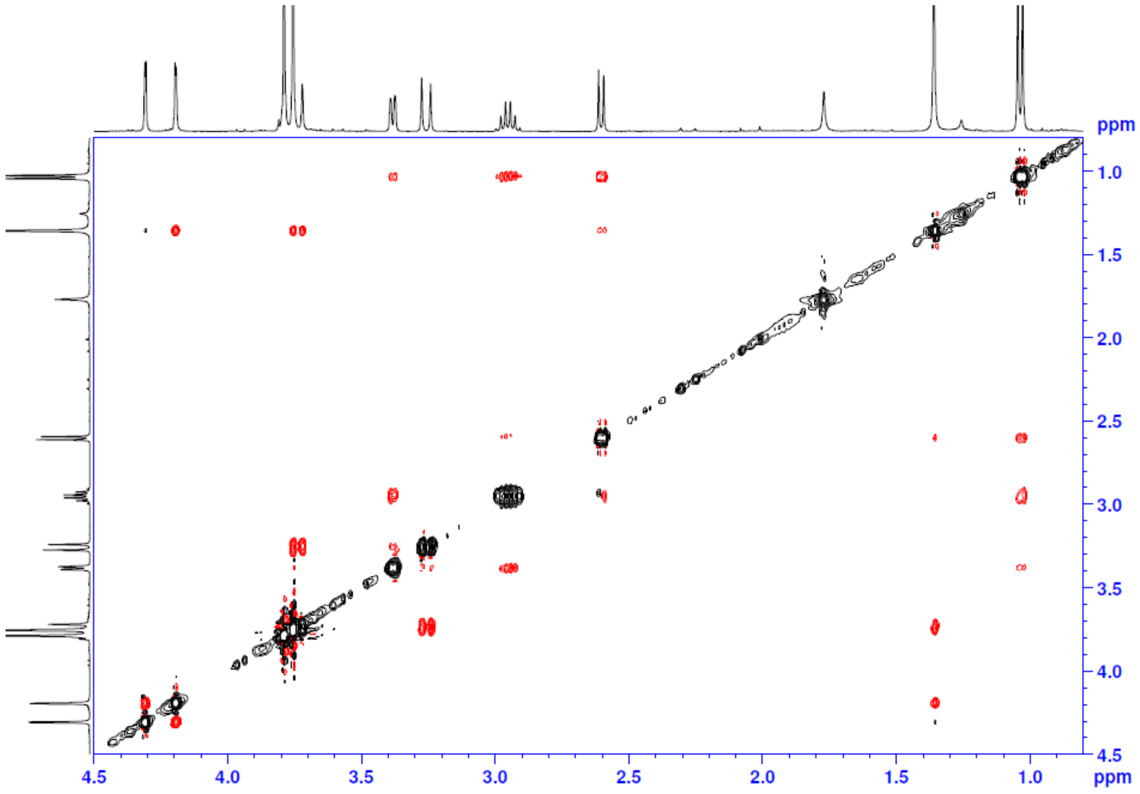

HSQC ^1H-^{13}C ($CDCl_3$), δ (ppm)

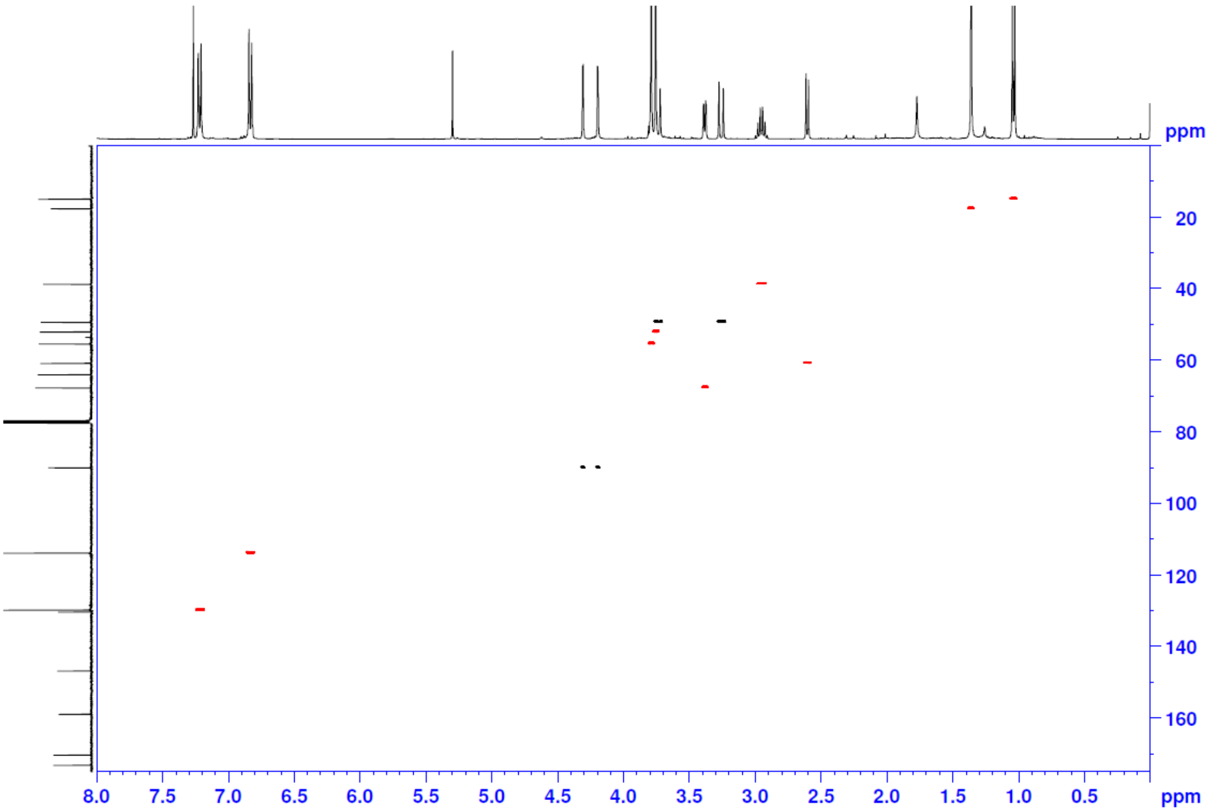

HMBC ^1H-^{13}C ($CDCl_3$), δ (ppm)

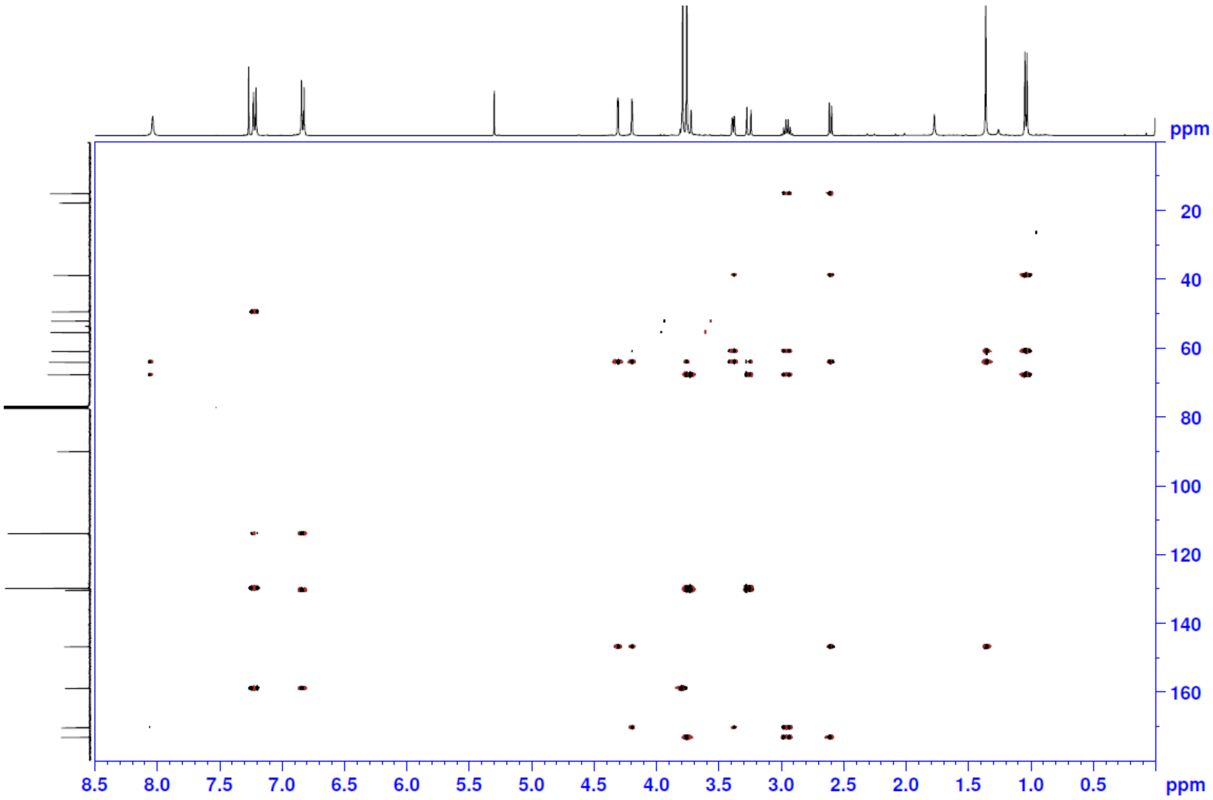

FT-IR (neat), ν (cm^{-1})

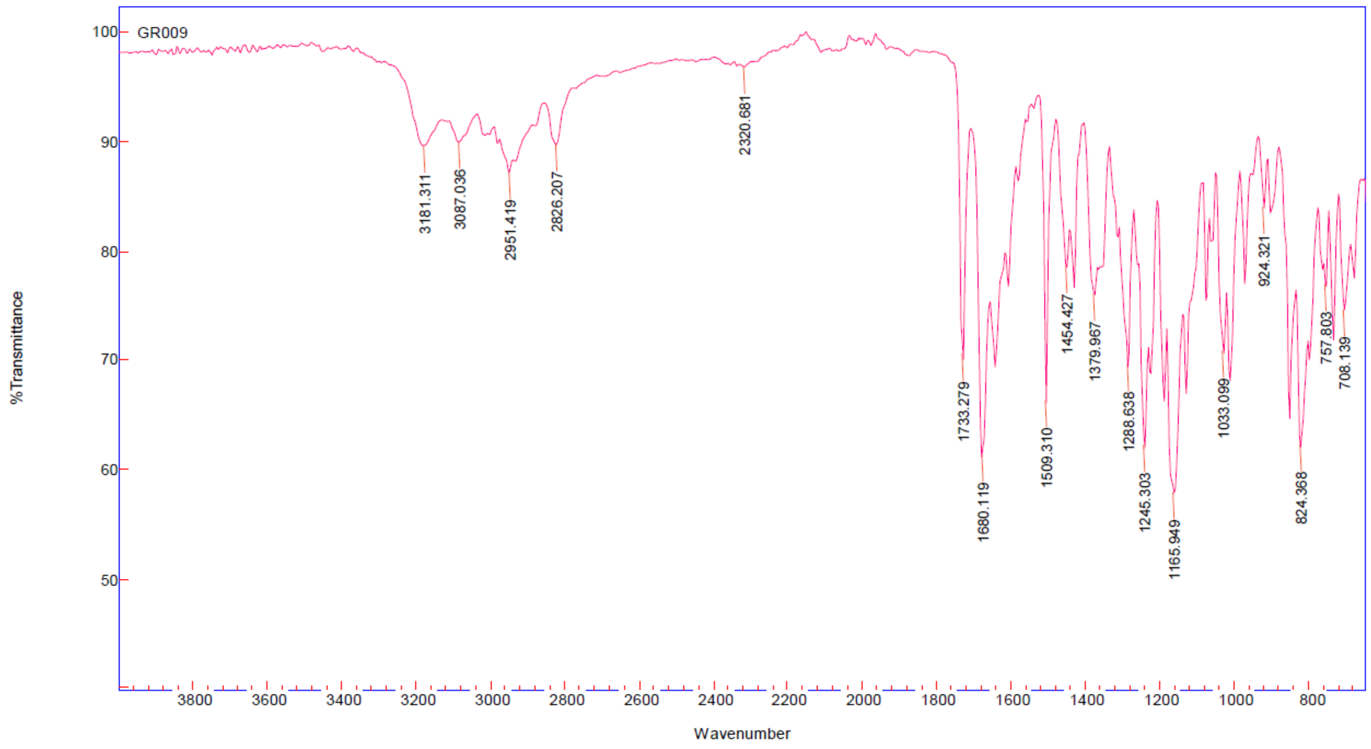

HPLC (λ = 220 nm)

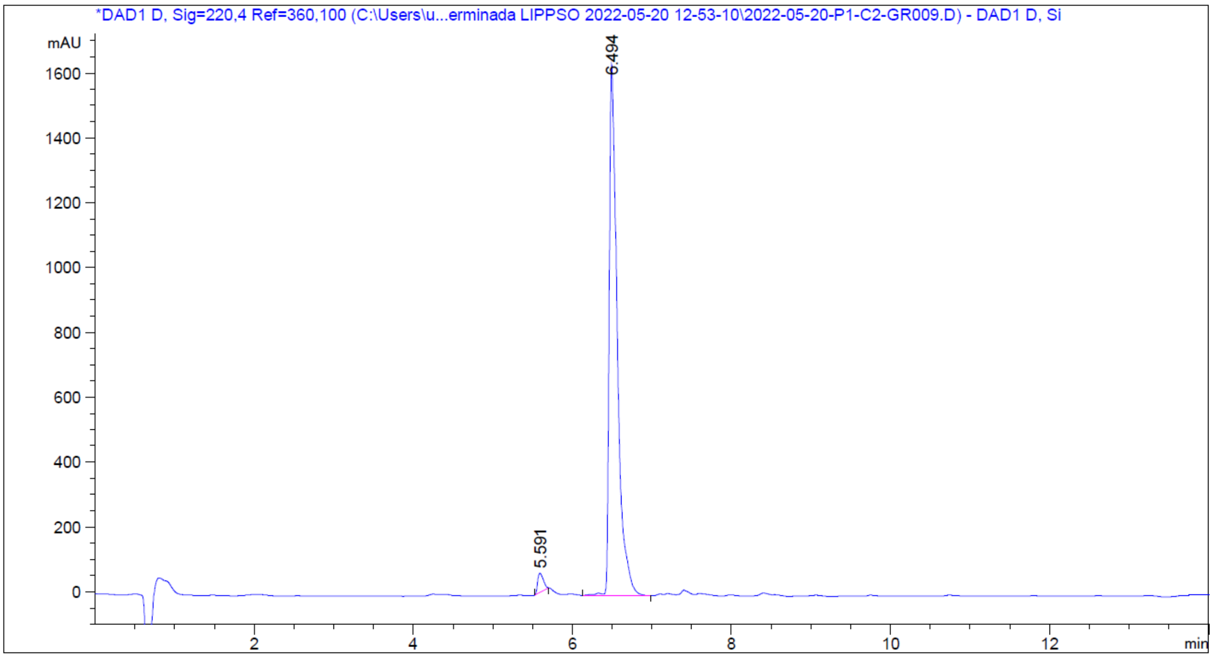

Peak #	RetTime [min]	Type	Width [min]	Area [mAU*s]	Height [mAU]	Area %
1	5.591	BBA	0.0795	281.11316	59.04367	2.3840
2	6.494	VB R	0.0969	1.15104e4	1639.57458	97.6160

Totals : 1.17915e4 1698.61826

ESI-MS (*m/z*)

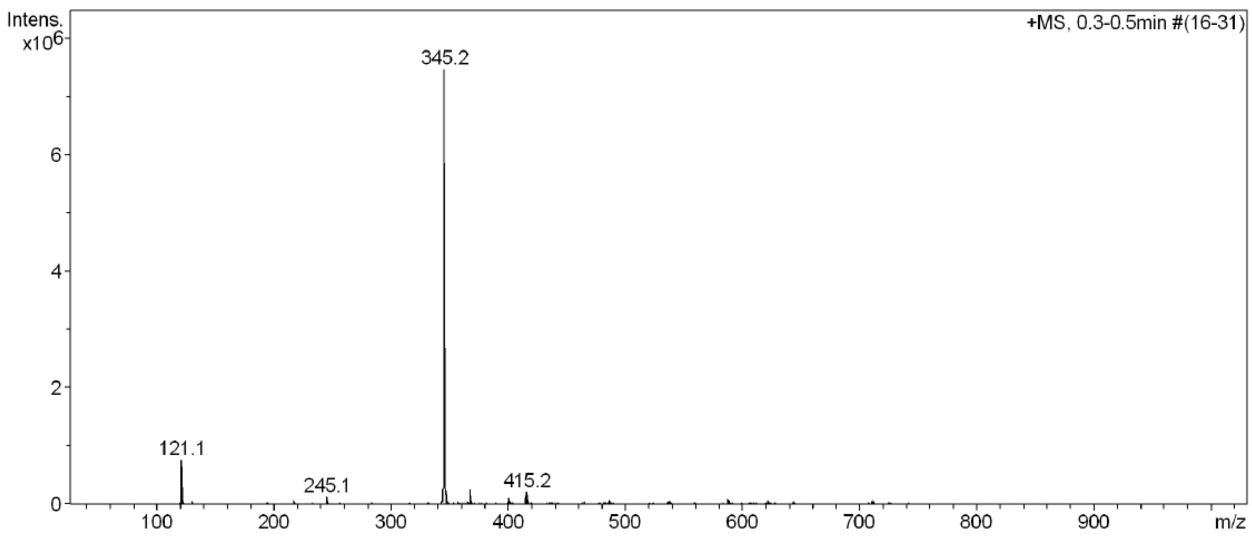

HRMS (*m/z*)

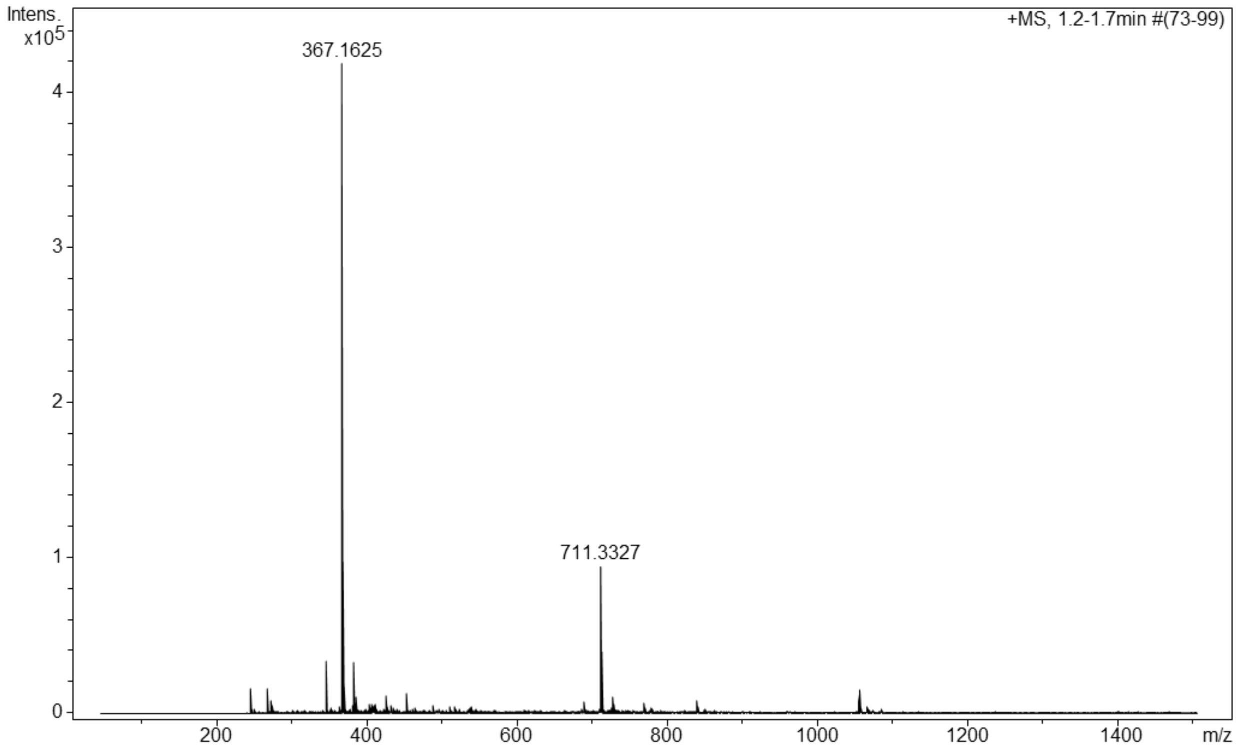

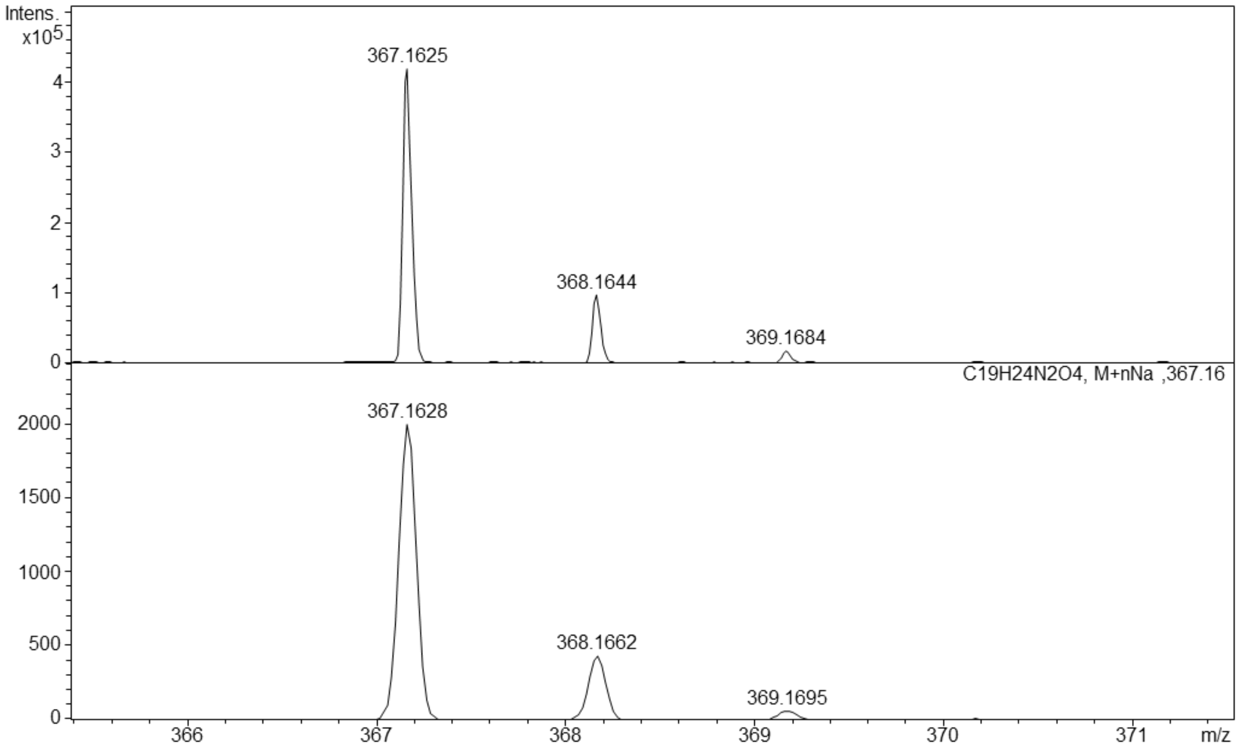

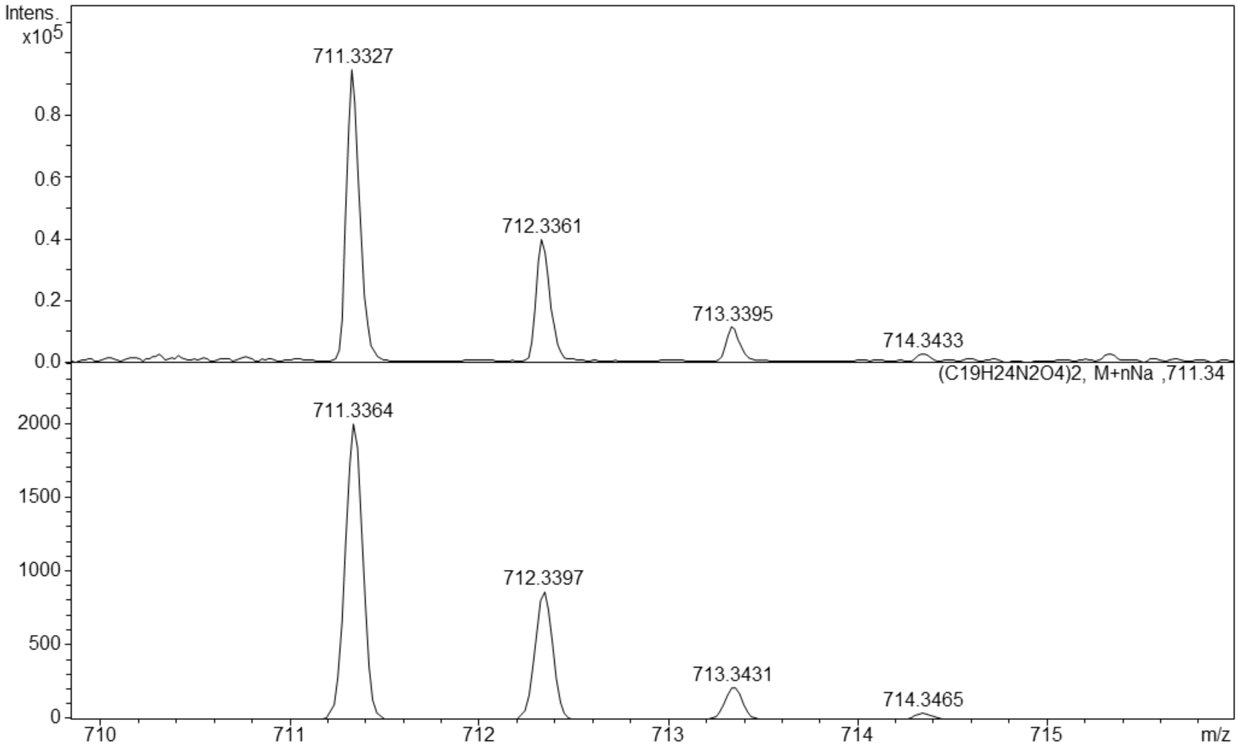

	Molecular formula	Calculated	Found
**[M+Na]$^+$**	$C_{19}H_{24}N_2O_4Na$	367.1628	367.1625
**[2M+Na]$^+$**	$(C_{19}H_{24}N_2O_4)_2Na$	711.3364	711.3327

Methyl 8-(4-methoxybenzyl)-5-methyl-4-methylene-2-oxo-7-phenyl-3,8-diazabicyclo[3.2.1]octane-6-carboxylate (20da) and methyl 8-(4-methoxybenzyl)-1-methyl-2-methylene-4-oxo-7-phenyl-3,8-diazabicyclo[3.2.1]octane-6-carboxylate (20db)

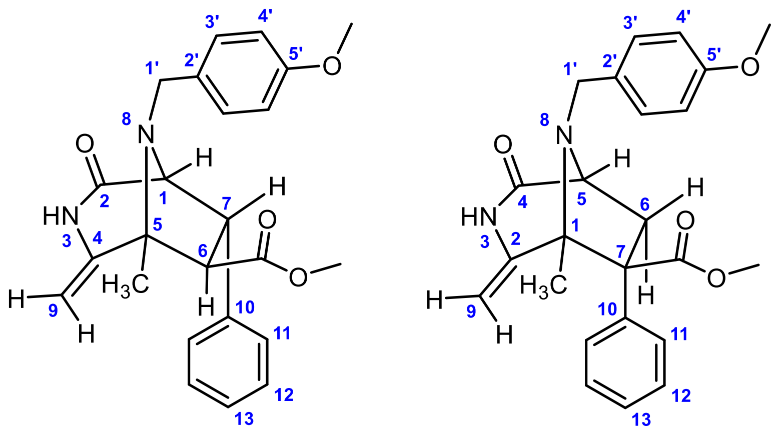

20da **20db**

^{1}H-NMR (400 MHz, CDCl$_3$), δ (ppm)

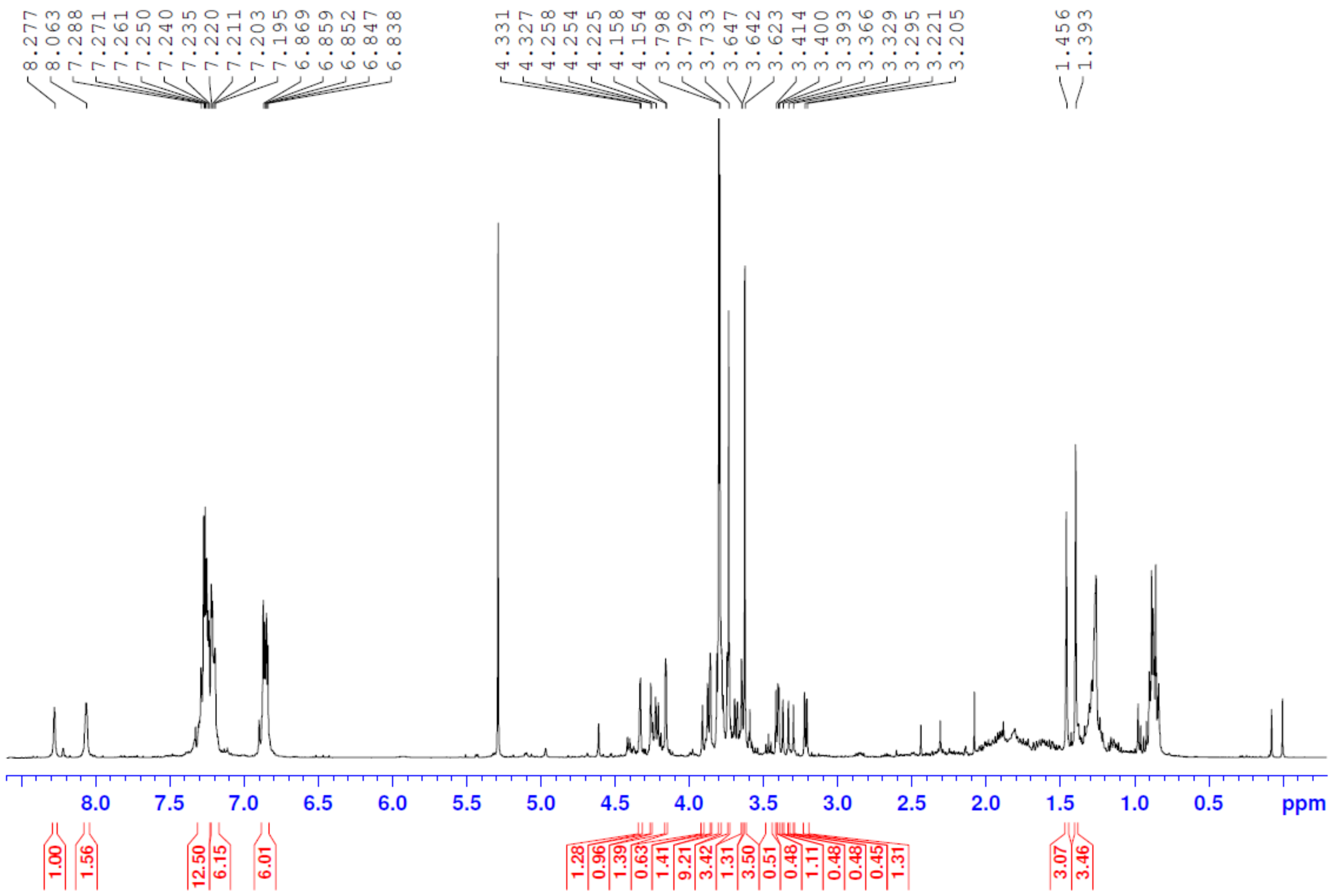

$^{13}C\{^1H\}$-NMR (100 MHz, CDCl₃), δ (ppm)

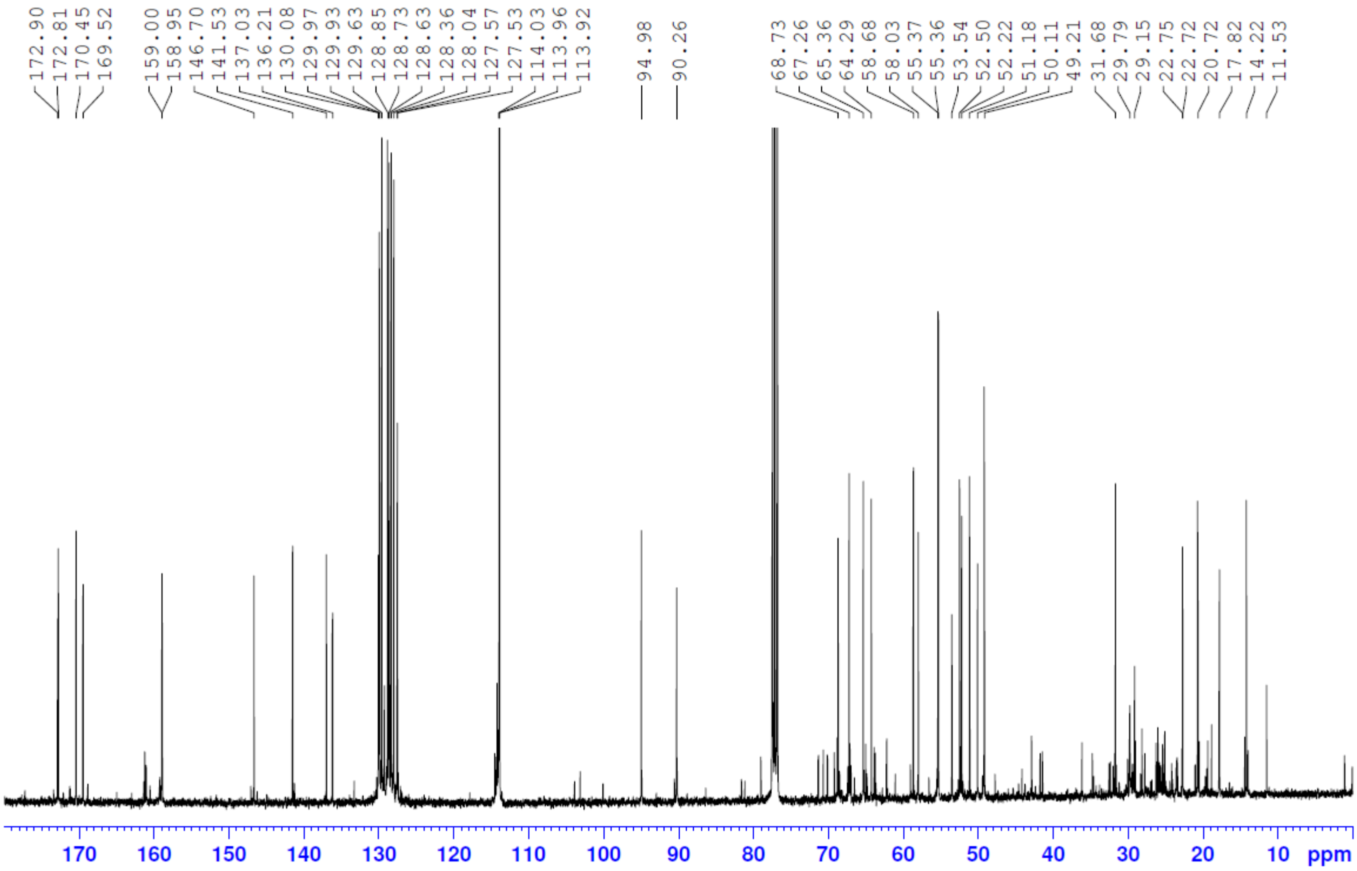

COSY ^1H-^1H (CDCl₃), δ (ppm)

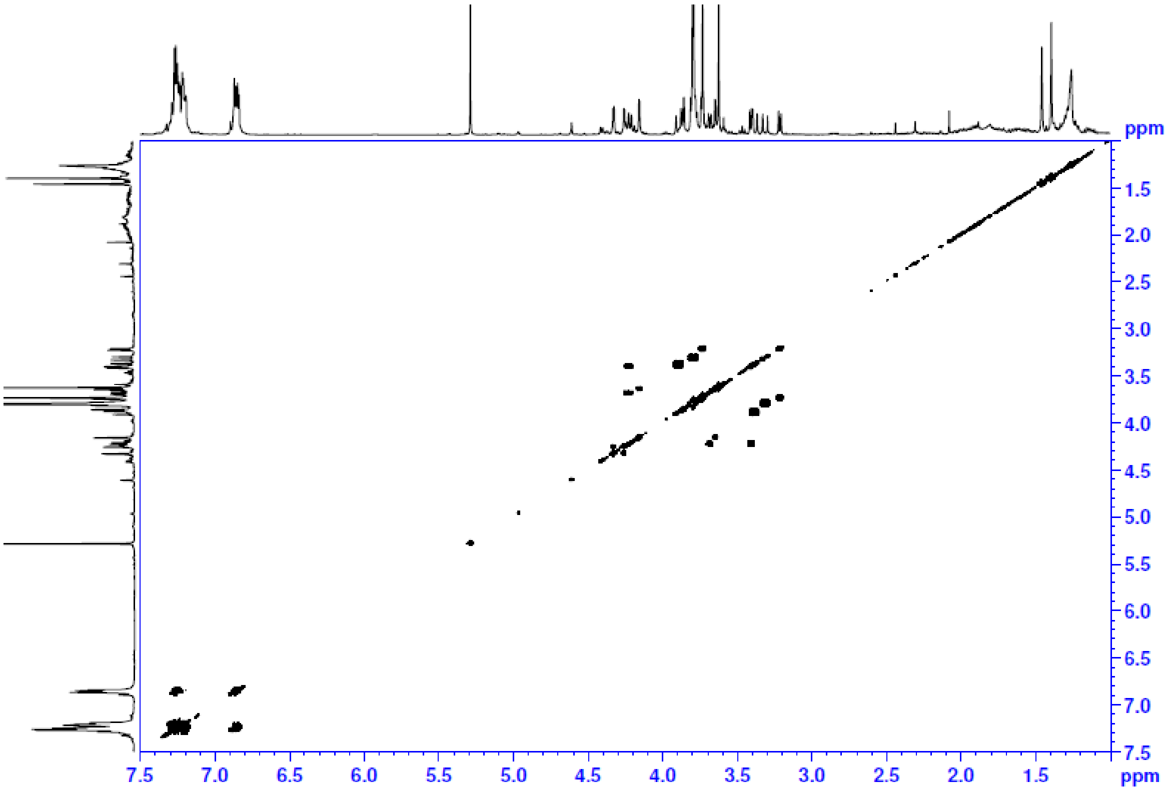

NOESY ^{1}H-^{1}H ($CDCl_3$), δ (ppm)

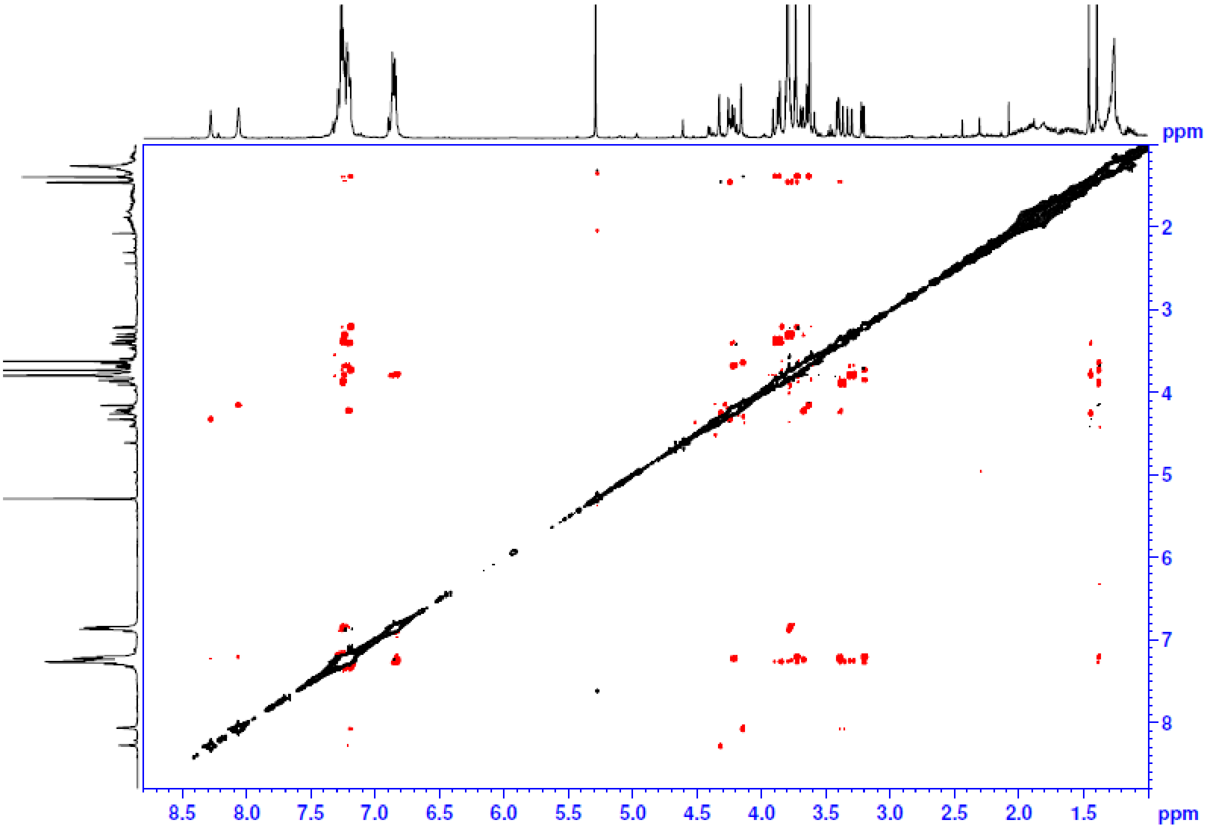

HSQC ^{1}H-^{13}C ($CDCl_3$), δ (ppm)

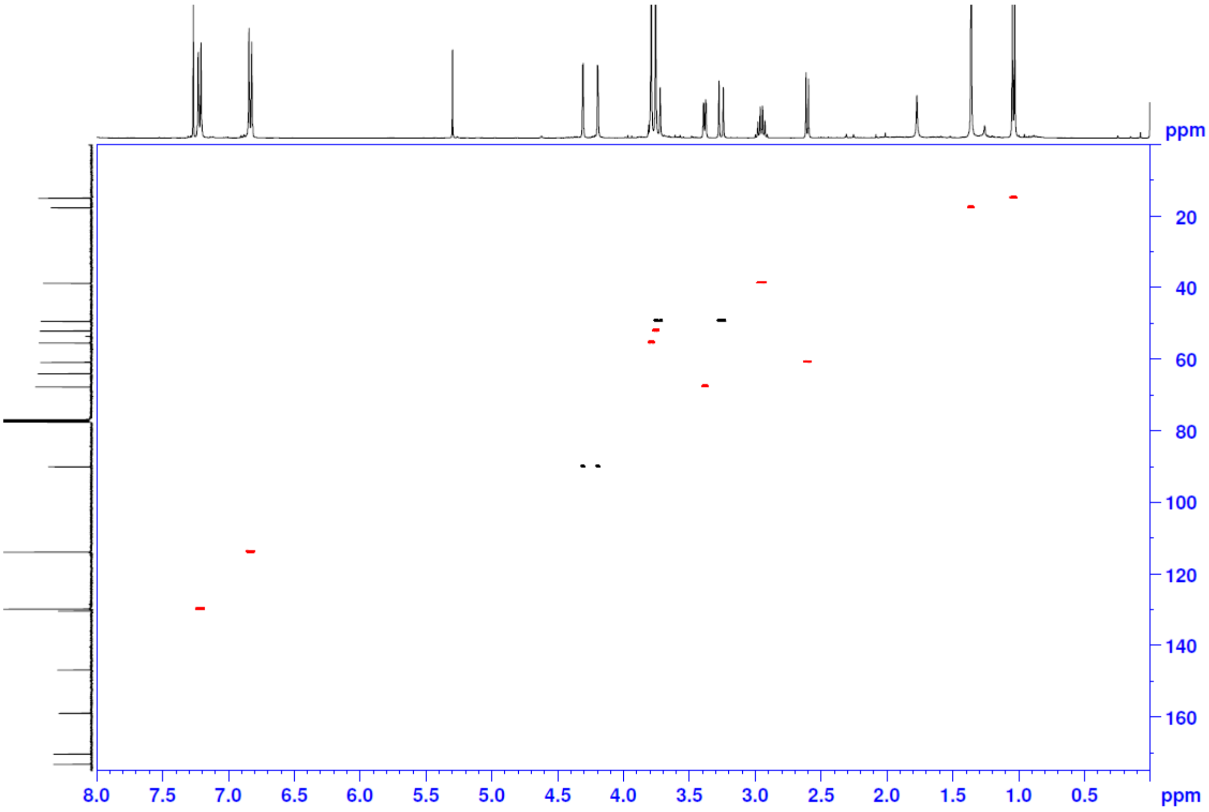

ESI-MS (*m/z*)

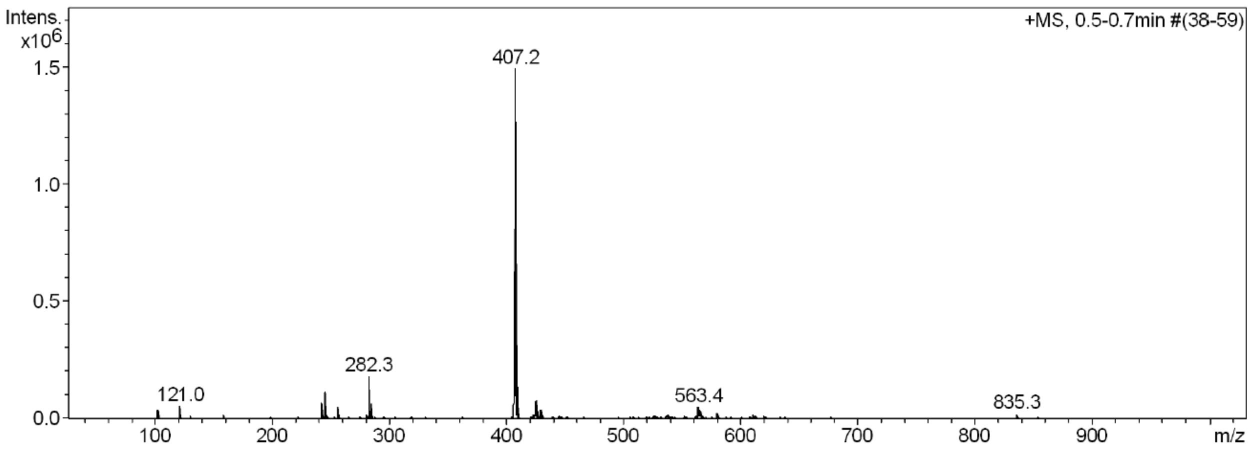

HRMS (*m/z*)

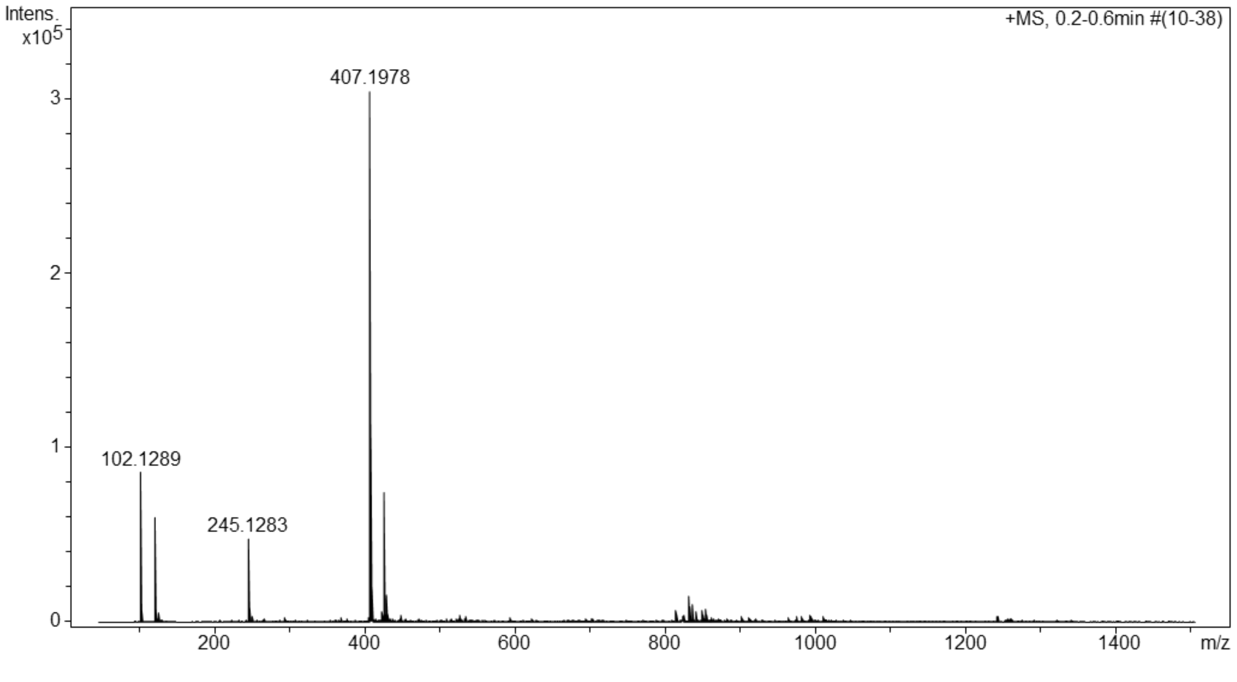

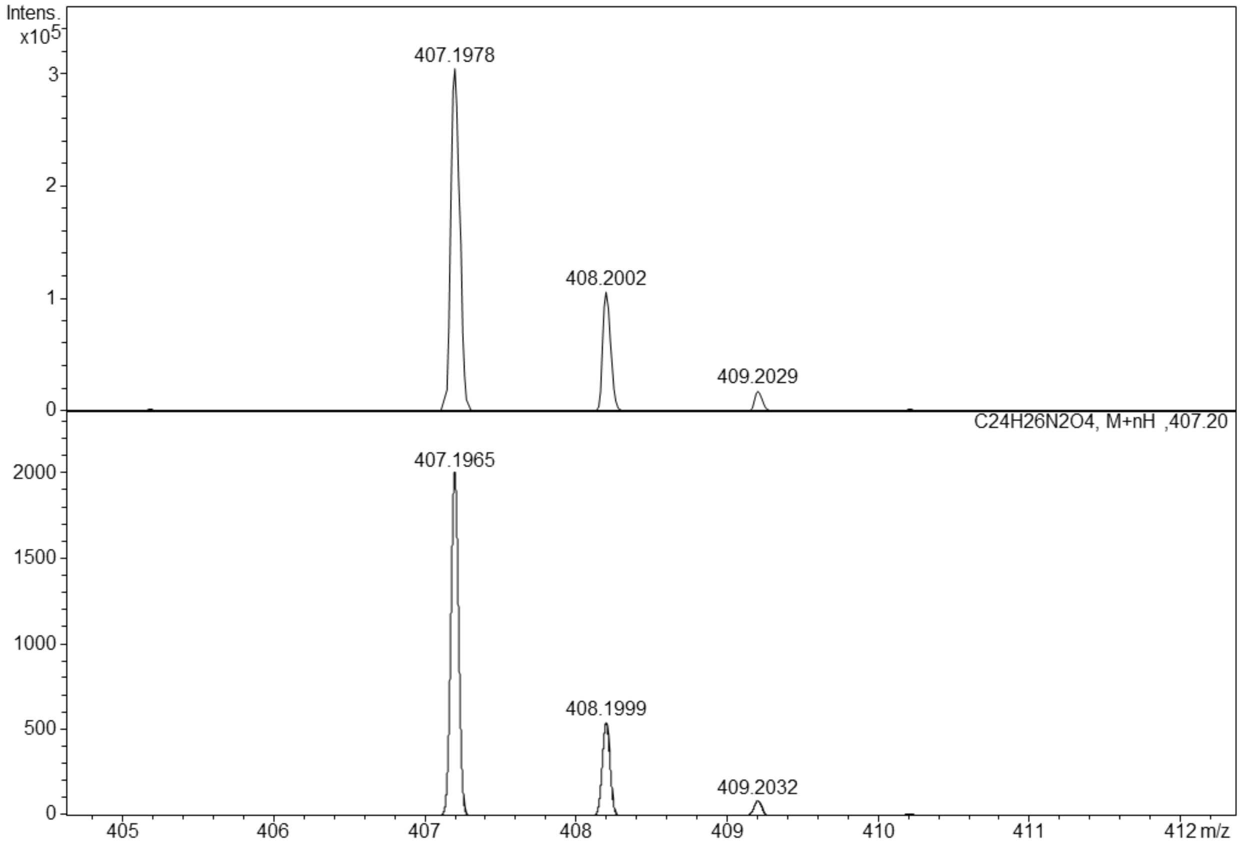

	Molecular formula	Calculated	Found
**[M+H]⁺**	$C_{24}H_{26}N_2O_4$	407.1965	407.1978

Methyl 8-(4-methoxybenzyl)-5-methyl-4-methylene-7-(4-nitrophenyl)-2-oxo-3,8-diazabicyclo[3.2.1]octane-6-carboxylate (20ea)

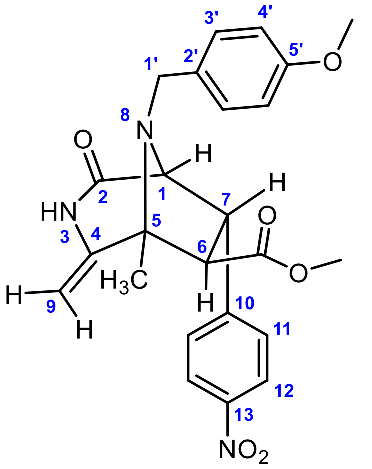

20ea

^{1}H-NMR (400 MHz, CDCl3), δ (ppm)

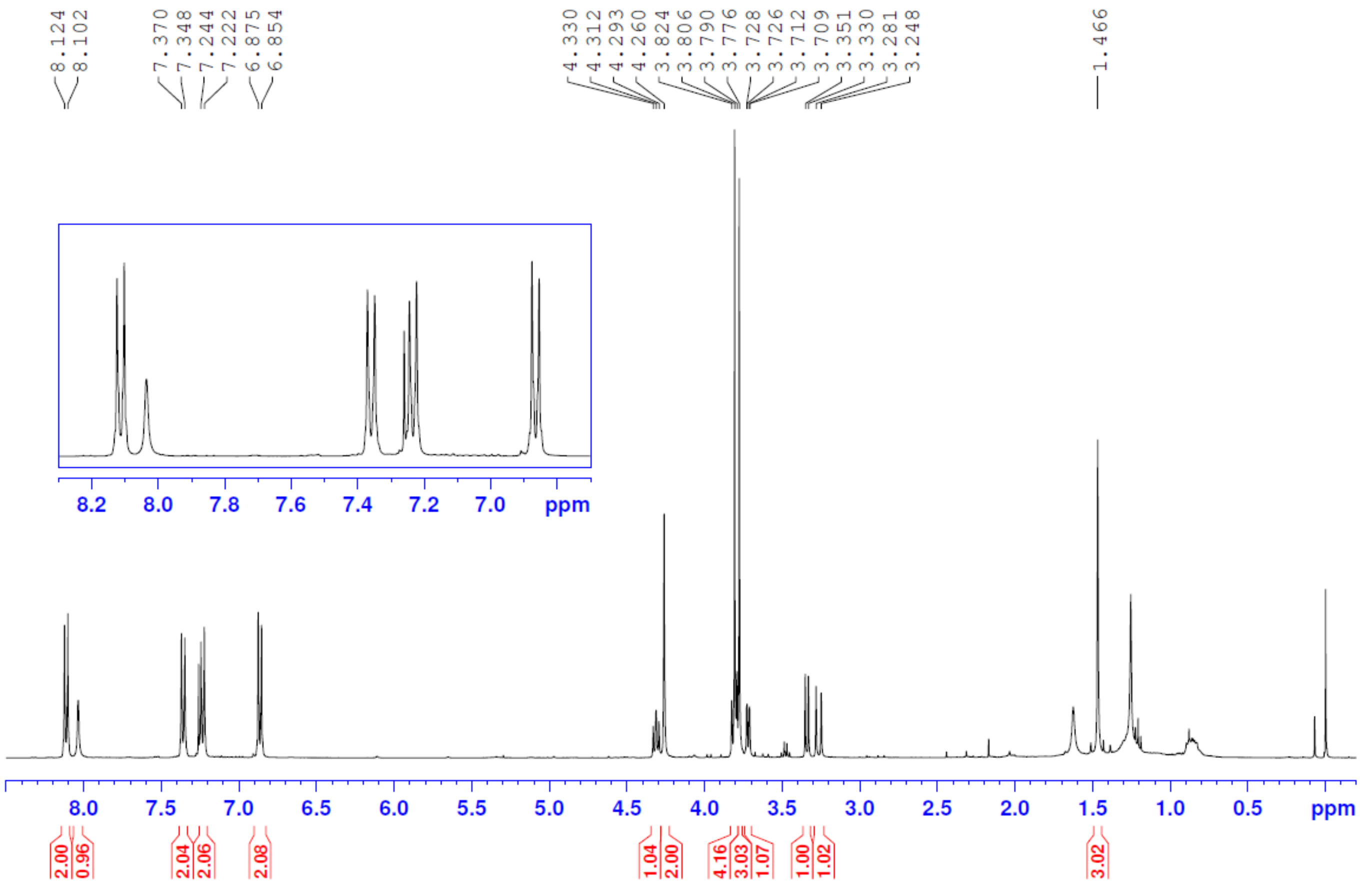

^{1}H-NMR (400 MHz, CDCl$_3$), δ (ppm) – Aliphatic zoom

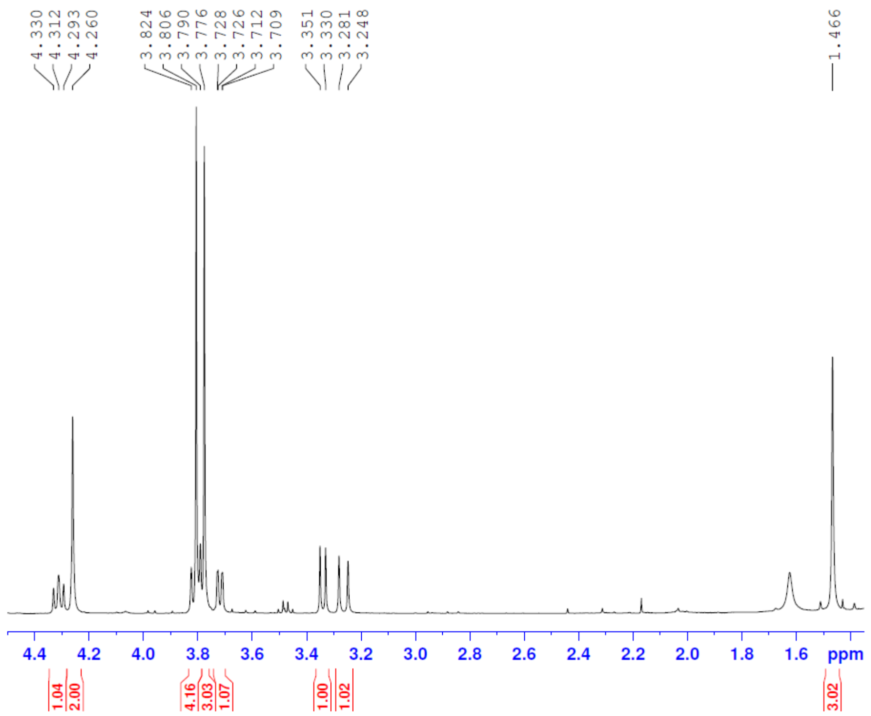

^{13}C{^{1}H}-NMR and DEPT135 (100 MHz, CDCl$_3$), δ (ppm)

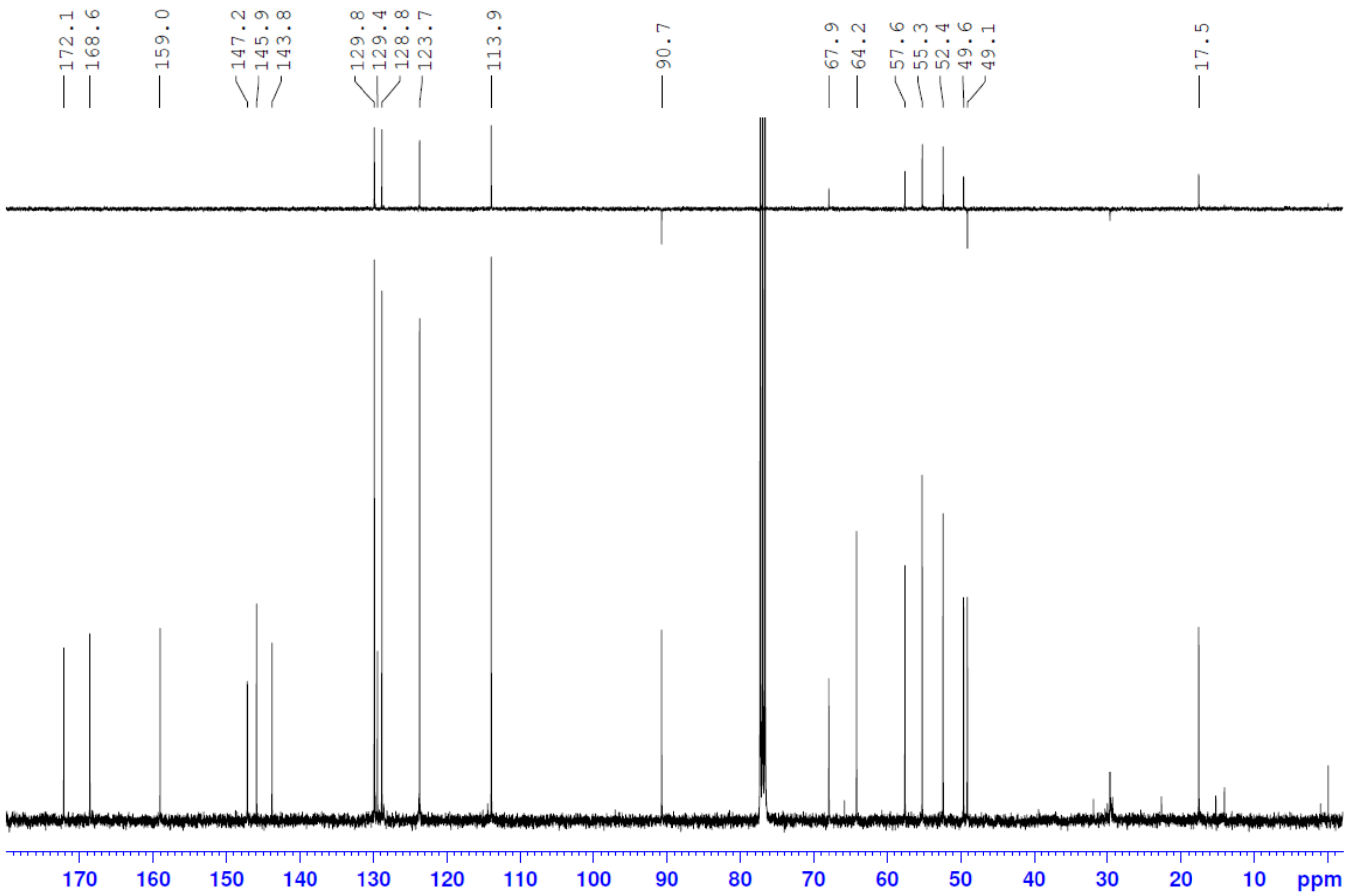

COSY ^{1}H-^{1}H ($CDCl_3$), δ (ppm)

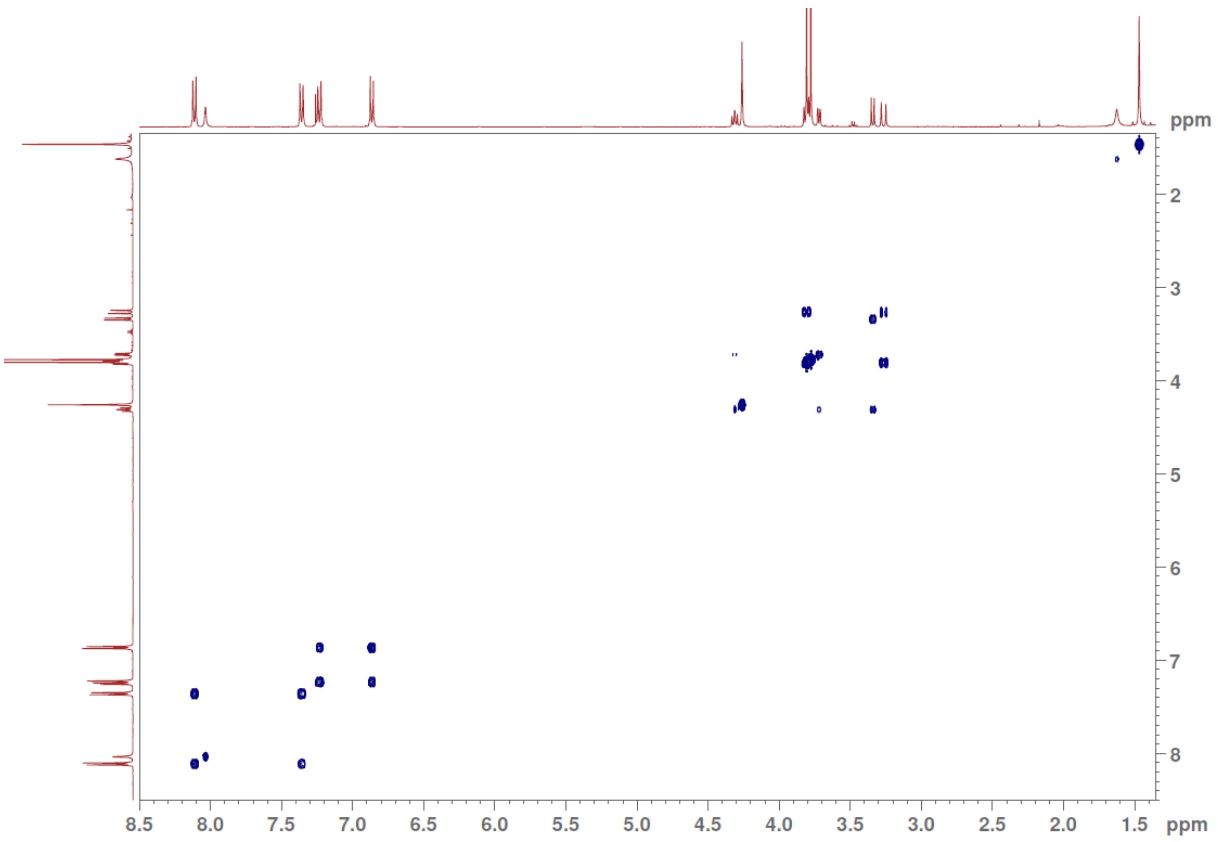

COSY ^{1}H-^{1}H ($CDCl_3$), δ (ppm) – Aliphatic Zoom

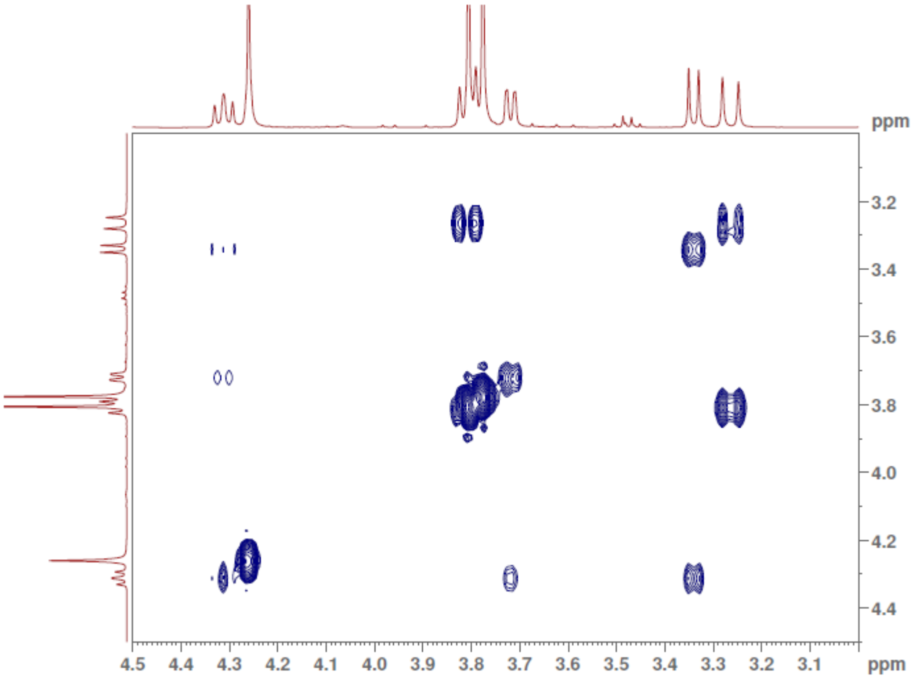

NOESY ^1H-^1H ($CDCl_3$), δ (ppm)

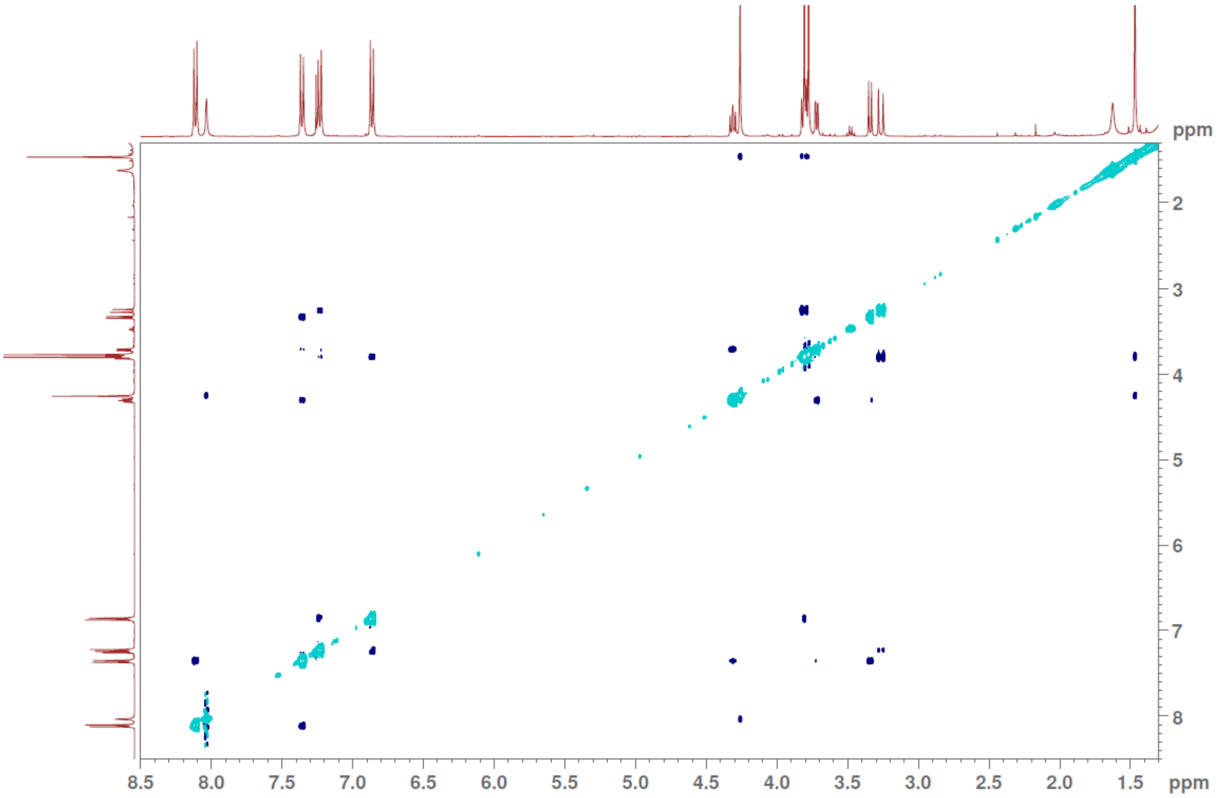

NOESY ^1H-^1H ($CDCl_3$), δ (ppm) – Aliphatic Zoom

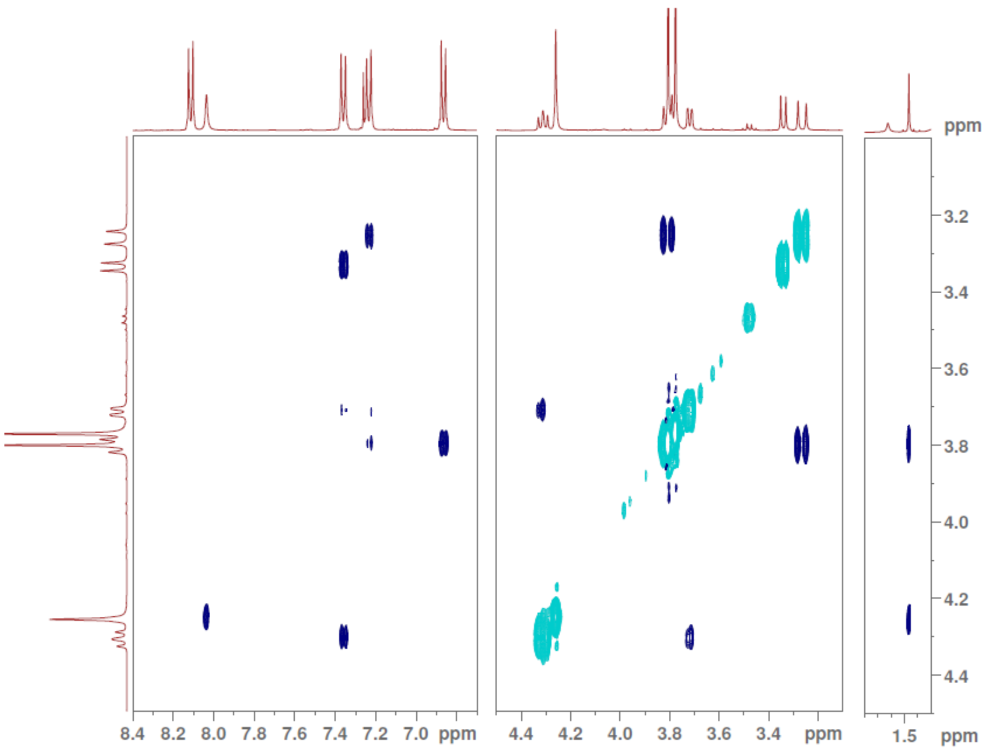

HSQC ^{1}H-^{13}C ($CDCl_3$), δ (ppm)

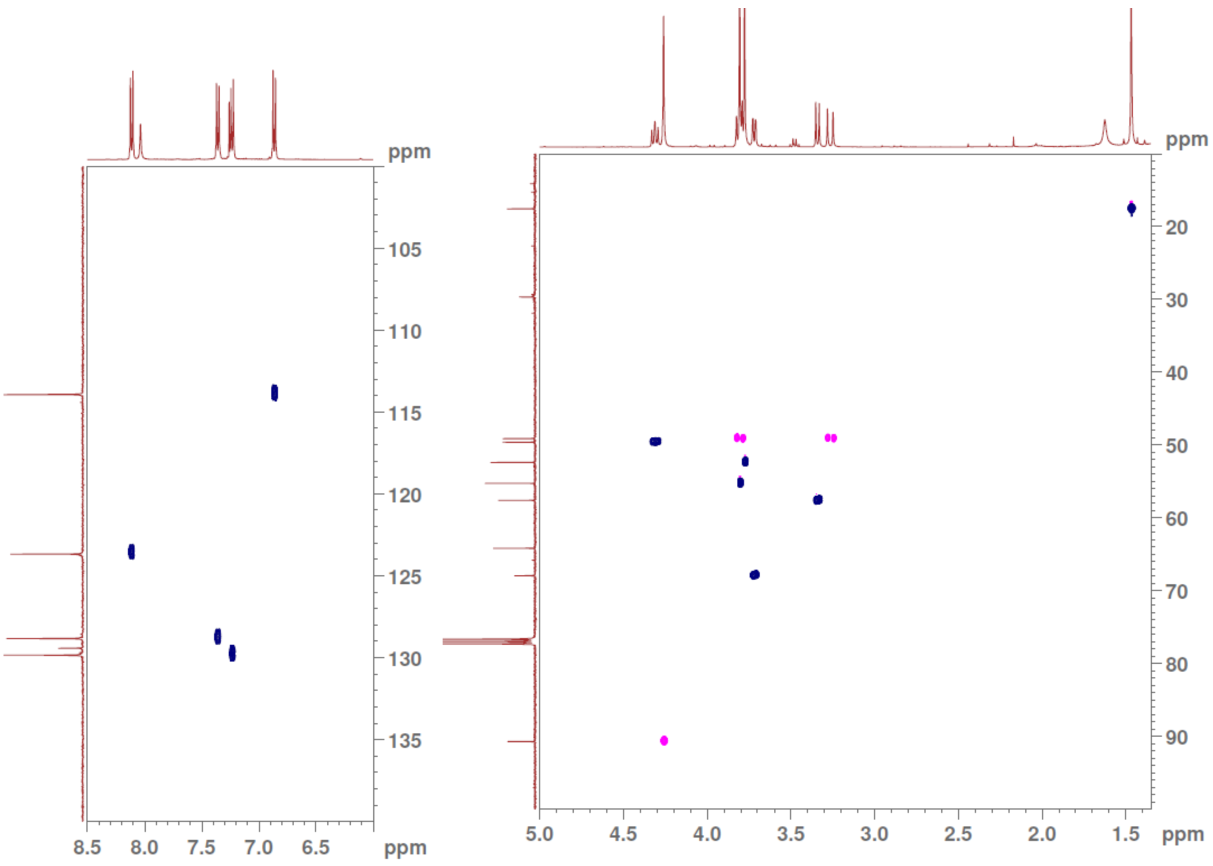

HMBC ^{1}H-^{13}C ($CDCl_3$), δ (ppm)

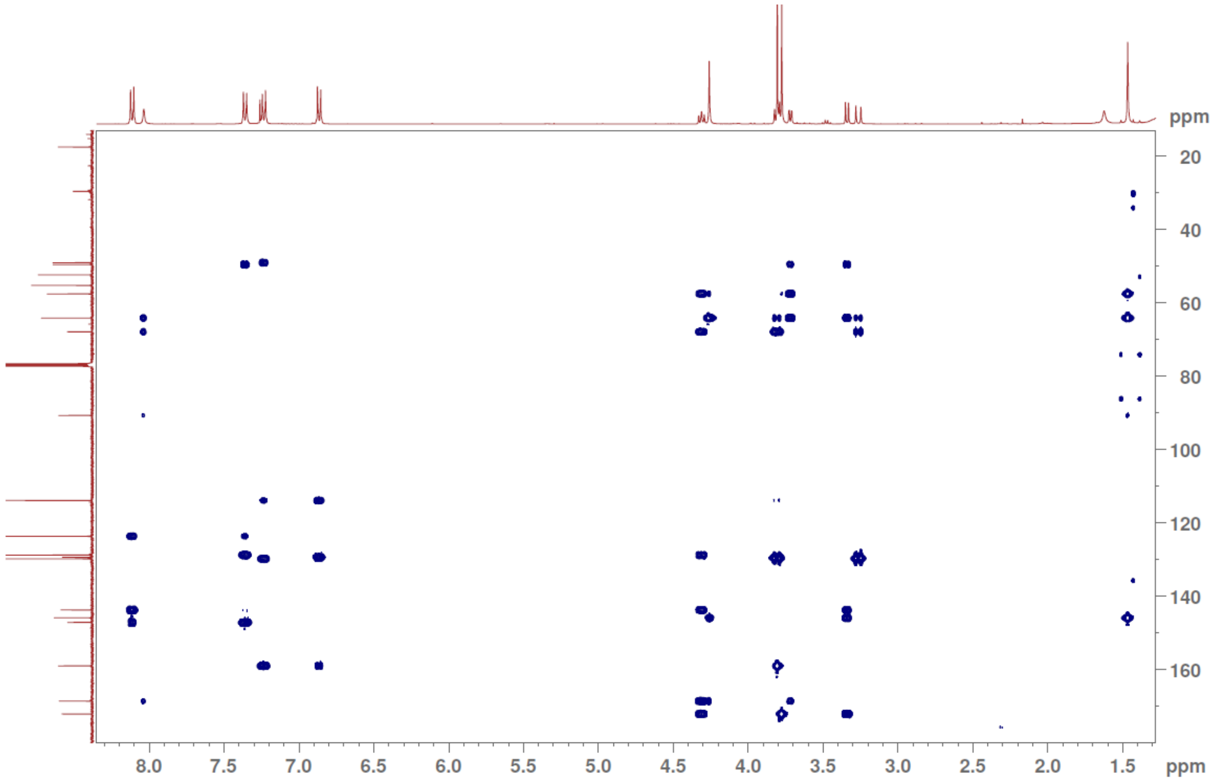

FT-IR (neat), ν (cm^{-1})

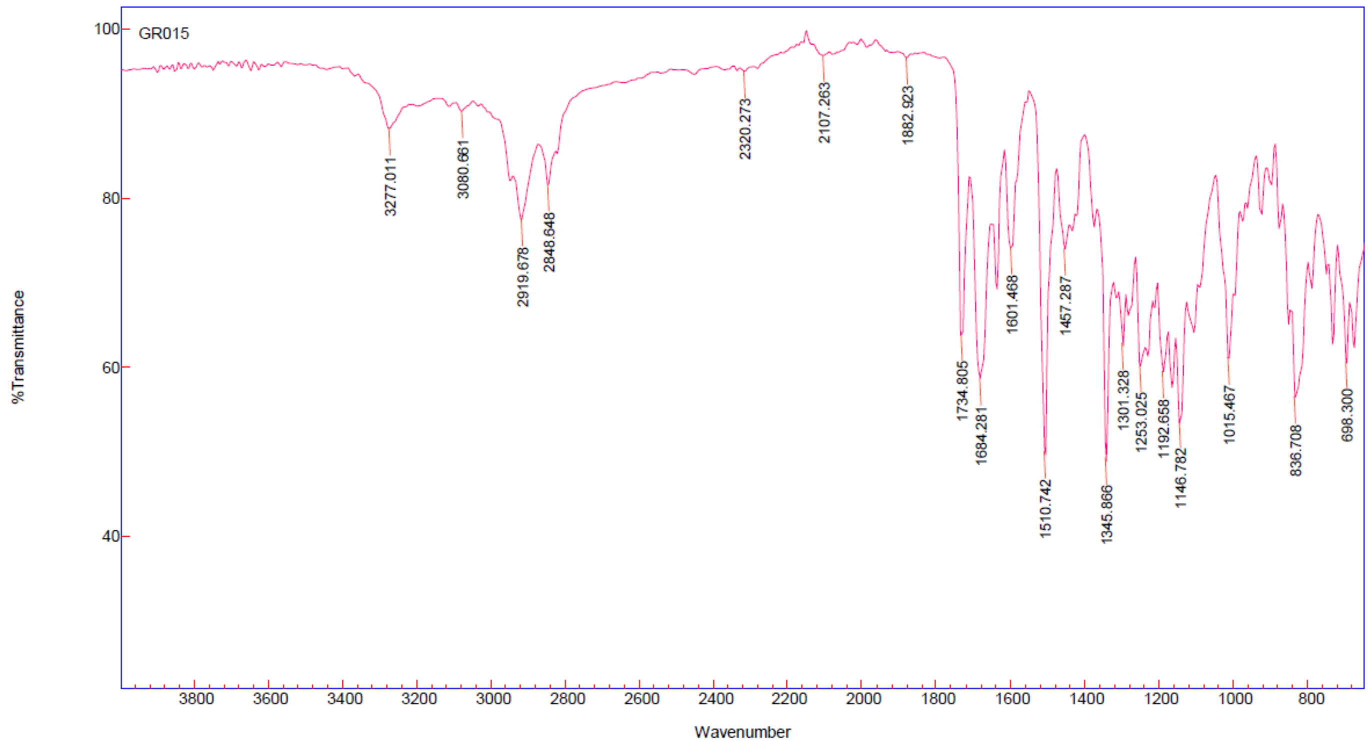

HPLC (λ = 220 nm)

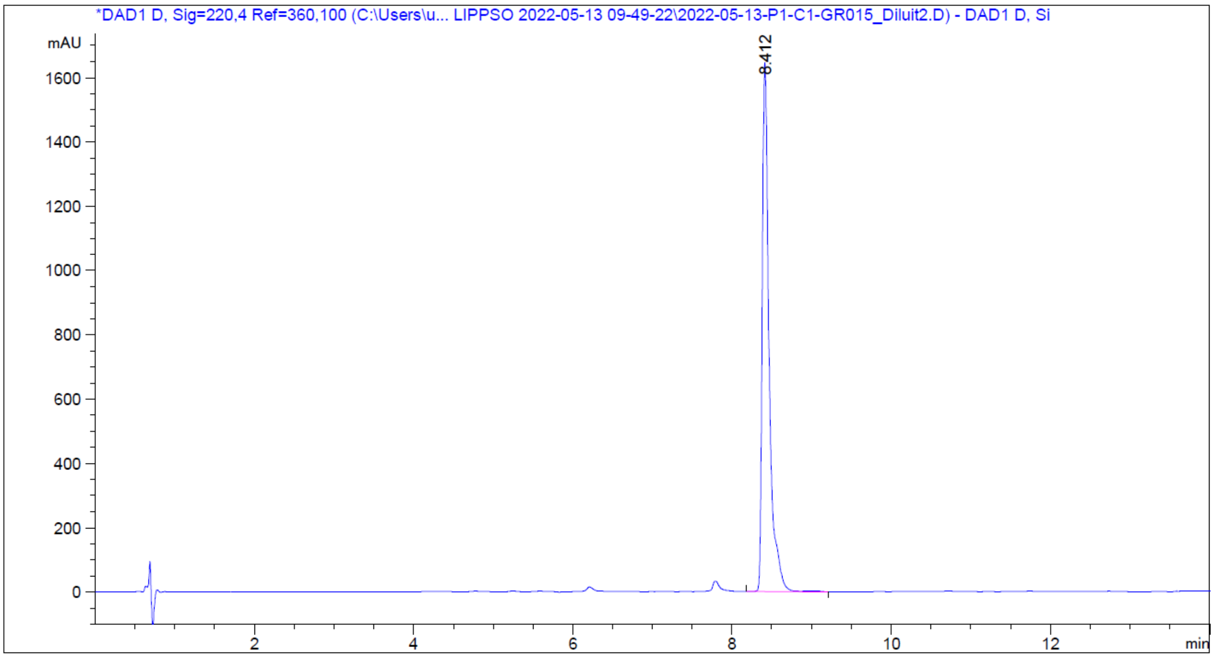

Peak #	RetTime [min]	Type	Width [min]	Area [mAU*s]	Height [mAU]	Area %
1	8.412	BV R	0.0904	9936.81445	1647.16992	100.0000

Totals : 9936.81445 1647.16992

ESI-MS (*m/z*)

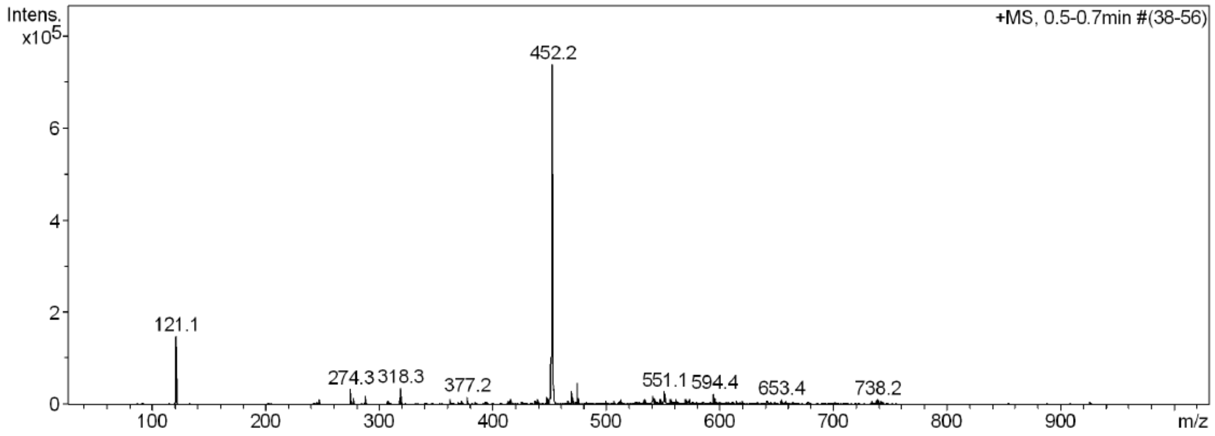

ESI-MS/MS (*m/z* = 452.2)

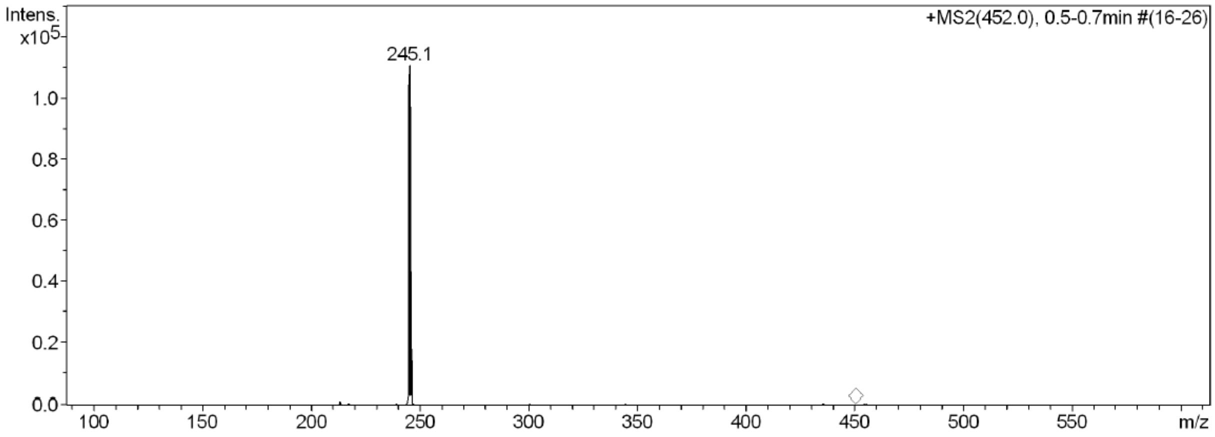

ESI-MS/MS/MS (*m/z* = 245.1)

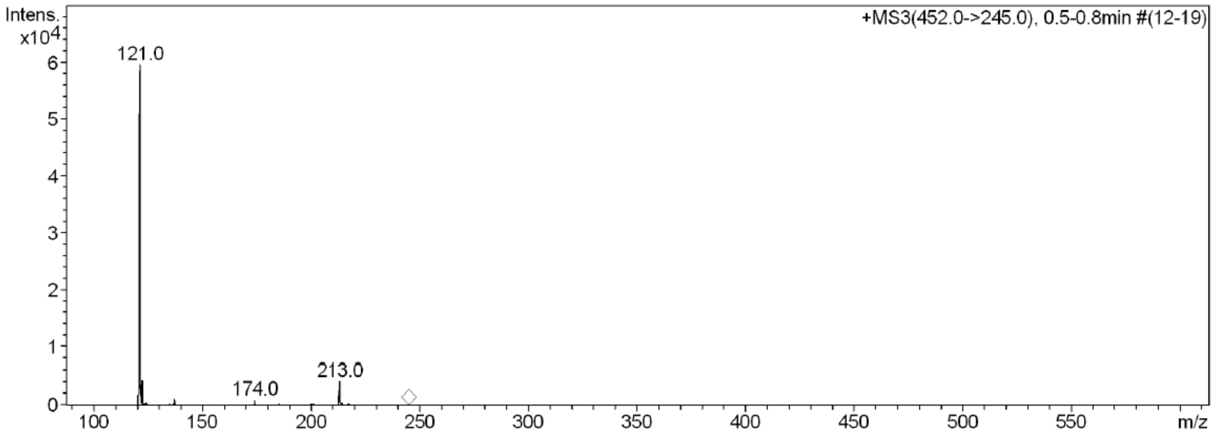

HRMS (*m/z*)

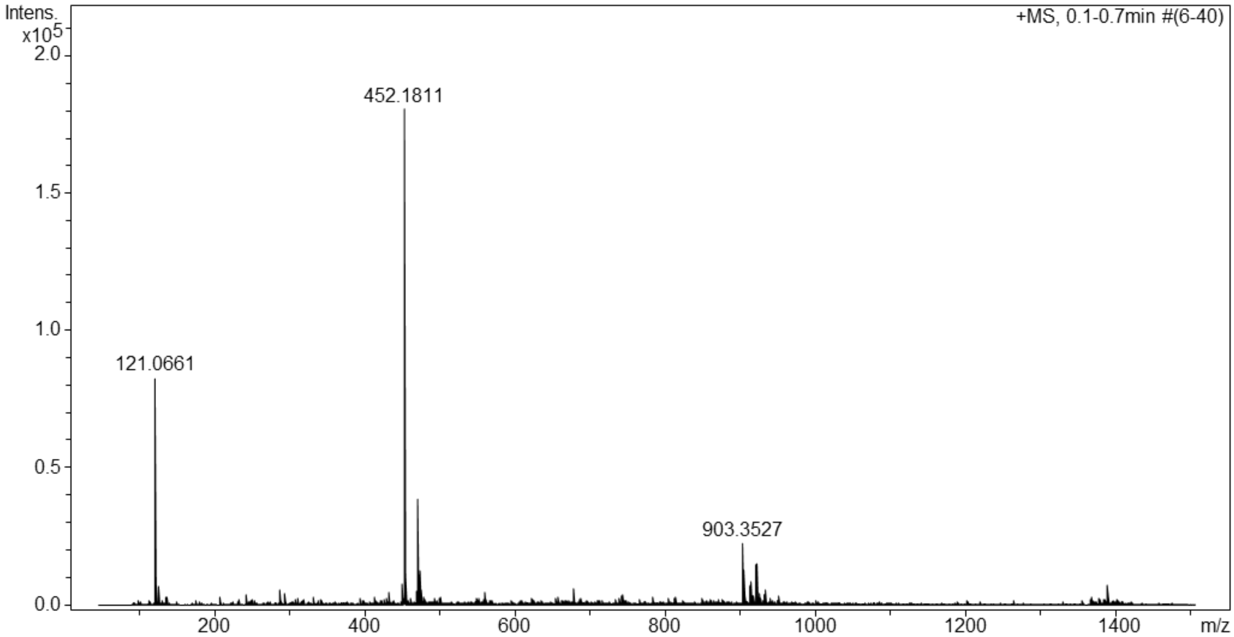

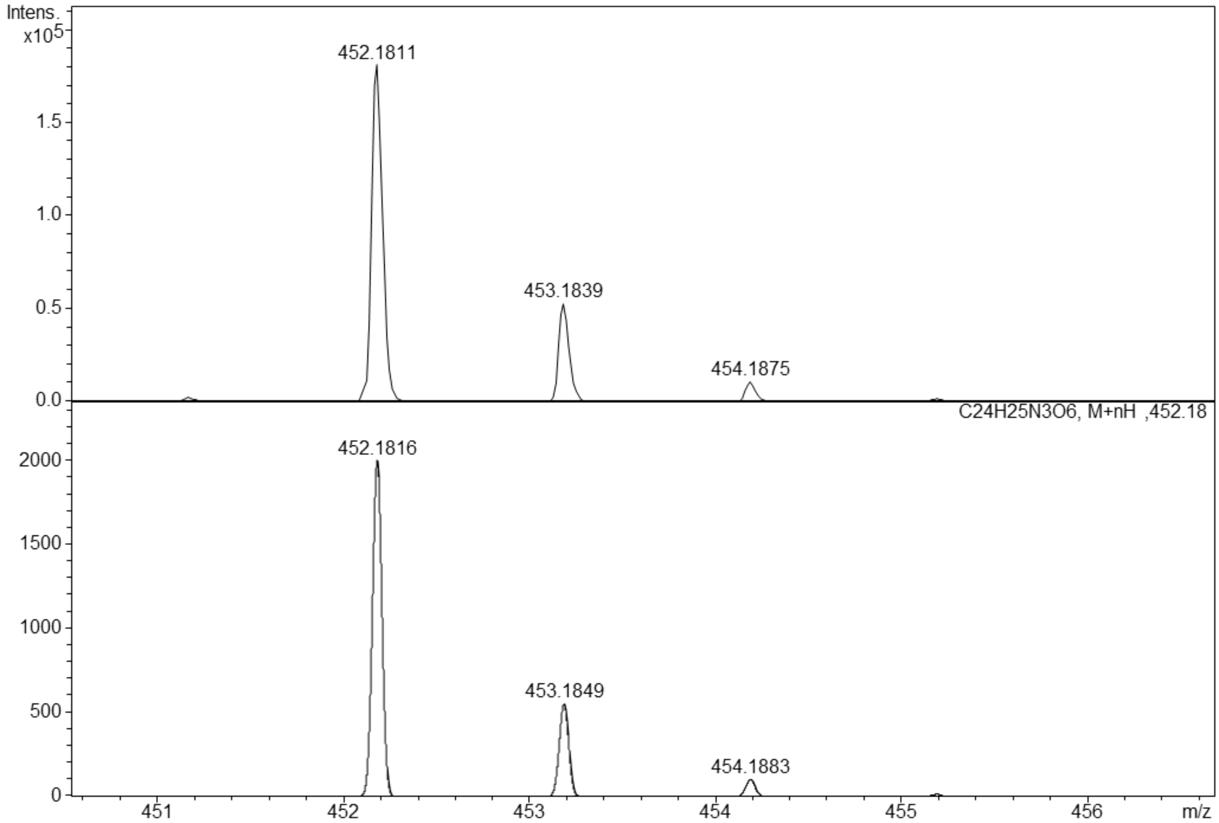

	Molecular formula	Calculated	Found
**$[M+H]^+$**	$C_{24}H_{26}N_3O_6$	452.1816	452.1811
**$[2M+H]^+$**	$(C_{24}H_{25}N_3O_6)_2$	903.3559	903.3527

Methyl 8-(4-methoxybenzyl)-5-methyl-4-methylene-7-(4-nitrophenyl)-2-oxo-3,8-diazabicyclo[3.2.1]octane-6-carboxylate (20ea) and methyl 8-(4-methoxybenzyl)-1-methyl-2-methylene-7-(4-nitrophenyl)-4-oxo-3,8-diazabicyclo[3.2.1]octane-6-carboxylate (20eb)

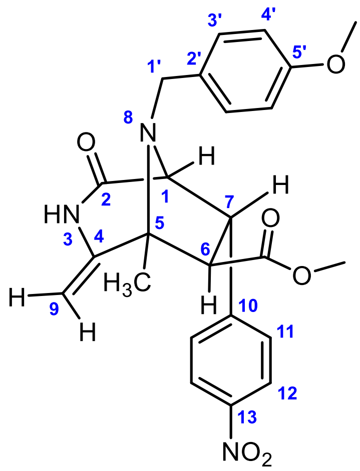

20ea

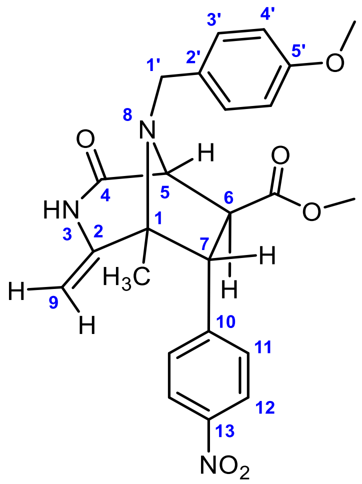

20eb

^{1}H-NMR (400 MHz, $CDCl_3$), δ (ppm)

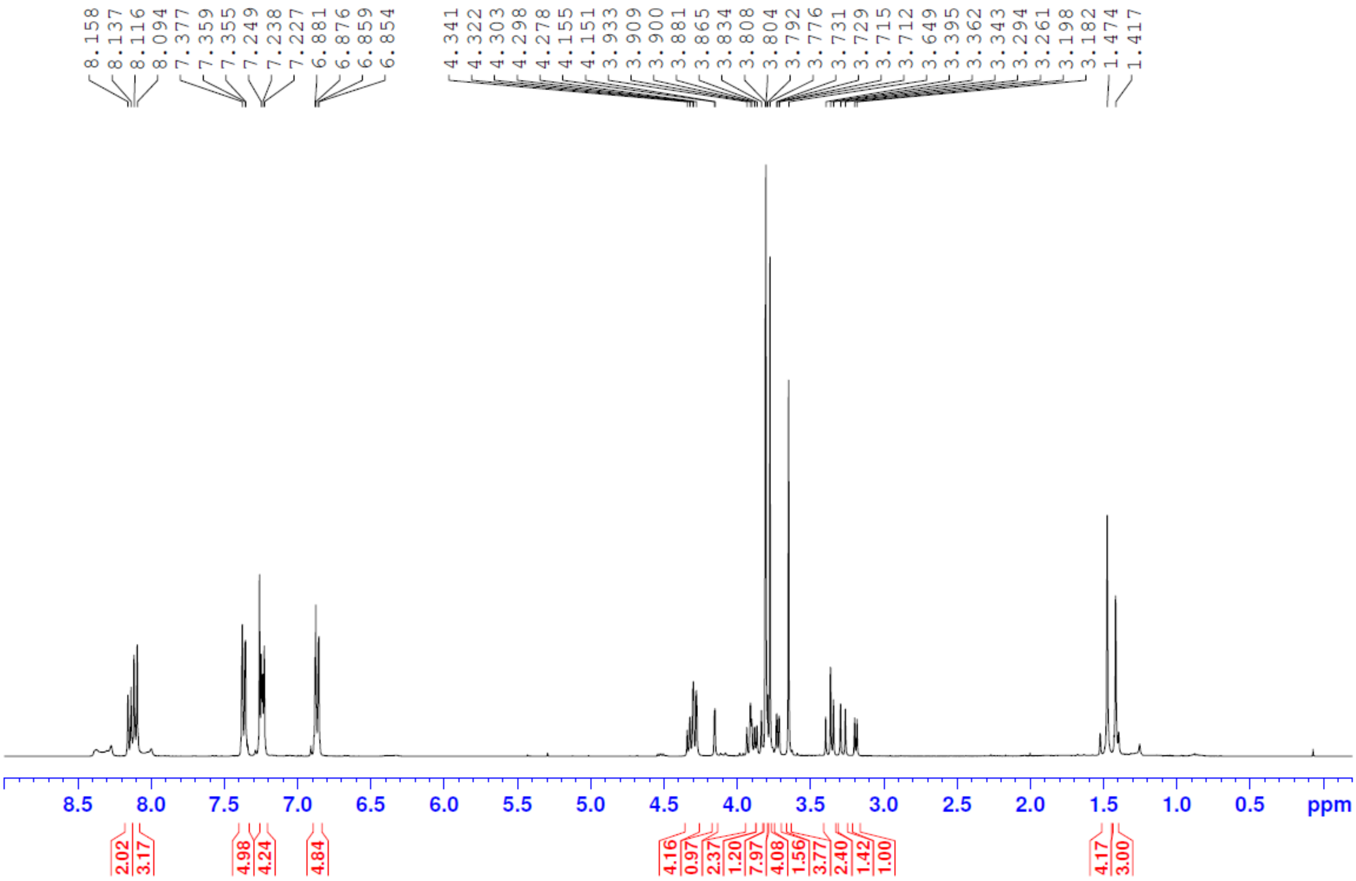

^{1}H-NMR (400 MHz, CDCl$_3$), δ (ppm) – Aliphatic zoom

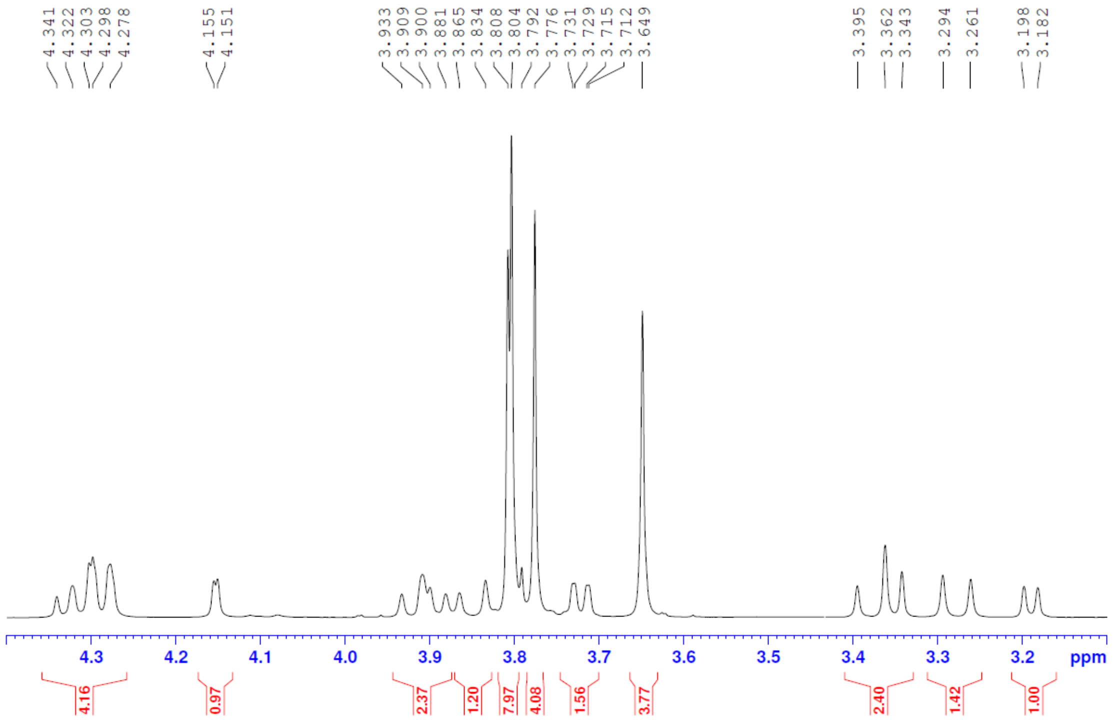

^{13}C{^{1}H}-NMR and DEPT135 (100 MHz, CDCl$_3$), δ (ppm)

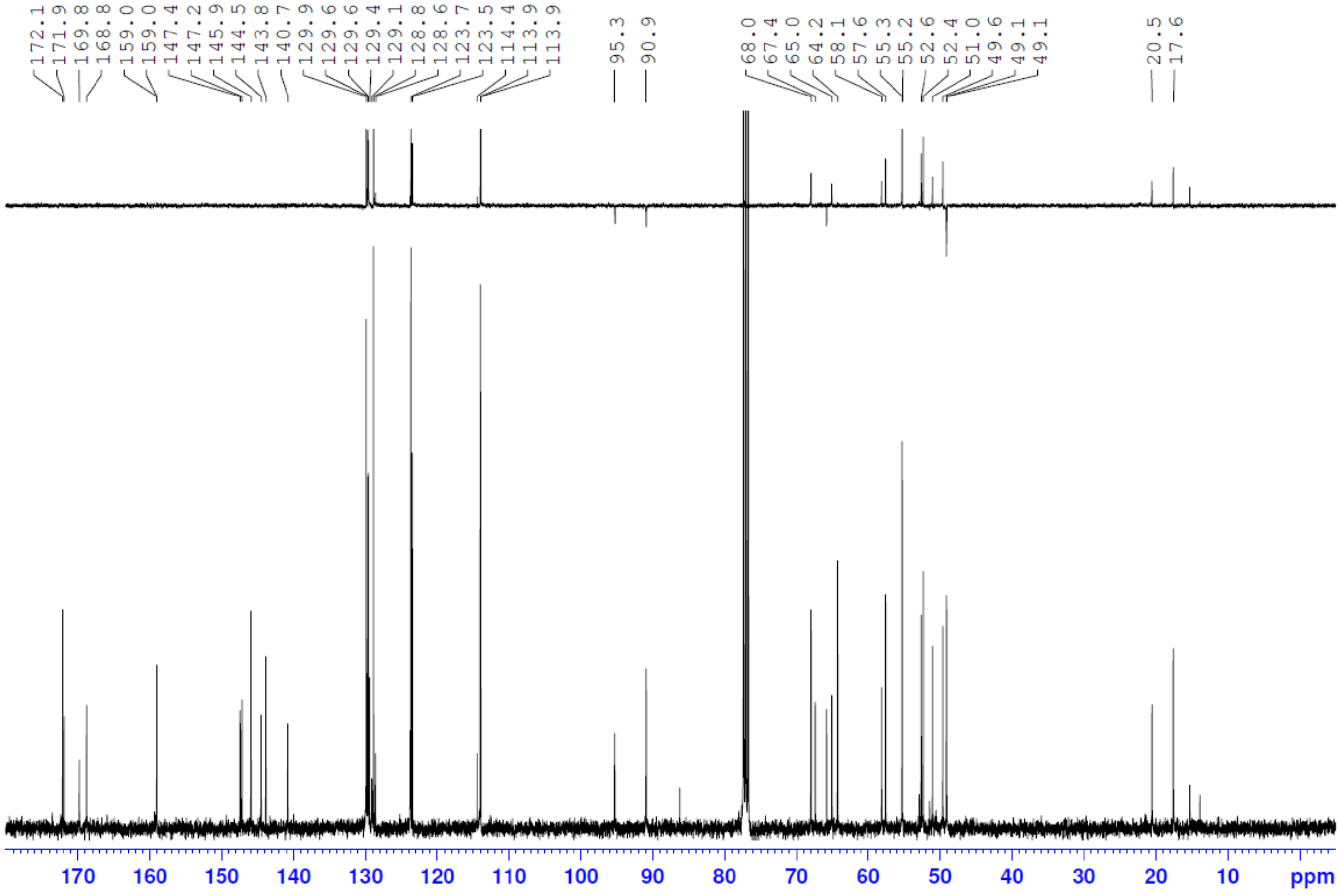

$^{13}C\{^1H\}$-NMR and DEPT135 (100 MHz, $CDCl_3$), δ (ppm) – Aliphatic zoom

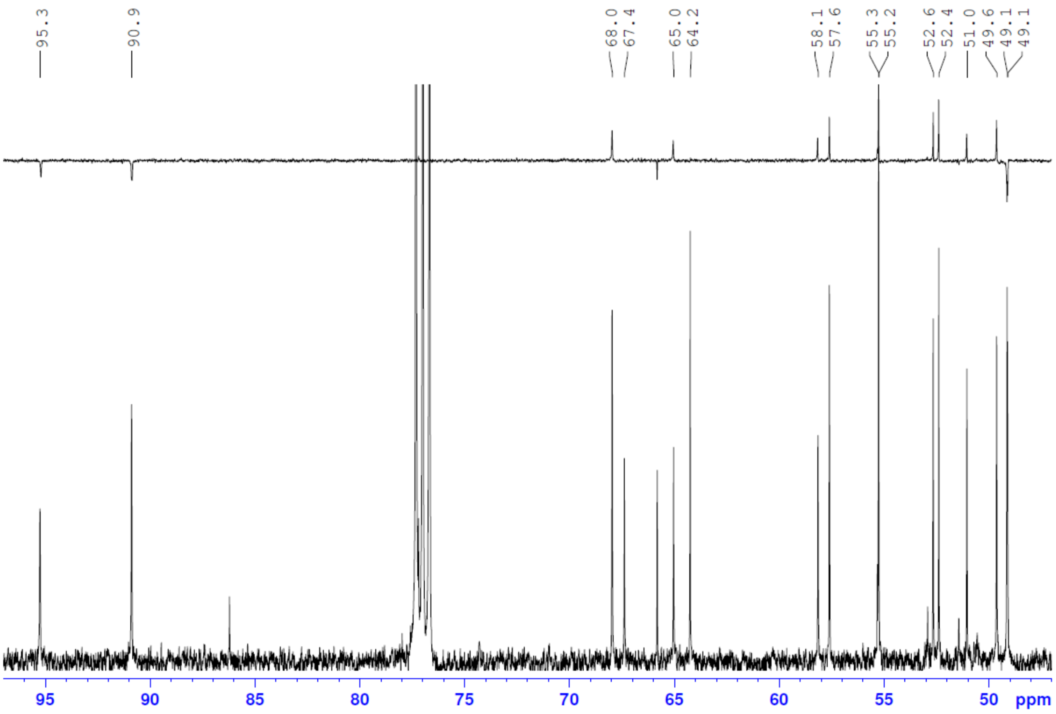

$^{13}C\{^1H\}$-NMR and DEPT135 (100 MHz, $CDCl_3$), δ (ppm) – Aromatic zoom

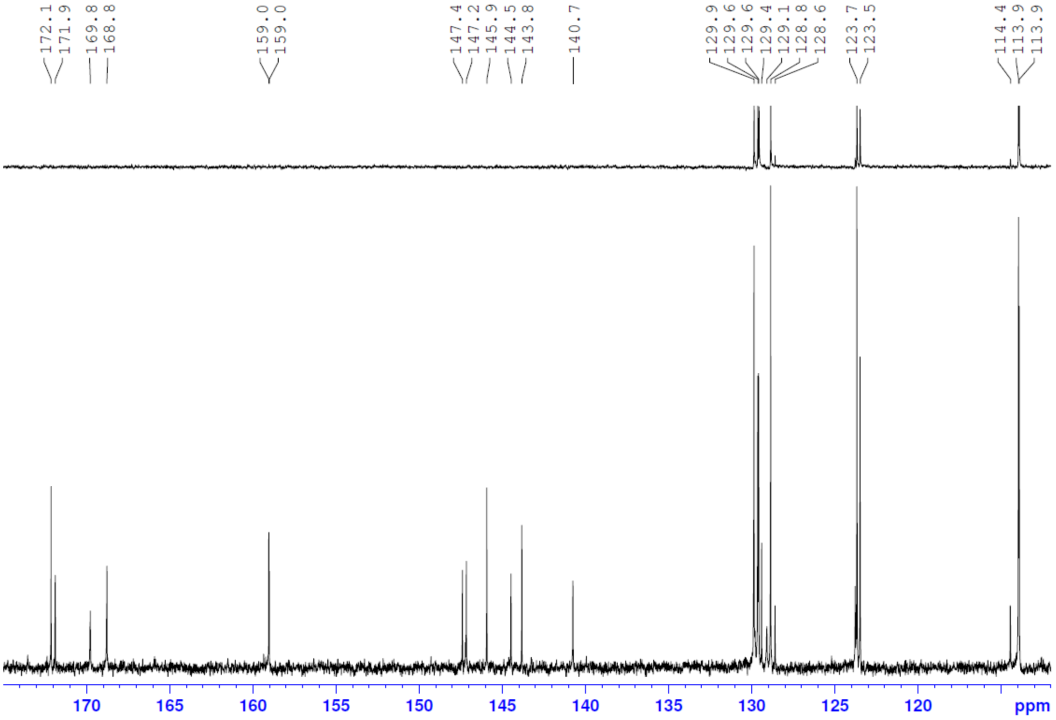

COSY ^1H-^1H ($CDCl_3$), δ (ppm) – Aliphatic zoom

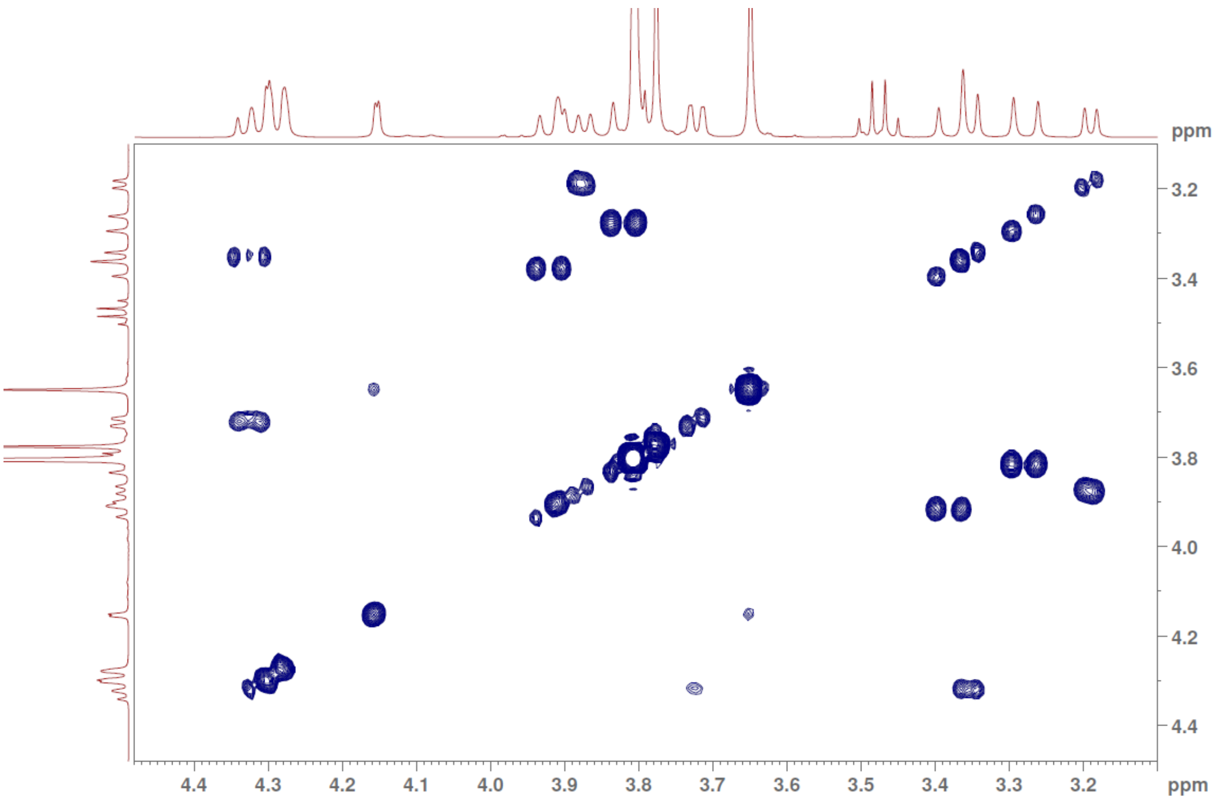

COSY ^1H-^1H ($CDCl_3$), δ (ppm) – Aromatic Zoom

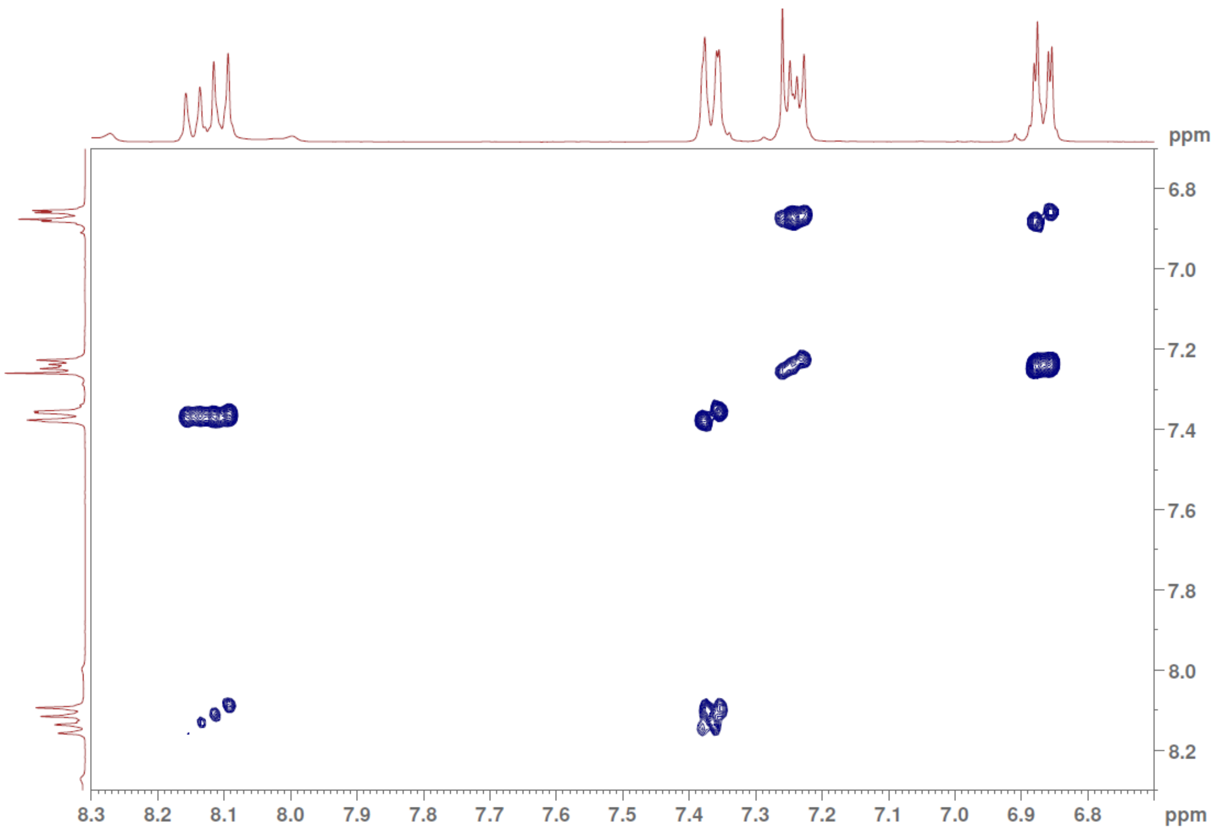

HSQC ^{1}H-^{13}C ($CDCl_3$), δ (ppm)

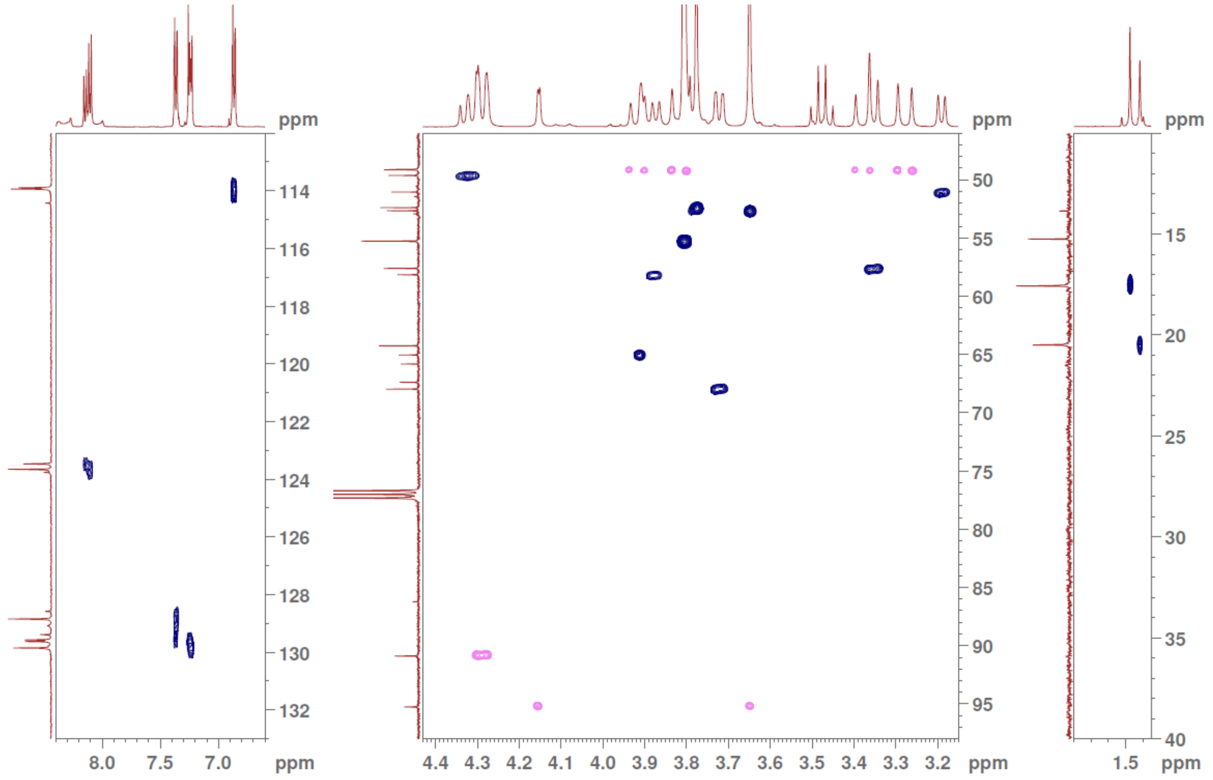

HMBC ^{1}H-^{13}C ($CDCl_3$), δ (ppm)

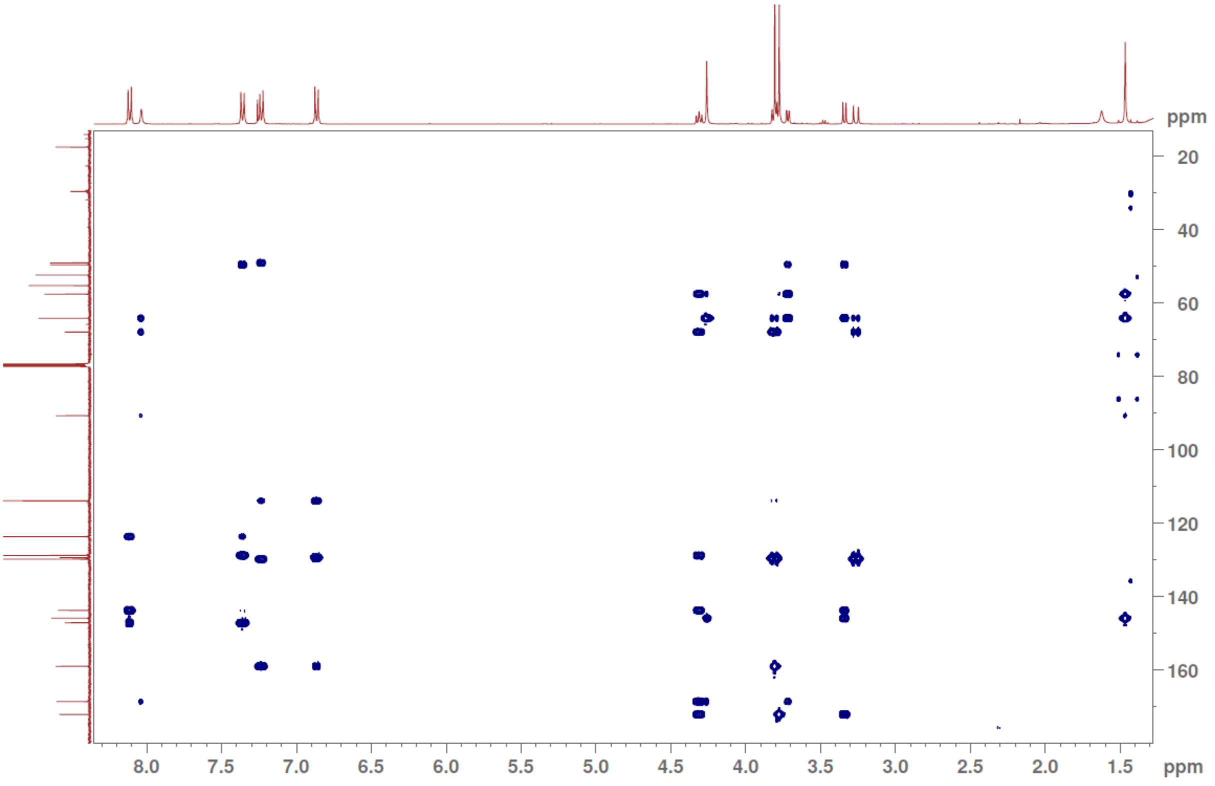

Methyl 5-(4-methoxybenzyl)-1-methyl-6-methylene-3-oxo-7-phenyl-2,5-diazabicyclo[2.2.2]octane-7-carboxylate (21f)

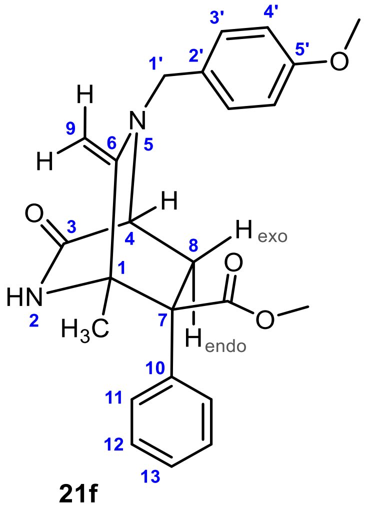

21f

^{1}H-NMR (400 MHz, CDCl3), δ (ppm)

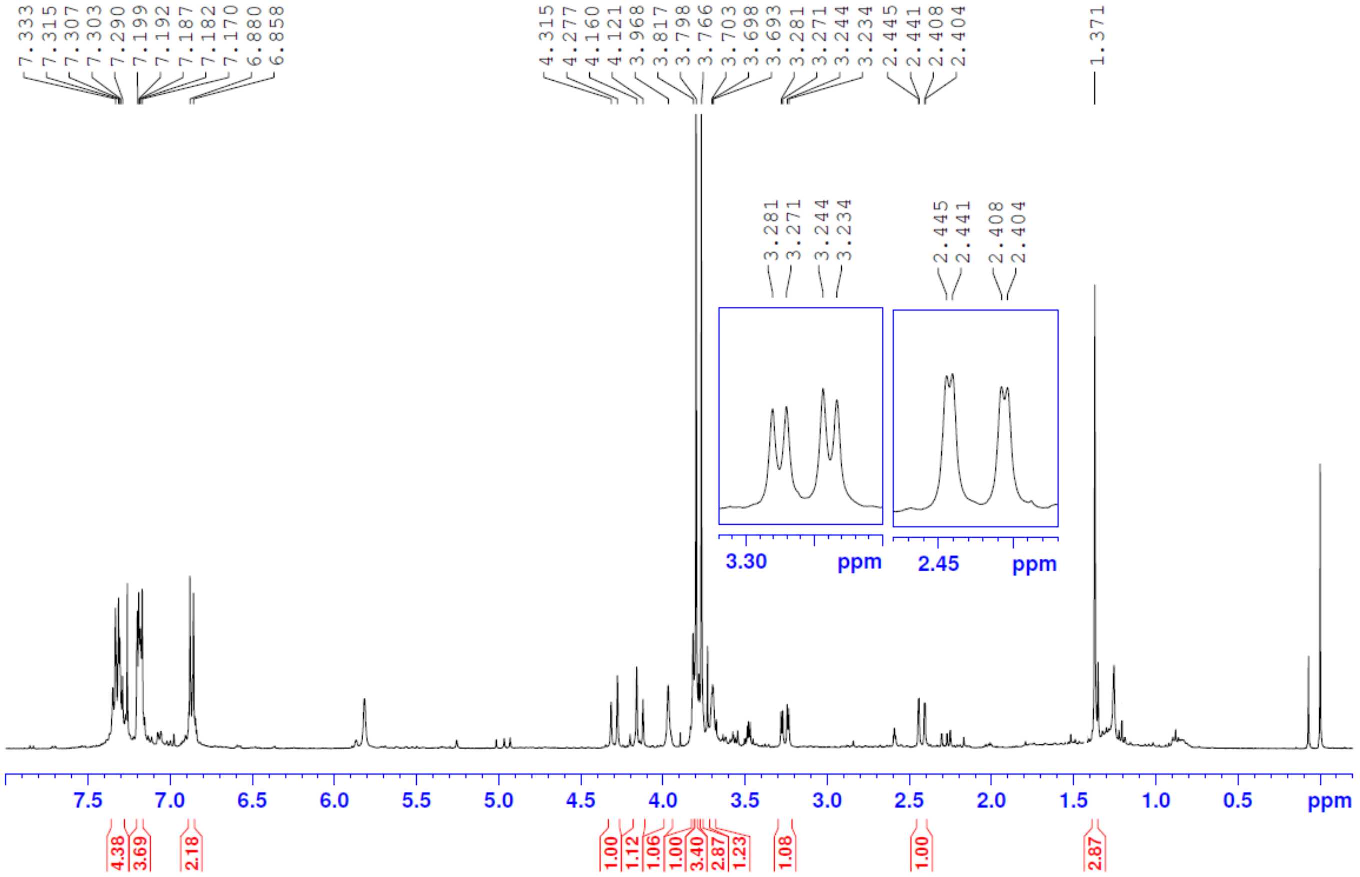

$^{13}C\{^{1}H\}$-NMR (100 MHz, $CDCl_3$), δ (ppm)

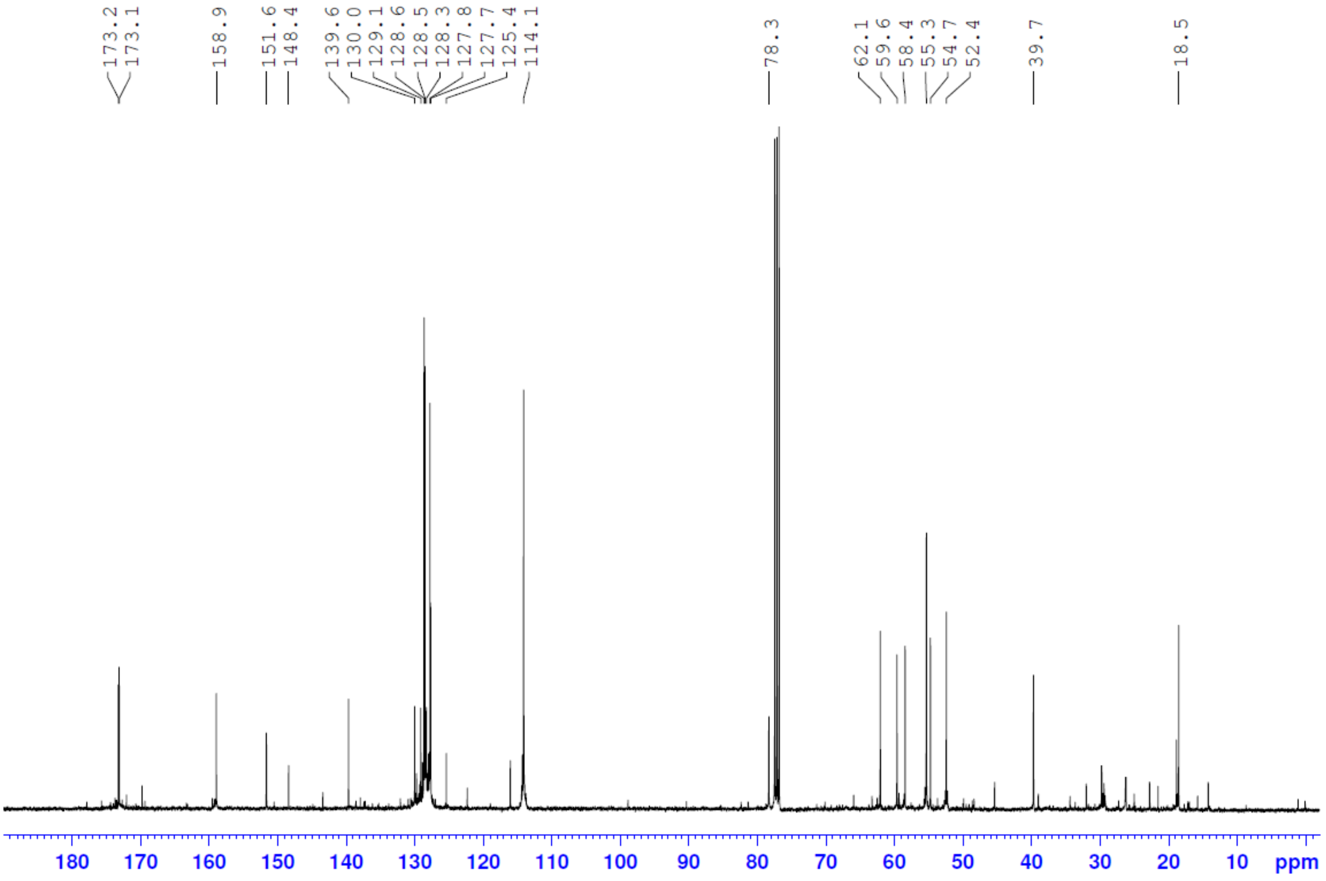

HSQC ^{1}H-^{13}C ($CDCl_3$), δ (ppm)

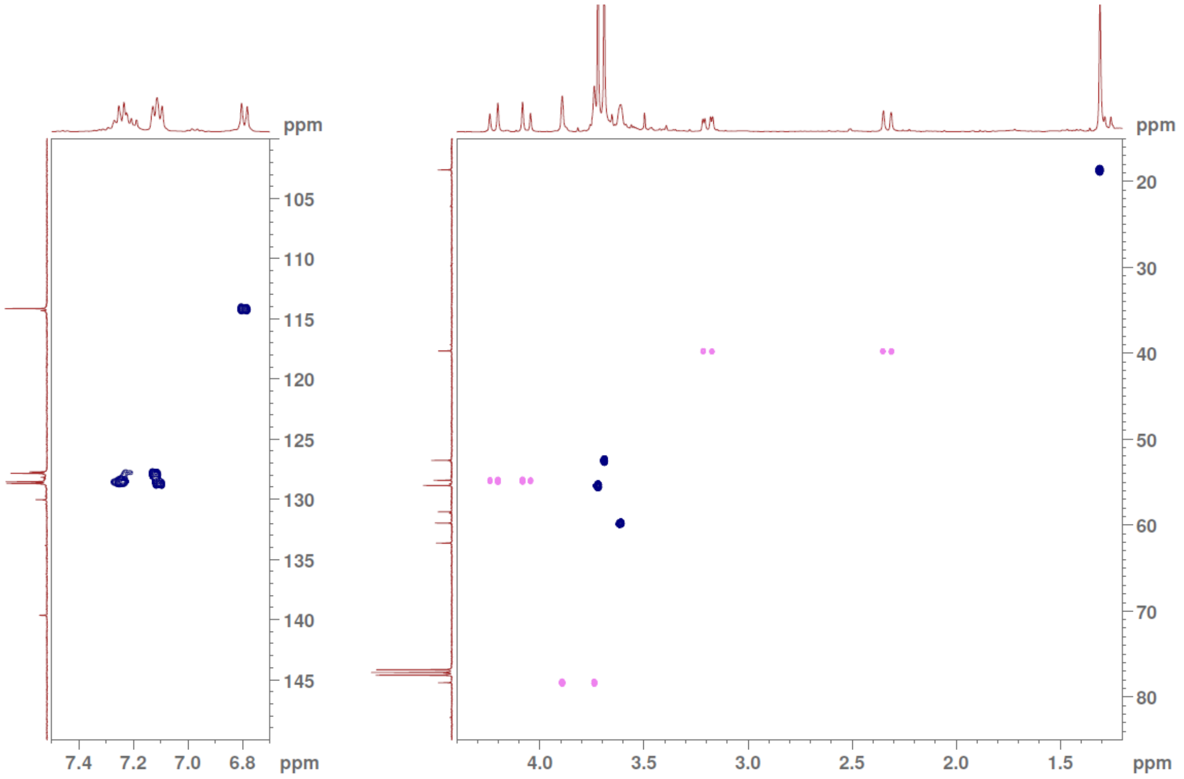

COSY ^{1}H-^{1}H ($CDCl_3$), δ (ppm)

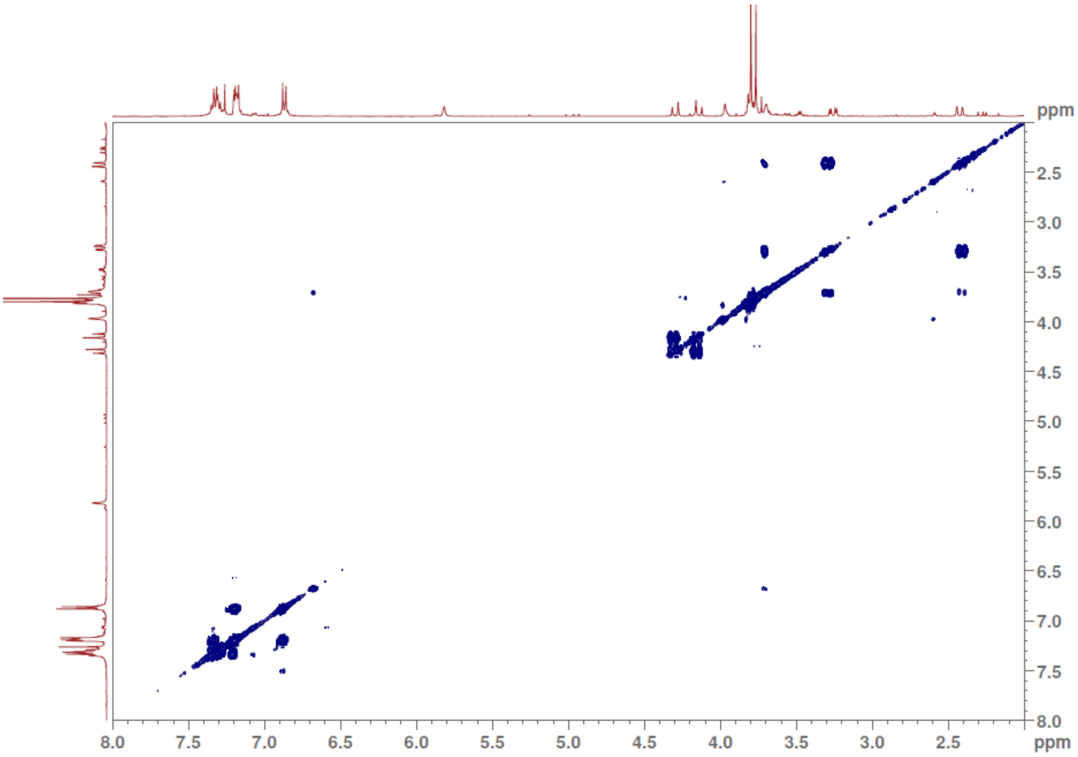

COSY ^{1}H-^{1}H ($CDCl_3$), δ (ppm) – Aliphatic Zoom

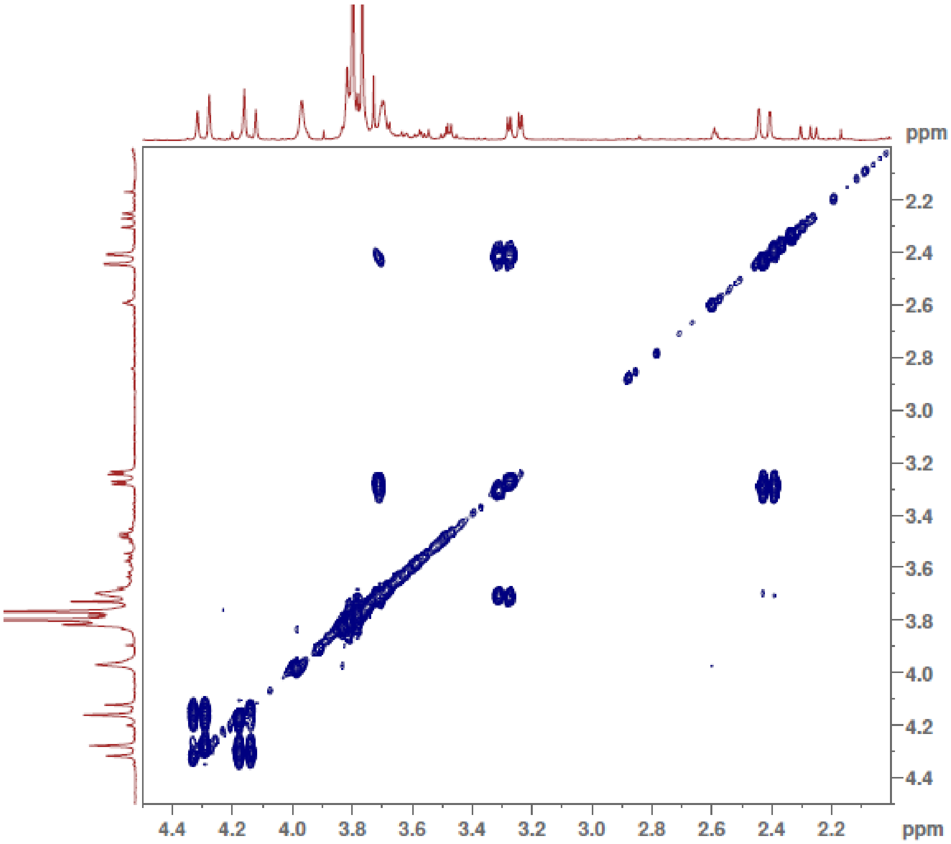

NOESY ^{1}H-^{1}H ($CDCl_3$), δ (ppm)

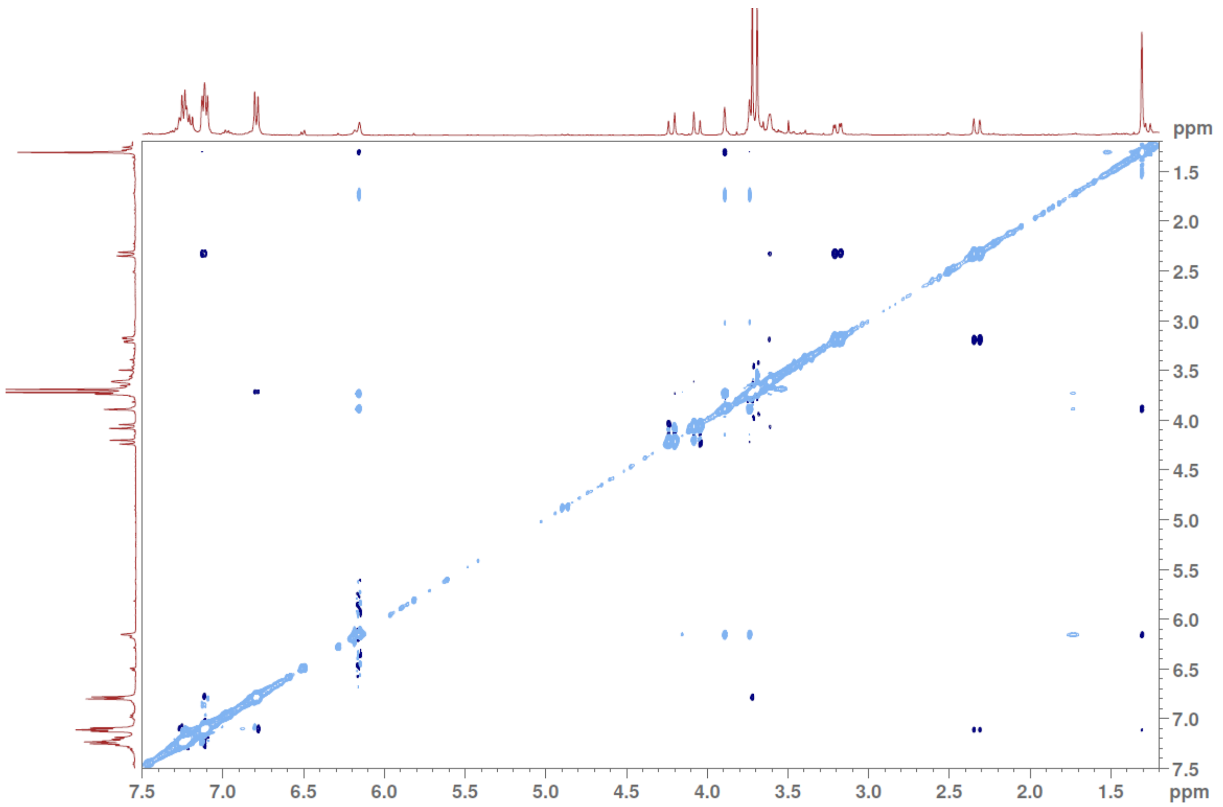

HMBC ^{1}H-^{13}C ($CDCl_3$), δ (ppm)

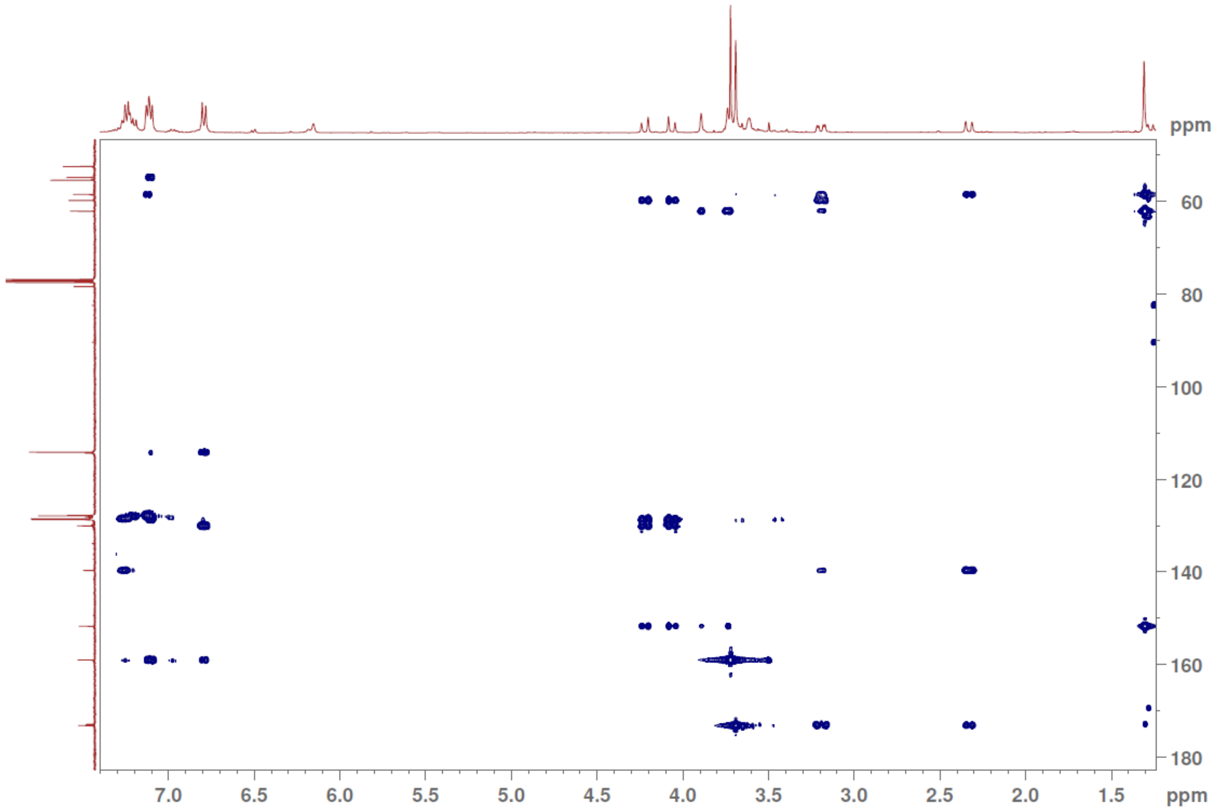

FT-IR (neat), ν (cm^{-1})

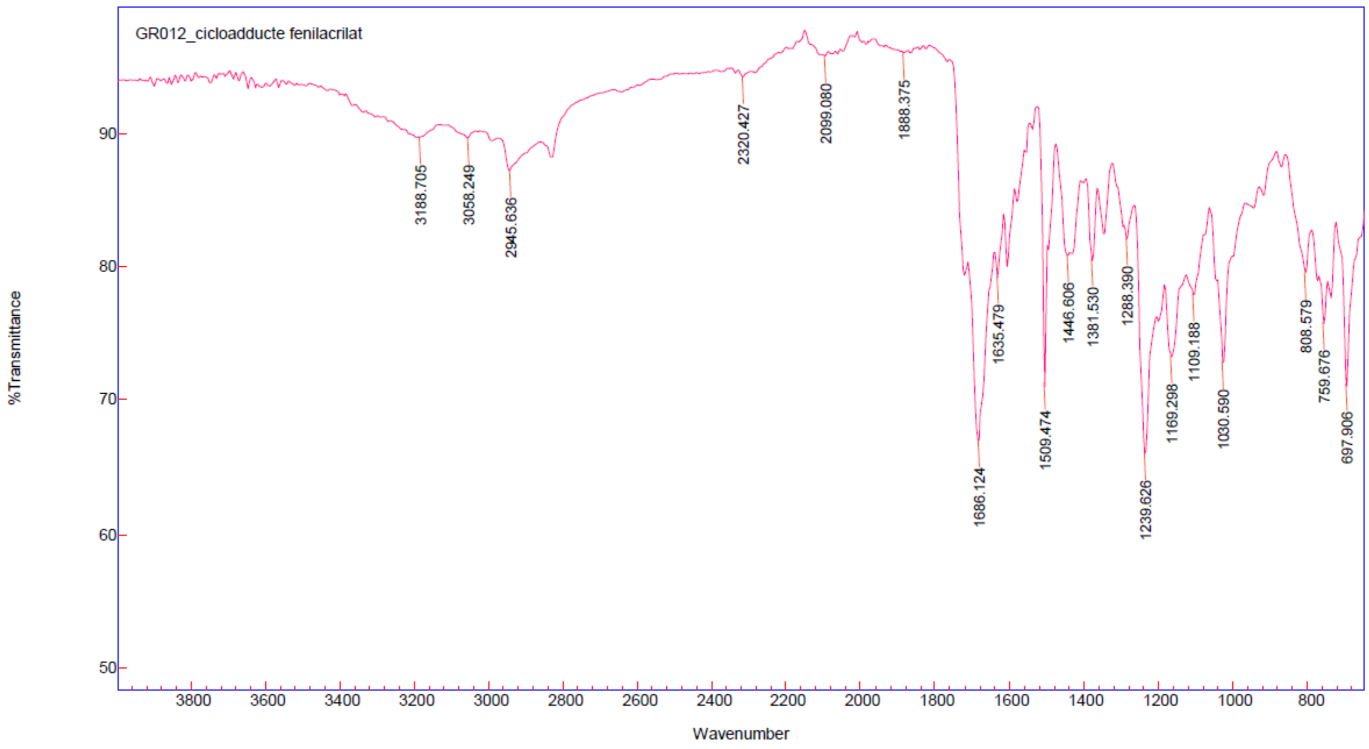

ESI-MS (*m/z*)

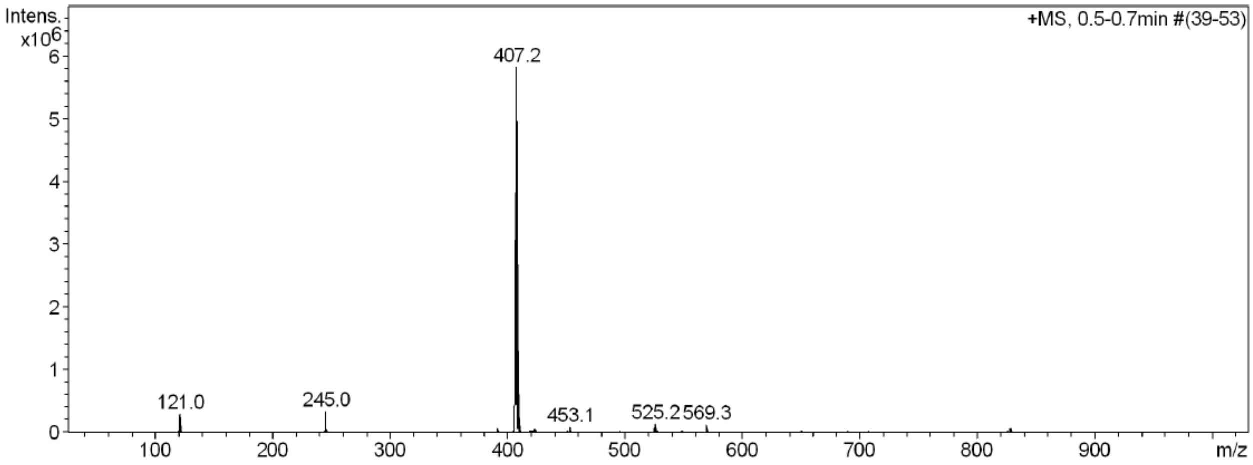

HRMS (*m/z*)

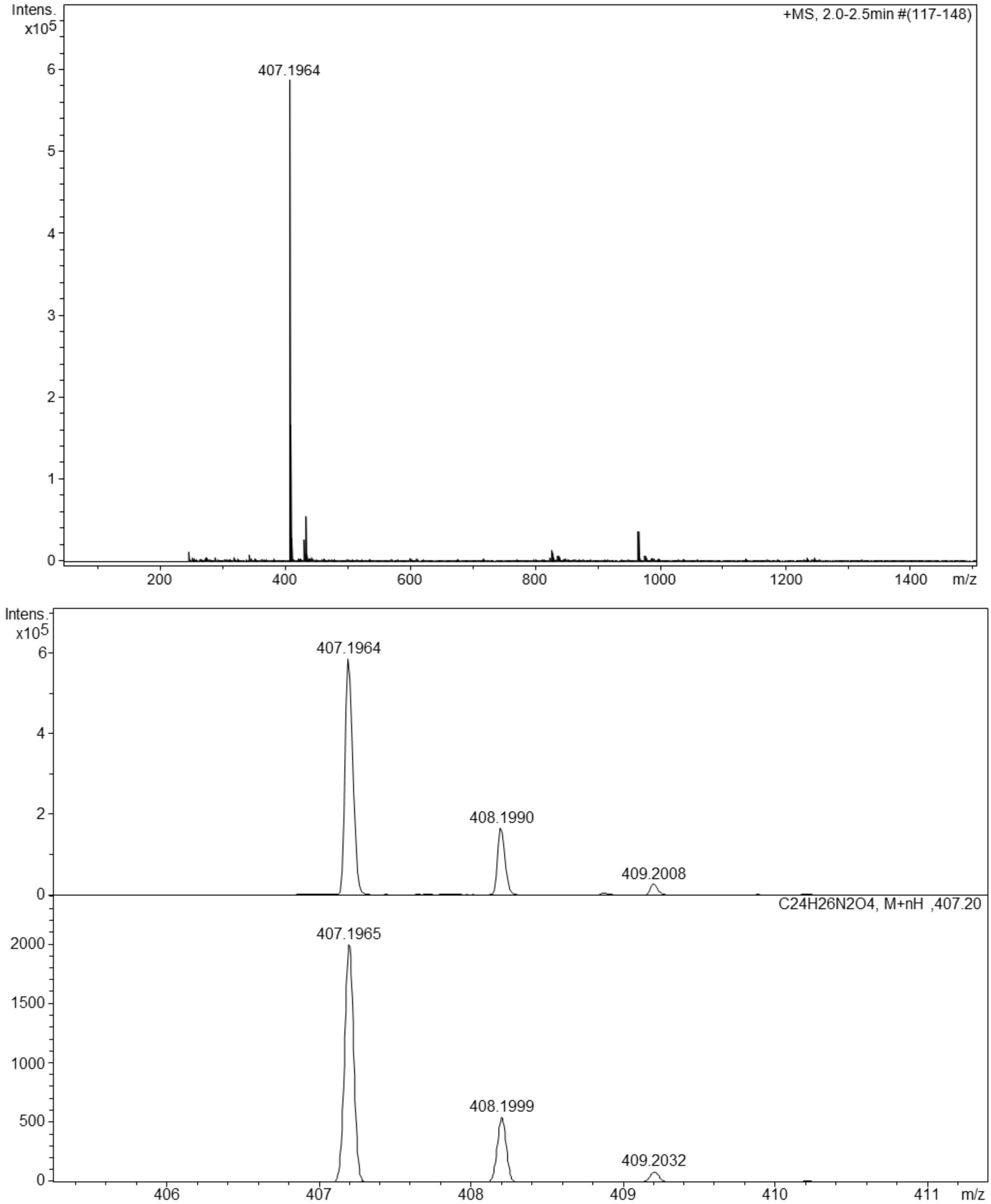

	Molecular formula	Calculated	Found
**[M+H]+**	$C_{24}H_{27}N_2O_4$	407.1965	407.1964

8-(4-Methoxybenzyl)-5-methyl-4-methylene-2-oxo-3,8-diazabicyclo[3.2.1]octane-6-carboxylic acid (22) and 8-(4-methoxybenzyl)-7,7a-dimethyl-1,3,4,4a-tetrahydro-3,7-epiminofuro[3,4-*b*]pyridine-2,5-dione (23)

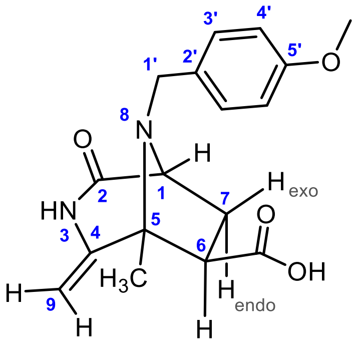

22

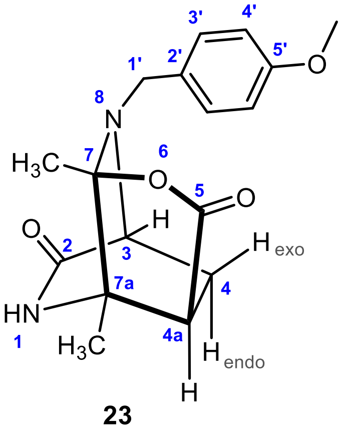

23

^{1}H-NMR (400 MHz, CDCl$_3$), δ (ppm)

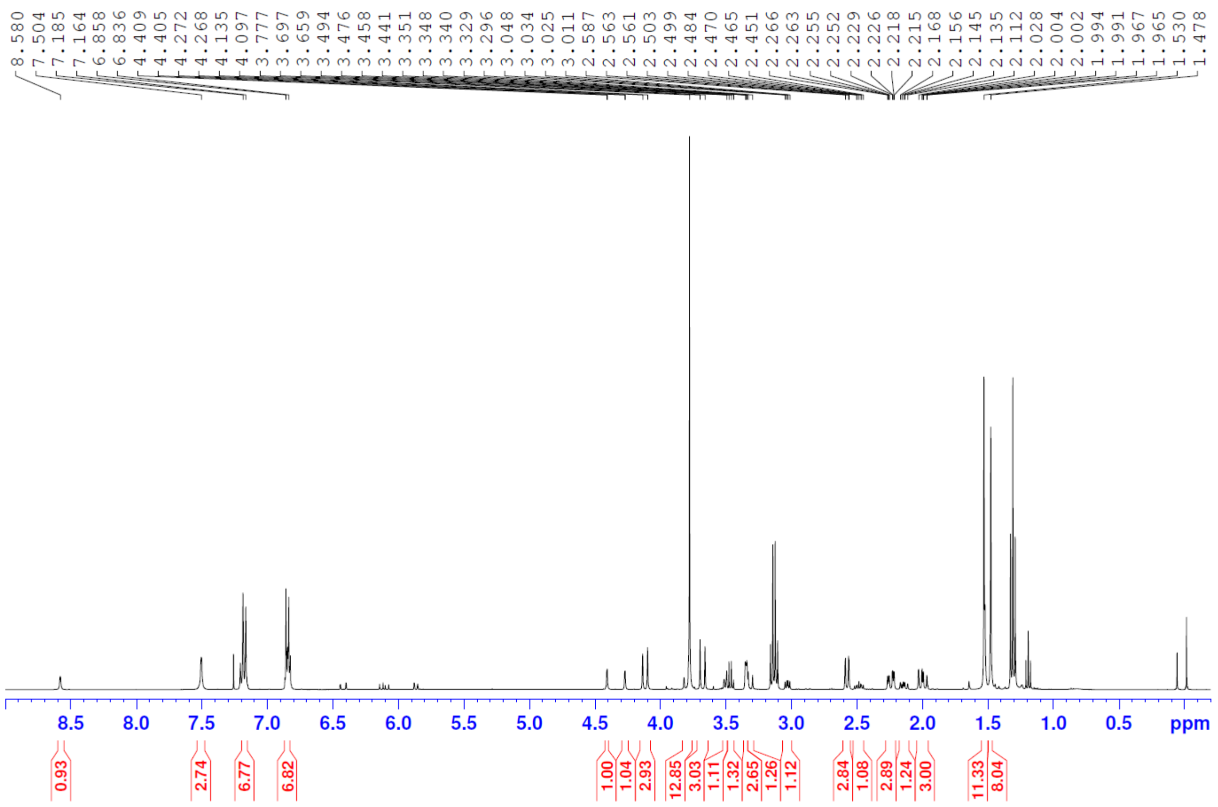

COSY ^1H-^1H ($CDCl_3$), δ (ppm)

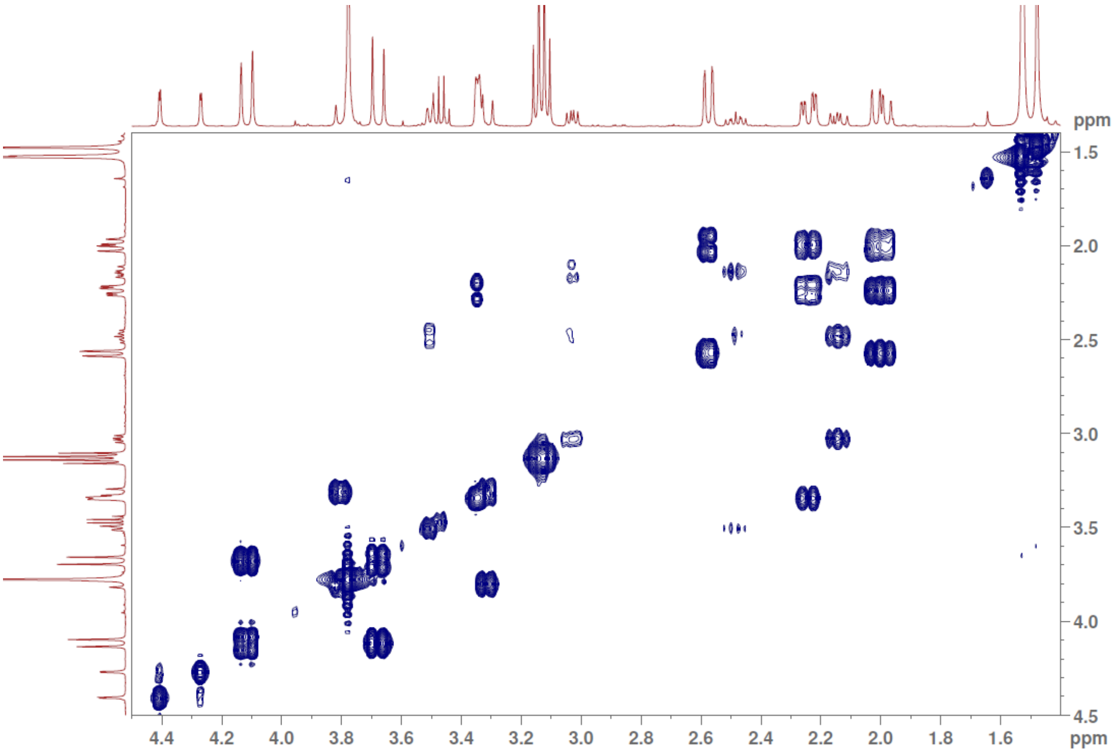

NOESY ^1H-^1H ($CDCl_3$), δ (ppm)

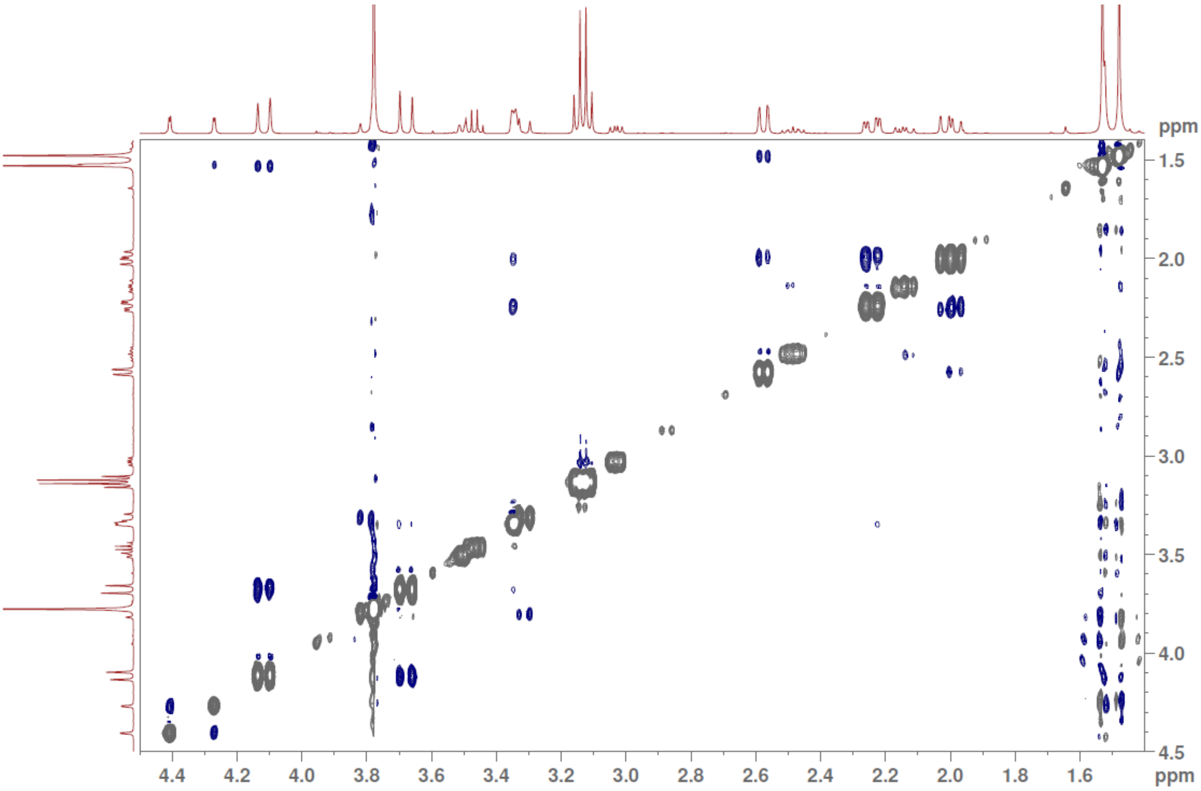

HSQC ^{1}H-^{13}C ($CDCl_3$), δ (ppm)

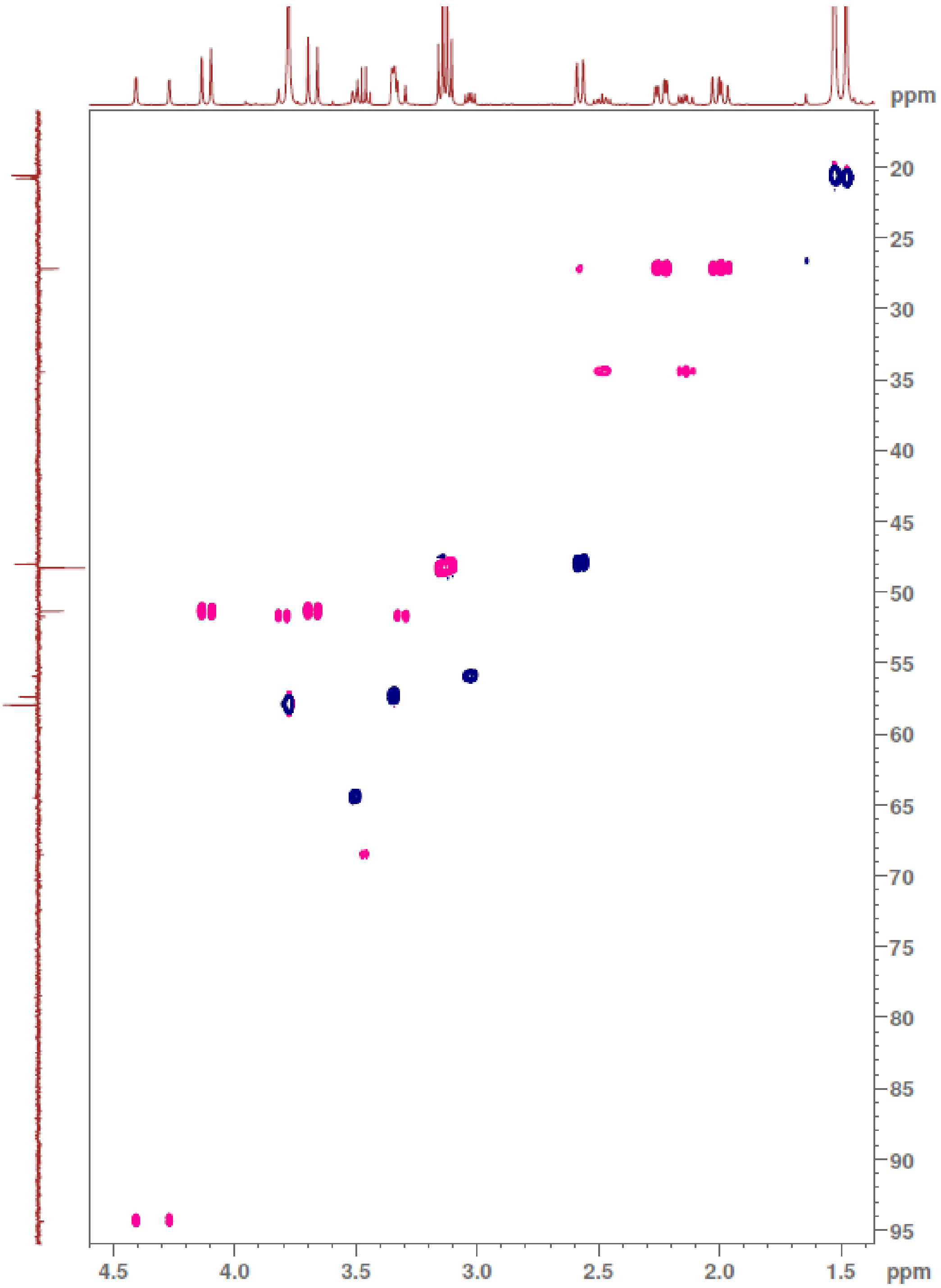

(–)-ESI-MS (*m*/*z*)

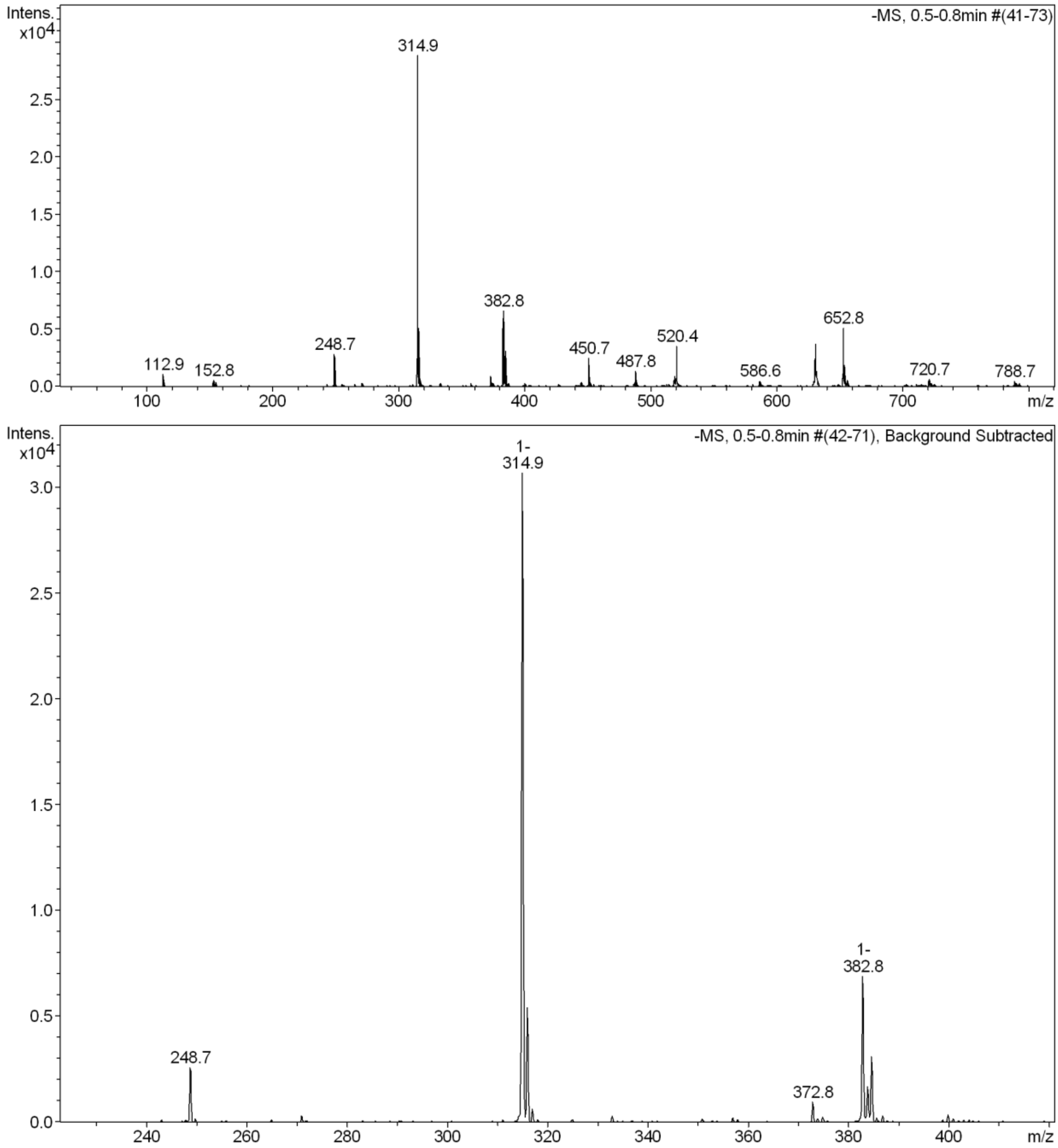

Intens.
x10^4
-MS, 0.5-0.8min #(41-73)
314.9
382.8
248.7
112.9
152.8
450.7
487.8
520.4
586.6
652.8
720.7
788.7
m/z
Intens.
x10^4
-MS, 0.5-0.8min #(42-71), Background Subtracted
1-
314.9
1-
382.8
248.7
372.8
m/z

8-(4-Methoxybenzyl)-7,7a-dimethyl-1,3,4,4a-tetrahydro-3,7-epiminofuro[3,4-*b*]pyridine-2,5-dione (23)

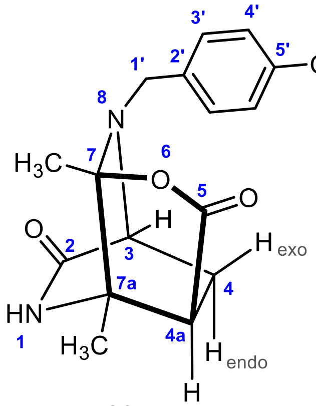

23

^{1}H-NMR (400 MHz, CDCl3), *δ* (ppm)

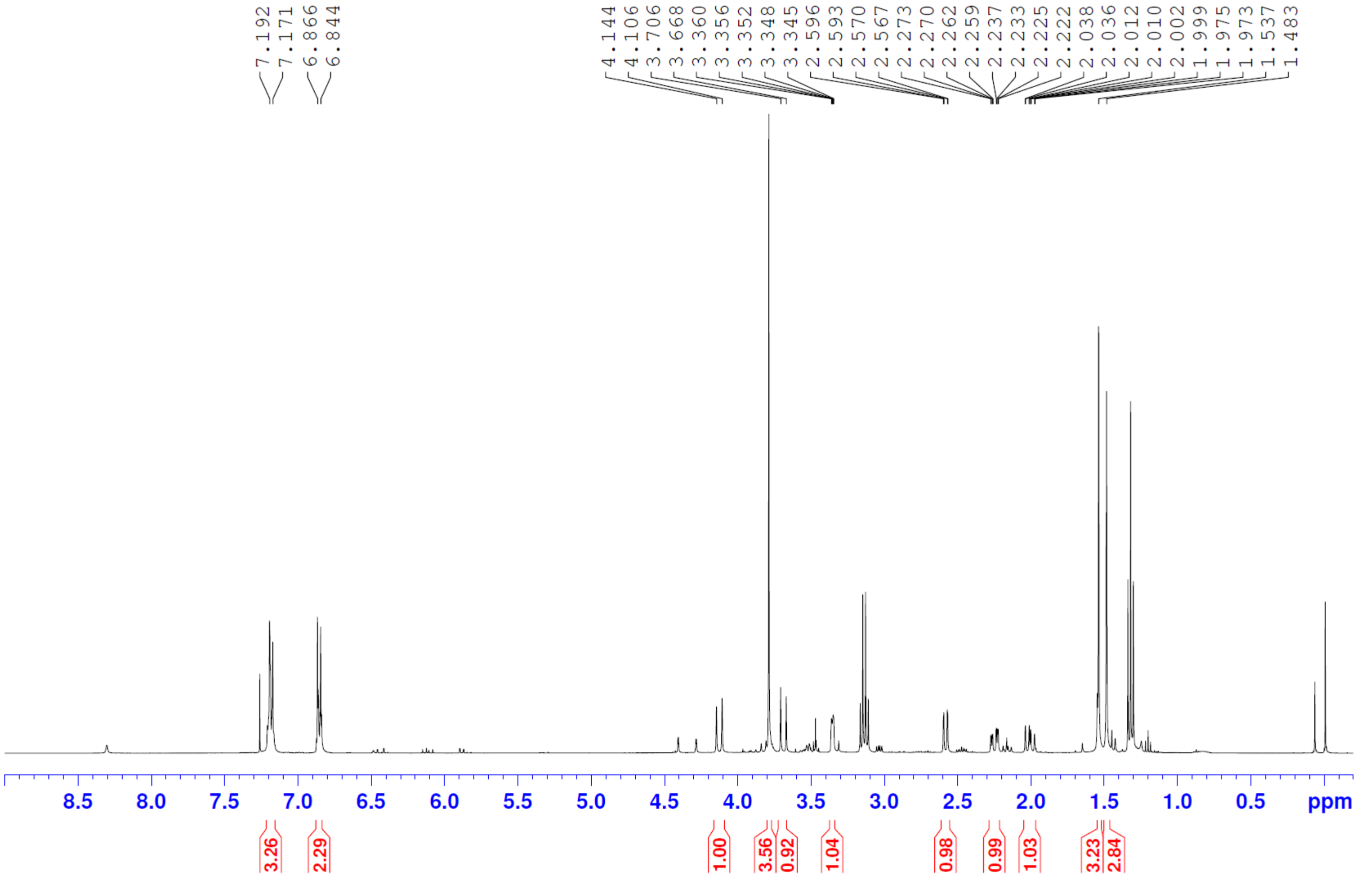

$^{13}C\{^{1}H\}$-NMR (100 MHz, CDCl$_3$), δ (ppm)

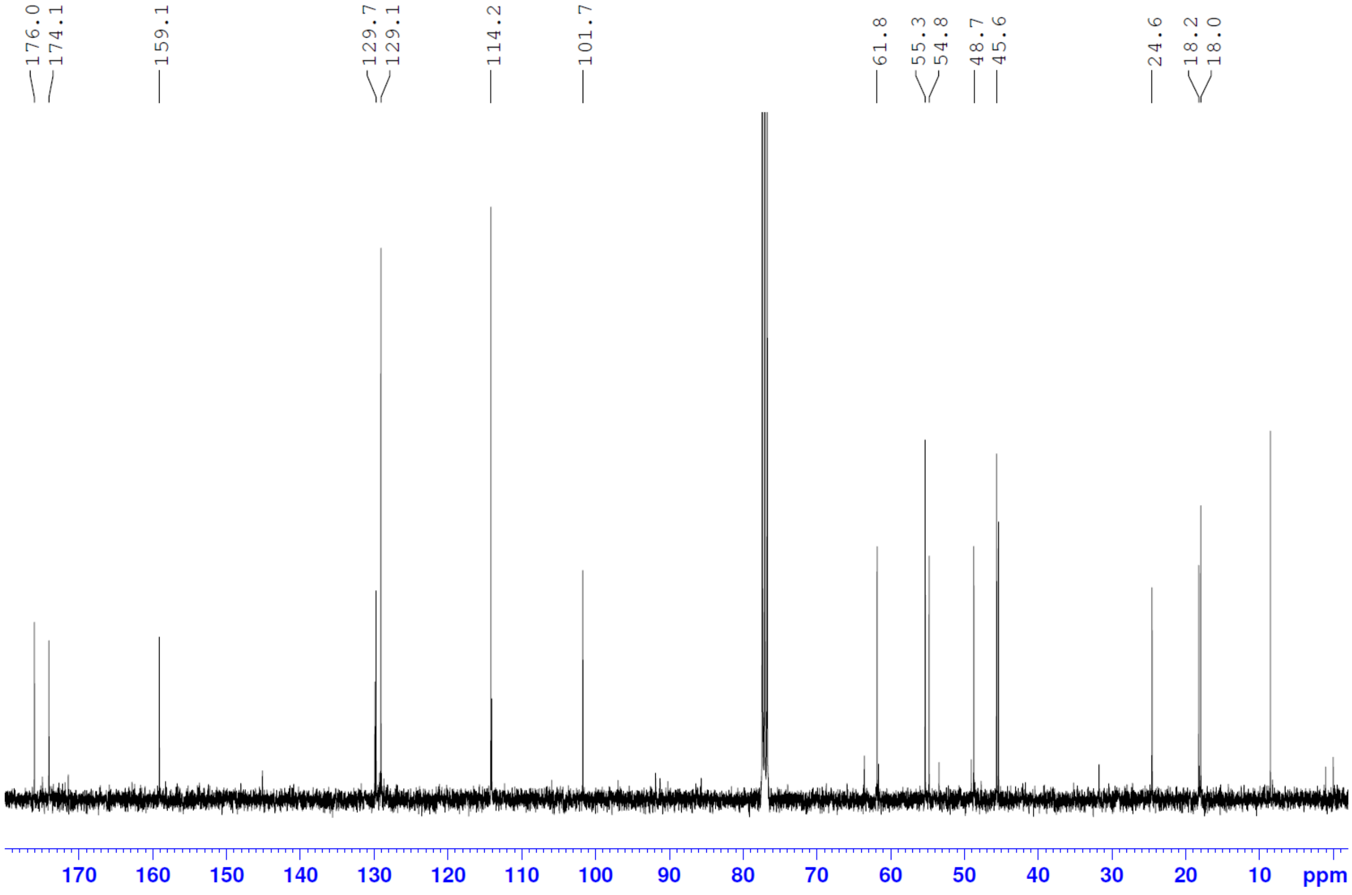

COSY ^{1}H-^{1}H (CDCl$_3$), δ (ppm)

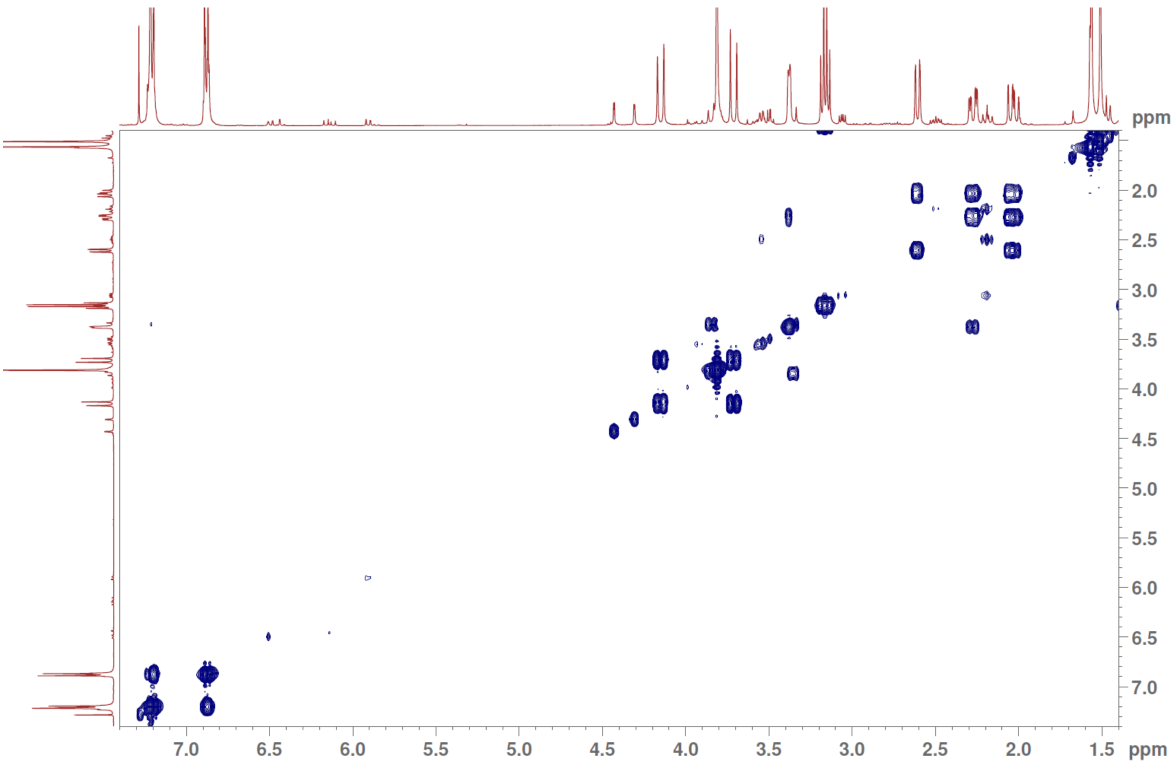

NOESY ^{1}H-^{1}H ($CDCl_3$), δ (ppm)

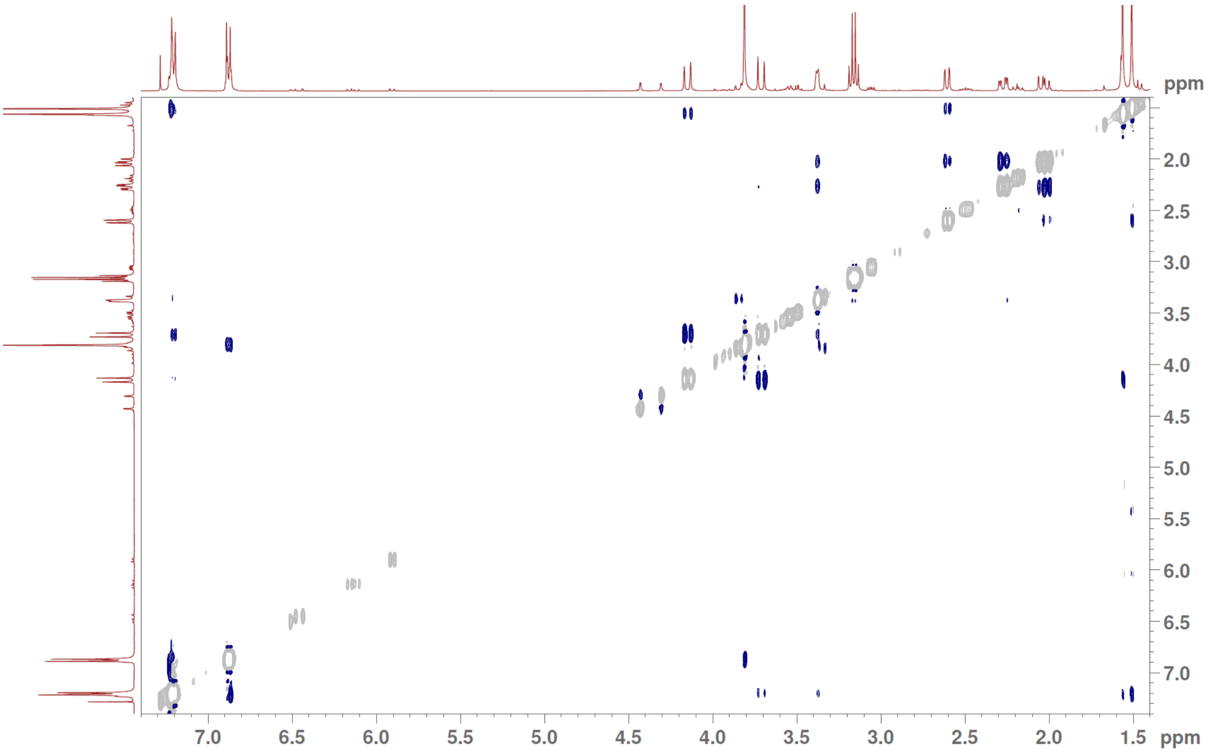

HSQC ^{1}H-^{13}C ($CDCl_3$), δ (ppm)

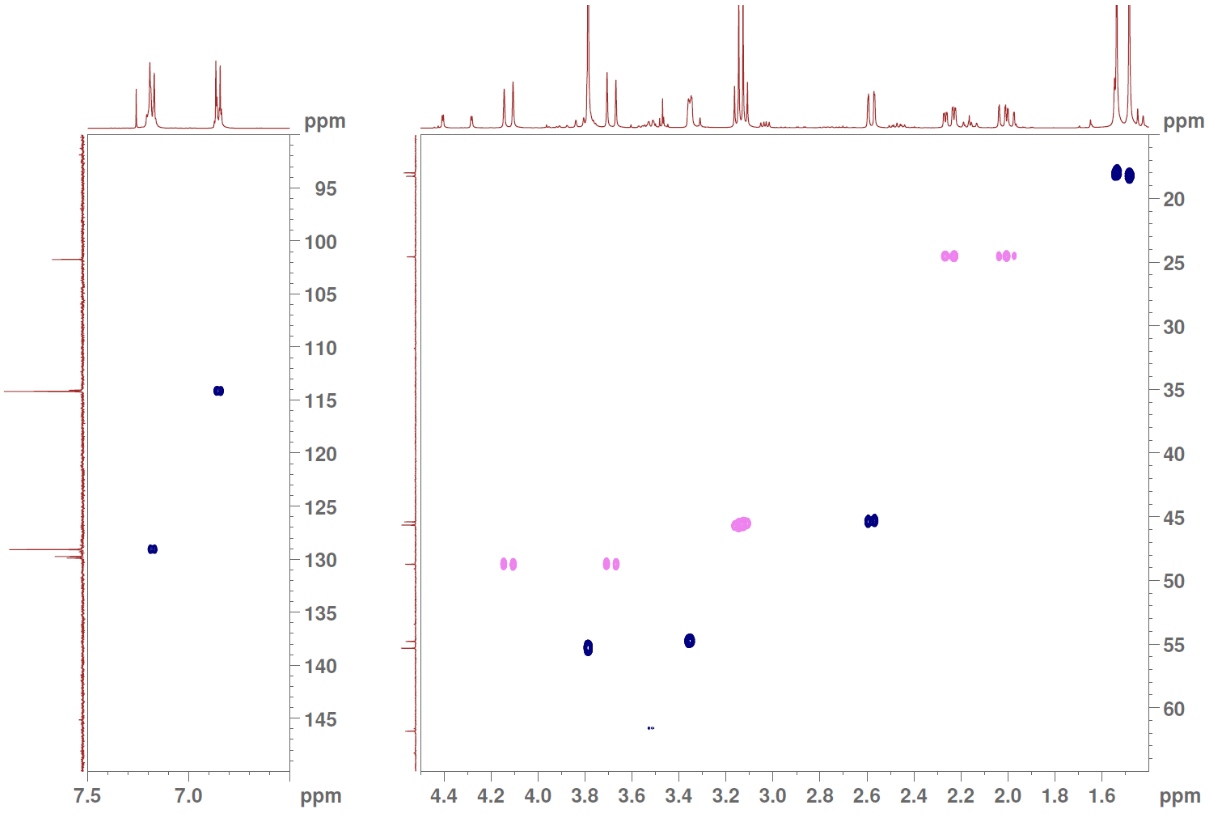

HMBC $^{1}H-^{13}C$ ($CDCl_3$), δ (ppm)

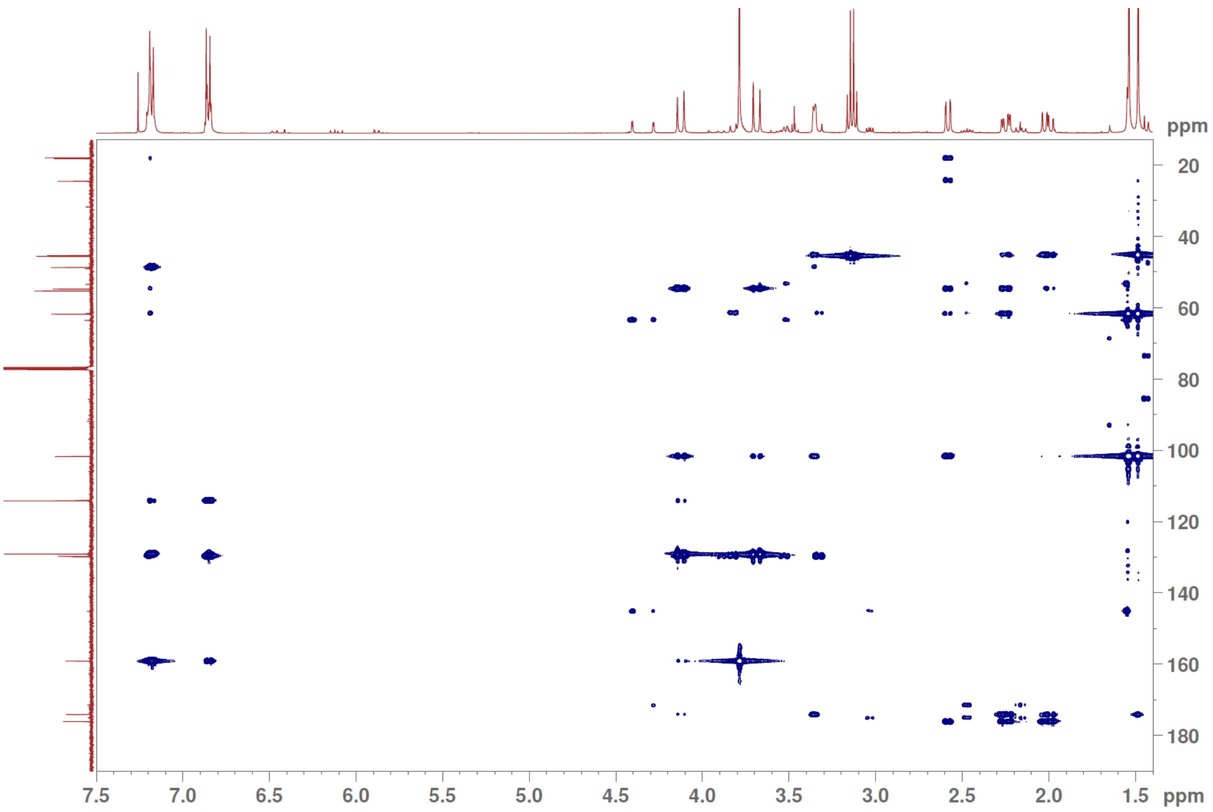

FT-IR (neat), ν (cm^{-1})

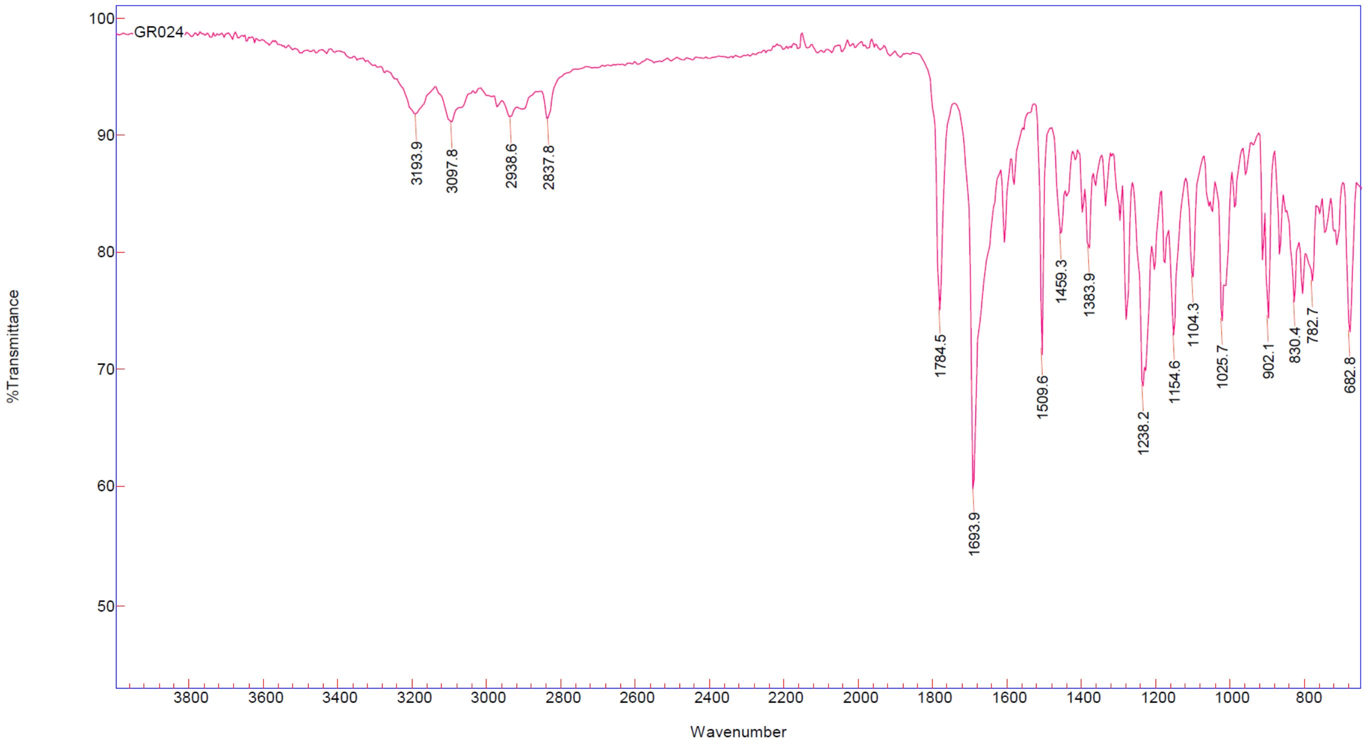

ESI-MS (*m/z*)

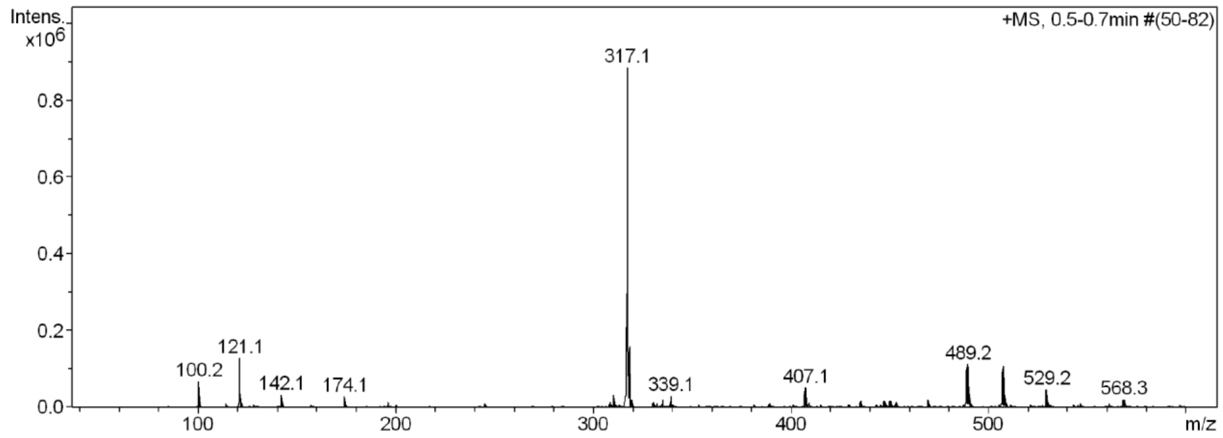

HRMS (*m/z*)

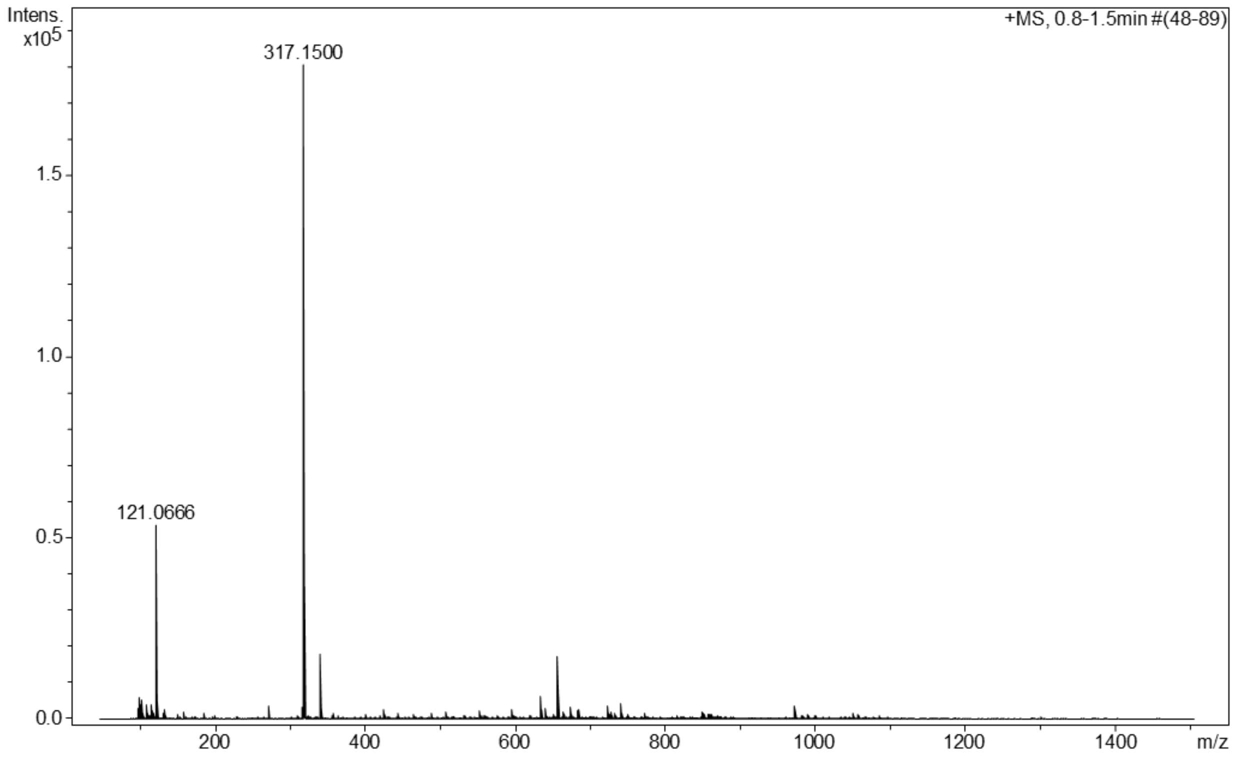

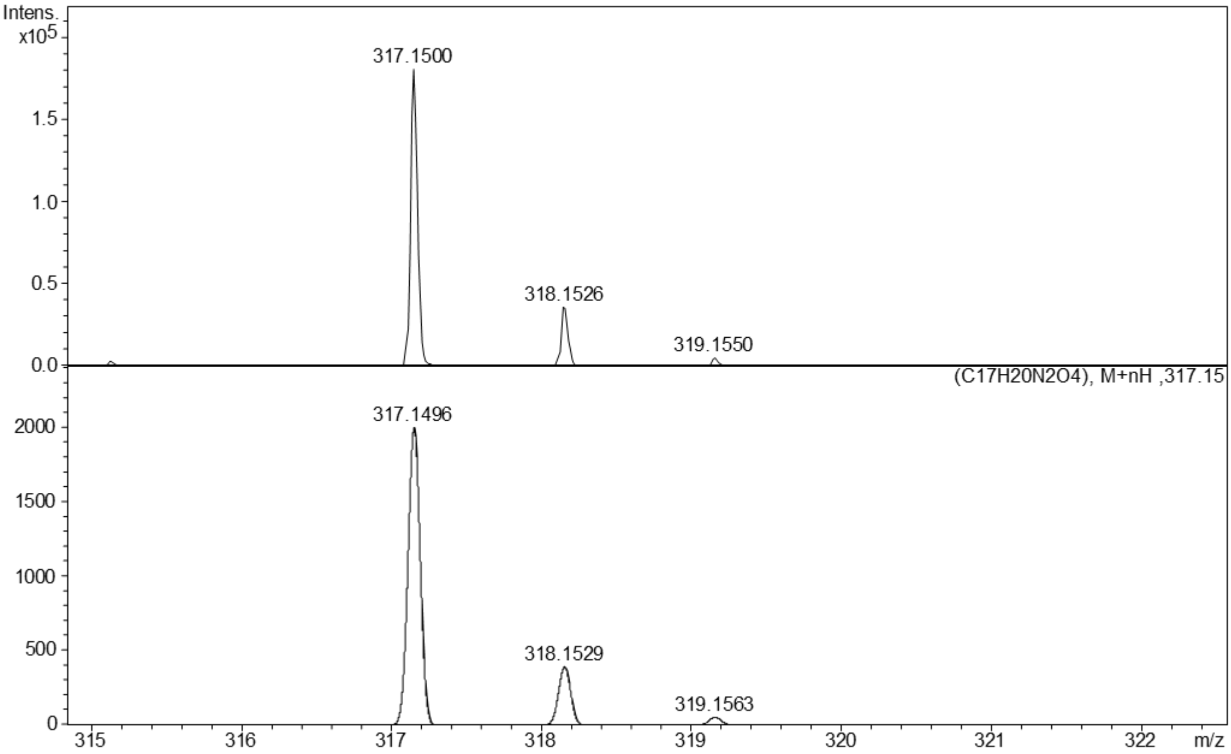

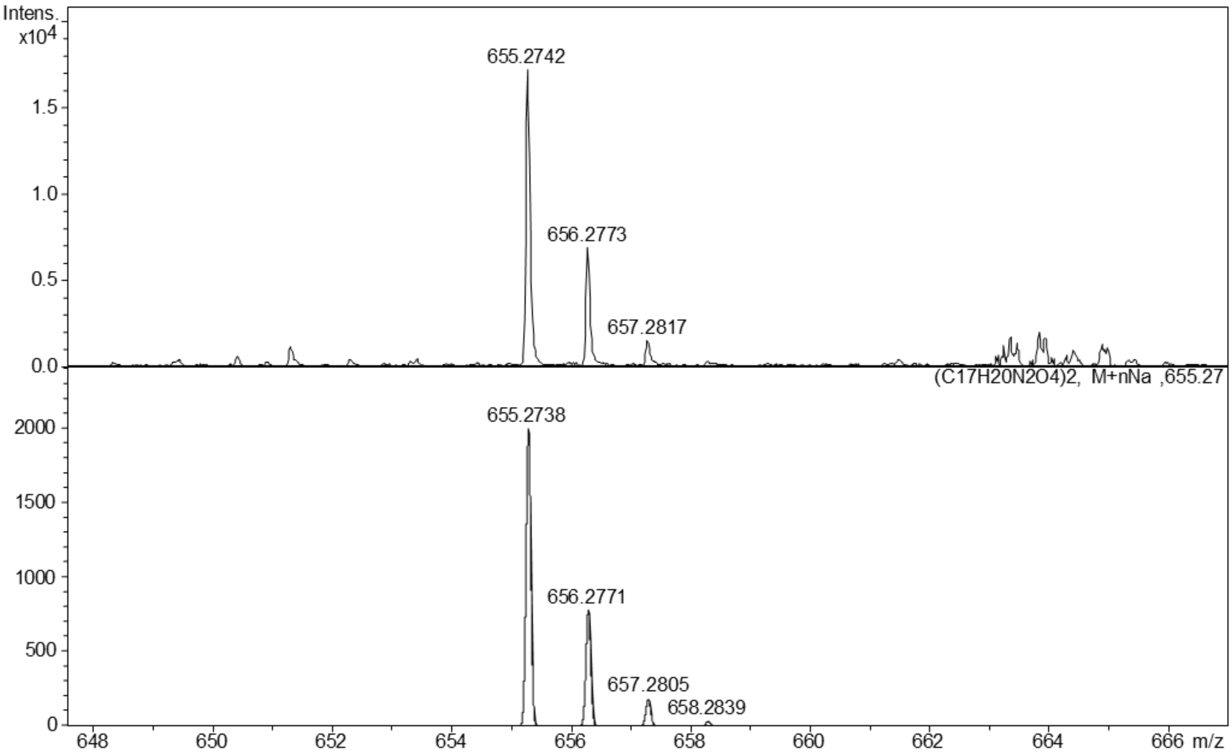

	Molecular formula	Calculated	Found
**$[M+H]^+$**	$C_{17}H_{21}N_2O_4$	317.1496	317.1500
**$[2M+Na]^+$**	$(C_{17}H_{20}N_2O_4)_2Na$	655.2738	655.2742

8-(4-Methoxybenzyl)-7,7a-dimethyl-4a-phenyl-3,4-dihydro-3,7-epiminofuro[3,4-*b*]pyridine-2,5(1*H*)-dione (24)

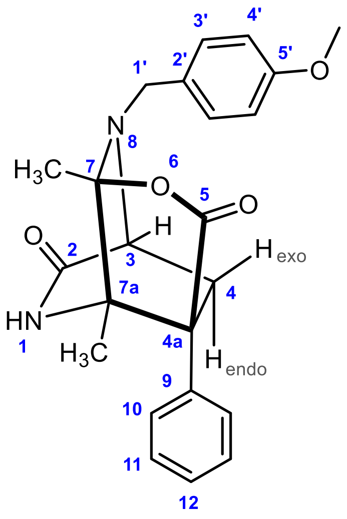

24

^{1}H-NMR (400 MHz, CDCl3), *δ* (ppm)

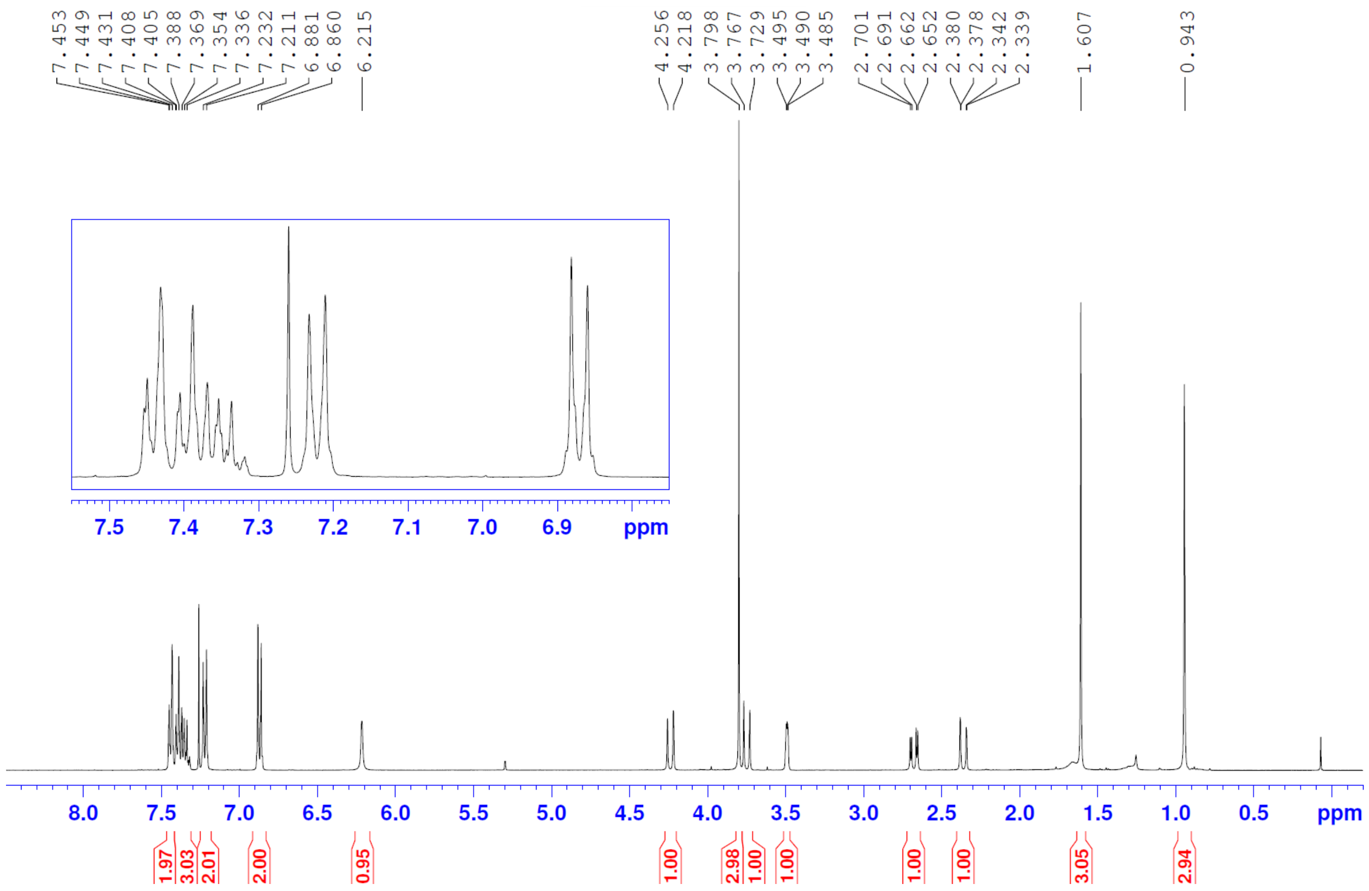

^{1}H-NMR (400 MHz, CDCl$_3$), *δ* (ppm) – Aliphatic zoom

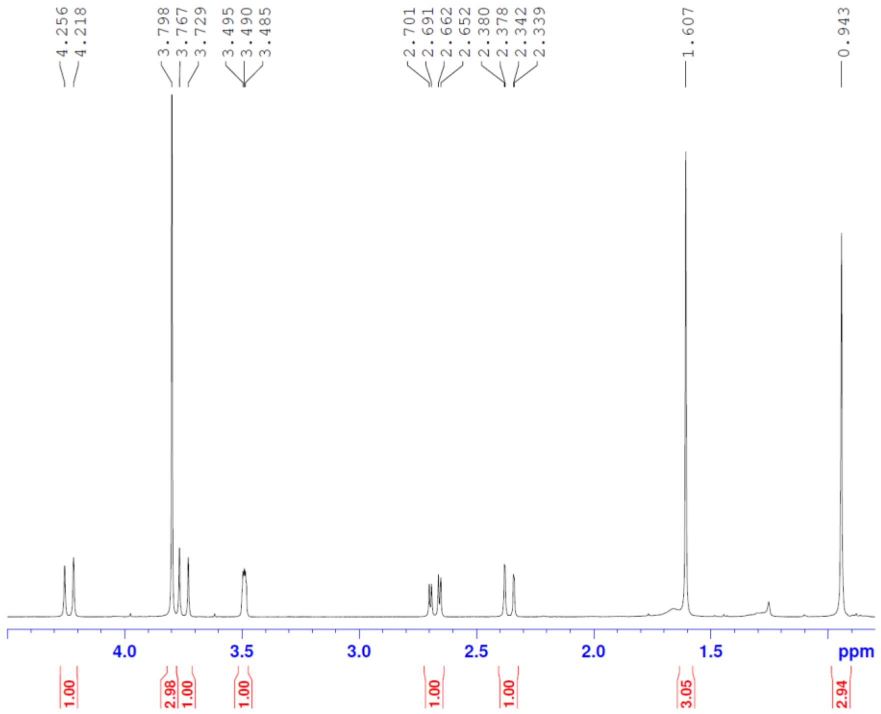

^{13}C{^{1}H}-NMR and DEPT135 (100 MHz, CDCl$_3$), *δ* (ppm)

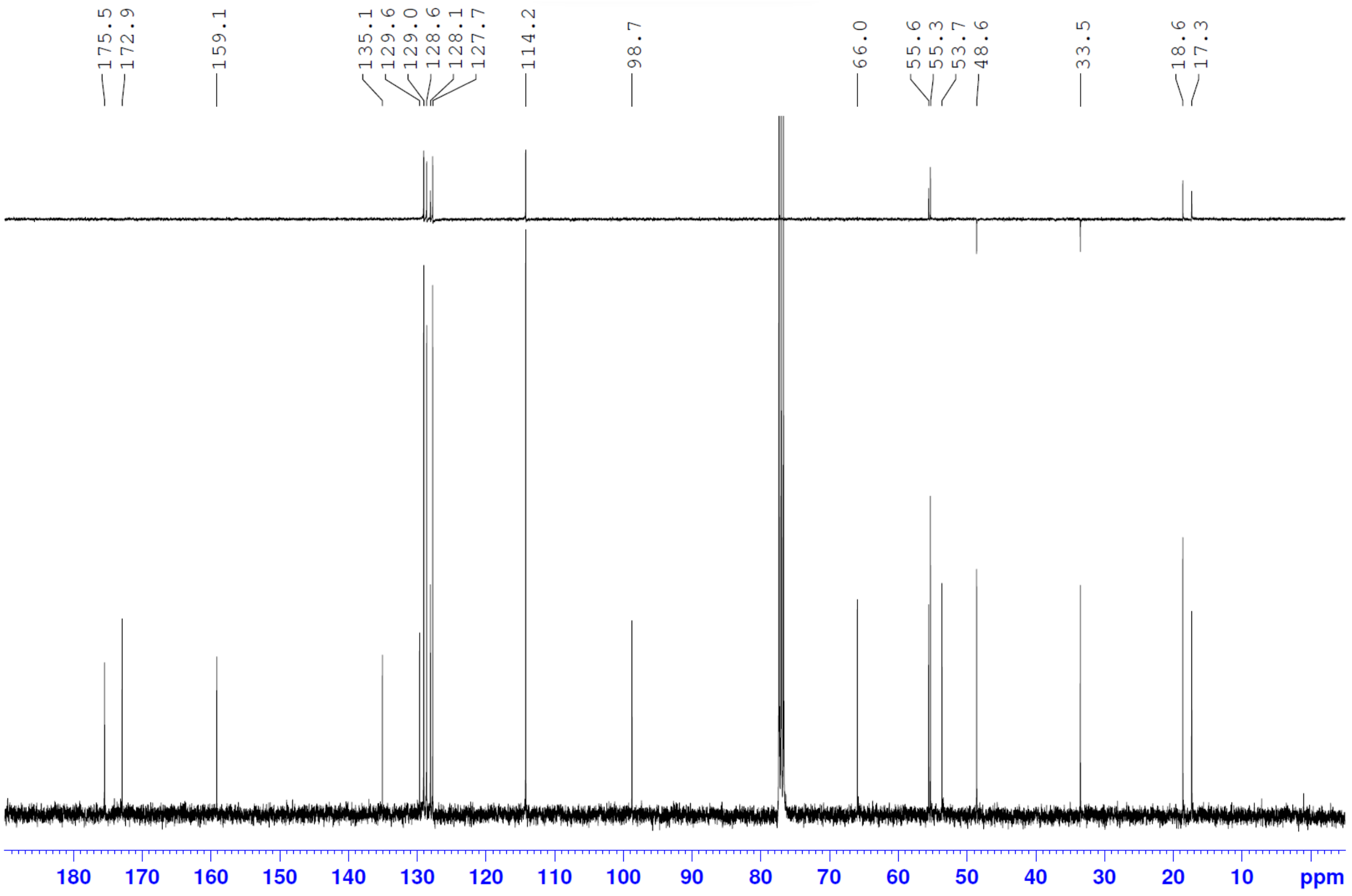

COSY ^{1}H-^{1}H ($CDCl_3$), δ (ppm)

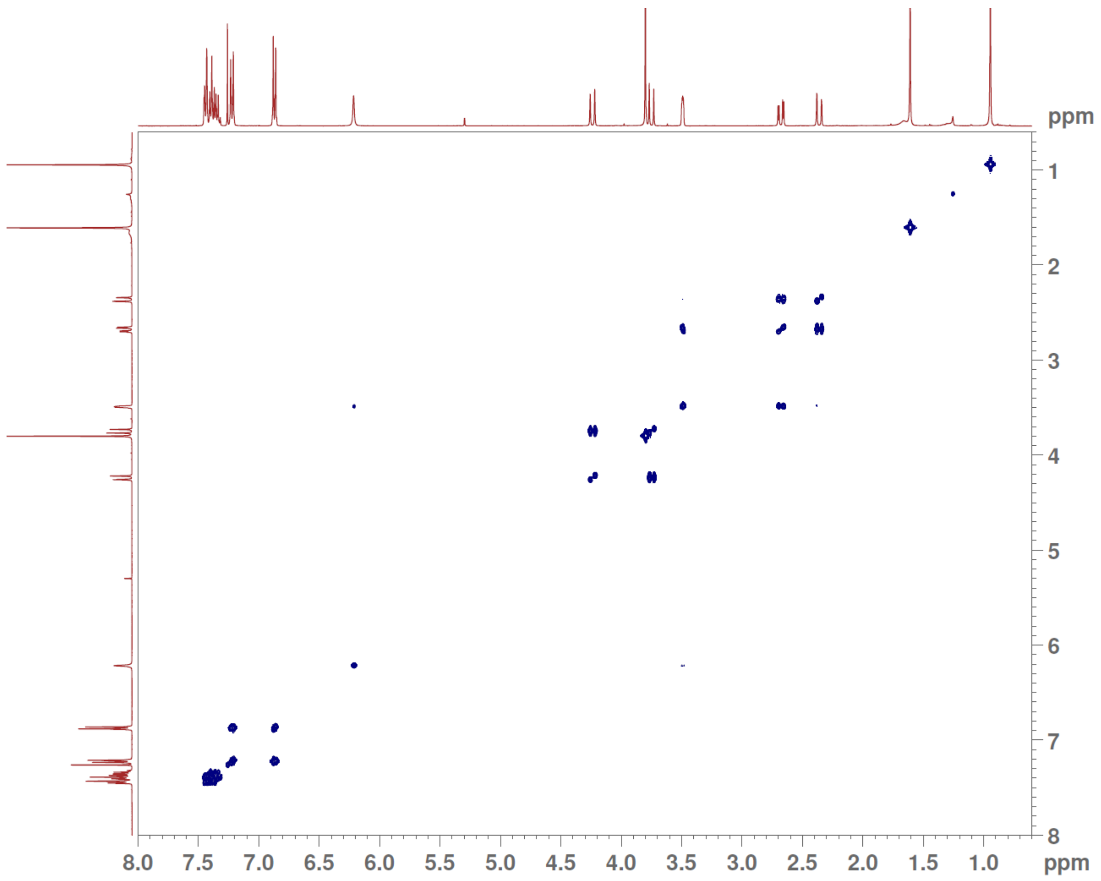

NOESY ^{1}H-^{1}H ($CDCl_3$), δ (ppm)

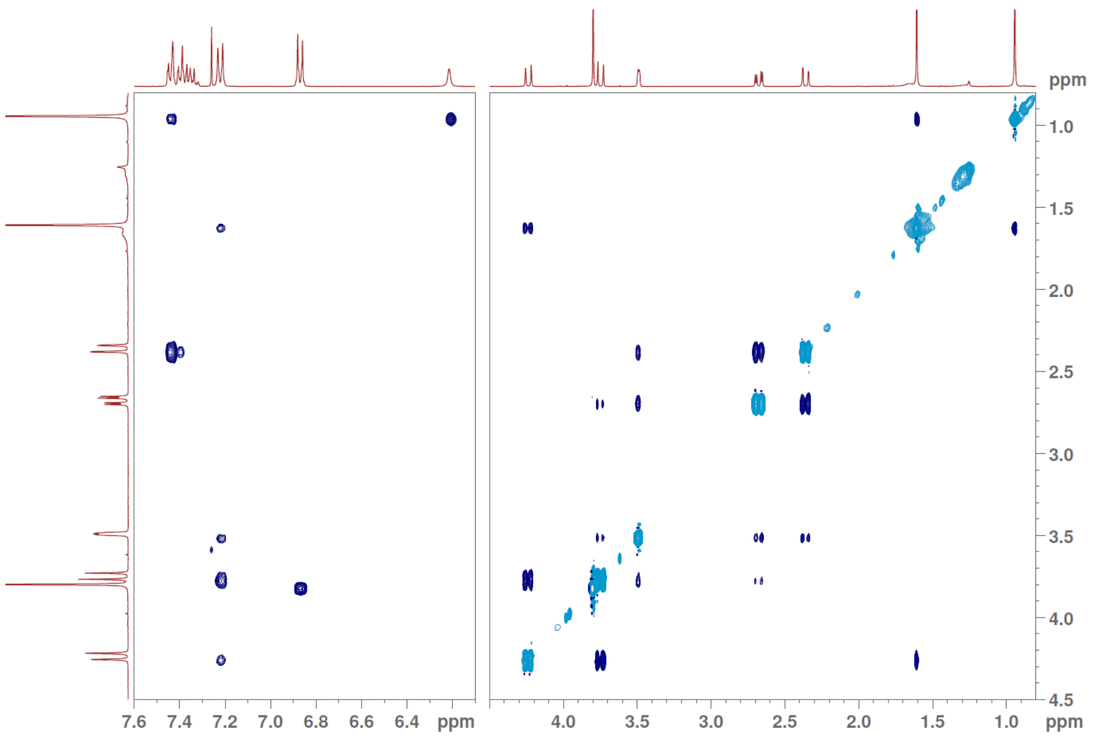

HSQC ^{1}H-^{13}C ($CDCl_3$), δ (ppm)

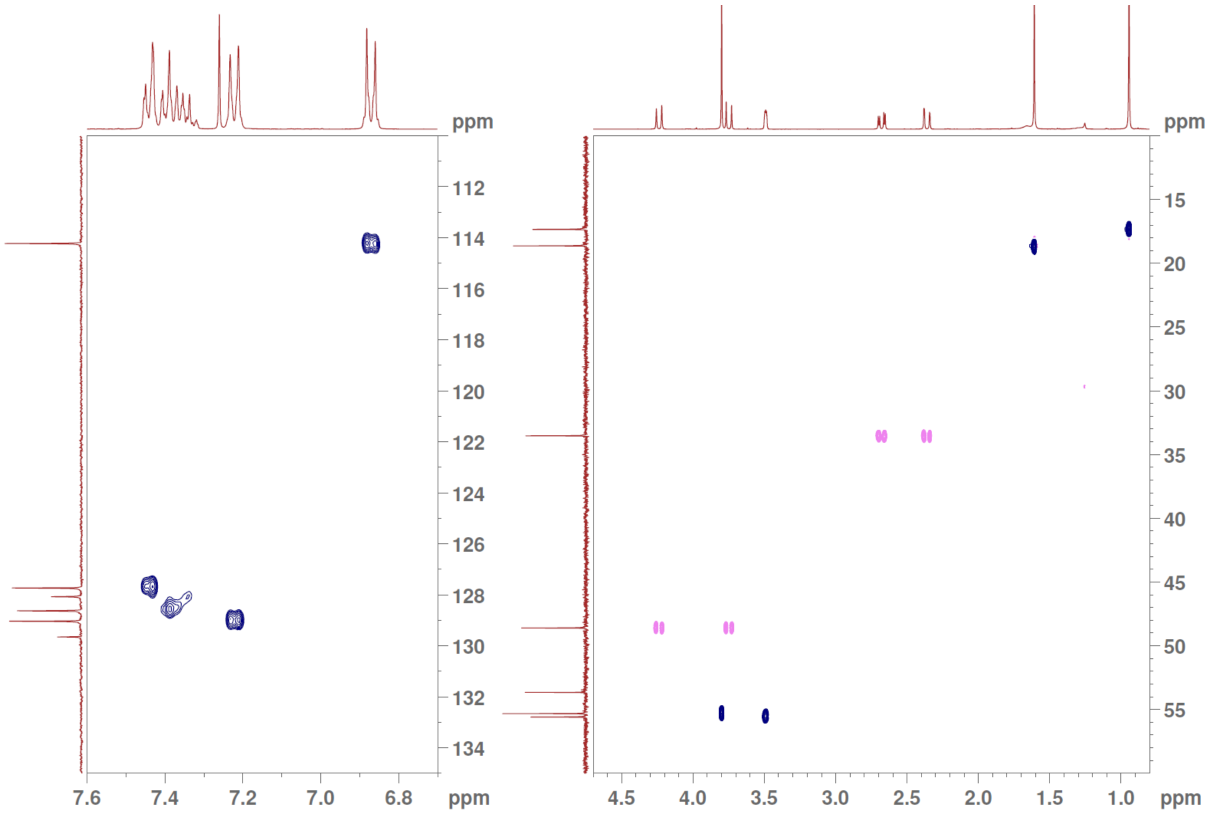

HMBC ^{1}H-^{13}C ($CDCl_3$), δ (ppm)

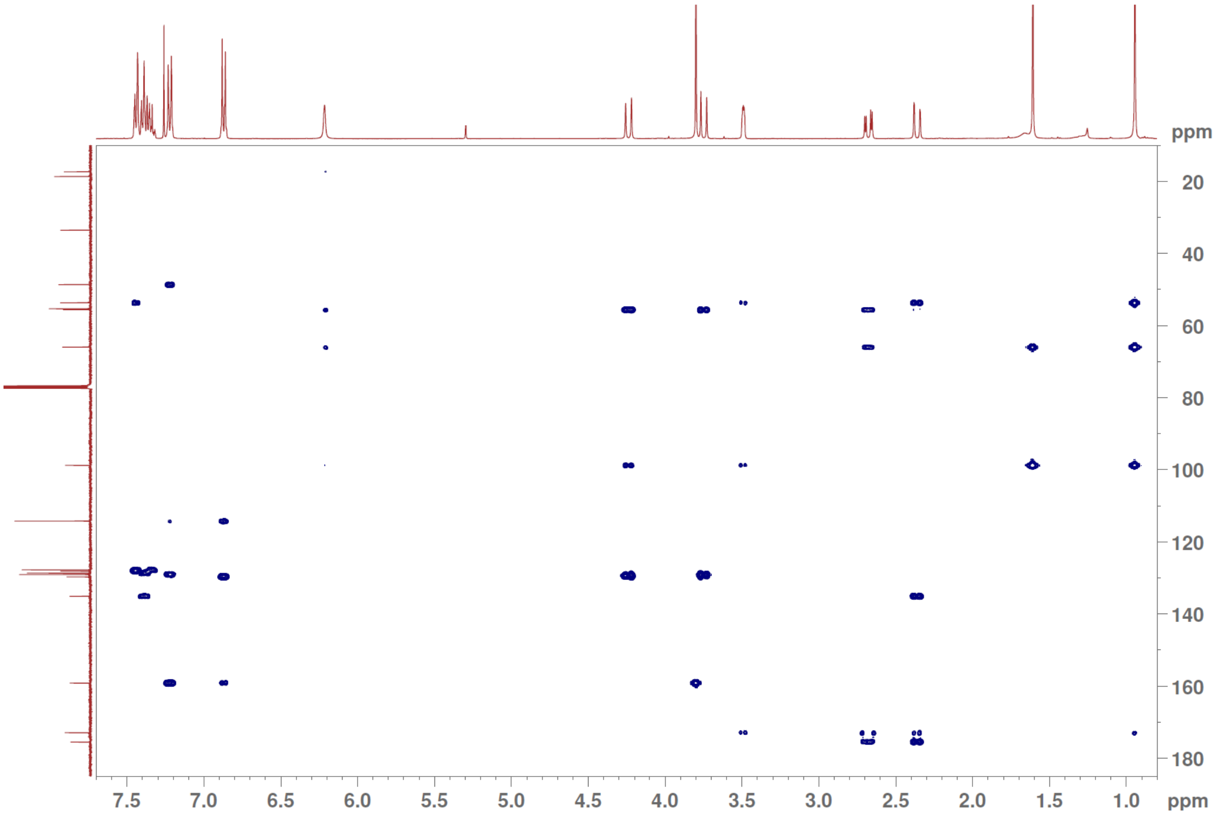

FT-IR (neat), ν (cm^{-1})

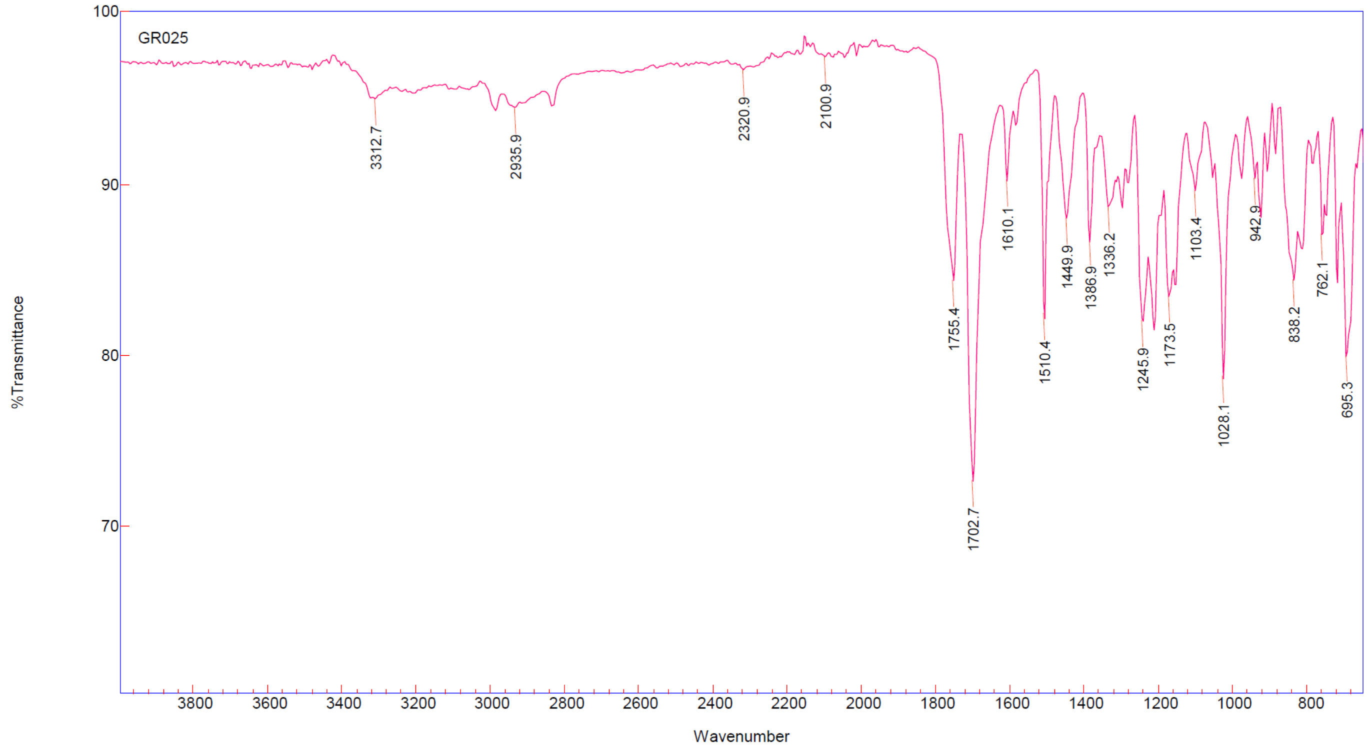

HPLC (λ = 220 nm)

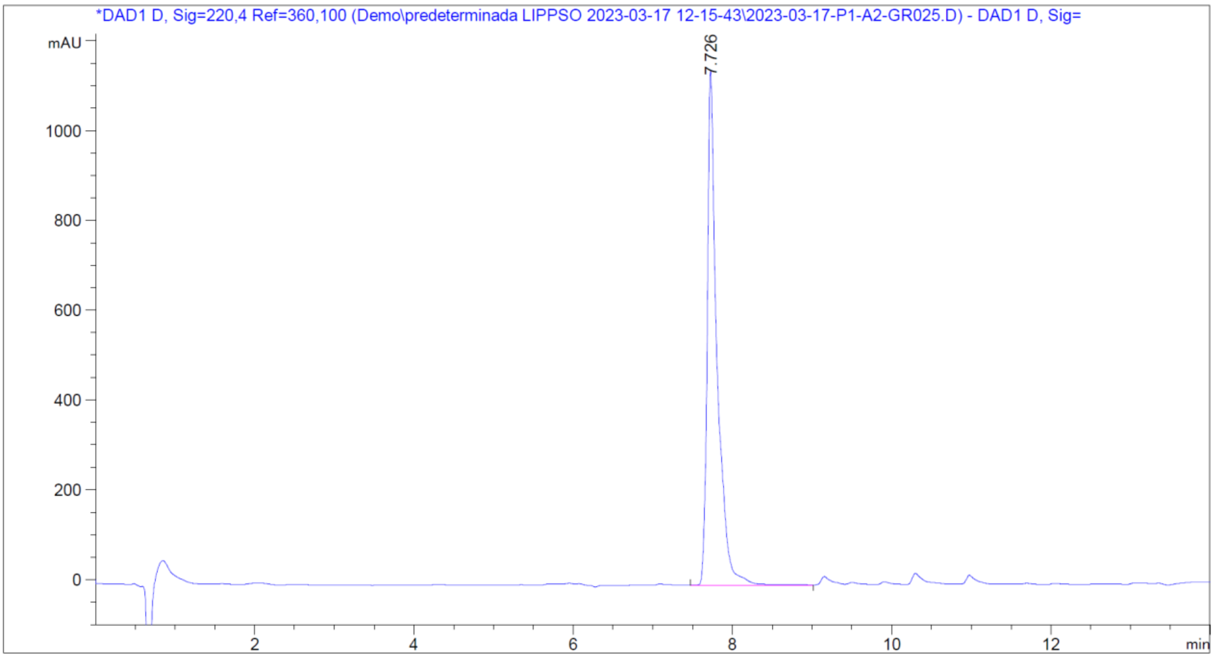

Peak #	RetTime [min]	Type	Width [min]	Area [mAU*s]	Height [mAU]	Area %
1	7.726	BV R	0.1185	9786.80371	1147.04639	100.0000
Totals :				9786.80371	1147.04639	

ESI-MS (*m/z*)

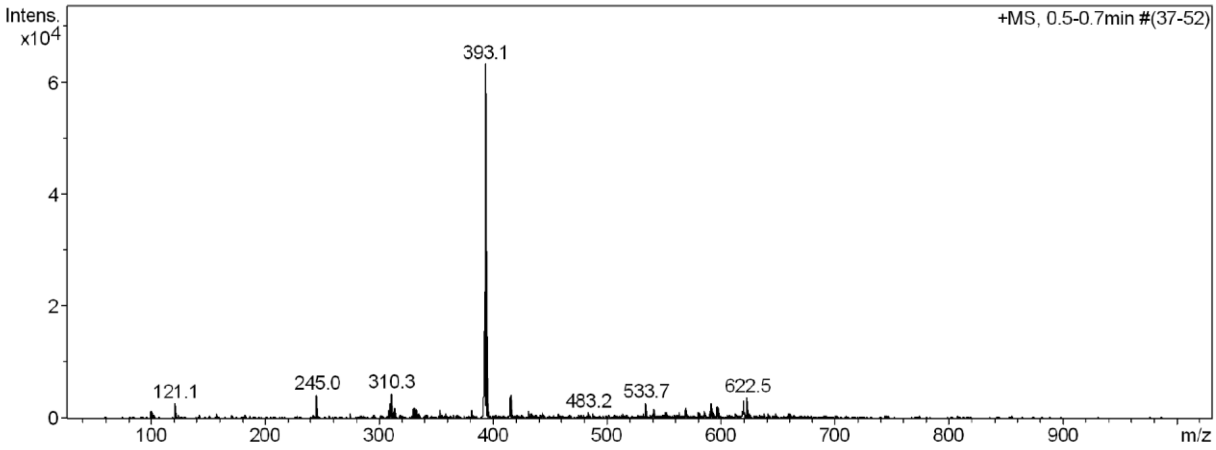

ESI-MS/MS (*m/z* = 393.1)

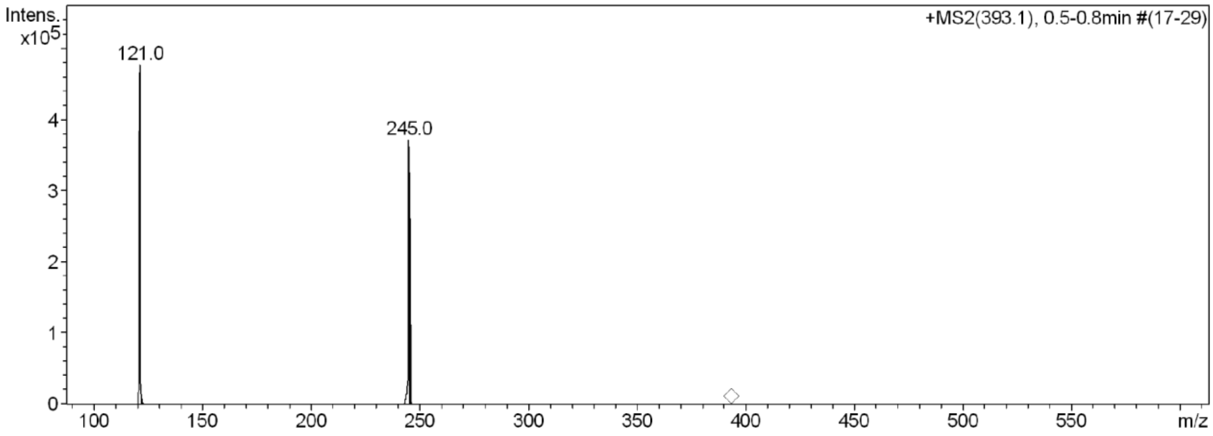

HRMS (*m/z*)

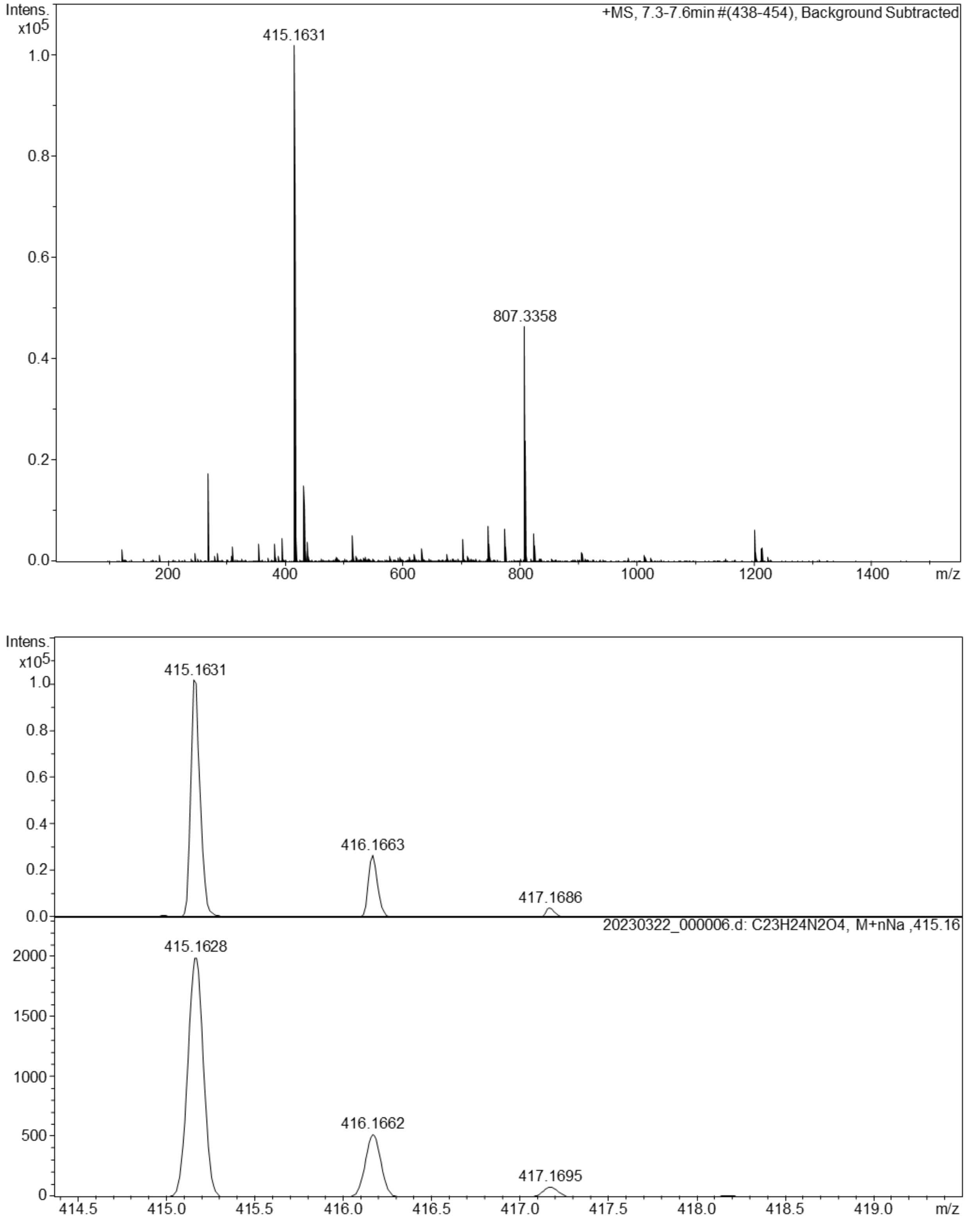

Intens.
x10^5
+MS, 7.3-7.6min #(438-454), Background Subtracted
415.1631
807.3358
m/z
415.1631
416.1663
417.1686
20230322_000006.d: C23H24N2O4, M+nNa ,415.16
415.1628
416.1662
417.1695
m/z

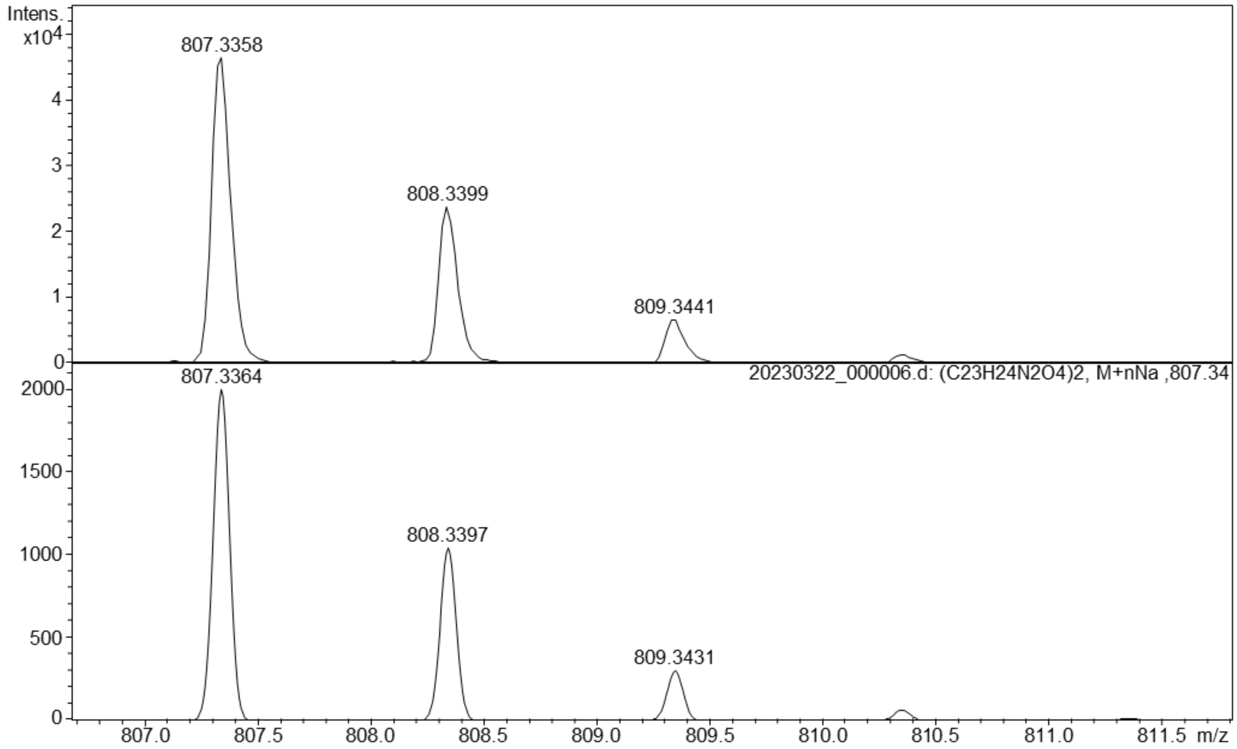

	Molecular formula	Calculated	Found
**[M+Na]+**	$C_{23}H_{24}N_2O_4Na$	415.1628	415.1631
**[2M+Na]+**	$(C_{23}H_{24}N_2O_4)_2Na$	807.3364	807.3358

2-(4-Methoxybenzyl)-8-(methoxycarbonyl)-3,4-dimethyl-6-oxo-2,5-diazabicyclo[2.2.2]oct-2-en-2-ium trifluoroacetate (25a)

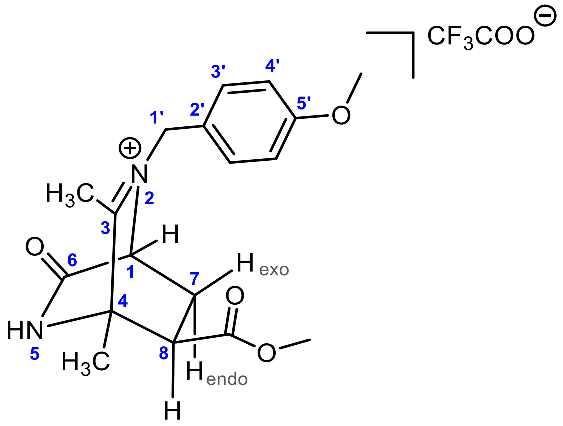

25a

^{1}H-NMR (400 MHz, CDCl$_3$), δ (ppm)

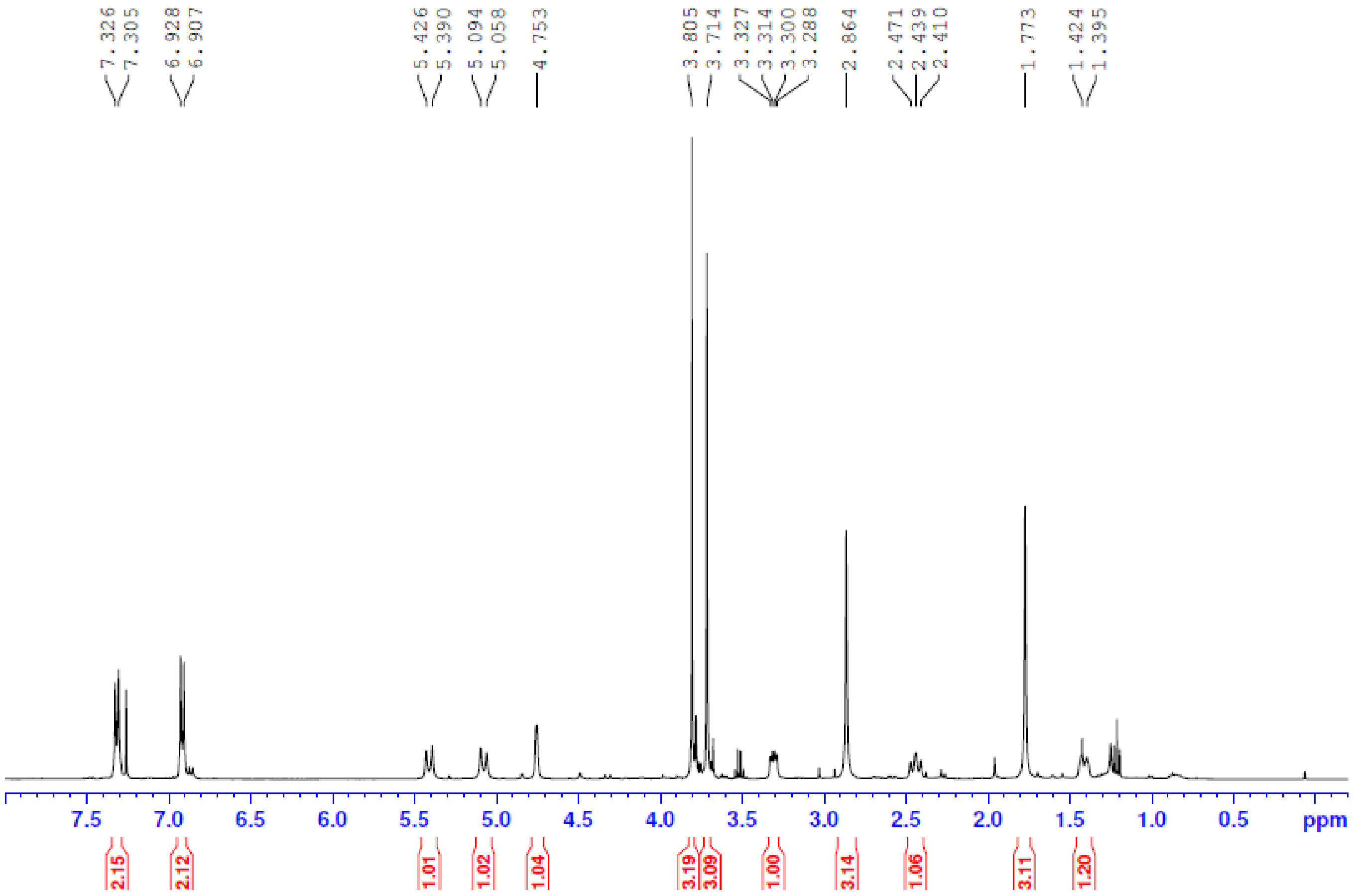

^{1}H-NMR (400 MHz, CDCl$_3$), δ (ppm) – Aliphatic zoom

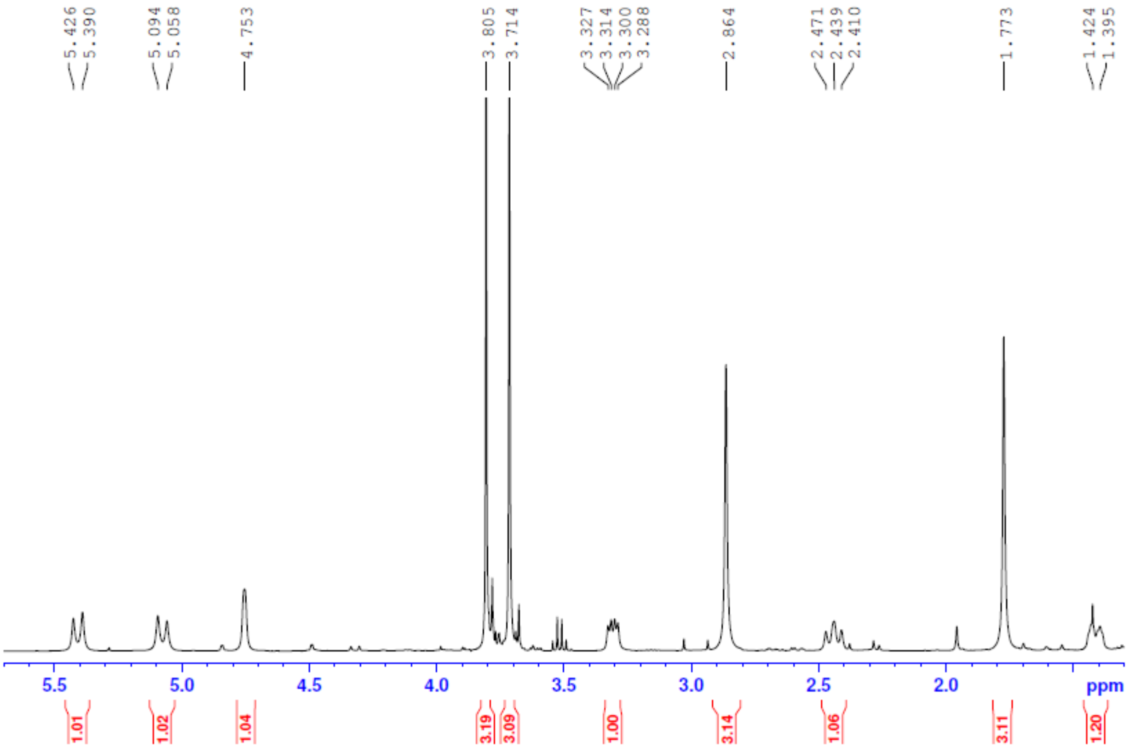

^{13}C{^{1}H}-NMR (100 MHz, CDCl$_3$), δ (ppm)

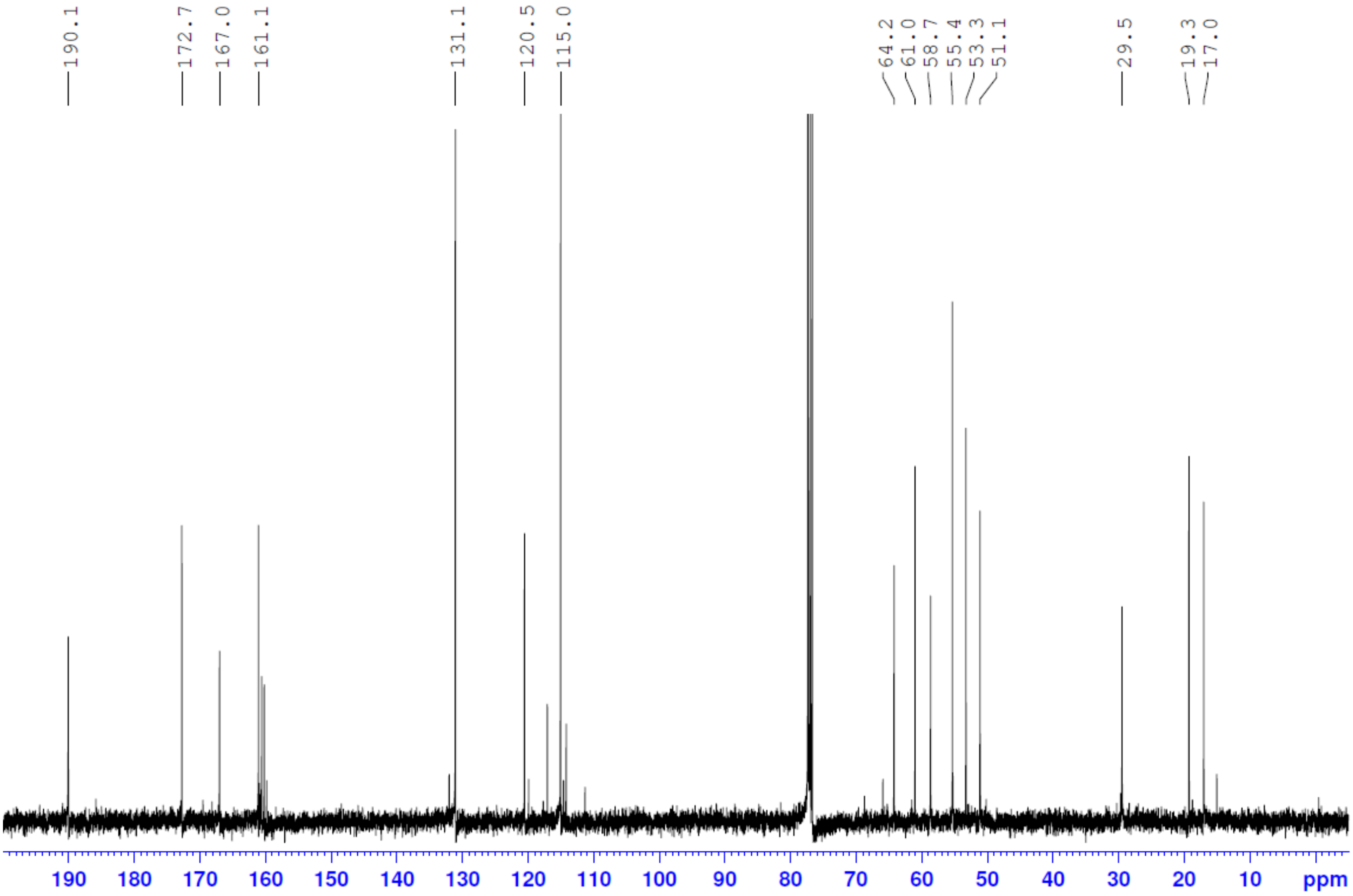

^{19}F-NMR (377 MHz, CDCl$_3$), δ (ppm)

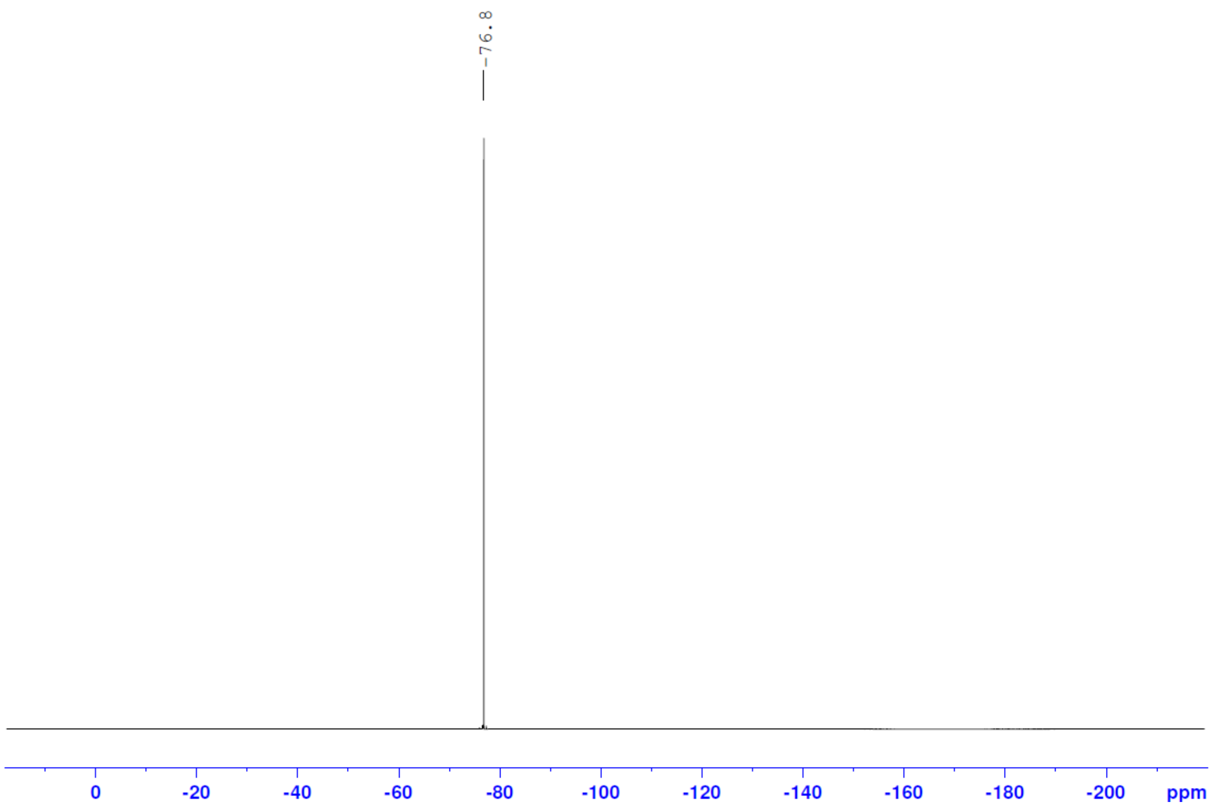

COSY ^{1}H-^{1}H (CDCl$_3$), δ (ppm)

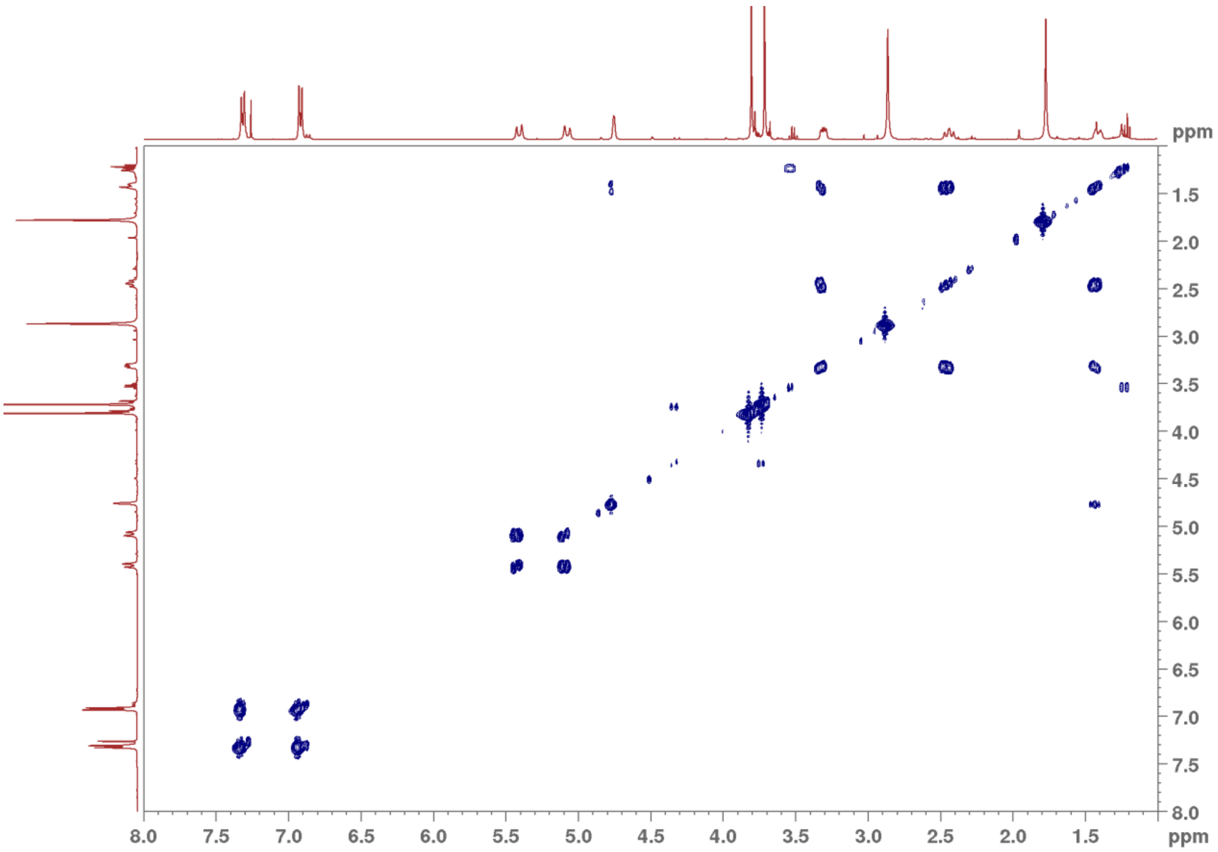

HSQC ^{1}H-^{13}C ($CDCl_3$), δ (ppm)

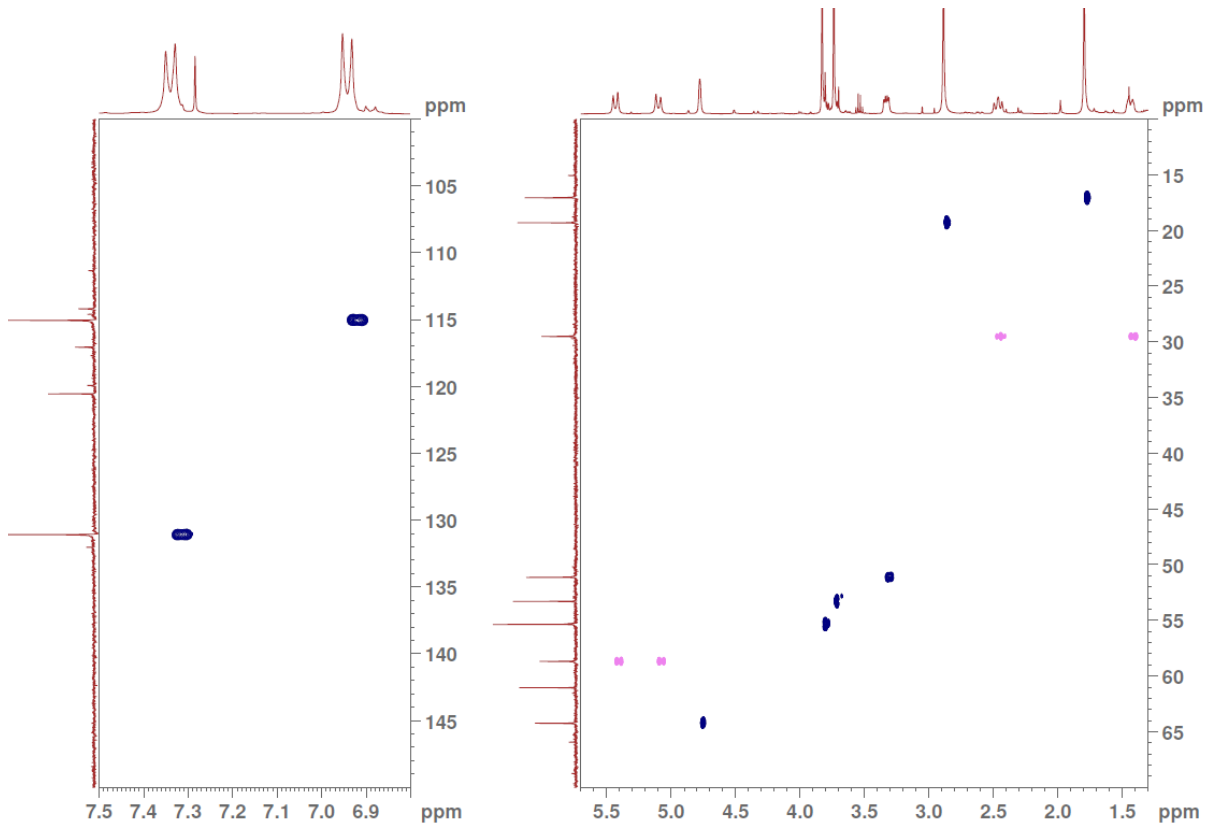

HMBC ^{1}H-^{13}C ($CDCl_3$), δ (ppm) – Aliphatic carbons

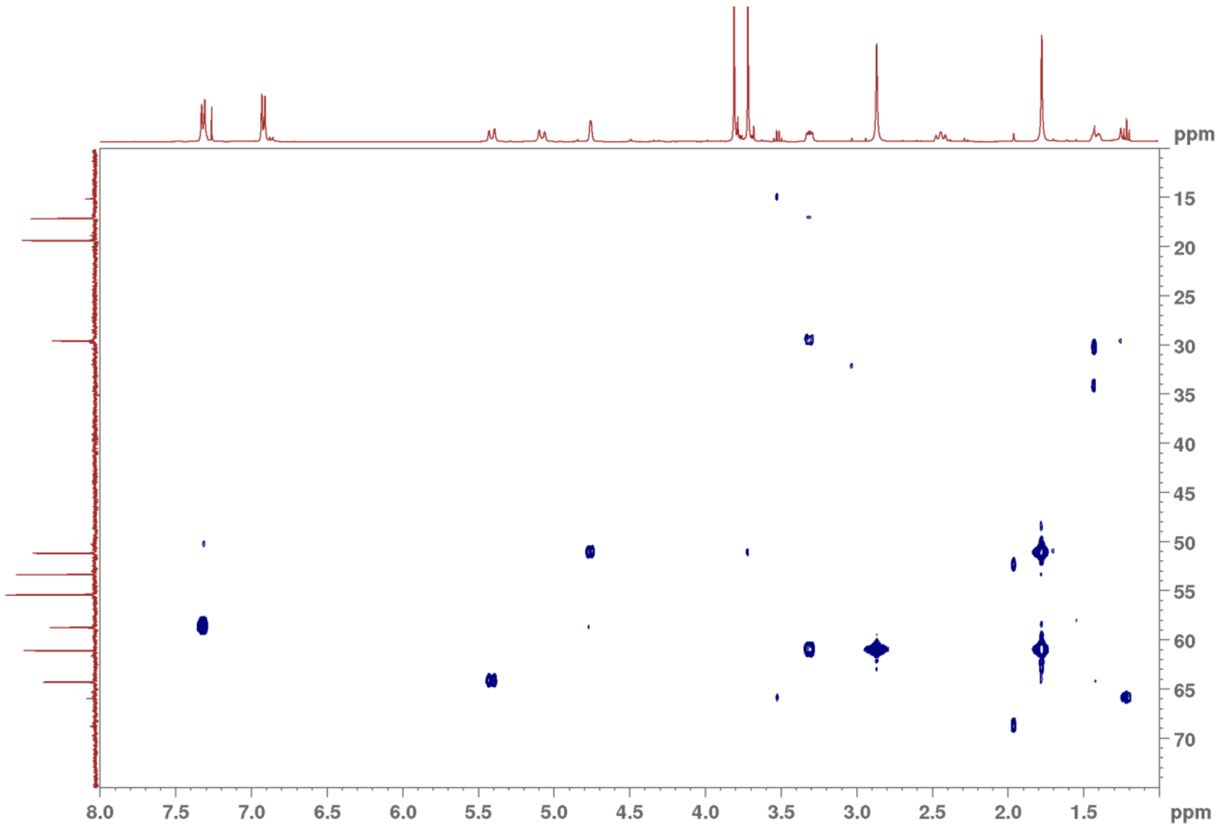

HMBC ^{1}H-^{13}C ($CDCl_3$), δ (ppm) – Aromatic carbons

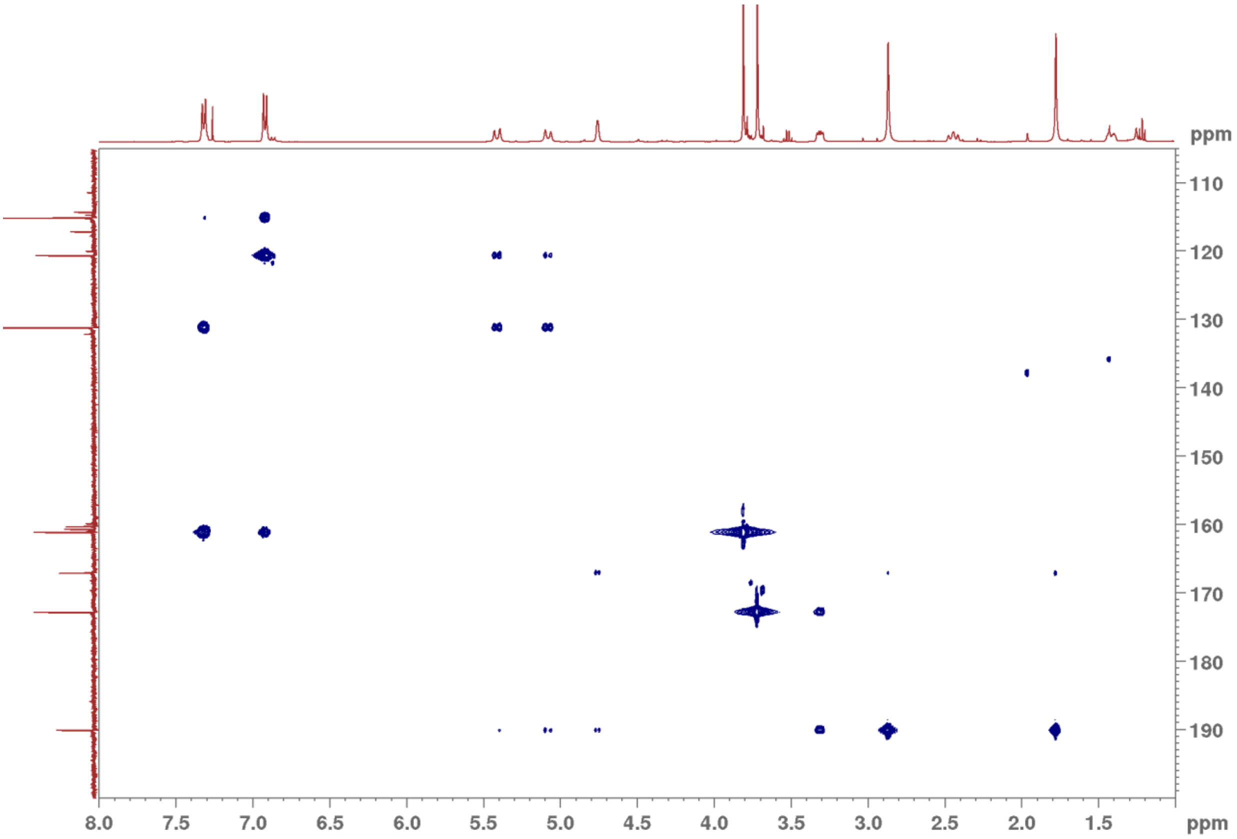

HPLC (λ = 220 nm)

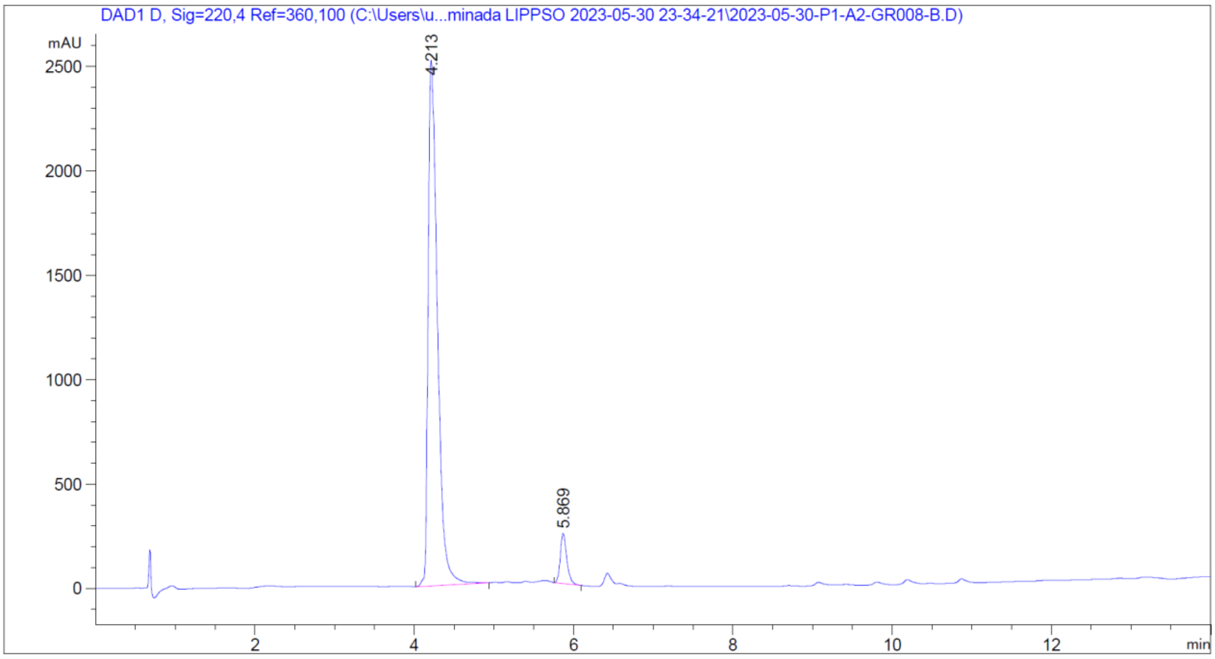

Peak #	RetTime [min]	Type	Width [min]	Area [mAU*s]	Height [mAU]	Area %
1	4.213	BV R	0.1256	2.04208e4	2517.47559	93.7615
2	5.869	BB	0.0850	1358.71777	240.11804	6.2385
Totals :				2.17795e4	2757.59363	

ESI-MS (*m/z*)

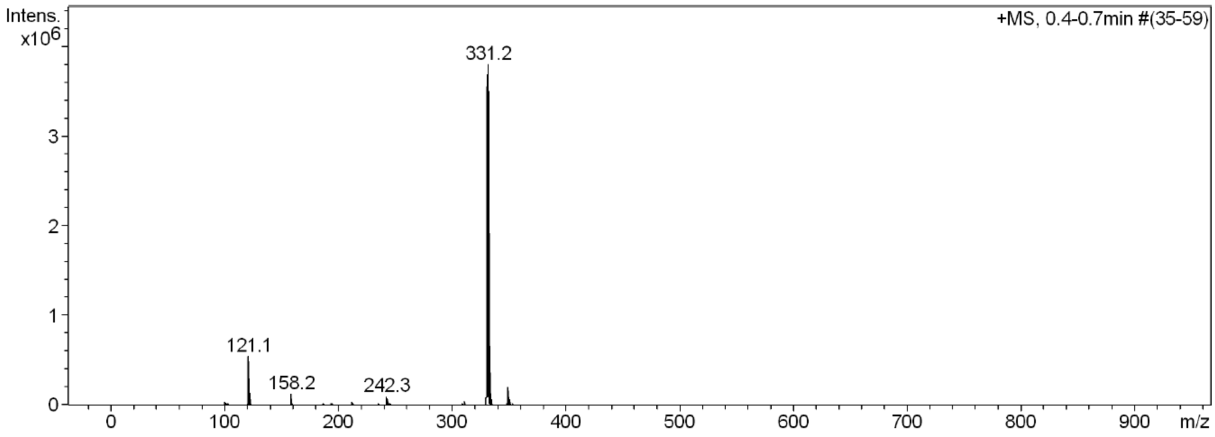

HRMS (*m/z*)

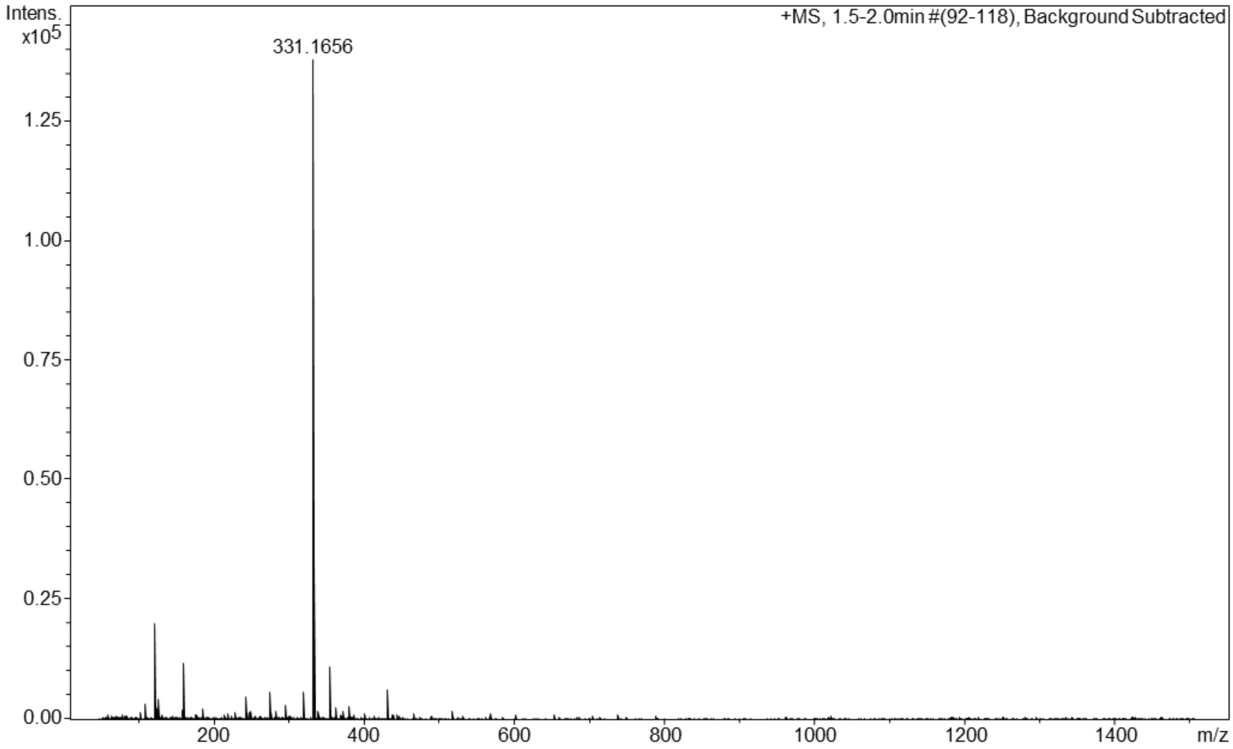

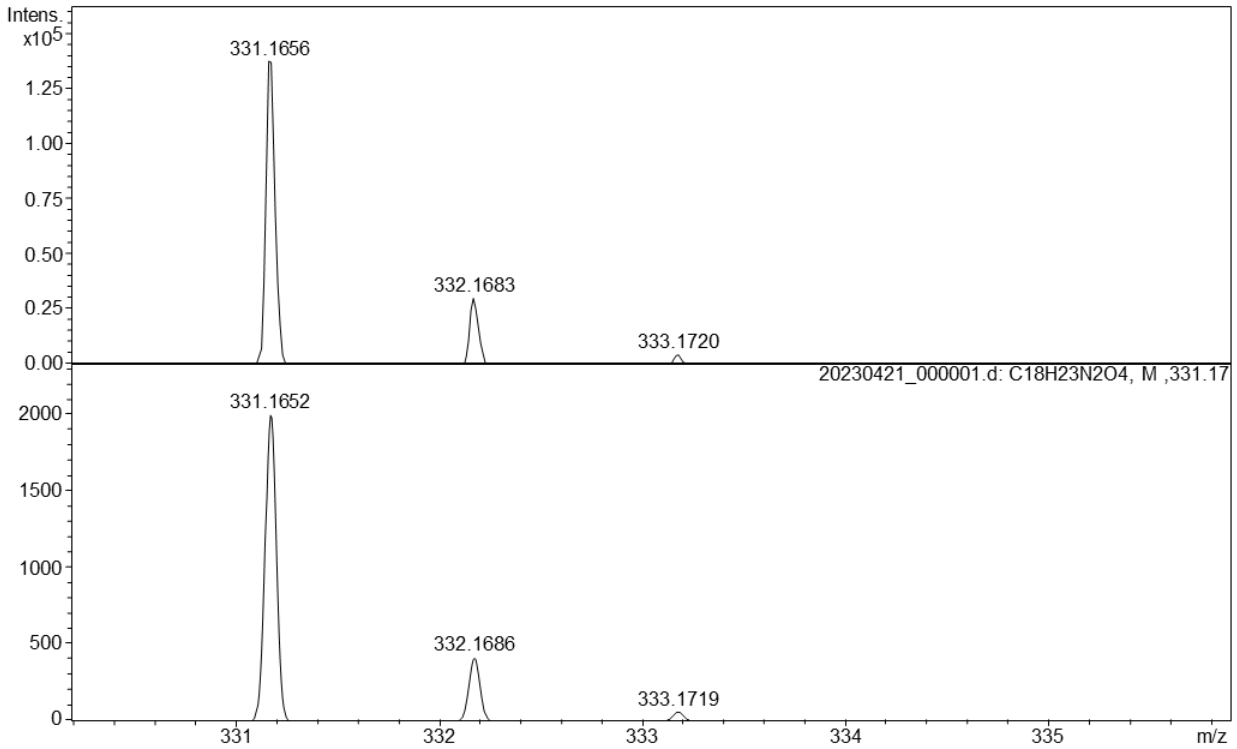

	**Molecular formula**	**Calculated**	**Found**
**[M]$^+$**	$C_{18}H_{23}N_2O_4$	331.1652	331.1656

Methyl 5-(4-methoxybenzyl)-1-methyl-6-methylene-3-oxo-2,5-diazabicyclo[2.2.2]octane-7-carboxylate (21a)

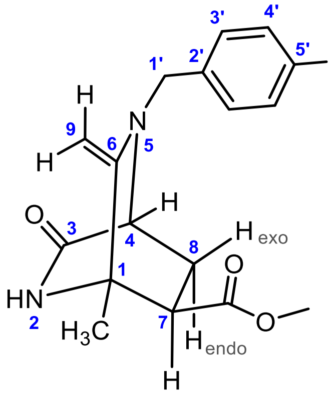

21a

^{1}H-NMR (400 MHz, CDCl$_3$), δ (ppm)

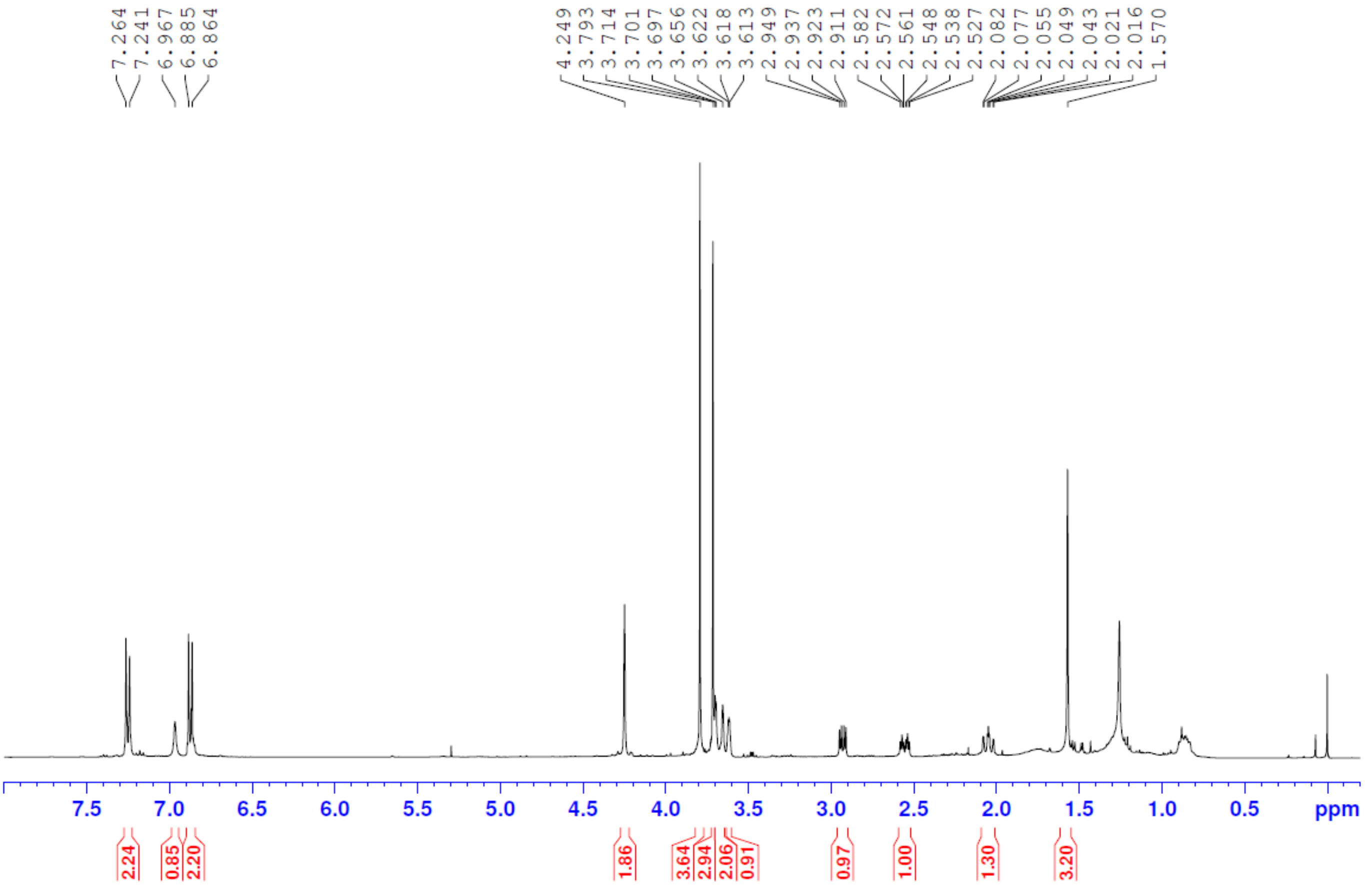

^{1}H-NMR (400 MHz, CDCl$_3$), δ (ppm) – Aliphatic zoom

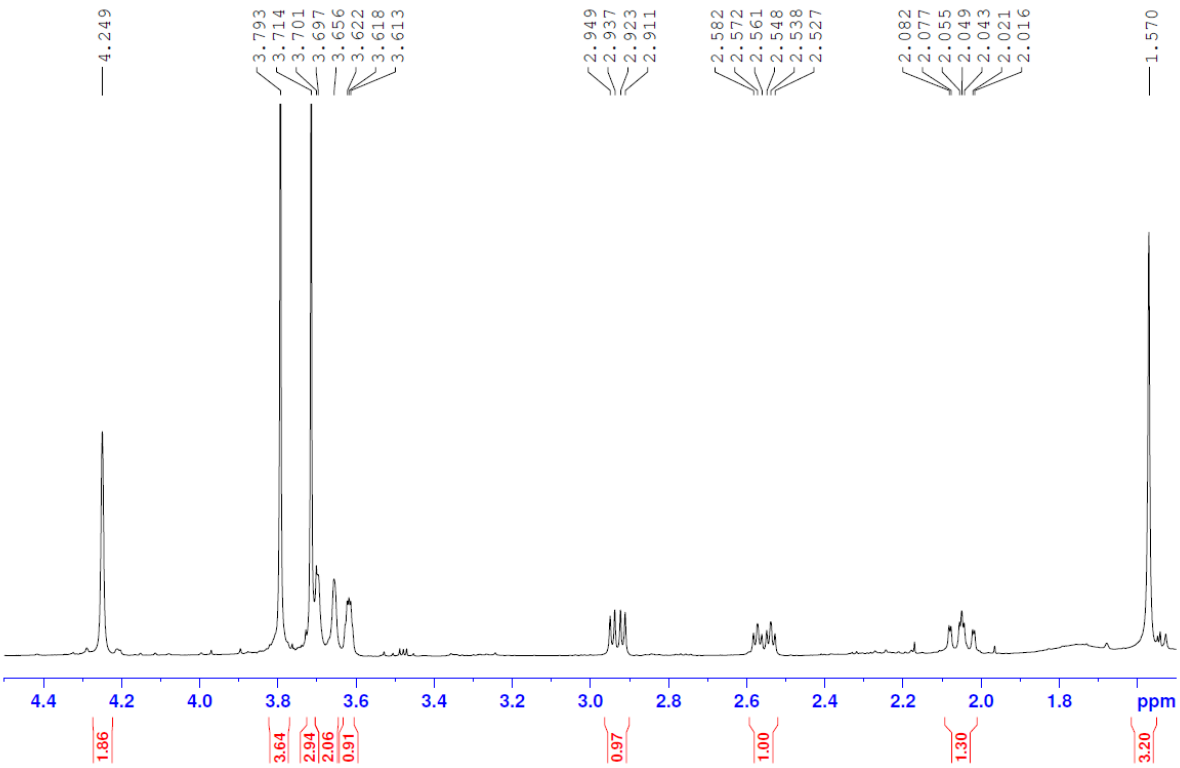

^{13}C{^{1}H}-NMR (100 MHz, CDCl$_3$), δ (ppm)

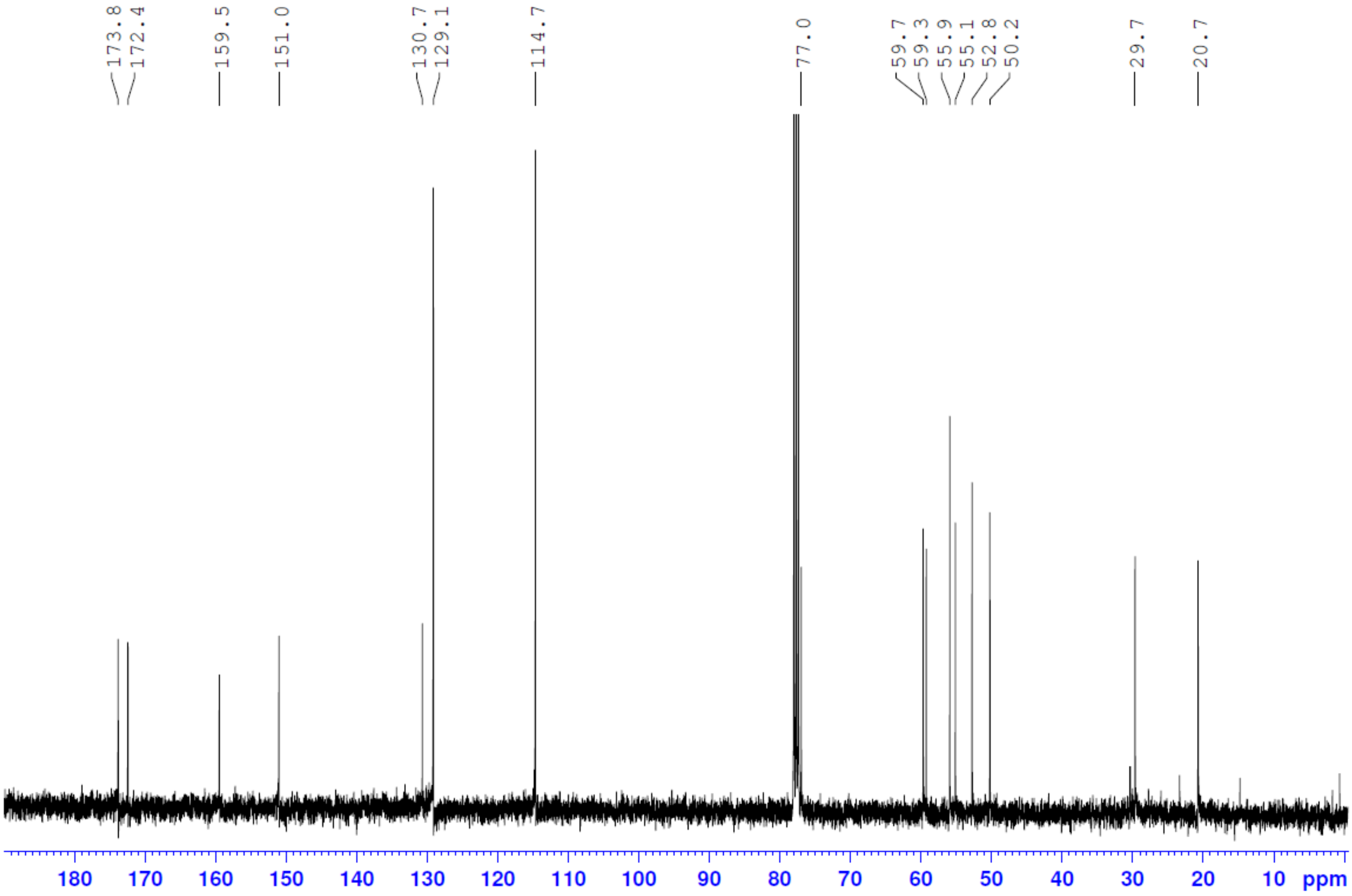

COSY ¹H-¹H (CDCl₃), δ (ppm)

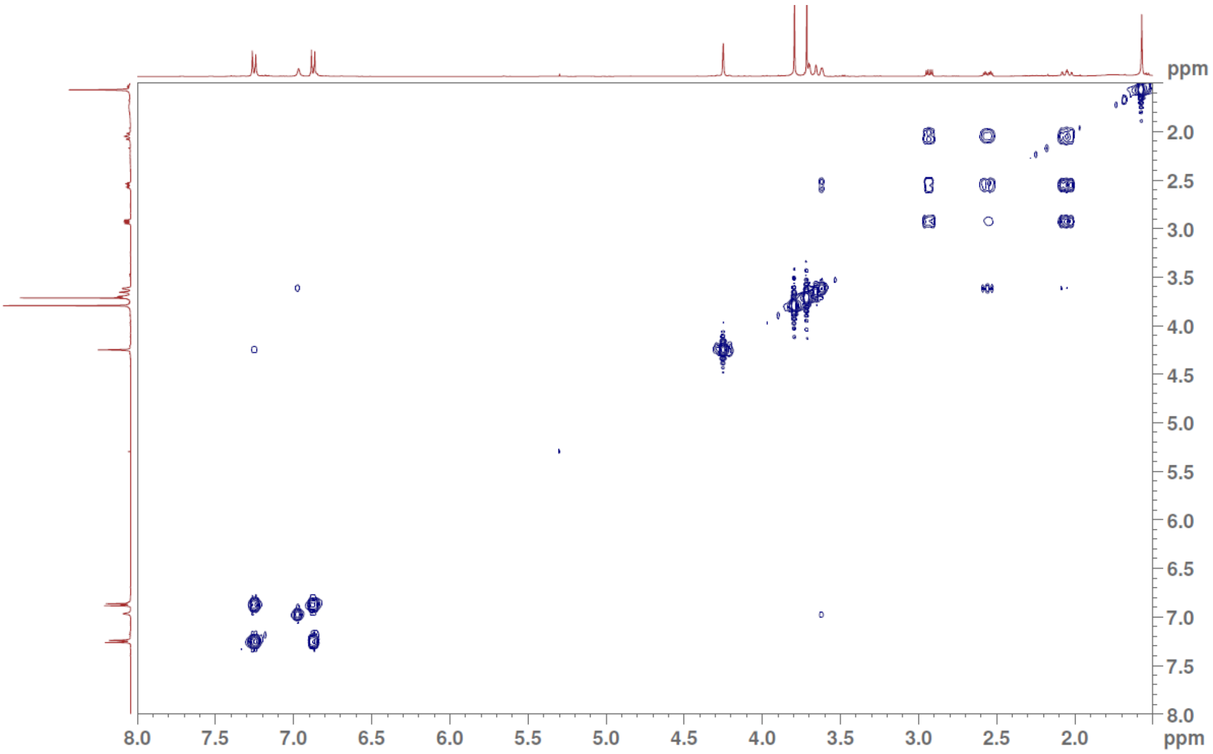

COSY ¹H-¹H (CDCl₃), δ (ppm) – Aliphatic Zoom

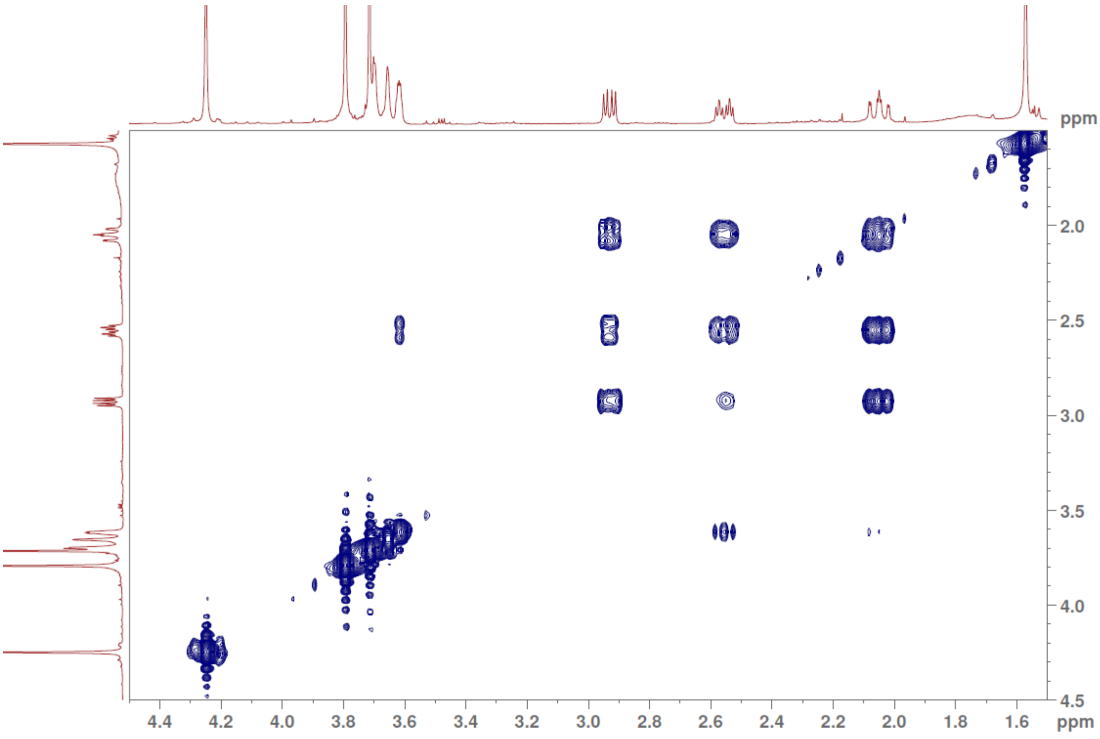

NOESY ^1H-^1H ($CDCl_3$), δ (ppm)

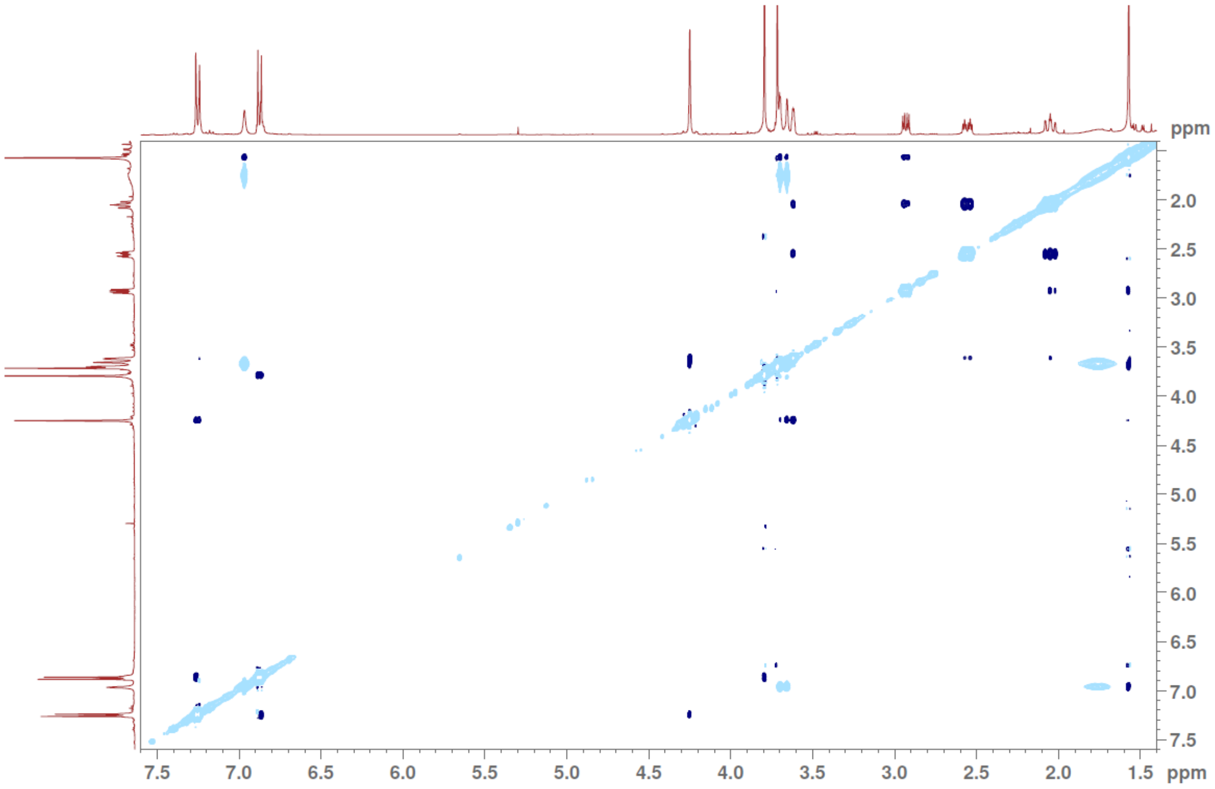

NOESY ^1H-^1H ($CDCl_3$), δ (ppm) – Aliphatic Zoom

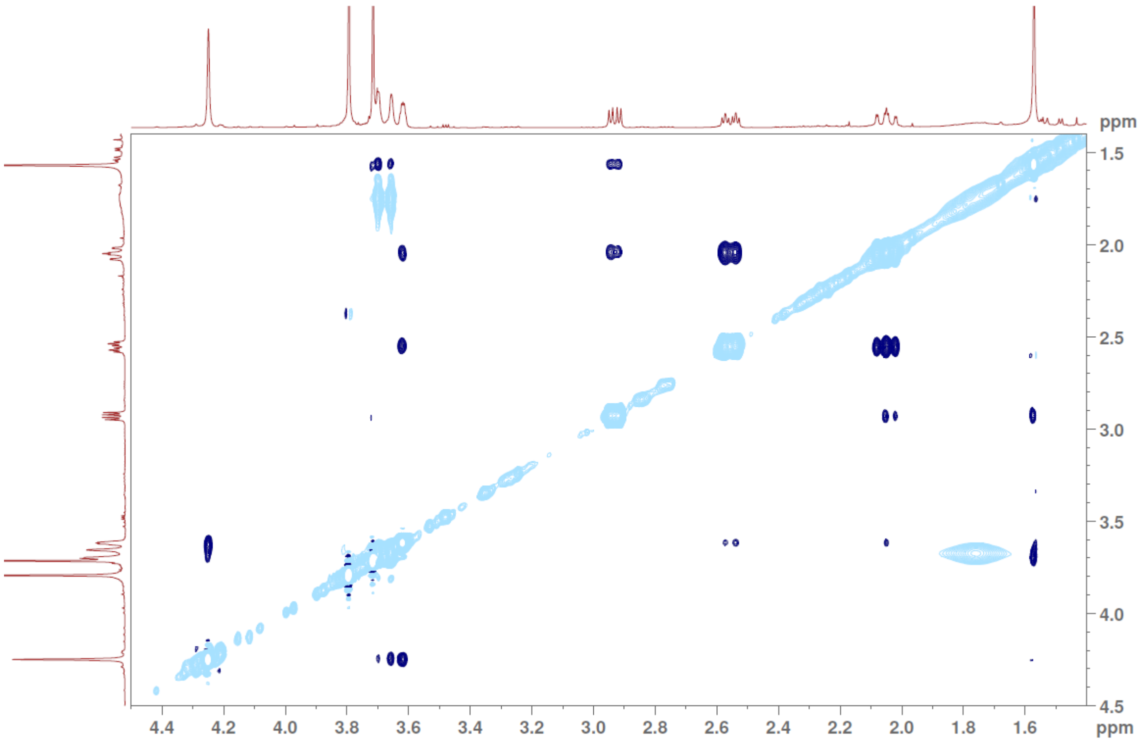

HSQC ^{1}H-^{13}C ($CDCl_3$), δ (ppm)

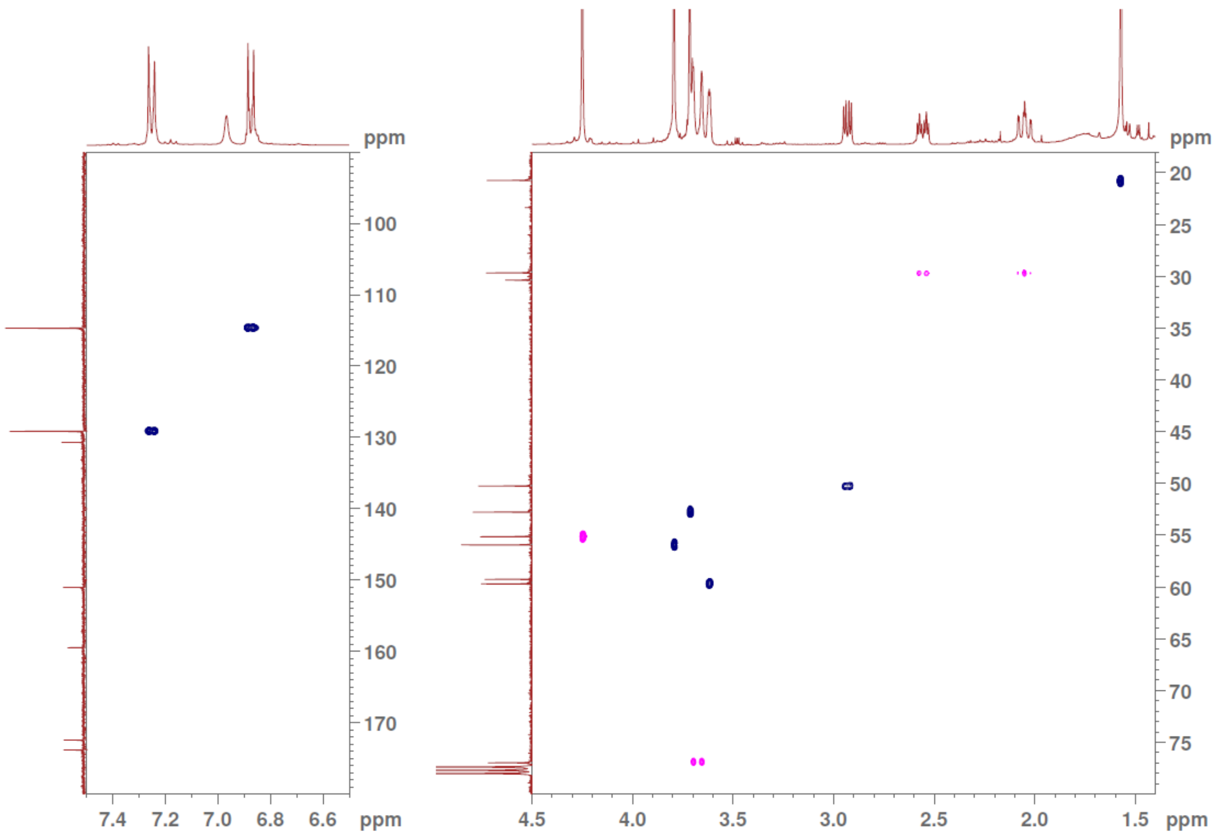

HMBC ^{1}H-^{13}C ($CDCl_3$), δ (ppm)

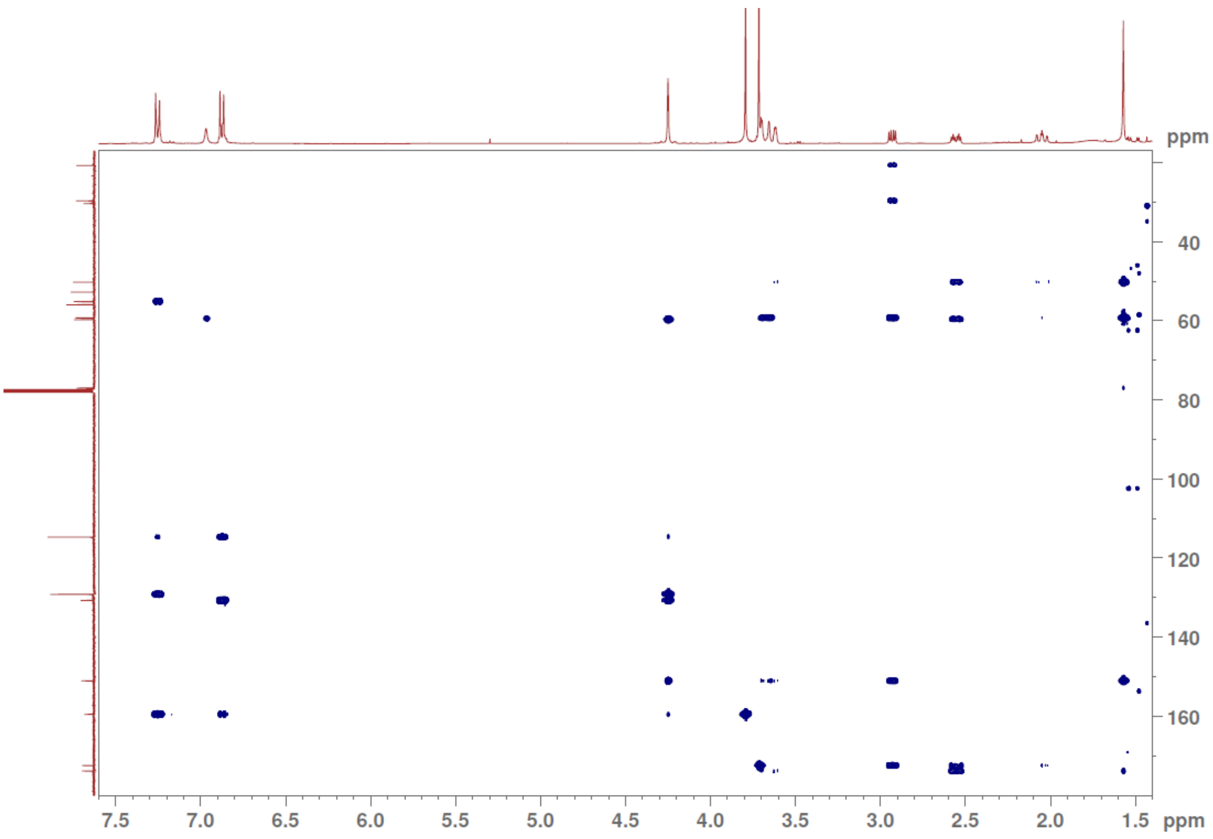

FT-IR (neat), ν (cm^{-1})

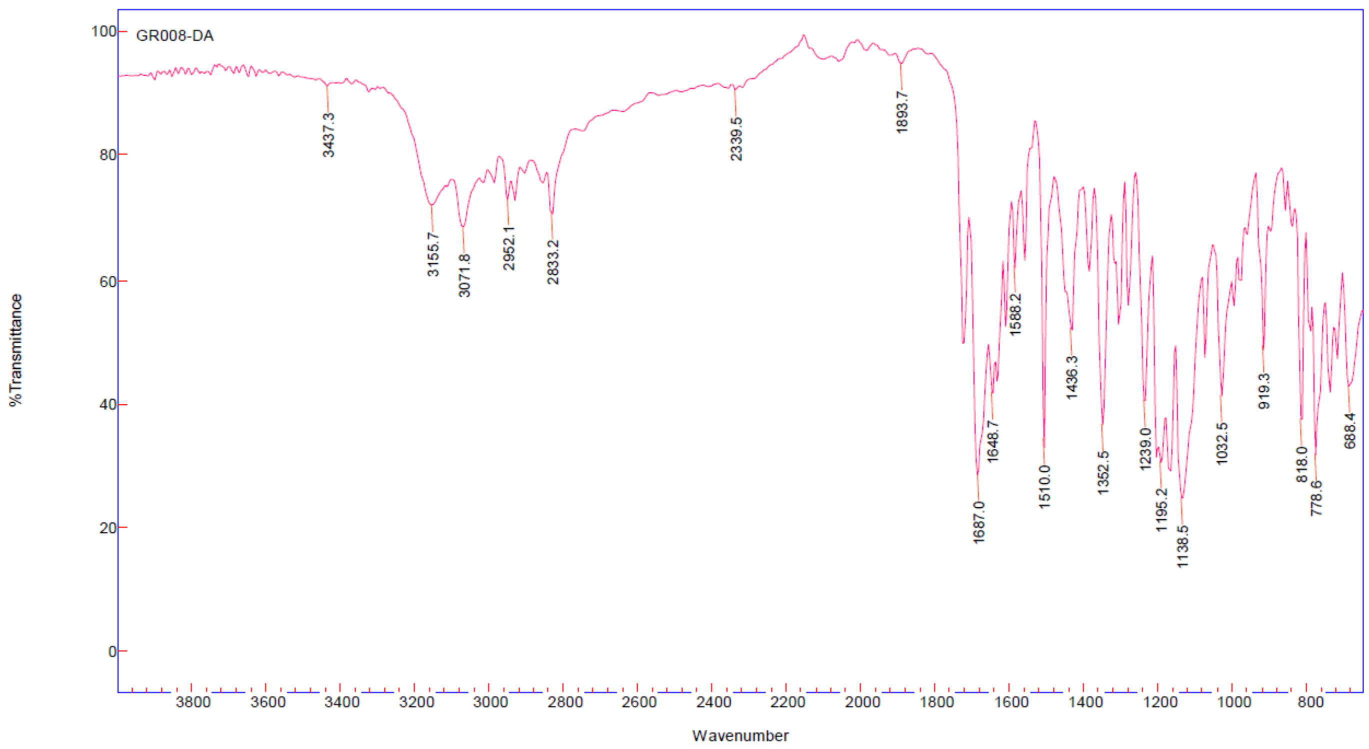

HPLC (λ = 220 nm)

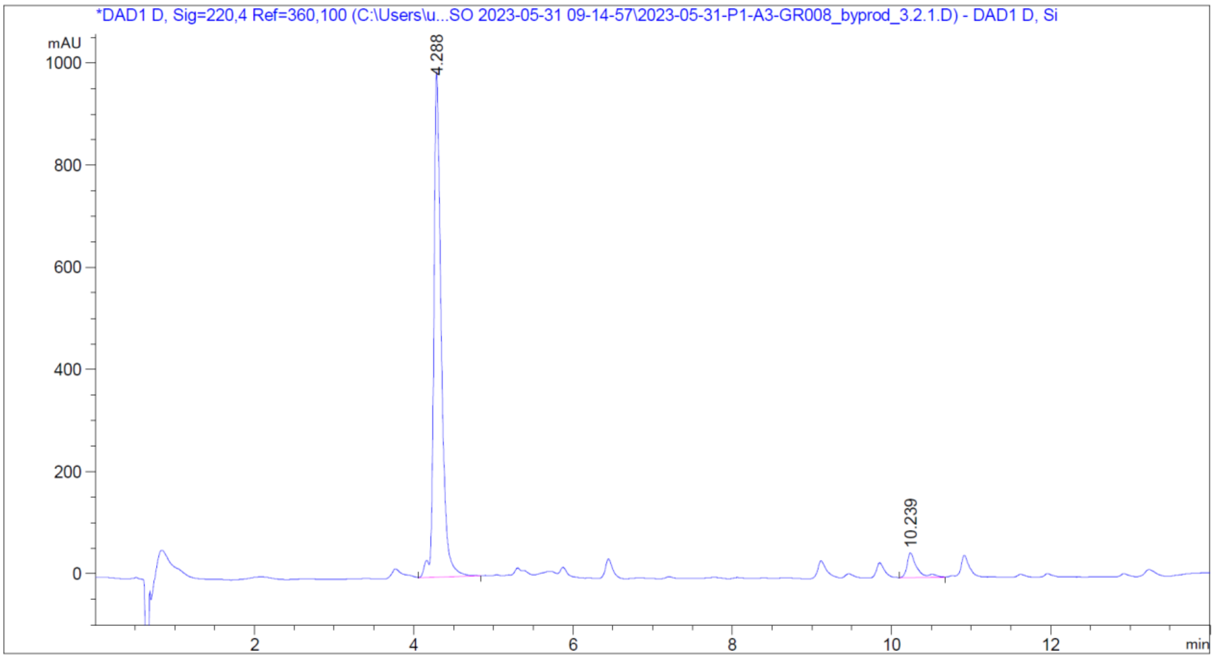

Peak #	RetTime [min]	Type	Width [min]	Area [mAU*s]	Height [mAU]	Area %
1	4.288	VV R	0.0962	6465.64160	989.78180	94.0617
2	10.239	BV R	0.1190	408.18530	48.08958	5.9383
Totals :				6873.82690	1037.87138	

ESI-MS (*m/z*)

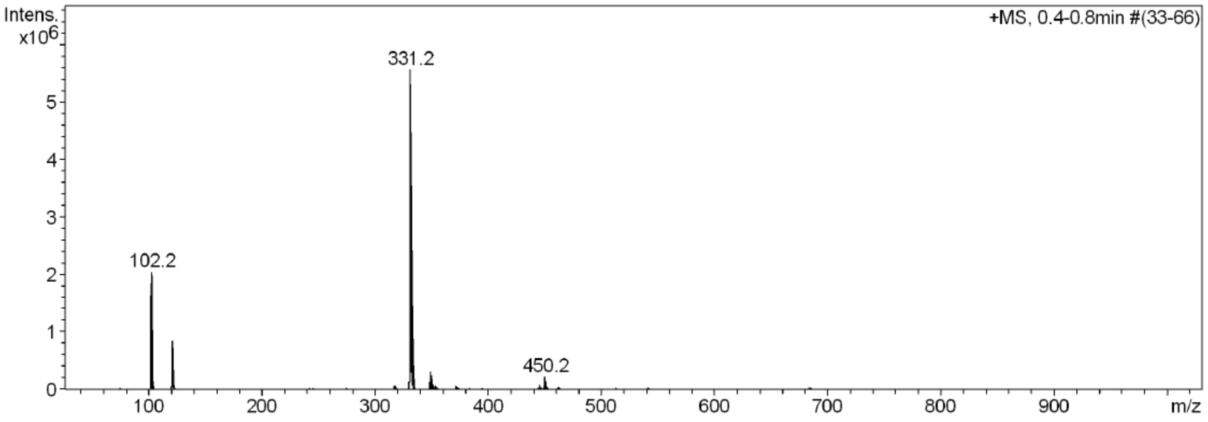

HRMS (*m/z*)

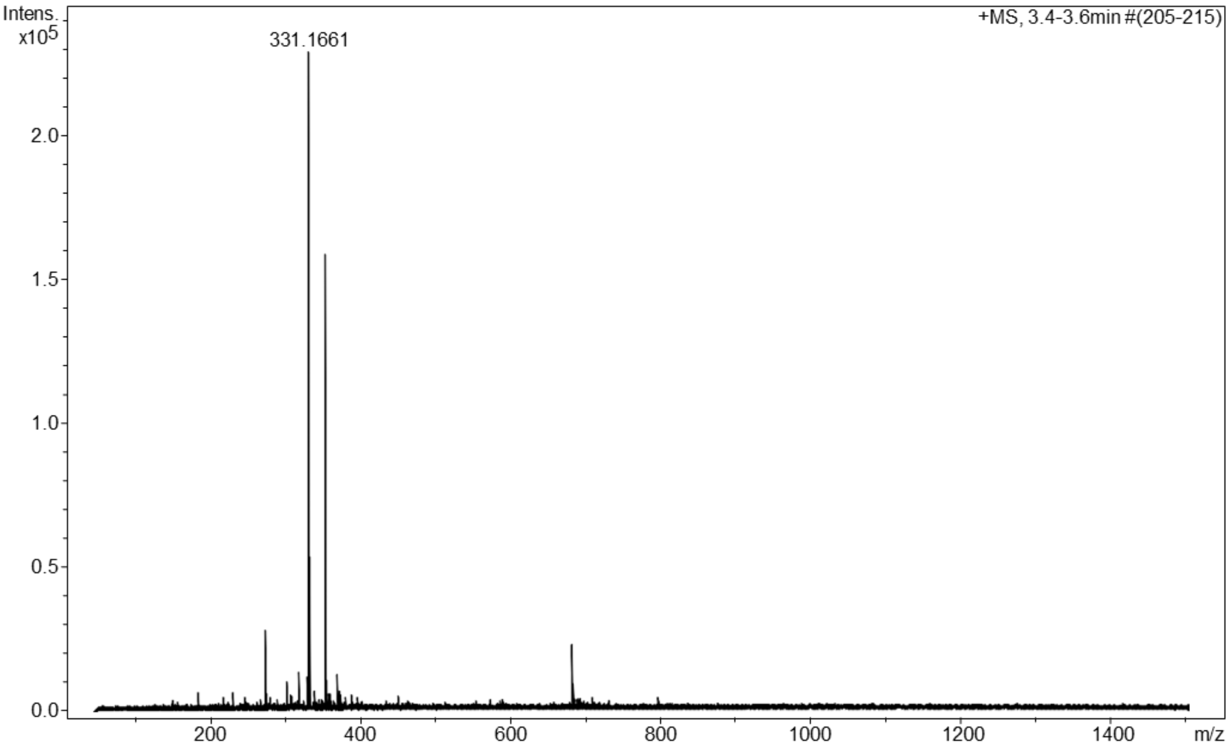

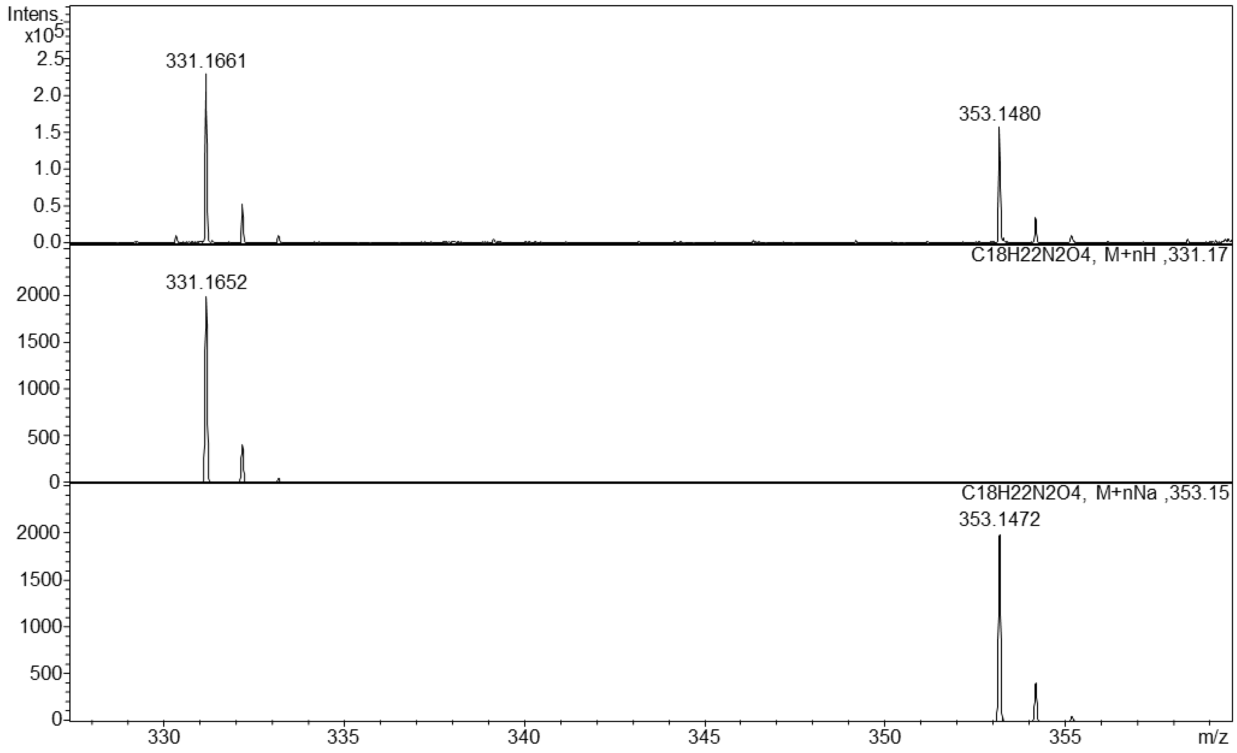

	Molecular formula	Calculated	Found
**[M+H]$^+$**	$C_{18}H_{23}N_2O_4$	331.1652	331.1661
**[M+Na]$^+$**	$C_{18}H_{22}N_2O_4Na$	353.1472	353.1480

2. Single-crystal X-ray diffraction reports

Methyl 8-(4-methoxybenzyl)-5-methyl-4-methylene-2-oxo-3,8-diazabicyclo[3.2.1]octane-6-carboxylate (20a)

ORTEP representation showing thermal ellipsoids at the 50% probability level:

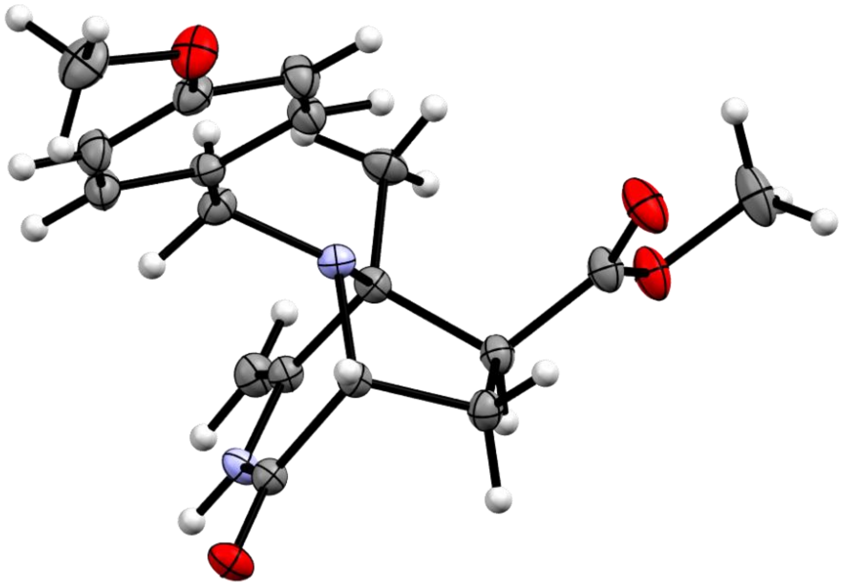

Crystal Structure Report for GR008

A colorless needle-like specimen of $C_{18}H_{22}N_2O_4$, approximate dimensions 0.170 mm x 0.170 mm x 0.400 mm, was used for the X-ray crystallographic analysis. The X-ray intensity data were measured on a D8 QUEST ECO three-circle diffractometer system equipped with a Ceramic x-ray tube (Mo Kα, λ = 0.71076 Å) and a doubly curved silicon crystal Bruker Triumph monochromator.

Table 1: Data collection details for GR008.

Axis	dx/mm	2θ/°	ω/°	φ/°	χ/°	Width/°	Frames	Time/s	Wavelength/Å	Voltage/kV	Current/mA	Temp./K
Omega	49.602	18.22	-161.98	-105.00	54.73	1.20	151	50.00	0.71076	50	20.0	101
Omega	49.602	18.22	-161.98	102.00	54.73	1.20	151	50.00	0.71076	50	20.0	101
Omega	49.602	18.22	-161.98	0.00	54.73	1.20	151	50.00	0.71076	50	20.0	101
Omega	49.602	18.22	-161.98	153.00	54.73	1.20	151	50.00	0.71076	50	20.0	101
Omega	49.602	18.22	-161.98	-54.00	54.73	1.20	151	50.00	0.71076	50	20.0	101
Omega	49.602	18.22	-161.98	-156.00	54.73	1.20	151	50.00	0.71076	50	20.0	101
Omega	49.602	18.22	-161.98	51.00	54.73	1.20	151	50.00	0.71076	50	20.0	101
Phi	49.602	0.00	0.00	0.00	54.73	1.20	300	50.00	0.71076	50	20.0	101

A total of 1357 frames were collected. The total exposure time was 18.85 hours. The frames were integrated with the Bruker SAINT software package using a narrow-frame algorithm. The integration of the data using a monoclinic unit cell yielded a total of 96887 reflections to a maximum θ angle of 30.53° (0.70 Å resolution), of which 5118 were independent (average redundancy 18.931, completeness = 99.1%, R_{int} = 2.69%, R_{sig} = 1.02%) and 4712 (92.07%) were greater than $2\sigma(F^2)$. The final cell constants of a = 9.264(3) Å, b = 7.8887(18) Å, c = 23.165(6) Å, β = 93.934(12)°, volume = 1688.9(7) Å^3, are based upon the refinement of the XYZ-centroids of 9679 reflections above 20 σ(I) with 5.458° < 2θ < 60.98°. Data were corrected for absorption effects using the Multi-Scan method (SADABS). The ratio of minimum to maximum apparent transmission was 0.956. The calculated minimum and maximum transmission coefficients (based on crystal size) are 0.9640 and 0.9840.

The structure was solved and refined using the Bruker SHELXTL Software Package, using the space group P 1 21/c 1, with Z = 4 for the formula unit, $C_{18}H_{22}N_2O_4$. The final anisotropic full-matrix least-squares refinement on F^2 with 220 variables converged at R1 = 4.24%, for the observed data and wR2 = 11.31% for all data. The goodness-of-fit was 1.098. The largest peak in the final difference electron density synthesis was 0.435 $e^-/Å^3$ and the largest hole was -0.218 $e^-/Å^3$ with an RMS deviation of 0.045 $e^-/Å^3$. On the basis of the final model, the calculated density was 1.299 g/cm^3 and F(000), 704 e^-.

Table 2. Sample and crystal data for GR001.

**Identification code**	GR001	
**Chemical formula**	$C_{18}H_{22}N_2O_4$	
**Formula weight**	330.37 g/mol	
**Temperature**	99(2) K	
**Wavelength**	0.71076 Å	
**Crystal size**	0.170 x 0.170 x 0.400 mm	
**Crystal habit**	colorless needle	
**Crystal system**	monoclinic	
**Space group**	P 1 21/c 1	
**Unit cell dimensions**	a = 9.264(3) Å	α = 90°
	b = 7.8887(18) Å	β = 93.934(12)°
	c = 23.165(6) Å	γ = 90°
**Volume**	1688.9(7) $Å^3$	
**Z**	4	
**Density (calculated)**	1.299 g/cm^3	
**Absorption coefficient**	0.092 mm^{-1}	
**F(000)**	704	

Table 3. Data collection and structure refinement for GR001.

**Diffractometer**	D8 QUEST ECO three-circle diffractometer	
**Radiation source**	Ceramic x-ray tube (Mo Kα, λ = 0.71076 Å)	
**Theta range for data collection**	2.92 to 30.53°	
**Index ranges**	-13<=h<=13, -11<=k<=11, -33<=l<=33	
**Reflections collected**	96887	
**Independent reflections**	5118 [R(int) = 0.0269]	
**Coverage of independent reflections**	99.1%	
**Absorption correction**	Multi-Scan	
**Max. and min. transmission**	0.9840 and 0.9640	
**Structure solution technique**	direct methods	
**Structure solution program**	SHELXT 2014/5 (Sheldrick, 2014)	
**Refinement method**	Full-matrix least-squares on F^2	
**Refinement program**	SHELXL-2017/1 (Sheldrick, 2017)	
**Function minimized**	$\Sigma w(F_o^2 - F_c^2)^2$	
**Data / restraints / parameters**	5118 / 0 / 220	
**Goodness-of-fit on F^2**	1.098	
**Δ/σ_{max}**	0.001	
**Final R indices**	4712 data; I>2σ(I)	R1 = 0.0424, wR2 = 0.1095
	all data	R1 = 0.0464, wR2 = 0.1131
**Weighting scheme**	$w=1/[\sigma^2(F_o^2)+(0.0499P)^2+0.7439P]$ where $P=(F_o^2+2F_c^2)/3$	
**Largest diff. peak and hole**	0.435 and -0.218 $eÅ^{-3}$	
**R.M.S. deviation from mean**	0.045 $eÅ^{-3}$	

Table 4. Atomic coordinates and equivalent isotropic atomic displacement parameters (Å^2) for GR001.

U(eq) is defined as one third of the trace of the orthogonalized U_{ij} tensor.

	x/a	y/b	z/c	U(eq)
C1	0.47704(10)	0.60322(11)	0.42762(4)	0.01458(16)
C2	0.49844(10)	0.77075(12)	0.45930(4)	0.01496(17)
N3	0.47115(9)	0.91123(10)	0.42674(3)	0.01580(16)
C4	0.42507(10)	0.91009(12)	0.36732(4)	0.01519(17)
C5	0.40400(10)	0.73357(12)	0.34138(4)	0.01529(17)
C6	0.26119(10)	0.65882(12)	0.36528(4)	0.01668(17)
C7	0.31480(10)	0.56291(13)	0.42086(4)	0.01814(18)
N8	0.51276(9)	0.61484(10)	0.36729(3)	0.01491(15)
C9	0.66569(10)	0.64901(13)	0.35978(4)	0.01817(18)
C10	0.75613(10)	0.49754(12)	0.37987(4)	0.01646(17)
C11	0.88697(11)	0.51502(13)	0.41217(5)	0.02060(19)
C12	0.97056(11)	0.37364(14)	0.42945(5)	0.0224(2)
C13	0.92252(11)	0.21192(13)	0.41371(5)	0.02050(19)
C14	0.78945(12)	0.19248(13)	0.38233(5)	0.0224(2)
C15	0.70800(11)	0.33361(13)	0.36590(4)	0.02004(19)
O16	0.99631(9)	0.06530(11)	0.42704(4)	0.02838(19)
C17	0.13613(12)	0.08215(17)	0.45623(6)	0.0310(3)
O18	0.53441(8)	0.77866(9)	0.51165(3)	0.01978(15)
C19	0.39684(11)	0.05621(13)	0.33976(4)	0.01986(19)
C20	0.40297(12)	0.73605(14)	0.27559(4)	0.02080(19)
C21	0.18195(11)	0.54352(13)	0.32135(4)	0.01974(19)
O22	0.20809(10)	0.39583(10)	0.31378(4)	0.02922(19)
O23	0.07780(8)	0.62989(10)	0.29072(4)	0.02393(17)
C24	0.99434(14)	0.53346(16)	0.24706(6)	0.0326(3)

Table 5. Bond lengths (Å) for GR001.

C1-N8	1.4611(12)	C1-C2	1.5181(13)
C1-C7	1.5337(14)	C1-H1	1.0
C2-O18	1.2369(12)	C2-N3	1.3545(12)
N3-C4	1.4130(12)	N3-H3	0.88
C4-C19	1.3348(13)	C4-C5	1.5239(13)
C5-N8	1.4730(12)	C5-C20	1.5235(14)
C5-C6	1.5828(14)	C6-C21	1.5163(14)
C6-C7	1.5456(14)	C6-H6	1.0
C7-H7A	0.99	C7-H7AB	0.99
N8-C9	1.4642(13)	C9-C10	1.5142(14)
C9-H9A	0.99	C9-H9AB	0.99
C10-C11	1.3870(14)	C10-C15	1.3987(14)
C11-C12	1.4005(15)	C11-H11	0.95
C12-C13	1.3916(15)	C12-H12	0.95
C13-O16	1.3681(13)	C13-C14	1.3959(15)
C14-C15	1.3836(15)	C14-H14	0.95
C15-H15	0.95	O16-C17	1.4257(14)
C17-H17A	0.98	C17-H17B	0.98
C17-H17C	0.98	C19-H19A	0.95
C19-H19B	0.95	C20-H20A	0.98
C20-H20B	0.98	C20-H20C	0.98

C21-O22	1.2053(13)	C21-O23	1.3434(12)
O23-C24	1.4464(13)	C24-H24A	0.98
C24-H24B	0.98	C24-H24C	0.98

Table 6. Bond angles (°) for GR001.

N8-C1-C2	112.16(7)	N8-C1-C7	101.53(7)
C2-C1-C7	108.93(8)	N8-C1-H1	111.3
C2-C1-H1	111.3	C7-C1-H1	111.3
O18-C2-N3	122.15(9)	O18-C2-C1	122.36(8)
N3-C2-C1	115.46(8)	C2-N3-C4	124.73(8)
C2-N3-H3	117.6	C4-N3-H3	117.6
C19-C4-N3	119.78(9)	C19-C4-C5	125.79(9)
N3-C4-C5	114.33(8)	N8-C5-C20	111.90(8)
N8-C5-C4	110.78(7)	C20-C5-C4	112.00(8)
N8-C5-C6	100.68(7)	C20-C5-C6	114.00(8)
C4-C5-C6	106.86(7)	C21-C6-C7	112.24(8)
C21-C6-C5	111.50(8)	C7-C6-C5	104.19(7)
C21-C6-H6	109.6	C7-C6-H6	109.6
C5-C6-H6	109.6	C1-C7-C6	103.91(7)
C1-C7-H7A	111.0	C6-C7-H7A	111.0
C1-C7-H7AB	111.0	C6-C7-H7AB	111.0
H7A-C7-H7AB	109.0	C1-N8-C9	114.18(8)
C1-N8-C5	103.66(7)	C9-N8-C5	118.35(8)
N8-C9-C10	109.74(8)	N8-C9-H9A	109.7
C10-C9-H9A	109.7	N8-C9-H9AB	109.7
C10-C9-H9AB	109.7	H9A-C9-H9AB	108.2
C11-C10-C15	118.01(9)	C11-C10-C9	122.14(9)
C15-C10-C9	119.84(9)	C10-C11-C12	121.38(9)
C10-C11-H11	119.3	C12-C11-H11	119.3
C13-C12-C11	119.64(9)	C13-C12-H12	120.2
C11-C12-H12	120.2	O16-C13-C12	124.76(9)
O16-C13-C14	115.75(9)	C12-C13-C14	119.49(9)
C15-C14-C13	120.03(10)	C15-C14-H14	120.0
C13-C14-H14	120.0	C14-C15-C10	121.41(9)
C14-C15-H15	119.3	C10-C15-H15	119.3
C13-O16-C17	116.86(9)	O16-C17-H17A	109.5
O16-C17-H17B	109.5	H17A-C17-H17B	109.5
O16-C17-H17C	109.5	H17A-C17-H17C	109.5
H17B-C17-H17C	109.5	C4-C19-H19A	120.0
C4-C19-H19B	120.0	H19A-C19-H19B	120.0
C5-C20-H20A	109.5	C5-C20-H20B	109.5
H20A-C20-H20B	109.5	C5-C20-H20C	109.5
H20A-C20-H20C	109.5	H20B-C20-H20C	109.5
O22-C21-O23	123.89(10)	O22-C21-C6	125.78(9)
O23-C21-C6	110.32(9)	C21-O23-C24	115.64(9)
O23-C24-H24A	109.5	O23-C24-H24B	109.5
H24A-C24-H24B	109.5	O23-C24-H24C	109.5
H24A-C24-H24C	109.5	H24B-C24-H24C	109.5

Table 7. Torsion angles (°) for GR001.

N8-C1-C2-O18	-148.82(9)	C7-C1-C2-O18	99.59(10)
N8-C1-C2-N3	33.13(11)	C7-C1-C2-N3	-78.45(10)
O18-C2-N3-C4	-177.77(9)	C1-C2-N3-C4	0.28(13)
C2-N3-C4-C19	177.89(9)	C2-N3-C4-C5	1.30(13)
C19-C4-C5-N8	147.92(10)	N3-C4-C5-N8	-35.73(11)
C19-C4-C5-C20	22.22(13)	N3-C4-C5-C20	-161.43(8)
C19-C4-C5-C6	-103.28(11)	N3-C4-C5-C6	73.07(10)
N8-C5-C6-C21	-96.92(9)	C20-C5-C6-C21	23.04(11)
C4-C5-C6-C21	147.32(8)	N8-C5-C6-C7	24.36(9)
C20-C5-C6-C7	144.32(8)	C4-C5-C6-C7	-91.39(8)
N8-C1-C7-C6	-33.33(9)	C2-C1-C7-C6	85.15(9)
C21-C6-C7-C1	125.99(8)	C5-C6-C7-C1	5.20(9)
C2-C1-N8-C9	65.33(10)	C7-C1-N8-C9	-178.53(8)
C2-C1-N8-C5	-64.83(9)	C7-C1-N8-C5	51.31(9)
C20-C5-N8-C1	-168.44(8)	C4-C5-N8-C1	65.80(9)
C6-C5-N8-C1	-46.98(8)	C20-C5-N8-C9	63.95(11)
C4-C5-N8-C9	-61.81(10)	C6-C5-N8-C9	-174.59(8)
C1-N8-C9-C10	67.76(10)	C5-N8-C9-C10	-169.79(8)
N8-C9-C10-C11	-137.91(9)	N8-C9-C10-C15	42.02(12)
C15-C10-C11-C12	1.30(15)	C9-C10-C11-C12	-178.76(9)
C10-C11-C12-C13	0.45(16)	C11-C12-C13-O16	178.55(10)
C11-C12-C13-C14	-1.89(16)	O16-C13-C14-C15	-178.83(10)
C12-C13-C14-C15	1.57(16)	C13-C14-C15-C10	0.21(16)
C11-C10-C15-C14	-1.63(15)	C9-C10-C15-C14	178.43(9)
C12-C13-O16-C17	-3.84(16)	C14-C13-O16-C17	176.58(10)
C7-C6-C21-O22	-33.03(15)	C5-C6-C21-O22	83.44(13)
C7-C6-C21-O23	147.85(9)	C5-C6-C21-O23	-95.67(10)
O22-C21-O23-C24	0.69(17)	C6-C21-O23-C24	179.82(10)

Table 8. Anisotropic atomic displacement parameters (Å^2) for GR001.

The anisotropic atomic displacement factor exponent takes the form: $-2\pi^2[h^2 a^{*2} U_{11} + ... + 2 h k a^* b^* U_{12}]$

	U_{11}	U_{22}	U_{33}	U_{23}	U_{13}	U_{12}
C1	0.0169(4)	0.0123(4)	0.0145(4)	0.0012(3)	0.0009(3)	0.0002(3)
C2	0.0156(4)	0.0134(4)	0.0158(4)	0.0014(3)	0.0011(3)	0.0007(3)
N3	0.0215(4)	0.0113(3)	0.0143(3)	0.0004(3)	-0.0009(3)	0.0005(3)
C4	0.0163(4)	0.0145(4)	0.0147(4)	0.0016(3)	0.0003(3)	-0.0002(3)
C5	0.0171(4)	0.0138(4)	0.0147(4)	0.0006(3)	-0.0002(3)	0.0006(3)
C6	0.0164(4)	0.0141(4)	0.0193(4)	-0.0003(3)	-0.0005(3)	-0.0006(3)
C7	0.0182(4)	0.0173(4)	0.0189(4)	0.0023(3)	0.0008(3)	-0.0025(3)
N8	0.0160(3)	0.0147(3)	0.0140(3)	0.0011(3)	0.0012(3)	0.0016(3)
C9	0.0173(4)	0.0171(4)	0.0204(4)	0.0015(3)	0.0037(3)	0.0002(3)
C10	0.0162(4)	0.0171(4)	0.0163(4)	-0.0008(3)	0.0028(3)	0.0006(3)
C11	0.0170(4)	0.0182(4)	0.0266(5)	-0.0029(4)	0.0013(3)	-0.0019(3)
C12	0.0141(4)	0.0243(5)	0.0285(5)	-0.0017(4)	-0.0007(3)	-0.0009(4)
C13	0.0174(4)	0.0195(4)	0.0249(5)	0.0014(4)	0.0038(3)	0.0027(3)
C14	0.0227(5)	0.0162(4)	0.0281(5)	-0.0031(4)	0.0000(4)	-0.0010(4)
C15	0.0196(4)	0.0188(4)	0.0212(4)	-0.0027(3)	-0.0020(3)	-0.0003(3)
O16	0.0217(4)	0.0217(4)	0.0416(5)	0.0039(3)	0.0010(3)	0.0059(3)
C17	0.0202(5)	0.0338(6)	0.0390(6)	0.0046(5)	0.0021(4)	0.0090(4)
O18	0.0283(4)	0.0158(3)	0.0147(3)	0.0007(2)	-0.0022(3)	0.0016(3)

	U_{11}	U_{22}	U_{33}	U_{23}	U_{13}	U_{12}
C19	0.0237(4)	0.0162(4)	0.0194(4)	0.0042(3)	-0.0002(3)	-0.0005(3)
C20	0.0260(5)	0.0224(5)	0.0138(4)	0.0003(3)	-0.0005(3)	0.0013(4)
C21	0.0190(4)	0.0167(4)	0.0228(4)	0.0010(3)	-0.0034(3)	-0.0007(3)
O22	0.0327(4)	0.0166(4)	0.0366(4)	-0.0045(3)	-0.0105(3)	0.0023(3)
O23	0.0220(4)	0.0179(3)	0.0302(4)	0.0009(3)	-0.0102(3)	-0.0010(3)
C24	0.0295(5)	0.0281(6)	0.0376(6)	-0.0019(5)	-0.0162(5)	-0.0052(5)

Table 9. Hydrogen atomic coordinates and isotropic atomic displacement parameters (Å^2) for GR001.

	x/a	y/b	z/c	U(eq)
H1	0.5323	0.5100	0.4483	0.017
H3	0.4830	1.0104	0.4438	0.019
H6	0.1961	0.7538	0.3754	0.02
H7A	0.2650	0.6040	0.4546	0.022
H7AB	0.2983	0.4395	0.4165	0.022
H9A	0.6971	0.7504	0.3825	0.022
H9AB	0.6793	0.6721	0.3185	0.022
H11	0.9206	0.6252	0.4228	0.025
H12	1.0596	0.3881	0.4518	0.027
H14	0.7548	0.0823	0.3723	0.027
H15	0.6175	0.3188	0.3447	0.024
H17A	1.1787	-0.0305	0.4630	0.046
H17B	1.1275	0.1393	0.4934	0.046
H17C	1.1983	0.1492	0.4324	0.046
H19A	0.4078	1.1605	0.3601	0.024
H19B	0.3658	1.0559	0.2998	0.024
H20A	0.4901	0.7935	0.2639	0.031
H20B	0.3170	0.7967	0.2596	0.031
H20C	0.4012	0.6195	0.2609	0.031
H24A	-0.0595	0.4442	0.2657	0.049
H24B	0.0597	0.4820	0.2205	0.049
H24C	-0.0737	0.6089	0.2254	0.049

Methyl 5-(4-methoxybenzyl)-1-methyl-6-methylene-3-oxo-2,5-diazabicyclo[2.2.2]octane-7-carboxylate (21a)

ORTEP representation showing thermal ellipsoids at the 50% probability level:

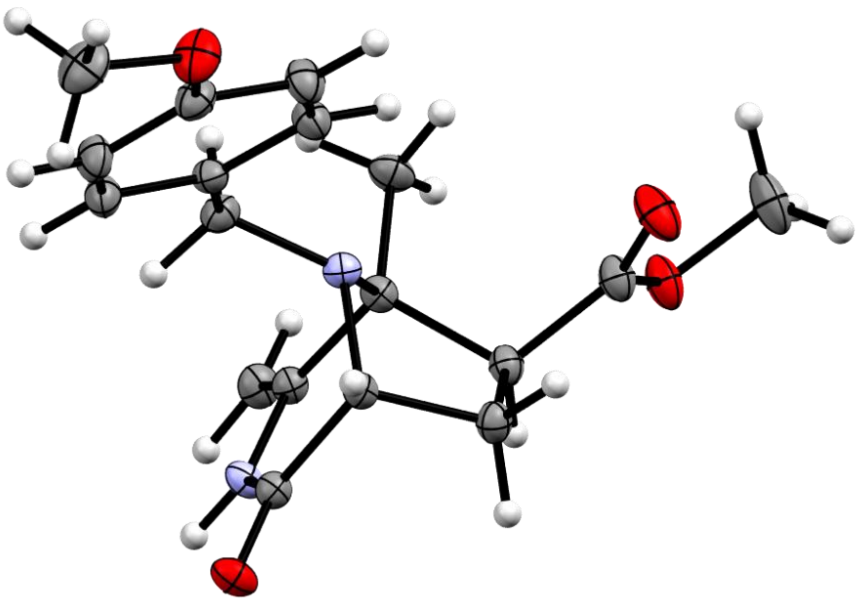

Crystal Structure Report for GR008_222

A colorless, prism-like specimen of $C_{18}H_{22}N_2O_4$, approximate dimensions 0.130 mm x 0.250 mm x 0.440 mm, was used for the X-ray crystallographic analysis.The X-ray intensity data were measured on a D8 QUEST ECO three-circle diffractometer system equipped with a Ceramic x-ray tube (Mo Kα, λ = 0.71073 Å) and a doubly curved silicon crystal Bruker Triumph monochromator.

Table 1: Data collection details for GR008_222.

Axis	dx/mm	2θ/°	ω/°	φ/°	χ/°	Width/°	Frames	Time/s	Wavelength/Å	Voltage/kV	Current/mA	Temp./K
Omega	39.533	6.06	-9.00	0.00	54.76	0.70	214	1.00	0.71076	50	20.0	180
Phi	39.533	6.06	-2.94	0.00	54.76	0.70	514	10.00	0.71076	50	20.0	180

A total of 728 frames were collected. The total exposure time was 1.49 hours. The frames were integrated with the Bruker SAINT software package using a narrow-frame algorithm. The integration of the data using an orthorhombic unit cell yielded a total of 25992 reflections to a maximum θ angle of 28.31° (0.75 Å resolution), of which 4187 were independent (average redundancy 6.208, completeness = 99.4%, R_{int} = 2.85%, R_{sig} = 2.08%) and 4020 (96.01%) were greater than $2\sigma(F^2)$.The final cell constants of a = 8.3896(3) Å, b = 25.8498(9) Å, c = 7.7856(3) Å, volume = 1688.46(11) $Å^3$, are based upon the refinement of the XYZ-centroids of 9924 reflections above 20 σ(I) with 5.464° < 2θ < 56.57°.Data were corrected for absorption effects using the Multi-Scan method (SADABS). The ratio of minimum to maximum apparent transmission was 0.951. The calculated minimum and maximum transmission coefficients (based on crystal size) are 0.9600 and 0.9880.

The structure was solved and refined using the Bruker SHELXTL Software Package, using the space group P n a 21, with Z = 4 for the formula unit, $C_{18}H_{22}N_2O_4$.The final anisotropic full-matrix least-squares refinement on F^2 with 224 variables converged at R1 = 3.74%, for the observed data and wR2 = 9.50% for all data. The goodness-of-fit was 1.116. The largest peak in the final difference electron density synthesis was 0.259 $e^-/Å^3$ and the largest hole was -0.222 $e^-/Å^3$ with an RMS deviation of 0.040 $e^-/Å^3$. On the basis of the final model, the calculated density was 1.300 g/cm^3 and F(000), 704 e^-.

Table 2. Sample and crystal data for GR008_222.

**Identification code**	GR008_222	
**Chemical formula**	$C_{18}H_{22}N_2O_4$	
**Formula weight**	330.37 g/mol	
**Temperature**	180(2) K	
**Wavelength**	0.71073 Å	
**Crystal size**	0.130 x 0.250 x 0.440 mm	
**Crystal habit**	colorless prism	
**Crystal system**	orthorhombic	
**Space group**	P n a 21	
**Unit cell dimensions**	a = 8.3896(3) Å	α = 90°
	b = 25.8498(9) Å	β = 90°
	c = 7.7856(3) Å	γ = 90°
**Volume**	1688.46(11) $Å^3$	
**Z**	4	
**Density (calculated)**	1.300 g/cm^3	
**Absorption coefficient**	0.092 mm^{-1}	
**F(000)**	704	

Table 3. Data collection and structure refinement for GR008_222.

**Diffractometer**	D8 QUEST ECO three-circle diffractometer	
**Radiation source**	Ceramic x-ray tube (Mo Kα, λ = 0.71073 Å)	
**Theta range for data collection**	2.73 to 28.31°	
**Index ranges**	-11<=h<=11, -34<=k<=34, -10<=l<=10	
**Reflections collected**	25992	
**Independent reflections**	4187 [R(int) = 0.0285]	
**Coverage of independent reflections**	99.4%	
**Absorption correction**	Multi-Scan	
**Max. and min. transmission**	0.9880 and 0.9600	
**Structure solution technique**	direct methods	
**Structure solution program**	XT, VERSION 2018/2	
**Refinement method**	Full-matrix least-squares on F^2	
**Refinement program**	SHELXL-2019/1 (Sheldrick, 2019)	
**Function minimized**	Σ w(F_o^2 - F_c^2)^2	
**Data / restraints / parameters**	4187 / 1 / 224	
**Goodness-of-fit on F^2**	1.116	
**Final R indices**	4020 data; I>2σ(I)	R1 = 0.0374, wR2 = 0.0934
	all data	R1 = 0.0394, wR2 = 0.0950
**Weighting scheme**	w=1/[$\sigma^2(F_o^2)+(0.0506P)^2+0.3236P$]<br>where P=($F_o^2+2F_c^2$)/3	
**Absolute structure parameter**	0.1(2)	
**Largest diff. peak and hole**	0.259 and -0.222 e$Å^{-3}$	
**R.M.S. deviation from mean**	0.040 e$Å^{-3}$	

Table 4. Atomic coordinates and equivalent isotropic atomic displacement parameters ($Å^2$) for GR008_222.

U(eq) is defined as one third of the trace of the orthogonalized U_{ij} tensor.

	x/a	y/b	z/c	U(eq)
C9	0.4737(3)	0.58736(8)	0.3078(3)	0.0293(4)
O12	0.2534(3)	0.70772(7)	0.4667(3)	0.0656(7)

O13	0.0963(2)	0.73084(5)	0.2522(2)	0.0362(4)
O15	0.95741(17)	0.49605(5)	0.42248(17)	0.0248(3)
O23	0.87037(18)	0.67266(6)	0.9292(2)	0.0366(4)
N2	0.0793(2)	0.55555(6)	0.2492(2)	0.0215(3)
N5	0.2720(2)	0.57507(7)	0.5271(2)	0.0253(4)
C1	0.1807(2)	0.60266(7)	0.2528(2)	0.0208(3)
C3	0.0366(2)	0.53580(7)	0.4015(2)	0.0202(3)
C4	0.0983(2)	0.57010(7)	0.5461(2)	0.0236(4)
C6	0.3231(2)	0.58876(7)	0.3638(2)	0.0226(4)
C7	0.0745(2)	0.64443(7)	0.3437(3)	0.0247(4)
C8	0.0235(3)	0.62359(8)	0.5214(3)	0.0312(5)
C10	0.2216(3)	0.61797(8)	0.0697(3)	0.0281(4)
C11	0.1548(3)	0.69681(7)	0.3621(3)	0.0279(4)
C14	0.1520(3)	0.78353(8)	0.2743(4)	0.0427(6)
C16	0.3779(3)	0.54841(8)	0.6456(3)	0.0305(5)
C17	0.5048(2)	0.58345(7)	0.7223(2)	0.0236(4)
C18	0.6322(3)	0.56172(8)	0.8116(3)	0.0281(4)
C19	0.7500(3)	0.59213(8)	0.8824(3)	0.0311(4)
C20	0.7457(2)	0.64553(8)	0.8594(3)	0.0257(4)
C21	0.6223(2)	0.66776(7)	0.7686(3)	0.0268(4)
C22	0.5010(3)	0.63665(8)	0.7021(3)	0.0279(4)
C24	0.8824(3)	0.72587(10)	0.8855(4)	0.0460(6)

Table 5. Bond lengths (Å) for GR008_222.

C9-C6	1.337(3)	C9-H9A	0.950000
C9-H9B	0.950000	O12-C11	1.195(3)
O13-C11	1.322(3)	O13-C14	1.450(3)
O15-C3	1.234(2)	O23-C20	1.371(2)
O23-C24	1.420(3)	N2-C3	1.339(2)
N2-C1	1.486(2)	N2-H2	0.82(3)
N5-C6	1.387(2)	N5-C16	1.455(3)
N5-C4	1.471(3)	C1-C6	1.518(3)
C1-C10	1.519(3)	C1-C7	1.568(3)
C3-C4	1.523(2)	C4-C8	1.530(3)
C4-H4	1.000000	C7-C11	1.519(3)
C7-C8	1.545(3)	C7-H7	1.000000
C8-H8A	0.990000	C8-H8B	0.990000
C10-H10A	0.980000	C10-H10B	0.980000
C10-H10C	0.980000	C14-H14A	0.980000
C14-H14B	0.980000	C14-H14C	0.980000
C16-C17	1.520(3)	C16-H16A	0.990000
C16-H16B	0.990000	C17-C22	1.384(3)
C17-C18	1.393(3)	C18-C19	1.377(3)
C18-H18	0.950000	C19-C20	1.392(3)
C19-H19	0.950000	C20-C21	1.379(3)
C21-C22	1.396(3)	C21-H21	0.950000
C22-H22	0.950000	C24-H24A	0.980000
C24-H24B	0.980000	C24-H24C	0.980000

Table 6. Bond angles (°) for GR008_222.

C6-C9-H9A	120.000000	C6-C9-H9B	120.000000
H9A-C9-H9B	120.000000	C11-O13-C14	115.36(19)
C20-O23-C24	117.03(18)	C3-N2-C1	116.68(15)
C3-N2-H2	122.8(19)	C1-N2-H2	119.7(19)
C6-N5-C16	120.88(19)	C6-N5-C4	114.90(16)
C16-N5-C4	120.00(16)	N2-C1-C6	105.51(14)
N2-C1-C10	108.97(15)	C6-C1-C10	114.74(16)
N2-C1-C7	104.33(15)	C6-C1-C7	110.70(15)
C10-C1-C7	111.84(16)	O15-C3-N2	125.35(17)
O15-C3-C4	124.69(16)	N2-C3-C4	109.95(15)
N5-C4-C3	108.22(15)	N5-C4-C8	108.32(16)
C3-C4-C8	107.10(16)	N5-C4-H4	111.000000
C3-C4-H4	111.000000	C8-C4-H4	111.000000
C9-C6-N5	125.74(19)	C9-C6-C1	124.37(18)
N5-C6-C1	109.79(16)	C11-C7-C8	110.41(17)
C11-C7-C1	113.87(17)	C8-C7-C1	108.72(15)
C11-C7-H7	107.900000	C8-C7-H7	107.900000
C1-C7-H7	107.900000	C4-C8-C7	108.31(16)
C4-C8-H8A	110.000000	C7-C8-H8A	110.000000
C4-C8-H8B	110.000000	C7-C8-H8B	110.000000
H8A-C8-H8B	108.400000	C1-C10-H10A	109.500000
C1-C10-H10B	109.500000	H10A-C10-H10B	109.500000
C1-C10-H10C	109.500000	H10A-C10-H10C	109.500000
H10B-C10-H10C	109.500000	O12-C11-O13	122.78(19)
O12-C11-C7	125.59(19)	O13-C11-C7	111.55(17)
O13-C14-H14A	109.500000	O13-C14-H14B	109.500000
H14A-C14-H14B	109.500000	O13-C14-H14C	109.500000
H14A-C14-H14C	109.500000	H14B-C14-H14C	109.500000
N5-C16-C17	113.24(16)	N5-C16-H16A	108.900000
C17-C16-H16A	108.900000	N5-C16-H16B	108.900000
C17-C16-H16B	108.900000	H16A-C16-H16B	107.700000
C22-C17-C18	118.34(18)	C22-C17-C16	122.09(18)
C18-C17-C16	119.54(18)	C19-C18-C17	121.30(18)
C19-C18-H18	119.300000	C17-C18-H18	119.300000
C18-C19-C20	119.76(19)	C18-C19-H19	120.100000
C20-C19-H19	120.100000	O23-C20-C21	124.24(18)
O23-C20-C19	115.90(19)	C21-C20-C19	119.85(18)
C20-C21-C22	119.82(18)	C20-C21-H21	120.100000
C22-C21-H21	120.100000	C17-C22-C21	120.88(18)
C17-C22-H22	119.600000	C21-C22-H22	119.600000
O23-C24-H24A	109.500000	O23-C24-H24B	109.500000
H24A-C24-H24B	109.500000	O23-C24-H24C	109.500000
H24A-C24-H24C	109.500000	H24B-C24-H24C	109.500000

Table 7. Torsion angles (°) for GR008_222.

C3-N2-C1-C6	54.2(2)	C3-N2-C1-C10	177.89(17)
C3-N2-C1-C7	-62.5(2)	C1-N2-C3-O15	-177.52(18)
C1-N2-C3-C4	3.3(2)	C6-N5-C4-C3	49.9(2)
C16-N5-C4-C3	-107.25(19)	C6-N5-C4-C8	-65.9(2)
C16-N5-C4-C8	136.96(18)	O15-C3-C4-N5	124.4(2)

N2-C3-C4-N5	-56.4(2)	O15-C3-C4-C8	-119.0(2)
N2-C3-C4-C8	60.2(2)	C16-N5-C6-C9	-11.5(3)
C4-N5-C6-C9	-168.35(18)	C16-N5-C6-C1	165.11(16)
C4-N5-C6-C1	8.2(2)	N2-C1-C6-C9	117.06(19)
C10-C1-C6-C9	-2.9(2)	C7-C1-C6-C9	-130.64(19)
N2-C1-C6-N5	-59.56(19)	C10-C1-C6-N5	-179.52(16)
C7-C1-C6-N5	52.74(19)	N2-C1-C7-C11	-179.98(16)
C6-C1-C7-C11	67.0(2)	C10-C1-C7-C11	-62.3(2)
N2-C1-C7-C8	56.47(19)	C6-C1-C7-C8	-56.6(2)
C10-C1-C7-C8	174.11(17)	N5-C4-C8-C7	56.8(2)
C3-C4-C8-C7	-59.7(2)	C11-C7-C8-C4	-124.38(18)
C1-C7-C8-C4	1.2(2)	C14-O13-C11-O12	-4.1(4)
C14-O13-C11-C7	172.9(2)	C8-C7-C11-O12	45.0(3)
C1-C7-C11-O12	-77.6(3)	C8-C7-C11-O13	-131.81(19)
C1-C7-C11-O13	105.6(2)	C6-N5-C16-C17	74.5(2)
C4-N5-C16-C17	-129.80(19)	N5-C16-C17-C22	10.2(3)
N5-C16-C17-C18	-167.87(19)	C22-C17-C18-C19	1.6(3)
C16-C17-C18-C19	179.7(2)	C17-C18-C19-C20	-2.4(3)
C24-O23-C20-C21	-8.4(3)	C24-O23-C20-C19	170.5(2)
C18-C19-C20-O23	-177.9(2)	C18-C19-C20-C21	1.1(3)
O23-C20-C21-C22	179.8(2)	C19-C20-C21-C22	0.9(3)
C18-C17-C22-C21	0.4(3)	C16-C17-C22-C21	-177.6(2)
C20-C21-C22-C17	-1.7(3)		

Table 8. Anisotropic atomic displacement parameters (Å^2) for GR008_222.

The anisotropic atomic displacement factor exponent takes the form: $-2\pi^2[h^2 a^{*2} U_{11} + ... + 2 h k a^* b^* U_{12}]$

	U_{11}	U_{22}	U_{33}	U_{23}	U_{13}	U_{12}
C9	0.0294(10)	0.0276(9)	0.0309(10)	-0.0001(8)	0.0006(8)	-0.0046(7)
O12	0.0881(16)	0.0258(8)	0.0828(16)	0.0077(9)	-0.0537(14)	-0.0106(9)
O13	0.0469(9)	0.0205(7)	0.0413(9)	0.0040(6)	-0.0102(8)	-0.0021(6)
O15	0.0312(7)	0.0219(6)	0.0213(6)	0.0001(5)	0.0010(6)	-0.0089(5)
O23	0.0288(7)	0.0369(9)	0.0442(9)	0.0020(7)	-0.0125(7)	-0.0079(6)
N2	0.0282(8)	0.0196(7)	0.0167(7)	-0.0007(6)	-0.0006(6)	-0.0069(6)
N5	0.0303(8)	0.0261(8)	0.0196(7)	0.0034(6)	-0.0053(7)	-0.0097(7)
C1	0.0274(9)	0.0164(7)	0.0186(8)	0.0006(6)	-0.0004(7)	-0.0054(7)
C3	0.0227(8)	0.0195(8)	0.0182(8)	-0.0005(6)	-0.0007(7)	-0.0021(6)
C4	0.0316(10)	0.0236(8)	0.0156(8)	-0.0023(7)	0.0019(7)	-0.0075(7)
C6	0.0288(9)	0.0170(7)	0.0219(8)	-0.0016(6)	-0.0021(8)	-0.0052(7)
C7	0.0280(9)	0.0182(8)	0.0280(10)	-0.0012(7)	-0.0009(8)	-0.0009(7)
C8	0.0406(12)	0.0251(9)	0.0279(10)	-0.0056(8)	0.0092(9)	-0.0012(8)
C10	0.0355(11)	0.0290(10)	0.0200(9)	0.0053(7)	0.0003(8)	-0.0072(8)
C11	0.0314(10)	0.0187(8)	0.0336(10)	-0.0003(8)	-0.0046(9)	-0.0004(7)
C14	0.0561(15)	0.0189(9)	0.0532(16)	0.0041(10)	-0.0056(13)	-0.0029(9)
C16	0.0427(12)	0.0217(9)	0.0271(10)	0.0023(8)	-0.0105(9)	-0.0075(8)
C17	0.0293(10)	0.0220(8)	0.0195(8)	-0.0010(7)	-0.0016(8)	-0.0016(7)
C18	0.0349(10)	0.0210(8)	0.0283(10)	0.0047(7)	-0.0014(9)	0.0009(7)
C19	0.0272(10)	0.0321(10)	0.0341(11)	0.0076(9)	-0.0061(9)	0.0045(8)
C20	0.0232(9)	0.0301(9)	0.0239(9)	-0.0025(7)	-0.0016(7)	-0.0008(7)
C21	0.0323(10)	0.0187(8)	0.0294(10)	-0.0038(7)	-0.0061(8)	0.0014(7)
C22	0.0303(10)	0.0225(9)	0.0310(10)	-0.0021(8)	-0.0102(9)	0.0024(8)
C24	0.0461(13)	0.0439(14)	0.0480(15)	0.0057(11)	-0.0134(12)	-0.0230(11)

Table 9. Hydrogen atomic coordinates and isotropic atomic displacement parameters (Å^2) for GR008_222.

	x/a	y/b	z/c	U(eq)
H9A	0.5566	0.5762	0.3824	0.035000
H9B	0.4979	0.5975	0.1935	0.035000
H2	0.065(3)	0.5404(11)	0.159(4)	0.028(7)
H4	0.0699	0.5554	0.6609	0.028000
H7	-0.0239	0.6492	0.2731	0.030000
H8A	0.0602	0.6473	0.6130	0.037000
H8B	-0.0941	0.6211	0.5276	0.037000
H10A	0.2884	0.6491	0.0710	0.042000
H10B	0.1232	0.6251	0.0061	0.042000
H10C	0.2795	0.5896	0.0139	0.042000
H14A	0.1106	0.8051	0.1809	0.064000
H14B	0.2688	0.7841	0.2721	0.064000
H14C	0.1142	0.7970	0.3847	0.064000
H16A	0.4308	0.5196	0.5843	0.037000
H16B	0.3137	0.5333	0.7398	0.037000
H18	0.6380	0.5252	0.8239	0.034000
H19	0.8337	0.5767	0.9466	0.037000
H21	0.6198	0.7041	0.7513	0.032000
H22	0.4148	0.6522	0.6423	0.034000
H24A	0.9801	0.7403	0.9350	0.069000
H24B	0.7899	0.7445	0.9311	0.069000
H24C	0.8854	0.7295	0.7602	0.069000

8-(4-methoxybenzyl)-7,7a-dimethyl-1,3,4,4a-tetrahydro-3,7-epiminofuro[3,4-*b*]pyridine-2,5-dione (23)

ORTEP representation showing thermal ellipsoids at the 50% probability level:

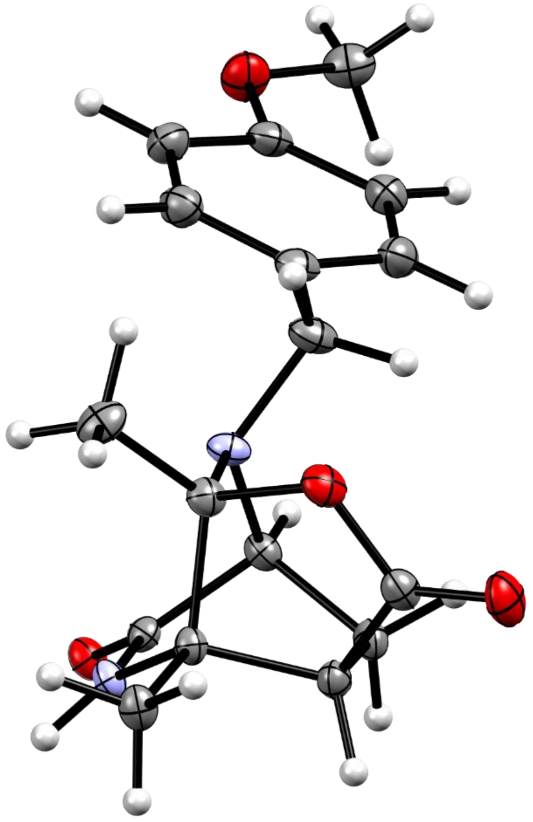

Crystal Structure Report for GR024

A colorless, block-like specimen of $C_{17}H_{20}N_2O_4$, approximate dimensions 0.180 mm x 0.180 mm x 0.230 mm, was used for the X-ray crystallographic analysis. The X-ray intensity data were measured on a D8 QUEST ECO three-circle diffractometer system equipped with a Ceramic x-ray tube (Mo Kα, λ = 0.71073 Å) and a doubly curved silicon crystal Bruker Triumph monochromator.

Table 1: Data collection details for GR024.

Axis	dx/mm	2θ/°	ω/°	φ/°	χ/°	Width / °	Frames	Time / s	Wavelength / Å	Voltage / kV	Current / mA	Temp./ K
Phi	49.608	0.00	0.00	0.00	54.78	1.00	180	1.00	0.71076	50	20.0	100
Omega	49.608	12.75	-168.25	102.00	54.78	1.00	182	10.00	0.71076	50	20.0	100
Omega	49.608	12.75	-168.25	0.00	54.78	1.00	182	10.00	0.71076	50	20.0	100

A total of 544 frames were collected. The total exposure time was 1.06 hours. The frames were integrated with the Bruker SAINT software package using a narrow-frame algorithm. The integration of the data using a monoclinic unit cell yielded a total of 18422 reflections to a maximum θ angle of 28.31° (0.75 Å resolution), of which 3777 were independent (average redundancy 4.877, completeness = 99.9%, R_{int} = 2.68%, R_{sig} = 2.15%) and 3232 (85.57%) were greater than $2\sigma(F^2)$. The final cell constants of a = 7.25910(10) Å, b = 10.4943(2) Å, c = 20.0152(4) Å, β = 90.2870(10)°, volume = 1524.72(5) Å^3, are based upon the refinement of the XYZ-centroids of 9214 reflections above 20 σ(I) with 5.625° < 2θ < 56.57°. Data were corrected for absorption effects using the Multi-Scan method (SADABS). The ratio of minimum to maximum apparent transmission was 0.923. The calculated minimum and maximum transmission coefficients (based on crystal size) are 0.9780 and 0.9820.

The structure was solved and refined using the Bruker SHELXTL Software Package, using the space group P 1 21/n 1, with Z = 4 for the formula unit, $C_{17}H_{20}N_2O_4$. The final anisotropic full-matrix least-squares refinement on F^2 with 234 variables converged at R1 = 4.40%, for the observed data and wR2 = 10.72% for all data. The goodness-of-fit was 1.055. The largest peak in the final difference electron density synthesis was 0.415 $e^-/Å^3$ and the largest hole was -0.195 $e^-/Å^3$ with an RMS deviation of 0.048 $e^-/Å^3$. On the basis of the final model, the calculated density was 1.378 g/cm^3 and F(000), 672 e^-.

Table 2. Sample and crystal data for GR024.

**Identification code**	GR024	
**Chemical formula**	$C_{17}H_{20}N_2O_4$	
**Formula weight**	316.35 g/mol	
**Temperature**	100(2) K	
**Wavelength**	0.71073 Å	
**Crystal size**	0.180 x 0.180 x 0.230 mm	
**Crystal habit**	colorless block	
**Crystal system**	monoclinic	
**Space group**	P 1 21/n 1	
**Unit cell dimensions**	a = 7.25910(10) Å	α = 90°
	b = 10.4943(2) Å	β = 90.2870(10)°
	c = 20.0152(4) Å	γ = 90°
**Volume**	1524.72(5) $Å^3$	
**Z**	4	
**Density (calculated)**	1.378 g/cm^3	
**Absorption coefficient**	0.099 mm^{-1}	
**F(000)**	672	

Table 3. Data collection and structure refinement for GR024.

**Diffractometer**	D8 QUEST ECO three-circle diffractometer	
**Radiation source**	Ceramic x-ray tube (Mo Kα, λ = 0.71073 Å)	
**Theta range for data collection**	2.99 to 28.31°	
**Index ranges**	-9<=h<=8, -13<=k<=14, -26<=l<=23	
**Reflections collected**	18422	
**Independent reflections**	3777 [R(int) = 0.0268]	
**Coverage of independent reflections**	99.9%	
**Absorption correction**	Multi-Scan	
**Max. and min. transmission**	0.9820 and 0.9780	
**Structure solution technique**	direct methods	
**Structure solution program**	XT, VERSION 2018/2	
**Refinement method**	Full-matrix least-squares on F^2	
**Refinement program**	SHELXL-2019/1 (Sheldrick, 2019)	
**Function minimized**	$\Sigma w(F_o^2 - F_c^2)^2$	
**Data / restraints / parameters**	3777 / 0 / 234	
**Goodness-of-fit on F^2**	1.055	
**Final R indices**	3232 data; I>2σ(I)	R1 = 0.0440, wR2 = 0.1022
	all data	R1 = 0.0532, wR2 = 0.1072
**Weighting scheme**	$w=1/[\sigma^2(F_o^2)+(0.0446P)^2+0.9330P]$<br>where $P=(F_o^2+2F_c^2)/3$	
**Largest diff. peak and hole**	0.415 and -0.195 $eÅ^{-3}$	
**R.M.S. deviation from mean**	0.048 $eÅ^{-3}$	

Table 4. Atomic coordinates and equivalent isotropic atomic displacement parameters ($Å^2$) for GR024.

U(eq) is defined as one third of the trace of the orthogonalized U_{ij} tensor.

	x/a	y/b	z/c	U(eq)
O7	0.09225(13)	0.62894(9)	0.62102(5)	0.0203(2)
O11	0.65729(12)	0.92502(9)	0.56066(5)	0.0171(2)
O13	0.11423(15)	0.45037(10)	0.56030(6)	0.0263(2)
O22	0.97798(15)	0.79630(10)	0.87888(5)	0.0259(2)
N3	0.35972(15)	0.87256(10)	0.53539(6)	0.0144(2)
N9	0.36074(15)	0.75157(11)	0.65448(5)	0.0159(2)
C1	0.51584(17)	0.73424(12)	0.60769(6)	0.0147(3)
C2	0.52251(17)	0.85489(12)	0.56631(6)	0.0136(2)
C4	0.21825(17)	0.77630(12)	0.54513(6)	0.0136(2)
C5	0.28794(18)	0.64018(12)	0.52869(7)	0.0156(3)
C6	0.15749(18)	0.55945(13)	0.56876(7)	0.0179(3)
C8	0.18274(17)	0.75592(13)	0.62102(7)	0.0160(3)
C10	0.48457(18)	0.62379(12)	0.55840(7)	0.0170(3)
C12	0.04787(18)	0.81189(13)	0.50523(7)	0.0179(3)
C14	0.05290(19)	0.84709(15)	0.65519(7)	0.0229(3)
C15	0.3694(2)	0.66726(14)	0.71328(7)	0.0212(3)
C16	0.53081(19)	0.69952(14)	0.75793(7)	0.0192(3)
C17	0.6719(2)	0.61266(14)	0.76749(7)	0.0220(3)
C18	0.8250(2)	0.64011(14)	0.80772(7)	0.0210(3)
C19	0.83411(19)	0.75752(14)	0.83900(7)	0.0197(3)
C20	0.6914(2)	0.84446(14)	0.83135(7)	0.0219(3)
C21	0.5421(2)	0.81623(14)	0.79093(7)	0.0218(3)
C23	0.1228(2)	0.70737(15)	0.89016(8)	0.0244(3)

Table 5. Bond lengths (Å) for GR024.

O7-C6	1.3620(17)	O7-C8	1.4856(16)
O11-C2	1.2300(16)	O13-C6	1.1989(17)
O22-C19	1.3729(16)	O22-C23	1.4231(18)
N3-C2	1.3440(16)	N3-C4	1.4543(16)
N3-H3	0.91(2)	N9-C8	1.4531(16)
N9-C15	1.4736(17)	N9-C1	1.4792(16)
C1-C2	1.5140(18)	C1-C10	1.5383(18)
C1-H1	1.000000	C4-C12	1.5157(17)
C4-C5	1.5513(17)	C4-C8	1.5566(18)
C5-C6	1.5051(18)	C5-C10	1.5531(18)
C5-H5	1.000000	C8-C14	1.5093(19)
C10-H10A	0.990000	C10-H10B	0.990000
C12-H12A	0.980000	C12-H12B	0.980000
C12-H12C	0.980000	C14-H14A	0.980000
C14-H14B	0.980000	C14-H14C	0.980000
C15-C16	1.5083(19)	C15-H15A	0.990000
C15-H15B	0.990000	C16-C17	1.384(2)
C16-C21	1.394(2)	C17-C18	1.399(2)
C17-H17	0.950000	C18-C19	1.384(2)
C18-H18	0.950000	C19-C20	1.389(2)
C20-C21	1.381(2)	C20-H20	0.950000
C21-H21	0.950000	C23-H23A	0.980000
C23-H23B	0.980000	C23-H23C	0.980000

Table 6. Bond angles (°) for GR024.

C6-O7-C8	108.95(10)	C19-O22-C23	117.21(12)
C2-N3-C4	117.54(11)	C2-N3-H3	121.5(12)
C4-N3-H3	120.5(12)	C8-N9-C15	114.92(10)
C8-N9-C1	112.95(10)	C15-N9-C1	113.71(11)
N9-C1-C2	105.67(10)	N9-C1-C10	112.85(10)
C2-C1-C10	106.51(10)	N9-C1-H1	110.500000
C2-C1-H1	110.500000	C10-C1-H1	110.500000
O11-C2-N3	124.97(12)	O11-C2-C1	125.34(11)
N3-C2-C1	109.68(11)	N3-C4-C12	109.46(10)
N3-C4-C5	112.33(10)	C12-C4-C5	112.41(10)
N3-C4-C8	110.28(10)	C12-C4-C8	114.16(11)
C5-C4-C8	97.83(10)	C6-C5-C4	101.45(10)
C6-C5-C10	108.25(10)	C4-C5-C10	108.71(10)
C6-C5-H5	112.600000	C4-C5-H5	112.600000
C10-C5-H5	112.600000	O13-C6-O7	121.87(13)
O13-C6-C5	128.85(13)	O7-C6-C5	109.27(11)
N9-C8-O7	111.35(10)	N9-C8-C14	111.52(11)
O7-C8-C14	106.94(11)	N9-C8-C4	107.63(10)
O7-C8-C4	101.46(10)	C14-C8-C4	117.51(11)
C1-C10-C5	107.13(10)	C1-C10-H10A	110.300000
C5-C10-H10A	110.300000	C1-C10-H10B	110.300000
C5-C10-H10B	110.300000	H10A-C10-H10B	108.500000
C4-C12-H12A	109.500000	C4-C12-H12B	109.500000
H12A-C12-H12B	109.500000	C4-C12-H12C	109.500000
H12A-C12-H12C	109.500000	H12B-C12-H12C	109.500000
C8-C14-H14A	109.500000	C8-C14-H14B	109.500000
H14A-C14-H14B	109.500000	C8-C14-H14C	109.500000
H14A-C14-H14C	109.500000	H14B-C14-H14C	109.500000
N9-C15-C16	111.62(11)	N9-C15-H15A	109.300000
C16-C15-H15A	109.300000	N9-C15-H15B	109.300000
C16-C15-H15B	109.300000	H15A-C15-H15B	108.000000
C17-C16-C21	118.15(13)	C17-C16-C15	120.41(13)
C21-C16-C15	121.44(13)	C16-C17-C18	121.95(14)
C16-C17-H17	119.000000	C18-C17-H17	119.000000
C19-C18-C17	118.68(13)	C19-C18-H18	120.700000
C17-C18-H18	120.700000	O22-C19-C18	124.17(13)
O22-C19-C20	115.75(13)	C18-C19-C20	120.08(13)
C21-C20-C19	120.43(13)	C21-C20-H20	119.800000
C19-C20-H20	119.800000	C20-C21-C16	120.67(14)
C20-C21-H21	119.700000	C16-C21-H21	119.700000
O22-C23-H23A	109.500000	O22-C23-H23B	109.500000
H23A-C23-H23B	109.500000	O22-C23-H23C	109.500000
H23A-C23-H23C	109.500000	H23B-C23-H23C	109.500000

Table 7. Torsion angles (°) for GR024.

C8-N9-C1-C2	66.54(13)	C15-N9-C1-C2	-160.16(10)
C8-N9-C1-C10	-49.47(14)	C15-N9-C1-C10	83.83(13)
C4-N3-C2-O11	176.87(12)	C4-N3-C2-C1	-1.84(15)

N9-C1-C2-O11	123.78(13)	C10-C1-C2-O11	-115.96(14)
N9-C1-C2-N3	-57.52(13)	C10-C1-C2-N3	62.73(13)
C2-N3-C4-C12	-178.76(11)	C2-N3-C4-C5	-53.15(15)
C2-N3-C4-C8	54.85(14)	N3-C4-C5-C6	156.84(10)
C12-C4-C5-C6	-79.18(12)	C8-C4-C5-C6	41.06(11)
N3-C4-C5-C10	42.89(14)	C12-C4-C5-C10	166.88(11)
C8-C4-C5-C10	-72.88(11)	C8-O7-C6-O13	175.27(12)
C8-O7-C6-C5	-3.22(14)	C4-C5-C6-O13	156.36(14)
C10-C5-C6-O13	-89.34(17)	C4-C5-C6-O7	-25.29(13)
C10-C5-C6-O7	89.00(12)	C15-N9-C8-O7	-36.97(15)
C1-N9-C8-O7	95.74(12)	C15-N9-C8-C14	82.40(14)
C1-N9-C8-C14	-144.89(12)	C15-N9-C8-C4	-147.33(11)
C1-N9-C8-C4	-14.62(14)	C6-O7-C8-N9	-83.95(12)
C6-O7-C8-C14	153.99(11)	C6-O7-C8-C4	30.32(12)
N3-C4-C8-N9	-43.52(13)	C12-C4-C8-N9	-167.23(11)
C5-C4-C8-N9	73.85(11)	N3-C4-C8-O7	-160.52(10)
C12-C4-C8-O7	75.76(12)	C5-C4-C8-O7	-43.15(11)
N3-C4-C8-C14	83.32(14)	C12-C4-C8-C14	-40.39(16)
C5-C4-C8-C14	-159.31(11)	N9-C1-C10-C5	48.23(13)
C2-C1-C10-C5	-67.27(12)	C6-C5-C10-C1	-95.14(12)
C4-C5-C10-C1	14.27(14)	C8-N9-C15-C16	-160.96(12)
C1-N9-C15-C16	66.69(15)	N9-C15-C16-C17	-115.75(14)
N9-C15-C16-C21	64.68(17)	C21-C16-C17-C18	-1.4(2)
C15-C16-C17-C18	179.00(13)	C16-C17-C18-C19	0.4(2)
C23-O22-C19-C18	-2.3(2)	C23-O22-C19-C20	177.31(13)
C17-C18-C19-O22	-179.06(13)	C17-C18-C19-C20	1.4(2)
O22-C19-C20-C21	178.38(13)	C18-C19-C20-C21	-2.0(2)
C19-C20-C21-C16	0.9(2)	C17-C16-C21-C20	0.8(2)
C15-C16-C21-C20	-179.65(13)		

Table 8. Anisotropic atomic displacement parameters (Å^2) for GR024.

The anisotropic atomic displacement factor exponent takes the form: $-2\pi^2[h^2 a^{*2} U_{11} + ... + 2 h k a^* b^* U_{12}]$

	U_{11}	U_{22}	U_{33}	U_{23}	U_{13}	U_{12}
O7	0.0191(5)	0.0211(5)	0.0208(5)	0.0024(4)	0.0007(4)	-0.0080(4)
O11	0.0133(4)	0.0172(5)	0.0209(5)	0.0035(4)	-0.0009(3)	-0.0029(3)
O13	0.0288(6)	0.0168(5)	0.0332(6)	0.0025(4)	-0.0057(4)	-0.0072(4)
O22	0.0257(5)	0.0260(5)	0.0261(5)	-0.0029(4)	-0.0086(4)	-0.0009(4)
N3	0.0127(5)	0.0128(5)	0.0175(5)	0.0032(4)	-0.0005(4)	-0.0012(4)
N9	0.0138(5)	0.0205(6)	0.0133(5)	0.0021(4)	0.0001(4)	-0.0023(4)
C1	0.0128(6)	0.0153(6)	0.0161(6)	0.0027(5)	-0.0006(4)	-0.0005(5)
C2	0.0130(6)	0.0136(6)	0.0142(6)	-0.0014(5)	0.0011(4)	0.0010(4)
C4	0.0121(5)	0.0124(6)	0.0162(6)	0.0003(5)	-0.0002(4)	-0.0011(4)
C5	0.0180(6)	0.0126(6)	0.0163(6)	0.0000(5)	-0.0016(5)	-0.0007(5)
C6	0.0179(6)	0.0166(6)	0.0192(6)	0.0024(5)	-0.0043(5)	-0.0027(5)
C8	0.0138(6)	0.0170(6)	0.0172(6)	0.0001(5)	0.0008(5)	-0.0041(5)
C10	0.0174(6)	0.0136(6)	0.0200(6)	0.0013(5)	0.0008(5)	0.0020(5)
C12	0.0148(6)	0.0162(6)	0.0226(7)	0.0007(5)	-0.0041(5)	-0.0010(5)
C14	0.0161(6)	0.0286(7)	0.0240(7)	-0.0058(6)	0.0032(5)	0.0008(5)
C15	0.0227(7)	0.0250(7)	0.0160(6)	0.0046(5)	-0.0006(5)	-0.0075(5)
C16	0.0219(7)	0.0224(7)	0.0132(6)	0.0029(5)	0.0004(5)	-0.0055(5)

C17	0.0291(7)	0.0183(7)	0.0186(6)	0.0001(5)	-0.0015(5)	-0.0037(6)
C18	0.0237(7)	0.0198(7)	0.0193(7)	0.0022(5)	-0.0011(5)	0.0010(5)
C19	0.0224(7)	0.0236(7)	0.0132(6)	0.0023(5)	-0.0009(5)	-0.0039(5)
C20	0.0268(7)	0.0208(7)	0.0180(6)	-0.0027(5)	-0.0007(5)	-0.0016(6)
C21	0.0225(7)	0.0233(7)	0.0197(7)	0.0015(5)	-0.0006(5)	0.0020(6)
C23	0.0206(7)	0.0304(8)	0.0220(7)	-0.0014(6)	-0.0003(5)	-0.0009(6)

Table 9. Hydrogen atomic coordinates and isotropic atomic displacement parameters (Å^2) for GR024.

	x/a	y/b	z/c	U(eq)
H3	0.343(3)	0.9362(19)	0.5053(10)	0.031(5)
H1	0.6340	0.7222	0.6328	0.017(4)
H5	0.2824	0.6207	0.4798	0.021(4)
H10A	0.4950	0.5410	0.5818	0.020(4)
H10B	0.5775	0.6264	0.5224	0.027(5)
H12A	-0.0474	0.7470	0.5117	0.027(5)
H12B	0.0790	0.8170	0.4577	0.023(4)
H12C	0.0019	0.8947	0.5204	0.026(5)
H14A	0.1022	0.9338	0.6523	0.032(5)
H14B	0.0401	0.8228	0.7022	0.029(5)
H14C	-0.0679	0.8438	0.6332	0.033(5)
H15A	0.3803	0.5777	0.6983	0.022(4)
H15B	0.2539	0.6754	0.7390	0.027(5)
H17	0.6646	0.5320	0.7461	0.030(5)
H18	0.9208	0.5793	0.8134	0.030(5)
H20	0.6964	0.9239	0.8541	0.031(5)
H21	0.4461	0.8770	0.7856	0.030(5)
H23A	1.2167	0.7460	0.9191	0.038(5)
H23B	1.1784	0.6838	0.8474	0.030(5)
H23C	1.0732	0.6310	0.9118	0.035(5)

8-(4-Methoxybenzyl)-7,7a-dimethyl-4a-phenyl-3,4-dihydro-3,7-epiminofuro[3,4-*b*]pyridine-2,5(1*H*)-dione (24)

ORTEP representation showing thermal ellipsoids at the 50% probability level:

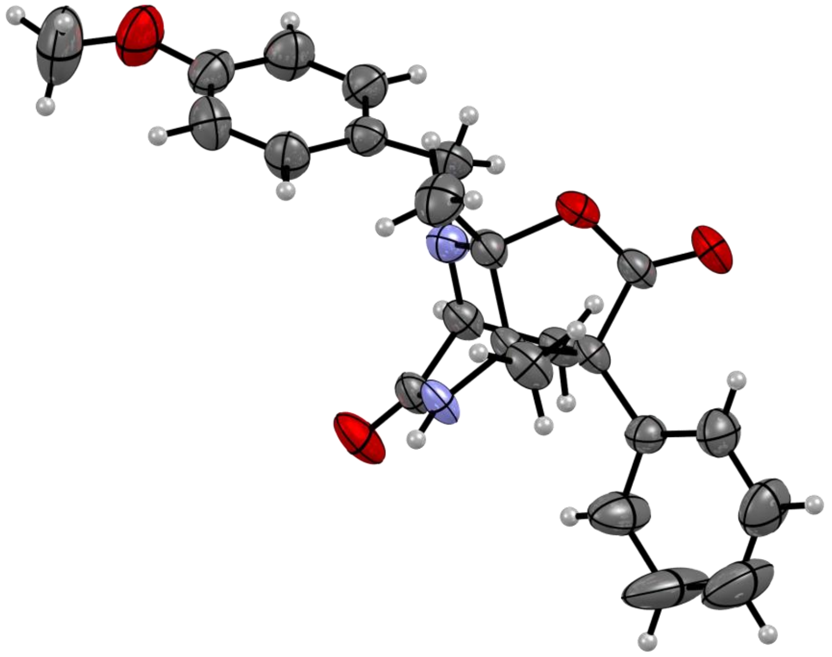

Crystal Structure Report for GR025

A colorless, block-like specimen of $C_{24}H_{25.26}Cl_{2.74}N_2O_4$, approximate dimensions 0.240 mm x 0.300 mm x 0.300 mm, was used for the X-ray crystallographic analysis. The X-ray intensity data were measured on a D8 QUEST ECO three-circle diffractometer system equipped with a Ceramic x-ray tube (Mo Kα, λ = 0.71073 Å) and a doubly curved silicon crystal Bruker Triumph monochromator.

Table 1: Data collection details for GR008_222.

Axis	dx/mm	2θ/°	ω/°	φ/°	χ/°	Width/°	Frames	Time/s	Wavelength/Å	Voltage/kV	Current/mA	Temp./K
Phi	59.688	12.07	13.07	0.00	54.76	1.20	300	10.00	0.71076	50	20.0	301
Phi	59.688	12.07	-168.93	0.00	54.76	1.20	300	10.00	0.71076	50	20.0	301

A total of 600 frames were collected. The total exposure time was 1.67 hours. The frames were integrated with the Bruker SAINT software package using a narrow-frame algorithm. The integration of the data using a monoclinic unit cell yielded a total of 46448 reflections to a maximum θ angle of 25.36° (0.83 Å resolution), of which 4559 were independent (average redundancy 10.188, completeness = 99.4%, R_{int} = 4.66%, R_{sig} = 2.27%) and 3460 (75.89%) were greater than $2\sigma(F^2)$. The final cell constants of a = 12.9829(13) Å, b = 7.8205(8) Å, c = 25.207(2) Å, β = 103.510(3)°, volume = 2488.5(4) $Å^3$, are based upon the refinement of the XYZ-centroids of 9926 reflections above 20 σ(I) with 5.468° < 2θ < 48.50°. Data were corrected for absorption effects using the Multi-Scan method (SADABS). The ratio of minimum to maximum apparent transmission was 0.856. The calculated minimum and maximum transmission coefficients (based on crystal size) are 0.8960 and 0.9160.

The structure was solved and refined using the Bruker SHELXTL Software Package, using the space group P 1 21/n 1, with Z = 4 for the formula unit, $C_{24}H_{25.26}Cl_{2.74}N_2O_4$. The final anisotropic full-matrix least-squares refinement on F^2 with 321 variables converged at R1 = 9.97%, for the observed data and wR2 = 35.72% for all data. The goodness-of-fit was 1.480. The largest peak in the final difference electron density synthesis was 1.010 $e^-/Å^3$ and the largest hole was -0.871 $e^-/Å^3$ with an RMS deviation of 0.085 $e^-/Å^3$. On the basis of the final model, the calculated density was 1.343 g/cm^3 and

F(000), 1048 e^-.

Table 2. Sample and crystal data for GR025.

**Identification code**	GR025	
**Chemical formula**	$C_{24}H_{25.26}Cl_{2.74}N_2O_4$	
**Formula weight**	503.00 g/mol	
**Temperature**	301(2) K	
**Wavelength**	0.71073 Å	
**Crystal size**	0.240 x 0.300 x 0.300 mm	
**Crystal habit**	colorless block	
**Crystal system**	monoclinic	
**Space group**	P 1 21/n 1	
**Unit cell dimensions**	a = 12.9829(13) Å	α = 90°
	b = 7.8205(8) Å	β = 103.510(3)°
	c = 25.207(2) Å	γ = 90°
**Volume**	2488.5(4) $Å^3$	
**Z**	4	
**Density (calculated)**	1.343 g/cm^3	
**Absorption coefficient**	0.373 mm^{-1}	
**F(000)**	1048	

Table 3. Data collection and structure refinement for GR025.

**Diffractometer**	D8 QUEST ECO three-circle diffractometer	
**Radiation source**	Ceramic x-ray tube (Mo Kα, λ = 0.71073 Å)	
**Theta range for data collection**	3.06 to 25.36°	
**Index ranges**	-15<=h<=15, -9<=k<=9, -30<=l<=30	
**Reflections collected**	46448	
**Independent reflections**	4559 [R(int) = 0.0466]	
**Coverage of independent reflections**	99.4%	
**Absorption correction**	Multi-Scan	
**Max. and min. transmission**	0.9160 and 0.8960	
**Structure solution technique**	direct methods	
**Structure solution program**	XT, VERSION 2018/2	
**Refinement method**	Full-matrix least-squares on F^2	
**Refinement program**	SHELXL-2019/1 (Sheldrick, 2019)	
**Function minimized**	Σ w(F_o^2 - F_c^2)^2	
**Data / restraints / parameters**	4559 / 0 / 321	
**Goodness-of-fit on F^2**	1.480	
**Final R indices**	3460 data; I>2σ(I)	R1 = 0.0997, wR2 = 0.3240
	all data	R1 = 0.1223, wR2 = 0.3572
**Weighting scheme**	w=1/[$\sigma^2(F_o^2)+(0.2000P)^2$]<br>where P=($F_o^2+2F_c^2$)/3	
**Largest diff. peak and hole**	1.010 and -0.871 $eÅ^{-3}$	
**R.M.S. deviation from mean**	0.085 $eÅ^{-3}$	

Table 4. Atomic coordinates and equivalent isotropic atomic displacement parameters ($Å^2$) for GR025.

U(eq) is defined as one third of the trace of the orthogonalized U_{ij} tensor.

	x/a	y/b	z/c	U(eq)
Cl2S	0.2147(4)	0.8517(7)	0.57537(16)	0.1596(18)
Cl3S	0.1875(3)	0.1097(9)	0.4956(2)	0.217(3)

	x/a	y/b	z/c	U(eq)
Cl4S	0.37142(18)	0.9029(3)	0.51810(11)	0.1016(10)
C1S	0.2777(9)	0.9953(15)	0.5443(5)	0.132(5)
Cl2'	0.2408(10)	0.7392(19)	0.5799(6)	0.214(9)
C1'	0.1341(15)	0.857(3)	0.5754(6)	0.132(5)
Cl3'	0.1119(9)	0.9786(16)	0.5216(5)	0.160(5)
O1	0.5315(3)	0.6932(4)	0.47003(10)	0.0639(9)
O2	0.3531(2)	0.4454(4)	0.27615(9)	0.0482(7)
O3	0.1827(2)	0.5094(4)	0.25548(11)	0.0587(8)
O4	0.9341(3)	0.9678(5)	0.32152(17)	0.0808(11)
N3	0.4471(2)	0.4625(4)	0.42443(11)	0.0431(8)
N8	0.4955(3)	0.5994(4)	0.33607(12)	0.0457(8)
C1	0.4516(3)	0.7082(5)	0.37285(14)	0.0410(9)
C2	0.4826(3)	0.6235(5)	0.42811(14)	0.0419(9)
C4	0.4433(3)	0.4379(5)	0.32579(14)	0.0418(9)
C5	0.3844(3)	0.4043(4)	0.37185(13)	0.0372(8)
C6	0.2867(3)	0.5268(5)	0.35043(13)	0.0382(8)
C7	0.3293(3)	0.7134(4)	0.35715(14)	0.0398(8)
C9	0.5160(4)	0.2981(6)	0.3143(2)	0.0659(12)
C10	0.3527(4)	0.2205(5)	0.37598(16)	0.0510(10)
C11	0.2638(3)	0.4924(5)	0.28953(14)	0.0427(9)
C12	0.1947(3)	0.4930(5)	0.37680(15)	0.0433(9)
C13	0.1993(5)	0.5558(8)	0.4285(2)	0.0792(17)
C14	0.1183(7)	0.5206(11)	0.4546(3)	0.127(3)
C15	0.0341(6)	0.4204(10)	0.4291(3)	0.112(3)
C16	0.0288(4)	0.3587(8)	0.3788(3)	0.0778(15)
C17	0.1092(3)	0.3918(6)	0.35277(19)	0.0573(11)
C18	0.5212(3)	0.6904(6)	0.29019(15)	0.0506(10)
C19	0.6308(3)	0.7673(5)	0.30399(15)	0.0447(9)
C20	0.7154(3)	0.6888(6)	0.33939(18)	0.0555(11)
C21	0.8179(4)	0.7518(6)	0.34626(19)	0.0610(11)
C22	0.8365(3)	0.8946(6)	0.31850(19)	0.0571(11)
C23	0.7523(4)	0.9768(6)	0.2845(2)	0.0637(12)
C24	0.6515(3)	0.9146(6)	0.27753(17)	0.0537(11)
C25	0.0227(5)	0.8811(11)	0.3521(4)	0.114(2)

Table 5. Bond lengths (Å) for GR025.

Cl2S-C1S	1.686(17)	Cl3S-C1S	1.734(11)
Cl4S-C1S	1.677(12)	C1S-H1S	0.980000
Cl2'-C1'	1.646(10)	C1'-Cl3'	1.626(10)
C1'-H1'A	0.970000	C1'-H1'B	0.970000
O1-C2	1.226(4)	O2-C11	1.332(5)
O2-C4	1.502(4)	O3-C11	1.201(5)
O4-C22	1.376(5)	O4-C25	1.402(8)
N3-C2	1.337(5)	N3-C5	1.457(4)
N3-H3	0.860000	N8-C4	1.429(5)
N8-C18	1.462(5)	N8-C1	1.468(5)
C1-C2	1.510(5)	C1-C7	1.545(5)
C1-H1	0.980000	C4-C9	1.516(6)
C4-C5	1.555(5)	C5-C10	1.505(5)
C5-C6	1.580(5)	C6-C11	1.518(4)

C6-C12	1.519(5)	C6-C7	1.556(5)
C7-H7A	0.970000	C7-H7B	0.970000
C9-H9A	0.960000	C9-H9B	0.960000
C9-H9C	0.960000	C10-H10A	0.960000
C10-H10B	0.960000	C10-H10C	0.960000
C12-C17	1.382(6)	C12-C13	1.381(6)
C13-C14	1.392(8)	C13-H13	0.930000
C14-C15	1.376(9)	C14-H14	0.930000
C15-C16	1.344(9)	C15-H15	0.930000
C16-C17	1.381(7)	C16-H16	0.930000
C17-H17	0.930000	C18-C19	1.508(6)
C18-H18A	0.970000	C18-H18B	0.970000
C19-C20	1.387(6)	C19-C24	1.388(6)
C20-C21	1.391(6)	C20-H20	0.930000
C21-C22	1.369(7)	C21-H21	0.930000
C22-C23	1.379(7)	C23-C24	1.369(6)
C23-H23	0.930000	C24-H24	0.930000
C25-H25A	0.960000	C25-H25B	0.960000
C25-H25C	0.960000		

Table 6. Bond angles (°) for GR025.

Cl4S-C1S-Cl2S	111.8(6)	Cl4S-C1S-Cl3S	112.1(8)
Cl2S-C1S-Cl3S	110.6(7)	Cl4S-C1S-H1S	107.400000
Cl2S-C1S-H1S	107.400000	Cl3S-C1S-H1S	107.400000
Cl3'-C1'-Cl2'	111.500000	Cl3'-C1'-H1'A	109.300000
Cl2'-C1'-H1'A	109.300000	Cl3'-C1'-H1'B	109.300000
Cl2'-C1'-H1'B	109.300000	H1'A-C1'-H1'B	108.000000
C11-O2-C4	110.8(2)	C22-O4-C25	117.1(5)
C2-N3-C5	117.9(3)	C2-N3-H3	121.100000
C5-N3-H3	121.100000	C4-N8-C18	118.3(3)
C4-N8-C1	112.9(3)	C18-N8-C1	114.3(3)
N8-C1-C2	105.6(3)	N8-C1-C7	112.4(3)
C2-C1-C7	106.5(3)	N8-C1-H1	110.700000
C2-C1-H1	110.700000	C7-C1-H1	110.700000
O1-C2-N3	125.4(3)	O1-C2-C1	125.0(3)
N3-C2-C1	109.6(3)	N8-C4-O2	111.1(3)
N8-C4-C9	112.5(3)	O2-C4-C9	105.6(3)
N8-C4-C5	108.0(3)	O2-C4-C5	101.8(3)
C9-C4-C5	117.3(3)	N3-C5-C10	109.8(3)
N3-C5-C4	111.0(3)	C10-C5-C4	113.7(3)
N3-C5-C6	110.8(3)	C10-C5-C6	113.1(3)
C4-C5-C6	97.9(3)	C11-C6-C12	115.1(3)
C11-C6-C7	104.9(3)	C12-C6-C7	114.3(3)
C11-C6-C5	100.9(3)	C12-C6-C5	113.3(3)
C7-C6-C5	107.1(3)	C1-C7-C6	108.8(3)
C1-C7-H7A	109.900000	C6-C7-H7A	109.900000
C1-C7-H7B	109.900000	C6-C7-H7B	109.900000
H7A-C7-H7B	108.300000	C4-C9-H9A	109.500000
C4-C9-H9B	109.500000	H9A-C9-H9B	109.500000
C4-C9-H9C	109.500000	H9A-C9-H9C	109.500000
H9B-C9-H9C	109.500000	C5-C10-H10A	109.500000

C5-C10-H10B	109.500000	H10A-C10-H10B	109.500000
C5-C10-H10C	109.500000	H10A-C10-H10C	109.500000
H10B-C10-H10C	109.500000	O3-C11-O2	121.3(3)
O3-C11-C6	129.4(3)	O2-C11-C6	109.2(3)
C17-C12-C13	118.1(4)	C17-C12-C6	122.7(3)
C13-C12-C6	119.1(4)	C12-C13-C14	120.3(5)
C12-C13-H13	119.800000	C14-C13-H13	119.800000
C15-C14-C13	119.9(5)	C15-C14-H14	120.100000
C13-C14-H14	120.100000	C16-C15-C14	120.3(5)
C16-C15-H15	119.800000	C14-C15-H15	119.800000
C15-C16-C17	120.2(5)	C15-C16-H16	119.900000
C17-C16-H16	119.900000	C12-C17-C16	121.2(4)
C12-C17-H17	119.400000	C16-C17-H17	119.400000
N8-C18-C19	112.9(3)	N8-C18-H18A	109.000000
C19-C18-H18A	109.000000	N8-C18-H18B	109.000000
C19-C18-H18B	109.000000	H18A-C18-H18B	107.800000
C20-C19-C24	117.6(4)	C20-C19-C18	122.3(4)
C24-C19-C18	119.9(3)	C19-C20-C21	120.9(4)
C19-C20-H20	119.500000	C21-C20-H20	119.500000
C22-C21-C20	120.2(4)	C22-C21-H21	119.900000
C20-C21-H21	119.900000	C21-C22-O4	125.5(4)
C21-C22-C23	119.2(4)	O4-C22-C23	115.2(4)
C24-C23-C22	120.7(4)	C24-C23-H23	119.700000
C22-C23-H23	119.700000	C23-C24-C19	121.3(4)
C23-C24-H24	119.400000	C19-C24-H24	119.400000
O4-C25-H25A	109.500000	O4-C25-H25B	109.500000
H25A-C25-H25B	109.500000	O4-C25-H25C	109.500000
H25A-C25-H25C	109.500000	H25B-C25-H25C	109.500000

Table 7. Torsion angles (°) for GR025.

C4-N8-C1-C2	69.4(4)	C18-N8-C1-C2	-151.6(3)
C4-N8-C1-C7	-46.4(4)	C18-N8-C1-C7	92.7(4)
C5-N3-C2-O1	176.0(4)	C5-N3-C2-C1	-2.9(5)
N8-C1-C2-O1	124.7(4)	C7-C1-C2-O1	-115.6(4)
N8-C1-C2-N3	-56.4(4)	C7-C1-C2-N3	63.3(4)
C18-N8-C4-O2	-46.0(4)	C1-N8-C4-O2	91.4(3)
C18-N8-C4-C9	72.1(4)	C1-N8-C4-C9	-150.5(3)
C18-N8-C4-C5	-156.8(3)	C1-N8-C4-C5	-19.5(4)
C11-O2-C4-N8	-89.1(3)	C11-O2-C4-C9	148.7(3)
C11-O2-C4-C5	25.7(4)	C2-N3-C5-C10	179.6(3)
C2-N3-C5-C4	53.0(4)	C2-N3-C5-C6	-54.7(4)
N8-C4-C5-N3	-38.7(4)	O2-C4-C5-N3	-155.8(3)
C9-C4-C5-N3	89.6(4)	N8-C4-C5-C10	-163.2(3)
O2-C4-C5-C10	79.8(4)	C9-C4-C5-C10	-34.9(5)
N8-C4-C5-C6	77.2(3)	O2-C4-C5-C6	-39.8(3)
C9-C4-C5-C6	-154.5(3)	N3-C5-C6-C11	156.8(3)
C10-C5-C6-C11	-79.4(3)	C4-C5-C6-C11	40.7(3)
N3-C5-C6-C12	-79.6(4)	C10-C5-C6-C12	44.1(4)
C4-C5-C6-C12	164.2(3)	N3-C5-C6-C7	47.3(3)
C10-C5-C6-C7	171.1(3)	C4-C5-C6-C7	-68.8(3)
N8-C1-C7-C6	50.7(4)	C2-C1-C7-C6	-64.5(3)

C11-C6-C7-C1	-97.4(3)	C12-C6-C7-C1	135.6(3)
C5-C6-C7-C1	9.3(3)	C4-O2-C11-O3	178.5(3)
C4-O2-C11-C6	1.8(4)	C12-C6-C11-O3	33.3(6)
C7-C6-C11-O3	-93.1(5)	C5-C6-C11-O3	155.6(4)
C12-C6-C11-O2	-150.3(3)	C7-C6-C11-O2	83.3(3)
C5-C6-C11-O2	-27.9(4)	C11-C6-C12-C17	18.9(5)
C7-C6-C12-C17	140.4(4)	C5-C6-C12-C17	-96.5(4)
C11-C6-C12-C13	-165.6(4)	C7-C6-C12-C13	-44.1(5)
C5-C6-C12-C13	79.0(5)	C17-C12-C13-C14	-1.6(9)
C6-C12-C13-C14	-177.3(6)	C12-C13-C14-C15	1.1(13)
C13-C14-C15-C16	-1.1(14)	C14-C15-C16-C17	1.6(12)
C13-C12-C17-C16	2.1(7)	C6-C12-C17-C16	177.6(5)
C15-C16-C17-C12	-2.1(9)	C4-N8-C18-C19	-136.9(4)
C1-N8-C18-C19	86.4(4)	N8-C18-C19-C20	34.7(5)
N8-C18-C19-C24	-150.6(4)	C24-C19-C20-C21	-2.6(6)
C18-C19-C20-C21	172.2(4)	C19-C20-C21-C22	0.9(7)
C20-C21-C22-O4	-179.4(4)	C20-C21-C22-C23	1.2(7)
C25-O4-C22-C21	5.4(8)	C25-O4-C22-C23	-175.1(5)
C21-C22-C23-C24	-1.5(7)	O4-C22-C23-C24	179.0(4)
C22-C23-C24-C19	-0.3(7)	C20-C19-C24-C23	2.3(6)
C18-C19-C24-C23	-172.6(4)		

Table 8. Anisotropic atomic displacement parameters (Å^2) for GR025.

The anisotropic atomic displacement factor exponent takes the form: $-2\pi^2[h^2 a^{*2} U_{11} + ... + 2 h k a^* b^* U_{12}]$

	U_{11}	U_{22}	U_{33}	U_{23}	U_{13}	U_{12}
Cl2S	0.206(4)	0.166(4)	0.130(3)	-0.013(3)	0.085(3)	-0.001(3)
Cl3S	0.119(3)	0.329(7)	0.200(4)	0.131(5)	0.031(3)	0.089(4)
Cl4S	0.0893(16)	0.0810(15)	0.1239(19)	-0.0257(12)	0.0038(13)	-0.0144(11)
C1S	0.114(7)	0.112(7)	0.129(8)	-0.076(7)	-0.052(6)	0.040(6)
Cl2'	0.160(10)	0.170(11)	0.227(13)	0.093(10)	-0.131(10)	-0.097(9)
C1'	0.114(7)	0.112(7)	0.129(8)	-0.076(7)	-0.052(6)	0.040(6)
Cl3'	0.130(7)	0.169(10)	0.157(9)	-0.049(7)	-0.014(7)	0.022(7)
O1	0.085(2)	0.0583(18)	0.0352(15)	-0.0034(12)	-0.0117(14)	-0.0256(16)
O2	0.0534(16)	0.0614(17)	0.0275(13)	-0.0061(11)	0.0050(11)	0.0000(13)
O3	0.0542(17)	0.079(2)	0.0324(14)	0.0016(12)	-0.0099(12)	0.0008(15)
O4	0.052(2)	0.089(3)	0.101(3)	0.008(2)	0.0163(19)	-0.0171(18)
N3	0.0523(19)	0.0414(17)	0.0272(14)	0.0064(12)	-0.0076(13)	-0.0080(14)
N8	0.0478(18)	0.0537(19)	0.0361(16)	0.0022(13)	0.0106(14)	-0.0044(15)
C1	0.048(2)	0.0395(18)	0.0323(17)	0.0017(14)	0.0022(15)	-0.0069(16)
C2	0.0406(19)	0.047(2)	0.0331(18)	0.0012(15)	-0.0012(15)	-0.0111(16)
C4	0.041(2)	0.048(2)	0.0333(18)	-0.0023(15)	0.0022(15)	0.0024(16)
C5	0.0420(19)	0.0398(19)	0.0251(16)	-0.0012(13)	-0.0017(14)	-0.0023(15)
C6	0.0395(19)	0.0435(19)	0.0278(17)	-0.0055(13)	0.0000(14)	-0.0033(15)
C7	0.050(2)	0.0368(18)	0.0306(16)	0.0017(13)	0.0049(15)	-0.0019(16)
C9	0.061(3)	0.060(3)	0.078(3)	-0.011(2)	0.019(2)	0.007(2)
C10	0.066(3)	0.0379(19)	0.044(2)	-0.0007(16)	0.0027(19)	-0.0036(18)
C11	0.046(2)	0.048(2)	0.0286(17)	-0.0008(14)	-0.0018(16)	-0.0055(16)
C12	0.043(2)	0.049(2)	0.0393(19)	-0.0063(15)	0.0129(16)	-0.0036(16)
C13	0.093(4)	0.092(4)	0.062(3)	-0.033(3)	0.037(3)	-0.039(3)
C14	0.159(7)	0.149(7)	0.109(5)	-0.062(5)	0.104(5)	-0.078(6)
C15	0.101(5)	0.138(6)	0.122(6)	-0.037(5)	0.078(5)	-0.045(5)

	U_{11}	U_{22}	U_{33}	U_{23}	U_{13}	U_{12}
C16	0.056(3)	0.083(3)	0.097(4)	-0.011(3)	0.023(3)	-0.024(3)
C17	0.049(2)	0.065(3)	0.057(2)	-0.011(2)	0.0096(19)	-0.012(2)
C18	0.048(2)	0.067(3)	0.0368(19)	0.0091(17)	0.0088(17)	-0.0027(19)
C19	0.048(2)	0.047(2)	0.0403(19)	0.0045(15)	0.0126(16)	0.0059(16)
C20	0.052(2)	0.057(3)	0.056(2)	0.0154(19)	0.009(2)	-0.0027(19)
C21	0.050(2)	0.066(3)	0.061(3)	0.003(2)	0.000(2)	0.005(2)
C22	0.048(2)	0.062(3)	0.064(3)	-0.010(2)	0.020(2)	-0.009(2)
C23	0.061(3)	0.061(3)	0.071(3)	0.012(2)	0.018(2)	-0.007(2)
C24	0.052(2)	0.055(2)	0.055(2)	0.0133(18)	0.0161(19)	0.0093(19)
C25	0.053(3)	0.129(6)	0.148(6)	0.012(5)	0.000(4)	-0.012(4)

Table 9. Hydrogen atomic coordinates and isotropic atomic displacement parameters (Å^2) for GR025.

	x/a	y/b	z/c	U(eq)
H1S	0.3133	1.0768	0.5721	0.158000
H1'A	0.0735	0.7833	0.5739	0.158000
H1'B	0.1429	0.9281	0.6077	0.158000
H3	0.4609	0.3954	0.4522	0.052000
H1	0.4810	0.8240	0.3743	0.049000
H7A	0.3051	0.7756	0.3232	0.048000
H7B	0.3030	0.7713	0.3854	0.048000
H9A	0.5706	0.2766	0.3465	0.099000
H9B	0.5477	0.3338	0.2853	0.099000
H9C	0.4759	0.1955	0.3037	0.099000
H10A	0.3090	0.1842	0.3417	0.077000
H10B	0.3140	0.2097	0.4039	0.077000
H10C	0.4151	0.1504	0.3850	0.077000
H13	0.2568	0.6218	0.4460	0.095000
H14	0.1211	0.5647	0.4891	0.152000
H15	-0.0195	0.3954	0.4468	0.134000
H16	-0.0292	0.2934	0.3614	0.093000
H17	0.1058	0.3452	0.3185	0.069000
H18A	0.4697	0.7808	0.2785	0.061000
H18B	0.5158	0.6117	0.2599	0.061000
H20	0.7035	0.5926	0.3588	0.067000
H21	0.8740	0.6967	0.3698	0.073000
H23	0.7642	1.0754	0.2662	0.076000
H24	0.5957	0.9721	0.2546	0.064000
H25A	1.0860	0.9410	0.3498	0.170000
H25B	1.0246	0.7673	0.3381	0.170000
H25C	1.0182	0.8754	0.3896	0.170000
